# Landscape of transcriptome variations uncovering known and novel driver events in colorectal carcinoma

**DOI:** 10.1038/s41598-019-57311-z

**Published:** 2020-01-16

**Authors:** Giovanna Pira, Paolo Uva, Antonio Mario Scanu, Paolo Cossu Rocca, Luciano Murgia, Elena Uleri, Claudia Piu, Alberto Porcu, Ciriaco Carru, Alessandra Manca, Ivana Persico, Maria Rosaria Muroni, Francesca Sanges, Caterina Serra, Antonia Dolei, Andrea Angius, Maria Rosaria De Miglio

**Affiliations:** 1 Department of Biomedical Sciences, University of Sassari, Viale San Pietro 43-b, 07100 Sassari, Italy; 2 CRS4, Science and Technology Park Polaris, Piscina Manna, 09010 Pula, CA Italy; 3 Department of Medical, Surgical and Experimental Sciences, University of Sassari, Viale San Pietro 8, 07100 Sassari, Italy; 4 Department of Diagnostic Services, “Giovanni Paolo II” Hospital, ASSL Olbia-ATS Sardegna, Via Bazzoni-Sircana, 07026 Olbia, Italy; 5 Department of Pathology, AOU Sassari, Via Matteotti 60, 07100 Sassari, Italy; 6 Istituto di Ricerca Genetica e Biomedica (IRGB), CNR, Cittadella Universitaria di Cagliari, 09042 Monserrato, CA Italy

**Keywords:** Colorectal cancer, Molecular medicine

## Abstract

We focused on an integrated view of genomic changes in Colorectal cancer (CRC) and distant normal colon tissue (NTC) to test the effectiveness of expression profiling on identification of molecular targets. We performed transcriptome on 16 primary coupled CRC and NTC tissues. We identified pathways and networks related to pathophysiology of CRC and selected potential therapeutic targets. CRC cells have multiple ways to reprogram its transcriptome: a functional enrichment analysis in 285 genes, 25% mutated, showed that they control the major cellular processes known to promote tumorigenesis. Among the genes showing alternative splicing, cell cycle related genes were upregulated (CCND1, CDC25B, MCM2, MCM3), while genes involved in fatty acid metabolism (ACAAA2, ACADS, ACAT1, ACOX, CPT1A, HMGCS2) were downregulated. Overall 148 genes showed differential splicing identifying 17 new isoforms. Most of them are involved in the pathogenesis of CRC, although the functions of these variants remain unknown. We identified 2 in-frame fusion events, KRT19-KRT18 and EEF1A1-HSP90AB1, encoding for chemical proteins in two CRC patients. We draw a functional interactome map involving integrated multiple genomic features in CRC. Finally, we underline that two functional cell programs are prevalently deregulated and absolutely crucial to determinate and sustain CRC phenotype.

## Introduction

Colorectal cancer is the second most common cancer in women and the third in men, and is the fourth most frequent source of cancer-related mortality^1^. Metastatic CRC (mCRC) is mainly not a curable disease^2^.

The opportunity to identify specific biomarkers and to detect unambiguous molecular targets, associated to early signs of cancer, will be instrumental to develop new targeted therapies and to reduce mortality for CRC. Molecular alterations involved in the biology of CRC were described as two distinct genetic pathways. The majority of sporadic cases of CRC (up to 85%) display chromosomal instability (CIN) while the remainder patients (15%) demonstrate microsatellite instability (MSI)^3^.

Although different critical genes and pathways have been related to pathogenesis of CRC, for many of these genomic alterations the prognostic and predictive roles are not known and do not influence treatment decision in the metastatic conditions^4^. The identification of K-RAS and more recently of NRAS gene mutations is a widely accepted molecular test in the clinical treatment decisions for mCRC^5^. The BRAF V600E mutation has been approved as a prognostic biomarker to identify patients who may develop an aggressive clinical outcome^6^. In mCRC patients has been demonstrated a relationship between MSI-high and an striking response to immune checkpoint blockade with anti-PD1 therapy^7^. High throughput sequencing platforms are the ideal candidates for an easy detection of gene mutational status in colorectal carcinoma.

The availability of new technologies as the cancer transcriptome analysis represents a usable tool to identify cancer-related mutations and/or alteration of gene regulation to explain the relationships between driver gene mutations and neoplastic cells behaviours^8^. Nannini *et al*. have tested the functional effectiveness of gene expression profiling, and proved that this approach allows molecular diagnosis and prognosis, disease sub-classification and treatment prediction by the identification of molecular targets^9^.

Our study is focused to obtain an integrated genetic/genomic understanding in colorectal carcinoma and distant NCT from patients diagnosed with CRC by using RNA-Seq. Several bioinformatics tools were used to identify pathways and networks, other than genetic alterations with the aim to understand the pathophysiology of CRC and select potential therapeutic targets.

## Results

### Transcriptome sequencing and mapping

Sixteen paired primary colorectal cancer and distant normal colon tissues were surgically removed from the Surgery Unit of the University of Sassari and enrolled in the study. The clinic-pathological features at diagnosis of CRC are reported in Table 1. Total RNA extracted from neoplastic and paired non-neoplastic tissues were subjected to high-throughput transcriptome sequencing.

**Table 1:** Clinic-pathological features of colorectal carcinoma.

Clinic-pathological features		Number (%)
Sex	Male	11 (68.8)
	Female	5 (31.2)
Age (years)	Median (range)	67.5 (50–89)
Site	Colon-right	9 (56.2)
	Colon-left	4 (25)
	Rectum	3 (18.8)
Grade	G1–G2	11 (68.8)
	G3	5 (31.2)
AJCC stage	I	3 (18.8)
	II	5 (31.2)
	III	7 (43.8)
	IV	1 (6.2)
KRAS*	Non-mutated	11 (68.8)
	Mutated	5 (31.2)

Grade: G1 Well differentiated, G2 Moderately differentiated, G3 Poorly differentiated; AJCC stage according to WHO criteria (Hamilton SR, 2010); KRAS, KRAS Proto-Oncogene, GTPase; *KRAS mutations were detected by RNA-Seq and Sanger Sequencing.

We sequenced an average of ~50 million 101-bp paired-end reads per sample. After quantification and quality controls, RNA-Seq data were aligned to the UCSC reference human genome GRCh37/hg19.

### Gene expression profiling

After transcripts quantification about 34.500 genes were detected, which included the majority of the annotated human reference genes. Comparing global gene expression between colorectal carcinoma and NCT, 1378 genes were showed differentially expressed, specifically 611 genes were overexpressed and 767 genes downregulated in CRC (see Supplementary Table S2). An unsupervised hierarchical clustering analysis of differentially expressed genes allowed to clearly separate normal colon tissues from tumour samples (see Supplementary Fig. S1).

**Figure 1:**
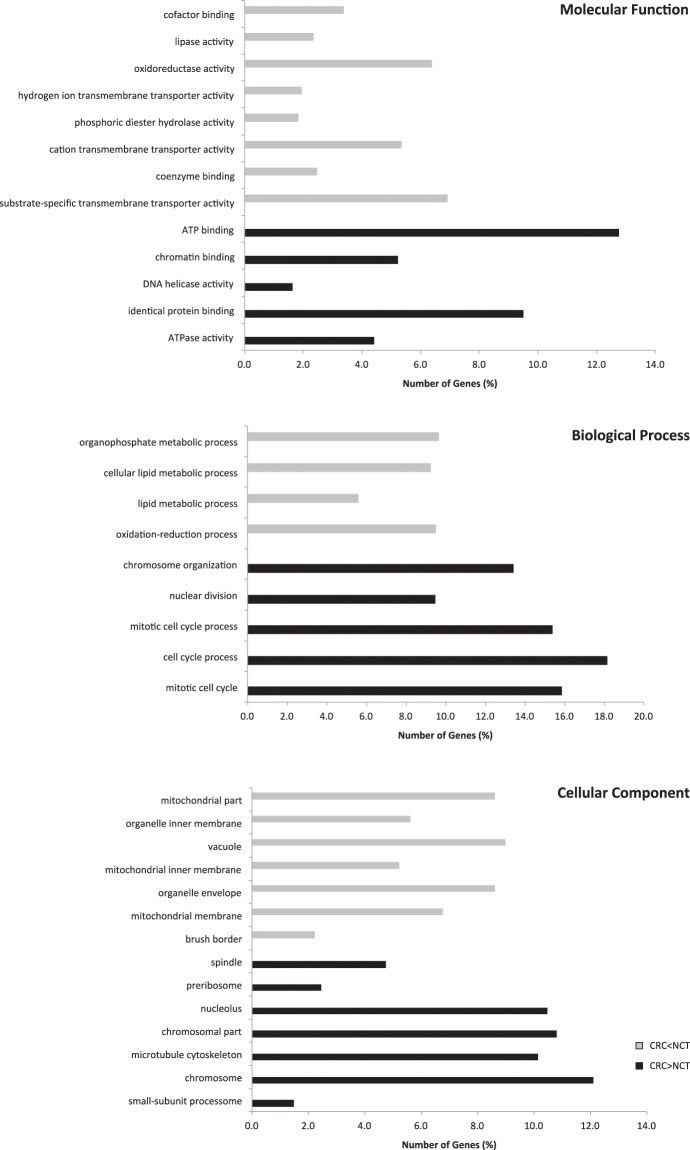
Gene Ontology (GO) enrichment of differentially expressed genes (DEGs) in colorectal carcinoma. Percentage of DEGs involved in each GO Molecular Functions term, GO Biological Processes term and GO Cellular Component term. The represented GO terms were significant at p-value < 0.1.

To investigate the role of the 1378 DEGs in tumour development, a functional enrichment analysis was performed. Interestingly, the DEGs overexpressed in CRC are involved in biological process such as chromosome organization, and mitotic cell cycle process. While the DEGs downregulated in CRC influenced organophosphate metabolic process, lipid metabolic process, and oxidation-reduction process. The DEGs were also enriched in molecular function and cellular component classes, as described in Supplementary Table S3, Fig. 1.

**Figure 2:**
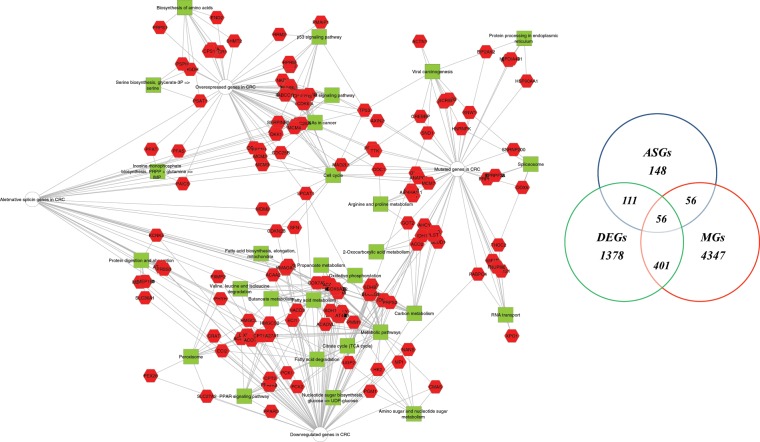
Colorectal tumour interactome and Venn diagram. On left, CRC interactome network developed using cytoscape of 1378 differentially deregulated genes, 148 alternative splicing genes, and 285 mutated genes. On right, Venn diagram of the overlap between differentially express genes (DEGs), mutated genes (MT) and alternative splicing genes (ASG).

The significant DEGs overexpressed in CRC were enriched in proteins that have key roles in cell cycle, p53 signalling pathway, Serine biosynthesis glycerate-3P => serine (KEGG amino acid metabolism pathway module), biosynthesis of amino acids, WNT signalling pathway, microRNA in cancer and inosine monophosphate biosynthesis, PRPP + glutamine => IMP (KEGG purine metabolism pathway module). The DEGs downregulated in CRC influenced pathways, such as fatty acid degradation and metabolism, metabolic pathway, citrate cycle, carbon metabolism, valine, leucine and isoleucine degradation, oxidative phosphorylation, peroxisome and PPAR signalling pathway, etc (see Supplementary Table S4, Fig. 2).

Interestingly, overexpression of LPCAT1 gene was shown in CRC, although all other members of the metabolic pathway were downregulated in tumours matched to NCT.

Moreover, we performed a functional enrichment analysis on differentially expressed genes identified in tumours with different clinic-pathological features. Tumours developed in the right colon are characterized by a downregulation of genes involved in ribosome pathway (RPL10, RPL10A, RPL12, RPL13, RPL14, RPL17, RPL21, RPL22, RPL2,6 RPL35A, RPL37A, RPL38, RPL39, RPL5, RPL6, RPS11, RPS15, RPS1,9 RPS21, RPS4X, RPS5, RPS9) matched to tumours developed in the left colon (Table S5A).

Furthermore, tumours without KRAS gene mutation show overexpression of ALDOB and CPS1 and downregulation of PGAM4 genes, which are involved in metabolic pathways such as biosynthesis of amino acids, gluconeogenesis, glycolysis and carbon metabolism (Table S5B). Finally, tumours with high grade (III-IV) are characterized by MAOB gene overexpression and CKMT2 gene downregulation, both related to arginine and proline metabolism pathway (Table S5C).

### Detection of gene mutations

As a first approach, cancer-specific somatic mutations were identified in each patient by comparing CRC with NCT variants and filtering out common variants. A variable range of single nucleotide variants (SNV)/INDEL were identified as tumour-specific somatic mutations in each patient.

We focused only on variants in coding region that may modify amino acids sequence and consequently change the protein function (Table 2). A total of 4347 mutated genes were identified.

**Table 2:** Cancer-specific somatic mutations identified by comparing colorectal variants with paired normal colon tissue variants.

Patient ID	n. of SNV/INDEL	n. of somatic variants	% of somatic variants	n. of somatic variants in coding region	Novel Variants	Known Variants	n. of genes
3	28614	1272	4,4	387	38	349	291
11	35635	2076	5,8	737	134	603	543
17	30561	3156	10,3	1247	173	1074	923
25	20721	761	3,7	188	28	160	113
35	26345	942	3,6	281	33	248	208
39	20255	778	3,8	208	23	185	153
43	38031	1631	4,3	531	57	474	391
49	27298	1192	4,4	423	35	388	286
51	42897	3399	7,9	1284	291	993	927
53	34784	1698	4,9	564	50	514	405
55	16282	646	4,0	210	27	183	154
59	26558	2254	8,5	873	165	708	644
61	21946	442	2,0	104	8	96	69
69	35369	1598	4,5	659	62	597	503
81	36339	4088	11,2	1615	241	1374	798
97	35441	2858	8,1	1151	271	880	861

We selected all genes that contained a protein-altering mutation in at least 4 of the 16 patient samples (25%). This process led to select 285 genes (see Supplementary Table S6) that were submitted to a functional enrichment analysis that demonstrated their connection to cell cycle, protein processing in the endoplasmic reticulum, spliceosome, citrate cycle, carbon metabolism, RNA transport, viral carcinogenesis pathways, etc. (see Supplementary Table S4, Fig. 2).

The list of 285 candidates comprises well known CRC driver genes such as TP53, KRAS, AXIN2 and ERBB2, although among the top twenty-five were identified 15 genes related to CRC pathogenesis such as CEBPZ, RRBP1, FXR1, LRPPRC, NAP1L1, AHCY, ALDH1A1, HNRNPU, ITGB1, KEAP1, NCL, OLA1, PLEC, REG4, YME1L1. In addition, we found in this list established cancer genes involved in other types of cancer, such as PDE4DIP, SF3B1, AHNAK, COPB2, CSDE1, KIF5B, NDUFA10, RSRC2.

### Detection of alternative splicing

The profile of mRNA splicing events was studied in all 32 transcriptomes. We detected 12800 significant alternative splicing (AS) events that occurred in CRC compared to NCT (Table 3). A total of 148 differentially splicing genes (DSGs) were identified in CRC, of which 17 genes were shown as novel splicing events (see Supplementary Table S7). Interestingly, 9 novel splicing genes out of 17 are shown to be involved in pathogenesis of CRC: PHLPP2, LAMA1, REG4, SLC9A1, ACAT1, CDC25B, UGP2, DPEP1 and PLCD3. Among the new splicing genes not previously related to CRC, noteworthy could be ARRDC4 and LRRN2. The ARRDC4 gene, member of arrestine family, which plays an important role in glucose metabolism and G-protein-coupled receptors, is involved in cell biology. Interestingly, gene expression profiles in EV71-infected macrophages have shown that the high level of ARRDC4 expression is positively correlated with the serum concentration of pro-inflammatory cytokines^10^. The LRRN2 gene, member of leucine-rich repeat superfamily, coded a protein that functions as cell-adhesion molecules or as signal transduction receptors^11^.

**Table 3:** Number of alternative splicing events occurring in colorectal carcinoma in comparison to normal tissues.

Event Type	NumEvents.JC + readsOnTarget	SigEvents.JC + readsOnTarget
SE	74824	4361 (1456:2905)
MXE	22385	6828 (5923:905)
A5SS	2792	581 (323:258)
A3SS	3409	755 (307:448)
RI	824	275 (155:120)

Type of alternative splicing event: SE: Skipped exon; MXE: Mutually exclusive exon; A5SS: Alternative 5′ splice site; A3SS: Alternative 3′ splice site; RI: Retained intron; NumEvents.JC + readsOnTarget: total number of events detected using both Junction Counts and reads on target; SigEvents.JC + readsOnTarget: number of significant events detected using both Junction Counts and reads on target; the numbers in the parentheses (n1:n2) indicate the number of significant events that have higher inclusion level for colorectal carcinoma (n1) or for normal tissue (n2).

A functional enrichment analysis was performed on all splicing genes showing their key roles in the cell cycle, in the metabolism and degradation of fatty acids and in the degradation pathways of valine, leucine and isoleucine (see Supplementary Table S4, Fig. 2).

**Figure 3:**
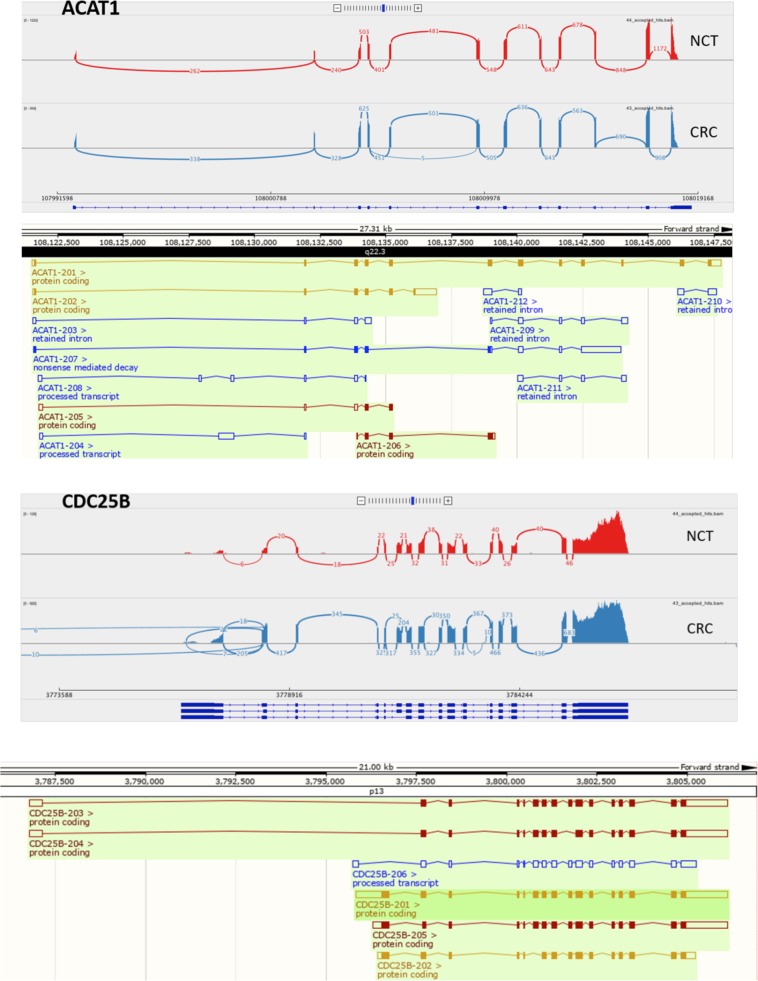
RNA-Seq reads coverage of the ACAT1 and CDC25B gene. The sashimi plot shows the junction supporting the exon skipping event in CRC respect to NBT. The CRC tissue tracks were drawn in light blue and the NBT in red. The ACAT1 and CDC25B known transcript were shown on A and B section, respectively.

We focused on ACAT1 and CDC25B genes involved in metabolism and cell cycle pathways as shown in Supplementary Table S4 and Fig. 2, which were affected by new alternative splicing events, downregulated and upregulated, respectively. The RNA-Seq data revealed a cassette exon exclusion of the exon 4 in the ACAT1 gene, and CDC25B gene showed an event of intron retention between exons 11 and 12 in CRC samples (Fig. 3). The functional meanings of these splicing variants should be established, although they are both in-frames, suggesting that the protein isoforms might be functional.

Finally, the degree of overlap between the DEGs, DSGs and mutated genes, showed that 56 genes were affected simultaneously by transcriptional and/or post-transcriptional deregulation (Fig. 2).

### Detection and validation of novel gene fusion transcripts

**Figure 4:**
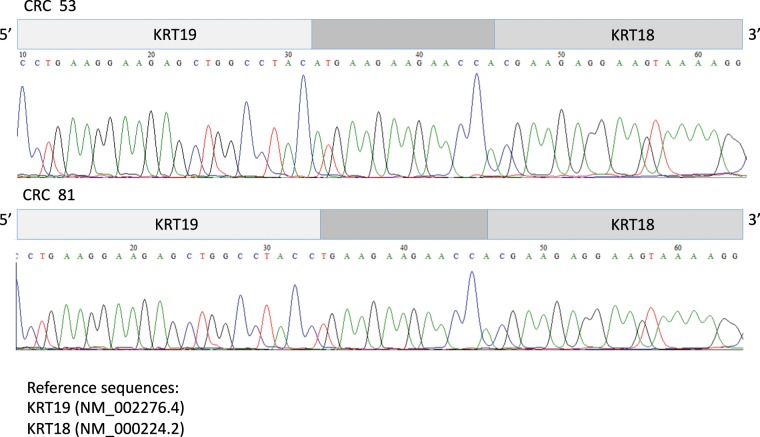
KRT19-KRT18 gene fusion in colorectal carcinoma. Sanger sequencing validation of fusion transcript structure according to presence of exon genes.

To identify gene fusion events, FusionCatcher algorithm was used^12^. We selected 138 gene fusion among the CRC patients based on following gene criteria: “*adjacent on the genome*”, “*coding for antisense*”, “known *oncogene*”, “*cancer associated*”, “*proto*-*oncogene or tumour suppressor gene*”, “*encoding for ribosomal protein*”, “*the fusion junction point is exactly at the known exon*’*s borders of both genes forming the candidate fusion*”. We focused on 2 gene-fusion events: EEF1A1-HSP90AB1 (chromosome 6) and KRT19-KRT18 (chromosome 17 and 12, respectively), both arising from frame fusions and shown in 2 out of 16 CRC patients (see Supplementary Table S8). The KRT19-KRT18 fusion transcripts (patients: number 53 and 81) were successfully amplified by RT-PCR and the fusion junctions were confirmed by the Sanger sequencing (see Supplementary Table S1, Fig. 4). Although the RNA Seq data were unambiguous, it was not possible to confirm by Sanger sequencing the EEF1A1-HSP90AB1 fusion transcripts (patients: number 11 and 43) probably due to an extremely limited number of transcripts. Supporting our findings, previous gene fusion events involving the EEF1A1 and HSP90AB1 genes have already been identified in HNSCC cell lines and haematological neoplasms, so they could be considered fragile sites and strengthen the credibility of our RNA Seq data^13^.

## Discussion

This study was justified by two fundamental assumptions: the RNA-Seq data generate an integrated multiple genomic model to configure the expression level, while the genomic structure of the CRC provides relevant biological predictions. We have identified about 1800 genes showing different aberrations and activity in CRC samples, as well as variations in mRNA expression, nucleotide sequence variations (mutations or alternative splicing) and gene fusion events. These transcriptional and post-transcriptional modifications influence functional cell process absolutely crucial to determinate and support the CRC phenotype.

Deregulation of several biological pathways is a hallmark in tumour biology. Our data highlight that a large number of differentially expressed genes and key pathways are involved in CRC biology. We proved that overexpressed genes identified in CRC are prevalently involved in pathways known to lead to tumour growth and progress, such as cell cycle, p53 signalling pathway, WNT signalling pathway and microRNA in cancer that regulate growth, differentiation, and cellular processes of apoptosis.

Conversely, genes down-regulated in CRC manage pathways that regulate cell metabolism, as well as fatty acid metabolism, citrate cycle, carbon and propanoate metabolism, amino acid metabolism; besides oxidative phosphorylation, peroxisome and PPAR signalling pathways. Brown *et al*. have recently identified various modifications in metabolites that involve CRC and adjacent mucosa metabolic pathway networks, showing the impact of metabolic networks for lipid, carbohydrate, amino acid, cofactors and vitamins pathways^14^. Hinnebusch *et al*. have shown that an altered short-chain fatty acid metabolism between CRC and the surrounding mucosa is remarkable due to the magnitude of the metabolites of short-chain fatty acids. Short-chain fatty acids such as butyrate, propionate, and valerate stopped the development in human CRC cells^15^.

Our data revealed that all genes engaged in metabolic processes are downregulated except for the LPCAT1 gene. This key enzyme in Lands cycle aides the synthesis of peculiar phosphatidylcholine species which constitute crucial molecules in eukaryotic membranes and main structural characteristics of serum lipoproteins^16^.

Mansilla *et al*. localized LPCAT1 protein in the endoplasmic reticulum and mitochondria where the protein plays a role in recycle LysoPtdCho and increase the palmitate content of PtdCho. In CRC, LysoPtdCho and PtdCho are upregulated and affect oncogenicity, especially increasing cell proliferation. The authors assume that LPCAT1 supplies the total increase of choline metabolite via improving PtdCho recycling and altering the specific lipid profile of CRCs^17^.

Downregulation of the genes involved in oxidative phosphorylation could affect energy production in CRC resulting in a shift from oxidative to substrate-level phosphorylation. Hinnebusch *et al*. observed the depletion of glucose-6-phosphate and fructose-6-phosphate levels in CRC compared to contiguous mucosa implying a Warburg effect^15^.

The downregulation of genes involved in PPAR signalling pathway identified in our study agrees with scientific work that has demonstrated the ability of PPARγ activation to induce neoplastic cell apoptosis and inhibition of cell growth *in vitro*^18^. PPARγ pathway can be assumed and studied as a novel druggable path for CRC.

Our results underlined that right-sided CRCs are characterized by a downregulation of genes involved in ribosome pathway compared to left-sided CRCs. Ribosomal proteins (RPs) are involved in the assembly of the basal translational machinery, and performed additional extra ribosomal functions in the cell including the regulation of cell growth and proliferation, differentiation, apoptosis, and DNA repair^19^. mRNA expression modification in the of RPs are frequent in cancer^20,21^. RPs expression changing might even prognostic or predictive. Low levels of RPL19 in patients with prostate cancer have associated with better survival^21^, conversely increased levels of RPS11 and RPS20 imply that survival of glioblastomas is limited^22^. The differences in RPs expression levels identified in our study suggests a different pathogenesis and clinical outcome in right- or left-sided CRC, and could represent a potential prognostic and therapeutic biomarkers for these tumours.

Our results underline a different gluconeogenesis and glycolysis activities between CRC patients with and without KRAS gene mutation. CRCs samples without KRAS gene mutations showed overexpression of ALDOB and CPS1 and downregulation of PGAM4 genes. Overexpression of ALDOB and other glycolysis-related genes with subsequent activation in the glycolytic pathway were identified in CRC studies “*in vivo*” and “*in vitro*”, arguing that high carbohydrates might activate the glycolytic pathway and be in close relation to CRC tumorigenesis^23^. Moreover, the upregulation of ALDOB promotes CRC metastasis by enabling epithelial–mesenchymal transition (EMT)^24^, PGAM4 gene is also involved in carbohydrate metabolism, presently it does not relate with development of cancer, but could represent a new candidate gene to investigate.

Advanced CRCs were characterized by overexpression of MAOB and downregulation of CKMT2 genes. The enzyme encoded by MAOB gene catalyses the oxidative deamination of biogenic and xenobiotic amines and is part of the flavin monoamine oxidase family. MAOB deregulation in CRC has not yet been studied, but overexpression of MAOB has been identified in metastatic breast cancer and caused shorter survival in brain metastasis^25^. Sharpe *et al*. have found a correlation between the highest MAOB overexpression and the highest grade of gliomas^26^. Overespression of MAOB increase levels of HiF-1α. HiF-1α protein is implicated in neoplastic process shifting from aerobic to anaerobic the cell metabolism. Meanwhile, upregulation of angiogenic proteins rises vascularization^27^. CKMT2 gene codifies a mitochondrial creatine kinase protein and CKMT2 gene is significantly expressed in left-sided CRC and predict its prognosis, affecting the survival and outcome^28^. Our experimental data might suggest that the downregulation of the mitochondrial protein kinase could be responsible of the shift of the ATP production to glycolysis, as a feature of rapidly growing tumours.

Xu *et al*.^29^ performed a transcriptome analysis on CRC biopsies, identifying DEGs GO enriched mainly in cell proliferation, lipid metabolism, as well in immune response and collagen catabolism. Comparison with our results, revealed a correlation with DEGs involved in the immune response and collagen catabolism (data not shown). We highlight we have found mutations in P4HA1 and P4HB genes, whose activity is essential for proper three-dimensional folding of procollagen chains. Overexpression of genes involved in extracellular matrix disassembly (MMPs, TIMP, FSCN1), could be responsible for cell neoplastic migration. Several genes involved in the immune system were identified as deregulated in both studies, such as interleukins, chemokines, APLN, TNFRSF11B, TNFS9, TCF7, AZGP1, strengthening the role of tumour extracellular environment alterations in the CRC biology.

The role of somatic mutations has been widely known in the cancer biology^30^. Candidate genes strategy is used to identify somatic mutations in neoplastic cells, but large-scale mutation profiling is not available for each tumour. RNA-Seq offers the complete characterization of transcript isoforms and identification of SNV and INDELs together with the digital count of mutated transcripts in neoplastic cells establishing the functional meaning of the mutated gene^31^.

We selected 285 mutated genes in at least 25% of CRC patients. In the first twenty-five positions we found genes known to matched to CRC or in general to cancer, and affecting 44–56% of the patients, confirming the complex and individual landscape of genetic mutations for CRC patients. An enrichment functional analysis proved their connection with cell cycle, spliceosome, RNA transport and protein processing, metabolism, viral carcinogenesis pathways, etc., controlling main cell processes known to promote tumorigenesis.

Based on gene expression and mutational status in our patients, we could argue that the canonical WNT pathway activation represents a central pathogenesis model in CRC. Our results identified only 1 out of 16 CRC patients affected by APC somatic mutation, and frequent somatic mutations in AXIN2 and CTNNB1, hallmarks of canonical WNT pathway activation. Mutation in APC and AXIN2 genes compromise the function of b-Catenin “destruction complex”, while activating mutations of CTNN1 decrease its proteosomic degradation^32^. Our work helps to argue that the activation of WNT pathway, could also be related to overexpression of LRP5/6, FZD, TCF4 and SOX9, which cooperate in the activation of the pathway as described in breast cancer^33^; and to downregulation of PPAR pathways, which ligand-bound PPARγ suppresses WNT pathway in neoplastic cells^34,35^. About 40% of KRAS and 19% of PIK3CA mutations have been shown in CRC. The canonical WNT pathway, could be stimulated directly by oncogenic or by crosstalk activation of KRAS/BRAF/MEK pathway. LRP6 phosphorylation by ERK1/2 may provide a point of convergence between KRAS/MAPK and WNT signalling during oncogenesis^36^. Finally, we identified strong overexpression of b-Catenin/TCF target genes, as MYC, CCND1, AXIN2, CD44, as additional support to our hypothesis.

Our data highlighted that MAD2L1 gene has shown different genetic aberrations in CRC whereby overexpression, missense mutations (in the 31% of tumours) and alternative splicing. Biological evidence, such as Arg558His and Leu84Met variants, indicate that MAD2L1 protein directly lead to chromosomal instability, that is recognized as a general property of carcinogenesis and contribute to the evolution of CRC origin and evolution^37,38^. MAD2L1 overexpression and mutation in CAL51 breast cell line, suggests that increased level of MAD2L1 transcripts potentially offset overexpression due to lack of normal function^39^. SGC7901 cells overexpressing Mad2beta variant became more resistant to adriamycin, vincristine and mitomycin by abrogating mitotic arrest and apoptosis, which might help gastric cancer cells to develop a multidrug resistance phenotype^40^. One of our best results is the recurrent deregulation of MAD2L1 gene in CRC, in concordance with the high frequency of CIN in these tumours: we think that could be used it as a new potential prognostic biomarker. The MAD2 gene represents the key effector downstream of the spindle group control point (SAC) and can be considered a feasible therapeutic target. Kaestner *et al*. have inhibited the SAC “*in vivo*” by using of specific siRNA-bound nanoparticles targeting MAD2 gene, as a consequence a severe chromosome mis-segregation and induction of aneuploidy has led to a decreased tumour growth by inducing apoptosis^41^. Therefore, the increased percentage of chromosome mis-segregation during mitosis could be considered as a basic model for targeted therapy in CRC patients.

RNA-Seq data analysis allowed us to identify alternative splicing and gene fusion events. The transcriptional plasticity generated by AS to remodel the proteome gives advantageous opportunities to cancer cells to disrupt the production of proteins needed to growth and spread the tumour^42^. Here we detected a total of 148 differentially splicing genes specific for CRC cells identifying new isoforms for seventeen genes. Most of them are known genes related to CRC pathogenesis, although the functions of these variants remained to be verified in future researches. Our study proves that CRC cells have multiple ways to reprogram its transcriptome: under our experimental conditions, multiple genes were simultaneously affected by different regulatory mechanisms. The interesting genes showing alternative splicing and increased expression level involved in the cell cycle pathway were CCND1, CDC25B, MCM2, MCM3 while ACAAA2, ACADS, ACAT1, ACOX, CPT1A, HMGCS2 show alternative splicing and downregulation involved in fatty acid metabolism.

Gene-fusion events may result in overexpression of a gene in one of the breakpoints or transcription of chimeric oncogenes. We identified 2 novel KRT19-KRT18 and EEF1A1-HSP90AB1 in-frame fusion events, encoding for chimeric proteins in two CRC patients. Mathias *et al*. proved that the downregulation of KRT18 and KRT19 is one of the distinctive molecular signs during EMT^43^. Abbas *et al*. have shown that EEF1A1 is involved in the tumour pathophysiology, and contributes to cell growth, cell cycle regulation, and maintenance of cell survival^44^. HSP90AB1 gene, member of the heat shock protein 90 family, mediates signal transduction, protein folding, degradation and morphological evolution. HSP90AB1 contributes to pathogenesis and progression of lung cancer, hepatocellular carcinoma, laryngeal carcinoma^45–47^. Additionally, we found a novel ALK-TRIM72 gene fusion in a CRC patient. ALK gene is frequently involved in fusion events in CRC^48–50^. We have not identified any downregulation of the gene-fusion described, assuming that the original function of these genes is not affected by the fusion events. Gene fusion events might be occurred due to genomic rearrangements, which are common events in tumours, conversely gene fusion events are not frequently identified in CRC^50^. Accordingly, discovering case-specific gene fusion could help to understand the complexity of the genomic and molecular aberrations involved in individual CRC biology. Considering the involvement of fusion transcripts expression in numerous cell processes, they could be considered as prognostic biomarkers and potential therapeutic targets.

Our study allowed to draw a functional interactome map involving integrated multiple genomic features identified in CRC. Our data underline that two functional cell programs are prevalently deregulated and absolutely crucial to determinate and sustain the CRC phenotype. The transcriptional and post-transcriptional aberrations identified in the neoplastic cells offer advantages for cell growth, proliferation and migration and disadvantages for apoptosis, conversely inhibit cell metabolism and determine the switch from oxidative to substrate-level phosphorylation in these cells. We have generated a valuable CRC transcriptome dataset for further investigations on mechanisms involved in tumour development, identification of gene signatures with prognostic and predictive value and most importantly of new therapeutic targets.

## Methods

The study was conducted according to the recommendations of the Helsinki Declaration and approved by the Azienda Sanitaria Locale Sassari Bioethics Committee (n. 2032/CE, 13/05/2014). All patients gave written informed consent for tissue banking and genetic analysis. Study population comprised anonymised patients consecutively diagnosed with CRC and treated by surgical resection in the Surgery Unit of Sassari University between 2013 and 2014. All cases achieved a final diagnosis of CRC according to World Health Organization criteria^51^.

### Samples and acid nucleic extraction

Whole-transcriptome and genomic analysis was performed on 16 paired matched fresh-frozen samples of primary CRC and NCT, the latter was taken about 10 cm from CRC.

Genomic DNA was obtained from neoplastic tissue, and total RNA was obtained from the same neoplastic and non-neoplastic specimens. Nucleic acids were extracted using the QIAamp DNA Mini Kit and miRNeasy Mini Kit (Qiagen, Hilden, Germany) following the manufacturer’s instructions.

The quantity and the quality of nucleic acids were assessed using Nanodrop ND1000 (EuroClone, Milan, Italy). The RNA quantity was evaluated by Qubit® RNA BR Assay Kit (ThermoFisher Scientific, Waltham, USA). The RNA integrity was assessed by the RNA Integrity Number (RIN) using the Agilent RNA 6000 Nano Kit on the BioAnalyzer 2100 (Agilent, Santa Clara, USA).

### Library preparation and sequencing procedures

Poly-A+ RNA was isolated from 1 µg of high-quality total RNA and libraries were prepared through two rounds of positive selection and purification using magnetic beads with poly-T oligos attached as suggested in the TruSeq RNA Sample Preparation manual (Illumina, San Diego, CA, USA). The indexed individual libraries were then pooled to obtain equimolar concentrations for each sample and then processed for cluster generation on a Paired-End Flow Cell using the cBot System (Illumina) and the TruSeq PE Cluster Generation kit v3 (Illumina). Sequencing runs were performed in 101 bp paired-end mode on an Illumina HiSeq 2000 using TruSeq SBS v3 reagents (Illumina).

### NGS data processing

Demultiplexed fastq files were obtained according to the Illumina Pipeline data analysis. Reads were quality-filtered using the standard Illumina process. Reads were then aligned to the UCSC Homo Sapiens reference genome (build GRCh37/hg19) using TopHat (http://tophat.cbcb.umd.edu/). TopHat were used to align reads to the genome and discovers transcript splice sites. The reads that did not align to the genome but that were mapped to these potential junctions were considered to bridge splice junctions. Reads per kilobase of transcript per million reads (RPKM) values were calculated using human RefSeq gene models and gene expression levels were estimated as FPKM values by the Cufflinks software. We performed Differentially Expressed Gene (DEG) analysis using the most frequently used programs: Cufflinks-Cuffdiff and DeSeq2^52^. Due to software constrains, we used Cuffdiff for an unmatched CRC vs NTC analysis while we performed the sample matched DEG analysis using DESeq2, and we retained the intersection of both to reduce the number of false positives. We used the cut-off of FDR corrected p-value < 0.05 for both Cuffdiff and DeSeq2 analysis. Detection of genes mutation was done using GATK Best Practices (https://software.broadinstitute.org/gatk/best-practices/). We used MuTect2: a somatic SNP and indel caller that combines the DREAM challenge-winning somatic genotyping engine of the original MuTect^53^ via local re-assembly of haplotypes. MuTect2 allows for a varying allelic fraction for each variant, as is often seen in tumours with purity less than 100%, multiple subclones, and/or copy number variation. VCF were annotated using Variant Effect Predictor (VEP) that determines the effect of SNPs, INDELs, etc. on genes, transcripts, and protein sequence. We applied SIFT and PolyPhen scores for predicting amino acid changes that affect protein function. Filtering was performed according to: genotype quality, pattern of inheritance, gene feature and excluding common variants in reference databases (dbSNP138, dbSNP141, 1000 Genomes and ExAC). Variants were evaluated for their phenotypic and biological function and possible causative variants. We have used rMATS 4.0.2 (http://rnaseq-mats.sourceforge.net/index.html)^54^ to identify differential alternative splicing events from RNA-Seq data. rMATS has much greater flexibility in the detection of differential alternative splicing, providing a statistical model. It identifies alternative splicing events corresponding to all major types of alternative splicing patterns and calculates the P value and false discovery rate for differential splicing.

The FusionCatcher program v0.99.4e (https://github.com/ndaniel/fusioncatcher)^55^ with the associated ENSEMBL, UCSC, and RefSeq databases allowed the discovery of fusion transcripts. FusionCatcher integrates four commonly used aligners (Bowtie, BLAT, STAR, and Bowtie2) to find reads of fusion transcripts. This algorithm compares its own output with a set of published databases, thus proving a detailed list of truly positive and false positive predicted gene fusion events. The candidate fusion transcripts were validated by Sanger sequencing, according to the breakpoints obtained by bioinformatic analysis in the matched tumour samples. KRAS mutation analyses were performed on exons 2 and 3 which are known to harbour the most frequent and significant mutations. PCR primers are listed in Supplementary Table S1.

### Functional classification and pathway analysis of deregulated genes

Gene Ontology (GO) enrichment analysis and KEGG pathway analysis were executed to survey biological functions of all genes showing genomic abnormalities by the online software ToppCluster (https://toppcluster.cchmc.org/)^56^. Terms with False Discovery Rate (FDR) corrected enrichment p-values < 0.05 were considered. While the interaction between altered genes and their matching terms were visualized by Cytoscape program (http://www.cytoscape.org)^57^.

### Ethics approval and informed consent

The study protocol was performed in accordance with the Declaration of Helsinki and approved by the Azienda Sanitaria Locale Sassari Bioethics Committee (n. 1140/L 05/21/2013). All patients gave written informed consent for tissue banking and genetic analysis.

## Supplementary information

Supplementary Information.

## Data Availability

The data that support the findings of this study are available from the corresponding author upon reasonable request.
