# Supplementary material for: Landscape of transcriptome variations uncovering known and novel driver events in colorectal carcinoma
Source: Sci Rep. 2020 Jan 16;10:432. doi: 10.1038/s41598-019-57311-z (PMC6965099; doi:10.1038/s41598-019-57311-z)
Supplement: Supplementary file 1 — Supplementary Information. [file 41598_2019_57311_MOESM1_ESM.pdf]

Supplementary Information

Landscape of transcriptome variations uncovering known and novel driver events in colorectal carcinoma

Giovanna Pira[1†], Paolo Uva[2†], Antonio Scanu[3], Paolo Cossu Rocca[3,4], Luciano Murgia[3], Elena Uleri[1], Claudia Piu[1], Alberto Porcu[3], Ciriaco Carru[1], Alessandra Manca[5], Ivana Persico[6], Maria Rosaria Muroni[3], Francesca Sanges[1], Caterina Serra[1], Antonia Dolei[1], Andrea Angius[1,6]*, Maria Rosaria De Miglio[3]

[1]Department of Biomedical Sciences, University of Sassari, Viale San Pietro 43-b, 07100 Sassari, Italy;

[2]CRS4, Science and Technology Park Polaris, Piscina Manna, 09010 Pula, CA, Italy;

[3]Department of Medical, Surgical and Experimental Sciences, University of Sassari, Viale San Pietro 8, 07100 Sassari, Italy;

[4]Department of Diagnostic Services, "Giovanni Paolo II" Hospital, ASSL Olbia-ATS Sardegna, Via Bazzoni-Sircana, 07026 Olbia, Italy;

[5]Department of Pathology, AOU Sassari, Via Matteotti 60, 07100 Sassari, Italy;

[6]Istituto di Ricerca Genetica e Biomedica (IRGB), CNR, Cittadella Universitaria di Cagliari, 09042 Monserrato (CA), Italy;

*__Corresponding author__

Andrea Angius, PhD

Istituto di Ricerca Genetica e Biomedica

Consiglio Nazionale delle Ricerche

Cittadella Universitaria di Cagliari - 09042 Monserrato (Cagliari) Italy

E-mail: andrea.angius@irgb.cnr.it

Table S1. Designed primers for specific gene amplification and Sanger sequencing validation

Gene target	Primers Sequence	Tm	Product size (bp)
KRAS exon 2	5'-GTTTGTATTAAAAGGTACTGGTGGA-3'	58 C°	270
	5'-ATCAAAGAATGGTCCTGCAC-3'		
KRAS exon 3	5'-TCAAGTCCTTTGCCCATTTT-3'	55 C°	300
	5'-AACCCACCTATAATGGTGAATATC-3'		
KRT19-KRT18	5'-GGTGCCACCATTGAGAACTCC-3'	54,4 C°	385
	5'-ACTTGTCTAGCTCCTCTCGGT-3'		
KRT19-KRT18 Nested	5'-GATCGAAGGCCTGAAGGAAGAG-3'	58,4 C°	111
	5'-GCATCTACCTCCACGGTCAA-3'		
EEF1A1-HSP90AB1 (sample 11)	5'-GGGTGGAGACTGAAGTTAGGC-3'	57 C°	385
	5'-AAGCCAGAAGATAGCAGGGC-3'		
EEF1A1-HSP90AB1 Nested (sample 11)	5'-TCAGGTGTCGTGAAAACTACCC-3'	58,4 C°	222
	5'-ACCACCAGGTCCTTAACTGC-3'		
EEF1A1-HSP90AB1 (sample 43)	5'-GGTGTCGTGAAAACTACCCCT-3'	55 C°	392
	5'-TGTCATCAGGGTTTCTGGTCC-3'		
EEF1A1-HSP90AB1 Nested (sample 43)	5'-GGACACGTAGATTCGGGCAA-3'	58,4 C°	225
	5'-CTTACCGCTGTCATCCTCCTC-3'		

Supplementary Fig. S1.

Heat map and hierarchical clustering dendrogram of differentially expressed mRNA transcripts. The column clustering showed a distinguishable expression profiling of 1378 differentially expressed mRNA transcripts between CRC and NCT. The scale of expression level was shown in the color key.

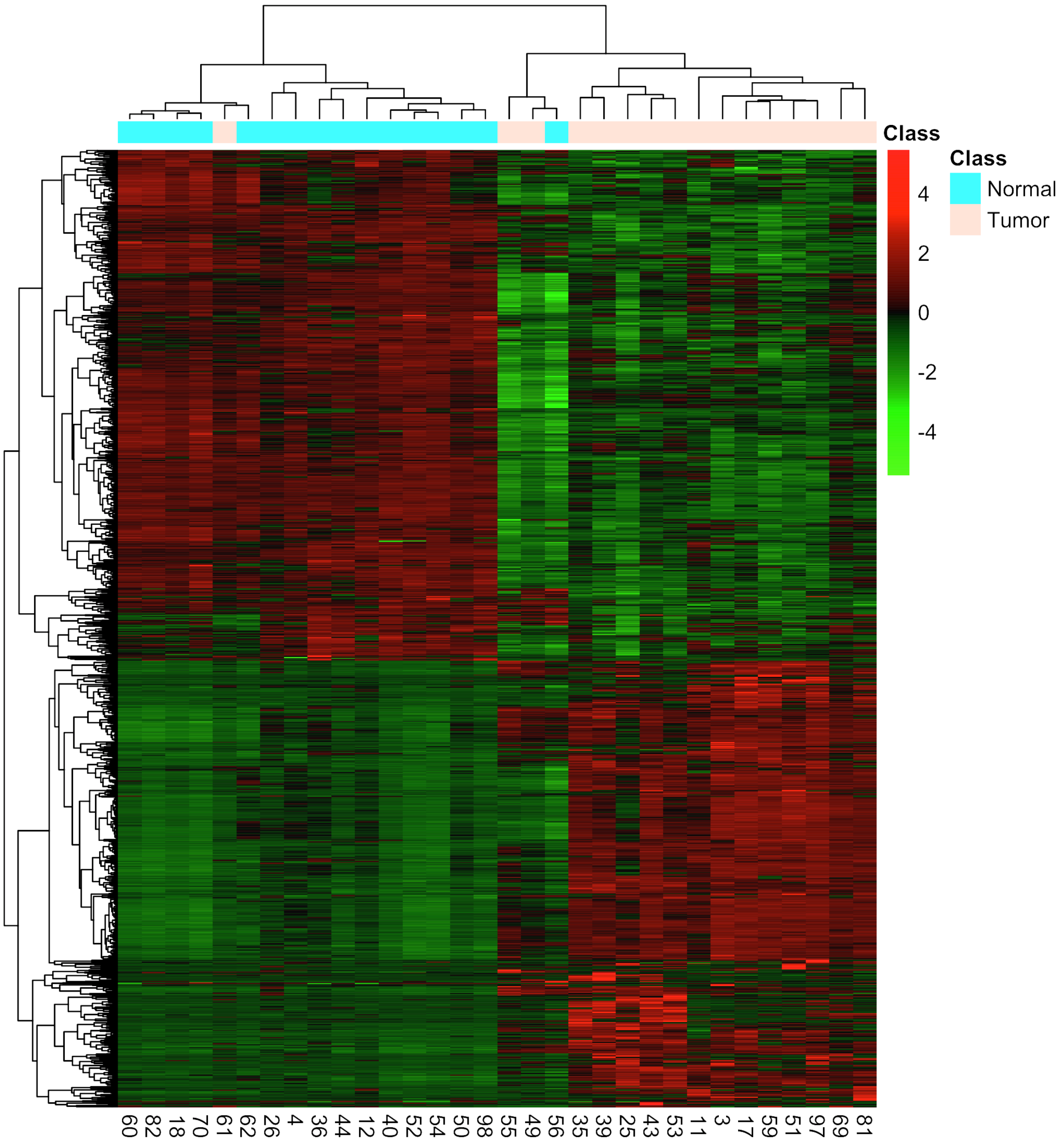

Table S2

Gene up- and downregulated in colorectal carcinoma.

test_id	gene_name	locus	Average expression CRC	NCT	log2 (CRC/NCT)	Class	p_value	q_value
XLOC_016809	LOC101927884	chr2:75719443-75822893	0,641	0,001	9,45	CRC > NCT	0,0028	0,0405866
XLOC_013943	NOTUM	chr17:79910379-79920497	40,940	0,088	8,86	CRC > NCT	5,00E-05	0,0020443
XLOC_007799	LOC100190940	chr12:130499823-130529900	3,131	0,009	8,46	CRC > NCT	5,00E-05	0,0020443
XLOC_005431	MUC6	chr11:1012622-1036706	1,121	0,004	8,14	CRC > NCT	0,0002	0,006387
XLOC_007709	LINC01234	chr12:114181235-114211506	4,033	0,016	8,00	CRC > NCT	0,0031	0,0433833
XLOC_018038	RPRM	chr2:154197365-154449793	0,876	0,005	7,50	CRC > NCT	0,00145	0,0259223
XLOC_006553	SLCO1B3	chr12:20963637-21070041	5,772	0,032	7,49	CRC > NCT	5,00E-05	0,0020443
XLOC_024731	LINC01411	chr5:173763356-173959512	2,764	0,016	7,42	CRC > NCT	5,00E-05	0,0020443
XLOC_029201	FEZF1-AS1	chr7:121941362-121954759	1,227	0,010	6,96	CRC > NCT	5,00E-05	0,0020443
XLOC_007089	PIWIL1	chr12:130822374-130856914	3,446	0,030	6,86	CRC > NCT	5,00E-05	0,0020443
XLOC_033390	CLDN2	chrX:106143292-106174300	51,599	0,463	6,80	CRC > NCT	5,00E-05	0,0020443
XLOC_008329	LINC00460	chr13:107028908-107036759	4,586	0,043	6,72	CRC > NCT	5,00E-05	0,0020443
XLOC_002525	LEMD1	chr1:205342379-205425214	5,732	0,061	6,55	CRC > NCT	5,00E-05	0,0020443
XLOC_029858	FEZF1	chr7:121941362-121954759	0,645	0,007	6,55	CRC > NCT	5,00E-05	0,0020443
XLOC_006046	MMP7	chr11:102391238-102401478	55,569	0,609	6,51	CRC > NCT	5,00E-05	0,0020443
XLOC_018093	MYO3B	chr2:171034654-171539570	0,390	0,005	6,30	CRC > NCT	5,00E-05	0,0020443
XLOC_021006	-	chr3:34226237-34477572	1,926	0,025	6,27	CRC > NCT	5,00E-05	0,0020443
XLOC_014283	DSG3	chr18:29027712-29058666	5,227	0,075	6,13	CRC > NCT	0,0001	0,003647
XLOC_012893	IGF2BP1	chr17:47074577-47133507	0,736	0,012	5,95	CRC > NCT	0,00175	0,0294656
XLOC_031695	CA9	chr9:35673893-35681182	45,781	0,744	5,94	CRC > NCT	5,00E-05	0,0020443
XLOC_023262	ANXA10	chr4:169013687-169115651	15,572	0,260	5,90	CRC > NCT	0,00035	0,0094833
XLOC_011497	TRIM72	chr16:31225202-31238552	1,118	0,019	5,90	CRC > NCT	0,0006	0,0139198
XLOC_019811	SIM2	chr21:38067491-38362677	2,207	0,037	5,89	CRC > NCT	0,00305	0,0429805
XLOC_005453	-	chr11:2227423-2233481	5,526	0,096	5,85	CRC > NCT	5,00E-05	0,0020443
XLOC_007554	-	chr12:75966943-76377950	0,790	0,014	5,81	CRC > NCT	5,00E-05	0,0020443
XLOC_011531	NKD1	chr16:50581545-50683157	10,217	0,194	5,72	CRC > NCT	5,00E-05	0,0020443
XLOC_010576	LOC101927286	chr15:97779480-98065041	8,426	0,172	5,61	CRC > NCT	5,00E-05	0,0020443
XLOC_021467	TM4SF4	chr3:149192367-149221181	17,539	0,362	5,60	CRC > NCT	5,00E-05	0,0020443
XLOC_025716	FOXQ1	chr6:1311758-1316660	16,776	0,348	5,59	CRC > NCT	5,00E-05	0,0020443
XLOC_008992	DHRS2	chr14:24099328-24114848	8,154	0,175	5,55	CRC > NCT	5,00E-05	0,0020443
XLOC_017723	-	chr2:67749743-67791797	0,945	0,020	5,53	CRC > NCT	0,00085	0,0178569
XLOC_025771	RNF182	chr6:13924676-13980496	0,536	0,013	5,37	CRC > NCT	0,00035	0,0094833

XLOC_017144	GAD1	chr2:171673199-171717659	2,159	0,057	5,26	CRC > NCT	5,00E-05	0,0020443
XLOC_007405	KRT5,KRT6A,KRT6B,KRT6C,KRT75	chr12:52817311-52914385	12,028	0,323	5,22	CRC > NCT	5,00E-05	0,0020443
XLOC_025155	ACSL6	chr5:131285666-131347761	4,074	0,110	5,21	CRC > NCT	5,00E-05	0,0020443
XLOC_013519	KRT23	chr17:39077238-39132428	26,390	0,716	5,20	CRC > NCT	5,00E-05	0,0020443
XLOC_022366	CLDN1	chr3:190023349-190041162	20,948	0,569	5,20	CRC > NCT	5,00E-05	0,0020443
XLOC_026417	UNC93A	chr6:167684583-167729774	1,847	0,050	5,19	CRC > NCT	0,0002	0,006387
XLOC_000996	DUSP27	chr1:167064086-167098402	8,384	0,233	5,17	CRC > NCT	0,00015	0,0050841
XLOC_026762	PGC	chr6:41704448-41721880	3,157	0,094	5,07	CRC > NCT	0,00325	0,0448628
XLOC_013599	ETV4	chr17:41605208-41623800	40,990	1,231	5,06	CRC > NCT	5,00E-05	0,0020443
XLOC_031271	CASC19,CCAT1	chr8:128187718-128231595	4,902	0,155	4,98	CRC > NCT	5,00E-05	0,0020443
XLOC_000598	EPHX4	chr1:92495532-92529212	4,406	0,141	4,97	CRC > NCT	5,00E-05	0,0020443
XLOC_006754	NXPH4	chr12:57610577-57620232	3,285	0,107	4,95	CRC > NCT	0,00075	0,0164378
XLOC_029566	INHBA	chr7:41724709-41818976	3,331	0,115	4,86	CRC > NCT	5,00E-05	0,0020443
XLOC_024232	LINC01021	chr5:27472387-27496508	1,125	0,042	4,74	CRC > NCT	5,00E-05	0,0020443
XLOC_009041	PAX9	chr14:37126772-37643075	0,727	0,027	4,73	CRC > NCT	0,0016	0,0279196
XLOC_007339	-	chr12:47779452-47821127	0,461	0,018	4,72	CRC > NCT	0,00075	0,0164378
XLOC_015461	PRKCG	chr19:54385466-54410906	1,075	0,042	4,69	CRC > NCT	0,0001	0,003647
XLOC_029307	PRSS1	chr7:142457211-142482401	2,445	0,097	4,66	CRC > NCT	0,00045	0,0114127
XLOC_026055	C6orf223	chr6:43963460-44042389	6,608	0,265	4,64	CRC > NCT	5,00E-05	0,0020443
XLOC_001596	HTR1D	chr1:23514265-23544010	2,942	0,120	4,62	CRC > NCT	5,00E-05	0,0020443
XLOC_011634	CDH3	chr16:68678150-68732957	13,142	0,549	4,58	CRC > NCT	5,00E-05	0,0020443
XLOC_019235	MYT1	chr20:62795826-62873606	0,721	0,031	4,55	CRC > NCT	0,0016	0,0279196
XLOC_029415	ELFN1-AS1	chr7:1705748-1787590	10,473	0,456	4,52	CRC > NCT	0,0002	0,006387
XLOC_013561	KRT17	chr17:39775691-39780882	43,031	1,876	4,52	CRC > NCT	5,00E-05	0,0020443
XLOC_007870	-	chr12:13693017-13700768	0,312	0,014	4,52	CRC > NCT	0,001	0,0199726
XLOC_011771	DPEP1	chr16:89679715-89708773	102,155	4,485	4,51	CRC > NCT	5,00E-05	0,0020443
XLOC_017788	REG3A	chr2:79384131-79386880	536,227	23,545	4,51	CRC > NCT	0,0004	0,01042
XLOC_004828	GYLTL1B	chr11:45943171-45950694	8,566	0,382	4,49	CRC > NCT	5,00E-05	0,0020443
XLOC_028731	CYP2W1	chr7:1022828-1029428	14,453	0,668	4,44	CRC > NCT	5,00E-05	0,0020443
XLOC_013108	CBX2	chr17:77751966-77762168	2,759	0,130	4,40	CRC > NCT	5,00E-05	0,0020443
XLOC_008113	PDX1	chr13:28393269-28500596	4,542	0,216	4,39	CRC > NCT	5,00E-05	0,0020443
XLOC_013000	CRAT40	chr17:63453459-63462600	0,610	0,029	4,37	CRC > NCT	0,0001	0,003647
XLOC_026489	TFAP2A	chr6:10396915-10419797	2,200	0,107	4,36	CRC > NCT	5,00E-05	0,0020443
XLOC_024733	MSX2	chr5:174151574-174157902	5,650	0,276	4,35	CRC > NCT	0,0003	0,0084946
XLOC_030839	DEFA6	chr8:6782215-6783598	80,854	4,007	4,33	CRC > NCT	5,00E-05	0,0020443
XLOC_017142	SP5	chr2:171556861-171655481	7,923	0,395	4,33	CRC > NCT	0,0005	0,0123209
XLOC_019589	LINC00659	chr20:61404039-61408310	1,359	0,068	4,31	CRC > NCT	5,00E-05	0,0020443
XLOC_025263	SH3TC2	chr5:148361712-148442737	0,888	0,045	4,30	CRC > NCT	5,00E-05	0,0020443

XLOC_030739	POU5F1B	chr8:128256407-128507161	9,277	0,475	4,29	CRC > NCT	5,00E-05	0,0020443
XLOC_007721	TESC	chr12:117476527-117579294	67,536	3,472	4,28	CRC > NCT	5,00E-05	0,0020443
XLOC_005761	TCN1	chr11:59620271-59634041	46,047	2,404	4,26	CRC > NCT	0,00085	0,0178569
XLOC_016313	IL11	chr19:55875749-55881831	1,890	0,102	4,21	CRC > NCT	5,00E-05	0,0020443
XLOC_006117	TMPRSS13	chr11:117771171-117800168	4,286	0,233	4,20	CRC > NCT	5,00E-05	0,0020443
XLOC_006819	LGR5	chr12:71831460-71980320	12,182	0,670	4,19	CRC > NCT	5,00E-05	0,0020443
XLOC_016300	TNNT1	chr19:55644065-55660722	6,595	0,363	4,18	CRC > NCT	5,00E-05	0,0020443
XLOC_017295	CPS1	chr2:211342405-211543857	13,797	0,764	4,17	CRC > NCT	0,00065	0,0147056
XLOC_020602	TMEM211	chr22:25331035-25342703	6,985	0,391	4,16	CRC > NCT	5,00E-05	0,0020443
XLOC_010527	ACAN	chr15:89346673-89418585	0,327	0,018	4,15	CRC > NCT	5,00E-05	0,0020443
XLOC_031797	PSAT1	chr9:80911990-80945009	33,833	1,934	4,13	CRC > NCT	5,00E-05	0,0020443
XLOC_001132	PKP1	chr1:201252579-201302121	2,332	0,134	4,13	CRC > NCT	0,00155	0,0273878
XLOC_023367	PPP2R2C	chr4:6322304-6565327	1,473	0,084	4,13	CRC > NCT	5,00E-05	0,0020443
XLOC_003626	ABCC2	chr10:101542354-101613399	1,964	0,113	4,12	CRC > NCT	5,00E-05	0,0020443
XLOC_014369	SERPINB5	chr18:61144143-61172320	59,412	3,517	4,08	CRC > NCT	5,00E-05	0,0020443
XLOC_001329	RYR2	chr1:237205701-237997371	0,659	0,039	4,07	CRC > NCT	0,00035	0,0094833
XLOC_034013	HS6ST2	chrX:131760037-132095423	2,513	0,150	4,07	CRC > NCT	5,00E-05	0,0020443
XLOC_024706	GABRP	chr5:170196700-170241321	2,799	0,167	4,07	CRC > NCT	5,00E-05	0,0020443
XLOC_025356	STC2	chr5:172741721-172756506	5,941	0,355	4,07	CRC > NCT	0,0004	0,01042
XLOC_012105	CRNDE	chr16:54951584-54963101	5,044	0,305	4,05	CRC > NCT	5,00E-05	0,0020443
XLOC_001872	LOC101926964	chr1:61121215-61292252	0,540	0,033	4,04	CRC > NCT	0,00045	0,0114127
XLOC_027126	PDE10A	chr6:165740775-166078124	2,151	0,130	4,04	CRC > NCT	5,00E-05	0,0020443
XLOC_033903	NXF3	chrX:102330722-102348087	2,913	0,179	4,02	CRC > NCT	0,00025	0,0074979
XLOC_017934	IL1A	chr2:113531491-113542971	3,984	0,249	4,00	CRC > NCT	0,0001	0,003647
XLOC_016013	CNTD2	chr19:40728114-40732616	3,632	0,227	4,00	CRC > NCT	5,00E-05	0,0020443
XLOC_016591	C2orf70	chr2:26785352-26864211	4,441	0,279	3,99	CRC > NCT	0,00015	0,0050841
XLOC_019540	SALL4	chr20:50398886-50419048	0,429	0,027	3,99	CRC > NCT	5,00E-05	0,0020443
XLOC_012531	DNAH2	chr17:7620671-7737062	0,422	0,027	3,98	CRC > NCT	5,00E-05	0,0020443
XLOC_007248	GRIN2B	chr12:13709027-14134950	0,727	0,046	3,97	CRC > NCT	0,0001	0,003647
XLOC_010348	C2CD4A	chr15:62358652-62364359	6,997	0,448	3,97	CRC > NCT	0,0001	0,003647
XLOC_019588	SLCO4A1-AS1	chr20:61268734-61304370	6,558	0,438	3,91	CRC > NCT	0,00335	0,0457925
XLOC_000283	OPRD1	chr1:29138653-29197772	0,325	0,022	3,88	CRC > NCT	0,00265	0,0390961
XLOC_033407	TDGF1P3	chrX:109763539-109766249	0,718	0,049	3,87	CRC > NCT	0,00095	0,0192909
XLOC_015473	LAIR2	chr19:55014012-55021902	2,604	0,179	3,86	CRC > NCT	0,00175	0,0294656
XLOC_026449	-	chr6:1283949-1289156	0,533	0,038	3,82	CRC > NCT	0,0005	0,0123209
XLOC_003790	VENTX	chr10:135051407-135055434	3,735	0,266	3,81	CRC > NCT	0,00295	0,0419277
XLOC_007366	DDN	chr12:49388932-49393088	0,460	0,033	3,78	CRC > NCT	0,00055	0,0131101
XLOC_030971	DUSP4	chr8:29190578-29208267	17,302	1,258	3,78	CRC > NCT	5,00E-05	0,0020443

XLOC_034008	FIRRE	chrX:130823786-130964861	0,678	0,051	3,74	CRC > NCT	0,0002	0,006387
XLOC_014314	SLC14A1	chr18:43303900-43332485	7,830	0,590	3,73	CRC > NCT	5,00E-05	0,0020443
XLOC_017884	CREG2	chr2:101962347-102003965	0,675	0,051	3,73	CRC > NCT	0,0001	0,003647
XLOC_023072	SPP1	chr4:88896801-88904563	64,141	4,945	3,70	CRC > NCT	5,00E-05	0,0020443
XLOC_005883	SPTBN2	chr11:66449971-66496260	3,358	0,264	3,67	CRC > NCT	5,00E-05	0,0020443
XLOC_004613	BRSK2	chr11:1411128-1483951	0,731	0,059	3,64	CRC > NCT	5,00E-05	0,0020443
XLOC_010780	WDR72	chr15:53805937-54055075	4,619	0,374	3,63	CRC > NCT	0,00015	0,0050841
XLOC_026807	PTCHD4	chr6:47845172-48079148	0,955	0,077	3,62	CRC > NCT	0,0008	0,0171221
XLOC_003414	DKK1	chr10:54074040-54077417	2,951	0,242	3,61	CRC > NCT	5,00E-05	0,0020443
XLOC_017908	EDAR	chr2:109510903-109605828	3,127	0,257	3,60	CRC > NCT	5,00E-05	0,0020443
XLOC_006052	MMP1	chr11:102654406-102714342	49,959	4,160	3,59	CRC > NCT	5,00E-05	0,0020443
XLOC_009499	AJUBA	chr14:23440327-23451851	4,123	0,347	3,57	CRC > NCT	5,00E-05	0,0020443
XLOC_011770	CPNE7	chr16:89642175-89663656	4,226	0,360	3,55	CRC > NCT	5,00E-05	0,0020443
XLOC_019273	SLC4A11	chr20:3207370-3220240	2,764	0,236	3,55	CRC > NCT	5,00E-05	0,0020443
XLOC_006671	KRT7	chr12:52623786-52761309	5,582	0,482	3,53	CRC > NCT	0,00125	0,0233782
XLOC_013521	KRT40	chr17:39133967-39143400	2,125	0,186	3,51	CRC > NCT	0,00065	0,0147056
XLOC_017426	FAM132B	chr2:239066272-239077532	0,720	0,063	3,51	CRC > NCT	0,0012	0,0226522
XLOC_019824	-	chr21:40358073-40396564	0,745	0,067	3,47	CRC > NCT	0,00245	0,0372298
XLOC_000488	PCSK9	chr1:55504991-55683128	10,917	0,991	3,46	CRC > NCT	0,0017	0,0290257
XLOC_025013	FOXD1	chr5:72742084-72744352	1,404	0,128	3,45	CRC > NCT	0,0028	0,0405866
XLOC_004105	MAT1A	chr10:82031573-82049434	1,019	0,093	3,45	CRC > NCT	0,0003	0,0084946
XLOC_029235	SMKR1,STRIP2	chr7:129074244-129152773	3,223	0,295	3,45	CRC > NCT	0,00015	0,0050841
XLOC_033995	APLN	chrX:128779235-128788933	2,951	0,271	3,44	CRC > NCT	0,0009	0,0185023
XLOC_004725	RASSF10	chr11:13030872-13036667	1,273	0,117	3,44	CRC > NCT	0,00075	0,0164378
XLOC_023477	ERVMER34-1	chr4:53607394-53617807	0,757	0,070	3,44	CRC > NCT	5,00E-05	0,0020443
XLOC_033710	NDP	chrX:43808022-43832921	0,831	0,077	3,43	CRC > NCT	0,0013	0,0239449
XLOC_034342	-	chrX:154044931-154045568	3,954	0,373	3,41	CRC > NCT	0,0009	0,0185023
XLOC_009622	-	chr14:54064707-54081120	1,537	0,148	3,38	CRC > NCT	0,0003	0,0084946
XLOC_031244	SNTB1	chr8:121547776-121826228	7,197	0,692	3,38	CRC > NCT	5,00E-05	0,0020443
XLOC_017227	SLC39A10	chr2:196477774-196933571	6,023	0,582	3,37	CRC > NCT	0,00065	0,0147056
XLOC_003736	BTBD16	chr10:124030820-124097838	1,113	0,108	3,36	CRC > NCT	0,0002	0,006387
XLOC_019555	BMP7	chr20:55743758-55841716	8,442	0,820	3,36	CRC > NCT	5,00E-05	0,0020443
XLOC_003788	KNDC1	chr10:134973862-135039916	0,497	0,049	3,33	CRC > NCT	0,00175	0,0294656
XLOC_012272	SLC7A5	chr16:87863623-87903100	27,237	2,704	3,33	CRC > NCT	5,00E-05	0,0020443
XLOC_024949	MCIDAS	chr5:54513301-54523165	0,380	0,038	3,32	CRC > NCT	0,0026	0,0386713
XLOC_029921	KIAA1549	chr7:138516119-138666064	1,510	0,152	3,31	CRC > NCT	5,00E-05	0,0020443
XLOC_002207	S100A2	chr1:153533584-153541068	9,548	0,972	3,30	CRC > NCT	5,00E-05	0,0020443
XLOC_015086	HPN	chr19:35531409-35604714	5,405	0,553	3,29	CRC > NCT	0,00055	0,0131101

XLOC_024 179	NKD2	chr5:1008927-1040830	5,616	0,583	3,27	CRC > NCT	5,00E-05	0,0020443
XLOC_030 850	DEFA5	chr8:6912821-6914261	143,332	14,993	3,26	CRC > NCT	5,00E-05	0,0020443
XLOC_015 007	GDF15	chr19:18493011-18500739	220,427	23,087	3,26	CRC > NCT	5,00E-05	0,0020443
XLOC_013 507	TNS4	chr17:38621719-38657909	24,283	2,545	3,25	CRC > NCT	5,00E-05	0,0020443
XLOC_005 455	ASCL2	chr11:2289519-2295262	43,198	4,528	3,25	CRC > NCT	5,00E-05	0,0020443
XLOC_023 716	SLC7A11	chr4:138948576-139163824	4,197	0,445	3,24	CRC > NCT	0,0001	0,003647
XLOC_023 487	NMU	chr4:56461395-56502865	13,532	1,440	3,23	CRC > NCT	0,0005	0,0123209
XLOC_019 203	COL9A3	chr20:61448113-61493115	14,798	1,584	3,22	CRC > NCT	0,00045	0,0114127
XLOC_032 392	SLC28A3	chr9:86885662-86983440	1,290	0,141	3,20	CRC > NCT	5,00E-05	0,0020443
XLOC_024 287	ITGA2	chr5:52285115-52410959	9,311	1,021	3,19	CRC > NCT	0,0037	0,0490331
XLOC_007 555	PHLDA1	chr12:76419192-76435278	40,451	4,460	3,18	CRC > NCT	5,00E-05	0,0020443
XLOC_018 853	TRIB3	chr20:361272-378203	33,814	3,793	3,16	CRC > NCT	5,00E-05	0,0020443
XLOC_021 076	TDGF1	chr3:46552180-46624005	8,312	0,953	3,13	CRC > NCT	0,00105	0,0206545
XLOC_029 940	CLEC5A	chr7:141627156-141647022	1,169	0,135	3,11	CRC > NCT	0,00055	0,0131101
XLOC_007 560	E2F7	chr12:77413513-77459360	1,257	0,145	3,11	CRC > NCT	0,00055	0,0131101
XLOC_016 164	FUT1	chr19:49251267-49258647	1,398	0,167	3,07	CRC > NCT	0,0013	0,0239449
XLOC_000 261	TRNP1	chr1:27320152-27329011	7,834	0,936	3,06	CRC > NCT	0,0001	0,003647
XLOC_031 239	TNFRSF11B	chr8:119935795-119964407	14,019	1,719	3,03	CRC > NCT	5,00E-05	0,0020443
XLOC_007 255	ERP27	chr12:15066893-15091483	12,039	1,511	2,99	CRC > NCT	0,00015	0,0050841
XLOC_006 146	TRIM29	chr11:119981991-120008863	32,101	4,057	2,98	CRC > NCT	5,00E-05	0,0020443
XLOC_006 097	TMPRSS5	chr11:113533115-113577144	1,711	0,218	2,97	CRC > NCT	0,0003	0,0084946
XLOC_014 829	TNFSF9	chr19:6531009-6536166	10,811	1,378	2,97	CRC > NCT	0,0017	0,0290257
XLOC_031 100	SBSPON	chr8:73969956-74005642	3,987	0,512	2,96	CRC > NCT	5,00E-05	0,0020443
XLOC_008 571	DACH1	chr13:72010961-72463132	5,936	0,763	2,96	CRC > NCT	5,00E-05	0,0020443
XLOC_030 992	LINC01605	chr8:37261041-37411742	9,502	1,226	2,95	CRC > NCT	0,0003	0,0084946
XLOC_020 965	KCNH8	chr3:19189952-19577973	1,246	0,162	2,94	CRC > NCT	0,0002	0,006387
XLOC_008 489	VWA8	chr13:42140733-42555701	19,197	2,504	2,94	CRC > NCT	5,00E-05	0,0020443
XLOC_021 212	ROBO2	chr3:77089293-77699114	0,466	0,061	2,93	CRC > NCT	0,00025	0,0074979
XLOC_023 171	HSPA4L	chr4:128703424-128762402	3,380	0,453	2,90	CRC > NCT	0,0001	0,003647
XLOC_000 285	PTPRU	chr1:29563027-29653325	3,534	0,475	2,89	CRC > NCT	0,0004	0,01042
XLOC_017 637	LOC102723854	chr2:43192835-43266845	7,988	1,078	2,89	CRC > NCT	0,0009	0,0185023
XLOC_001 638	NR0B2	chr1:27237974-27240567	3,431	0,463	2,89	CRC > NCT	0,0008	0,0171221
XLOC_020 278	RAB36	chr22:23487512-23507802	1,715	0,232	2,88	CRC > NCT	0,0003	0,0084946
XLOC_026 125	EVADR	chr6:71093877-71109159	38,742	5,277	2,88	CRC > NCT	0,0005	0,0123209
XLOC_007 509	PPM1H	chr12:63037601-63328665	5,706	0,785	2,86	CRC > NCT	5,00E-05	0,0020443
XLOC_016 553	MYCN	chr2:16076386-16087129	1,141	0,158	2,86	CRC > NCT	0,0013	0,0239449
XLOC_023 529	CXCL5	chr4:74861358-74864446	12,056	1,676	2,85	CRC > NCT	0,00245	0,0372298
XLOC_032 530	RNF183	chr9:116059372-116065415	3,699	0,521	2,83	CRC > NCT	0,00015	0,0050841

XLOC_015692	PRR36	chr19:7933475-7939326	0,953	0,135	2,82	CRC > NCT	0,00045	0,0114127
XLOC_030790	MAPK15	chr8:144797933-144804633	1,231	0,174	2,82	CRC > NCT	0,00225	0,0351876
XLOC_008363	ATP11A	chr13:113344308-113541526	4,880	0,717	2,77	CRC > NCT	0,0003	0,0084946
XLOC_031090	SLCO5A1	chr8:70579285-70750023	0,447	0,066	2,77	CRC > NCT	0,00025	0,0074979
XLOC_020285	MMP11	chr22:24113368-24126509	15,529	2,367	2,71	CRC > NCT	5,00E-05	0,0020443
XLOC_029922	ZC3HAV1L	chr7:138706249-138721778	3,875	0,593	2,71	CRC > NCT	0,00115	0,0220507
XLOC_011615	LRRC36	chr16:67360746-67419524	1,134	0,175	2,70	CRC > NCT	0,0011	0,0213843
XLOC_033636	ASB9	chrX:15262108-15288589	6,553	1,011	2,70	CRC > NCT	5,00E-05	0,0020443
XLOC_020319	ZNRF3	chr22:29279754-29453476	4,185	0,649	2,69	CRC > NCT	5,00E-05	0,0020443
XLOC_002473	KIF14	chr1:200520624-200589862	1,757	0,274	2,68	CRC > NCT	0,00025	0,0074979
XLOC_032211	CDKN2A	chr9:21967137-21994610	9,631	1,514	2,67	CRC > NCT	0,00095	0,0192909
XLOC_032174	FREM1	chr9:14734663-14910993	3,618	0,584	2,63	CRC > NCT	0,00215	0,0341212
XLOC_002097	REG4	chr1:120336640-120355341	973,767	158,797	2,62	CRC > NCT	5,00E-05	0,0020443
XLOC_017756	FBXO41	chr2:73481071-73513456	1,357	0,224	2,60	CRC > NCT	0,0029	0,0416718
XLOC_029960	FAM131B	chr7:143013218-143059840	0,864	0,144	2,59	CRC > NCT	0,00115	0,0220507
XLOC_020782	CELSR1	chr22:46754006-46933067	2,610	0,435	2,59	CRC > NCT	0,00105	0,0206545
XLOC_006647	ASIC1	chr12:50451109-50477544	2,575	0,430	2,58	CRC > NCT	0,0006	0,0139198
XLOC_016557	VSNL1	chr2:17719277-17837706	10,279	1,728	2,57	CRC > NCT	5,00E-05	0,0020443
XLOC_019271	LZTS3	chr20:3143262-3154238	7,539	1,276	2,56	CRC > NCT	5,00E-05	0,0020443
XLOC_020950	SLC6A6	chr3:14444065-14644479	10,530	1,800	2,55	CRC > NCT	0,00025	0,0074979
XLOC_025036	HOMER1	chr5:78668510-78810251	1,655	0,283	2,55	CRC > NCT	0,0038	0,049887
XLOC_011849	PRSS33	chr16:2833951-2837684	2,366	0,405	2,55	CRC > NCT	0,00235	0,0361928
XLOC_021541	ECT2	chr3:172468474-172539277	19,961	3,538	2,50	CRC > NCT	5,00E-05	0,0020443
XLOC_013794	AXIN2	chr17:63524677-63557745	32,673	5,808	2,49	CRC > NCT	5,00E-05	0,0020443
XLOC_026402	SLC22A3	chr6:160769359-160880191	6,391	1,137	2,49	CRC > NCT	0,00045	0,0114127
XLOC_032238	AQP3	chr9:33441143-33447631	32,604	5,858	2,48	CRC > NCT	5,00E-05	0,0020443
XLOC_006574	ARNTL2,SMCO2	chr12:27485786-27655165	4,127	0,751	2,46	CRC > NCT	5,00E-05	0,0020443
XLOC_030012	XRCC2	chr7:152338500-152373250	1,042	0,191	2,45	CRC > NCT	0,001	0,0199726
XLOC_017157	CDCA7	chr2:174192078-174233718	38,359	7,068	2,44	CRC > NCT	5,00E-05	0,0020443
XLOC_014812	UHRF1	chr19:4903091-4962630	3,120	0,577	2,44	CRC > NCT	0,0002	0,006387
XLOC_003464	PALD1	chr10:72237661-72328207	8,821	1,635	2,43	CRC > NCT	5,00E-05	0,0020443
XLOC_030455	FZD3	chr8:28351721-28432056	3,853	0,718	2,42	CRC > NCT	0,00225	0,0351876
XLOC_018891	SMOX	chr20:4129425-4168411	9,727	1,823	2,42	CRC > NCT	5,00E-05	0,0020443
XLOC_028881	ANLN	chr7:36363574-36493401	11,591	2,181	2,41	CRC > NCT	0,0009	0,0185023
XLOC_006592	BICD1	chr12:32259615-32536558	2,526	0,478	2,40	CRC > NCT	0,001	0,0199726
XLOC_011778	MC1R,TUBB3	chr16:89981505-90002507	24,602	4,668	2,40	CRC > NCT	5,00E-05	0,0020443
XLOC_011437	SBK1	chr16:28303839-28335170	0,732	0,140	2,39	CRC > NCT	0,0028	0,0405866
XLOC_001229	PROX1	chr1:213991105-214214847	2,736	0,524	2,39	CRC > NCT	0,0002	0,006387

XLOC_021965	CADPS	chr3:62384018-62861573	5,784	1,109	2,38	CRC > NCT	0,00115	0,0220507
XLOC_024507	SLC12A2	chr5:127270364-127525691	60,888	11,779	2,37	CRC > NCT	5,00E-05	0,0020443
XLOC_003883	NEBL	chr10:21068900-21464625	10,268	1,997	2,36	CRC > NCT	5,00E-05	0,0020443
XLOC_019982	URB1	chr21:33653321-33765312	2,968	0,580	2,36	CRC > NCT	0,00025	0,0074979
XLOC_023489	PPAT	chr4:57259526-57301802	5,407	1,070	2,34	CRC > NCT	5,00E-05	0,0020443
XLOC_003585	CEP55	chr10:95256358-95290536	17,203	3,420	2,33	CRC > NCT	5,00E-05	0,0020443
XLOC_029218	HILPDA	chr7:128092260-128110518	21,893	4,398	2,32	CRC > NCT	0,00055	0,0131101
XLOC_017411	SH3BP4	chr2:235860627-235964432	9,638	1,942	2,31	CRC > NCT	0,0011	0,0213843
XLOC_029778	TFR2	chr7:100218038-100239351	1,106	0,223	2,31	CRC > NCT	0,00145	0,0259223
XLOC_010513	ALPK3	chr15:85359910-85416793	2,278	0,461	2,31	CRC > NCT	0,00175	0,0294656
XLOC_019198	SLCO4A1	chr20:61268734-61304370	12,880	2,612	2,30	CRC > NCT	0,00025	0,0074979
XLOC_003264	MCM10	chr10:13203553-13253191	2,648	0,540	2,30	CRC > NCT	0,00025	0,0074979
XLOC_029781	EPHB4	chr7:100399470-100425986	22,419	4,582	2,29	CRC > NCT	5,00E-05	0,0020443
XLOC_031107	JPH1	chr8:75146933-75233713	7,069	1,449	2,29	CRC > NCT	0,0001	0,003647
XLOC_028820	STK31	chr7:23749785-23872130	0,878	0,183	2,26	CRC > NCT	0,0016	0,0279196
XLOC_030690	CTHRC1	chr8:104373819-104395232	16,443	3,435	2,26	CRC > NCT	0,0013	0,0239449
XLOC_017933	CKAP2L	chr2:113483689-113522254	3,204	0,671	2,26	CRC > NCT	0,00015	0,0050841
XLOC_009625	BMP4	chr14:54416454-54423554	37,571	7,887	2,25	CRC > NCT	5,00E-05	0,0020443
XLOC_001231	CENPF	chr1:214776419-214842730	6,583	1,387	2,25	CRC > NCT	5,00E-05	0,0020443
XLOC_003955	ZNF239	chr10:44050417-44070189	3,139	0,662	2,24	CRC > NCT	0,00095	0,0192909
XLOC_026346	SAMD5	chr6:147829762-147891425	7,519	1,594	2,24	CRC > NCT	5,00E-05	0,0020443
XLOC_034002	ZNF280C	chrX:129330297-129402922	1,106	0,235	2,24	CRC > NCT	0,0031	0,0433833
XLOC_017935	IL1B	chr2:113587321-113624193	113,147	24,023	2,24	CRC > NCT	0,0002	0,006387
XLOC_024533	TCF7	chr5:133450401-133488751	10,638	2,296	2,21	CRC > NCT	5,00E-05	0,0020443
XLOC_025793	SOX4	chr6:21589689-21602655	23,560	5,116	2,20	CRC > NCT	5,00E-05	0,0020443
XLOC_029658	STX1A	chr7:73113533-73134017	4,068	0,885	2,20	CRC > NCT	0,0007	0,0155855
XLOC_026363	MTHFD1L	chr6:151186814-151423322	16,384	3,578	2,19	CRC > NCT	0,0003	0,0084946
XLOC_015315	SULT2B1	chr19:49055410-49102686	19,507	4,289	2,19	CRC > NCT	0,0006	0,0139198
XLOC_005176	DDIAS	chr11:82612736-82645700	2,613	0,575	2,18	CRC > NCT	0,0008	0,0171221
XLOC_030452	ESCO2	chr8:27632057-27662607	4,438	0,980	2,18	CRC > NCT	0,00045	0,0114127
XLOC_000742	PHGDH	chr1:120204832-120286850	39,124	8,686	2,17	CRC > NCT	0,00015	0,0050841
XLOC_018885	CDC25B	chr20:3767358-3786787	39,682	8,811	2,17	CRC > NCT	5,00E-05	0,0020443
XLOC_033756	SHROOM4	chrX:50329787-50557283	1,937	0,430	2,17	CRC > NCT	0,0005	0,0123209
XLOC_019604	SRMS	chr20:62169669-62180512	4,187	0,939	2,16	CRC > NCT	0,0009	0,0185023
XLOC_016887	NCAPH	chr2:97001478-97042833	6,564	1,474	2,15	CRC > NCT	0,0004	0,01042
XLOC_003396	ZNF488	chr10:48333006-48373867	1,144	0,257	2,15	CRC > NCT	0,00325	0,0448628
XLOC_030782	RHPN1	chr8:144451009-144466392	4,597	1,047	2,13	CRC > NCT	0,0001	0,003647
XLOC_001148	LGR6	chr1:202162918-202290712	3,229	0,738	2,13	CRC > NCT	0,00145	0,0259223

XLOC_024547	TGFBI	chr5:135364583-135399507	251,604	57,543	2,13	CRC > NCT	0,0012	0,0226522
XLOC_005097	MYEOV	chr11:69061543-69187280	14,223	3,253	2,13	CRC > NCT	0,00015	0,0050841
XLOC_007712	TBX3	chr12:115107940-115121969	8,788	2,014	2,13	CRC > NCT	5,00E-05	0,0020443
XLOC_031944	COL27A1	chr9:116916589-117074800	4,724	1,083	2,13	CRC > NCT	0,0002	0,006387
XLOC_029814	PUS7	chr7:105096616-105162708	6,231	1,433	2,12	CRC > NCT	5,00E-05	0,0020443
XLOC_002558	NEK2	chr1:211831598-211849154	10,199	2,356	2,11	CRC > NCT	0,0003	0,0084946
XLOC_017156	ZAK	chr2:173938337-174146764	13,443	3,109	2,11	CRC > NCT	0,00085	0,0178569
XLOC_013030	SOX9	chr17:70067124-70173130	35,928	8,340	2,11	CRC > NCT	5,00E-05	0,0020443
XLOC_029901	PODXL	chr7:131184762-131241381	12,737	2,960	2,11	CRC > NCT	5,00E-05	0,0020443
XLOC_002261	IQGAP3	chr1:156495029-156542396	5,475	1,278	2,10	CRC > NCT	0,00025	0,0074979
XLOC_031057	CA8	chr8:61097823-61194099	4,507	1,053	2,10	CRC > NCT	0,00215	0,0341212
XLOC_030743	MIR1204,MIR1207,PVT1	chr8:128806740-129113502	11,875	2,778	2,10	CRC > NCT	0,00255	0,0382395
XLOC_023013	CXCL1	chr4:74733931-74737633	79,540	18,677	2,09	CRC > NCT	0,0005	0,0123209
XLOC_031242	DSCC1	chr8:120846180-120868207	5,339	1,264	2,08	CRC > NCT	0,0009	0,0185023
XLOC_028757	FSCN1	chr7:5632435-5646307	24,151	5,722	2,08	CRC > NCT	5,00E-05	0,0020443
XLOC_003592	HELLS	chr10:96305523-96373681	3,537	0,848	2,06	CRC > NCT	0,0016	0,0279196
XLOC_031252	ATAD2	chr8:124332089-124428623	7,736	1,855	2,06	CRC > NCT	5,00E-05	0,0020443
XLOC_030766	PTP4A3	chr8:142384460-142517330	12,766	3,069	2,06	CRC > NCT	5,00E-05	0,0020443
XLOC_008139	RFC3	chr13:34392198-34540695	10,938	2,640	2,05	CRC > NCT	0,0021	0,0336498
XLOC_018286	AP1S3	chr2:224614479-224702688	3,857	0,931	2,05	CRC > NCT	0,0005	0,0123209
XLOC_026041	PTK7	chr6:43044005-43129458	15,655	3,781	2,05	CRC > NCT	5,00E-05	0,0020443
XLOC_001713	CLSPN	chr1:36186116-36235665	1,482	0,358	2,05	CRC > NCT	0,0016	0,0279196
XLOC_006432	TEAD4	chr12:3065875-3155189	11,189	2,705	2,05	CRC > NCT	0,0007	0,0155855
XLOC_024707	RANBP17	chr5:170288885-170727437	1,382	0,334	2,05	CRC > NCT	0,00335	0,0457925
XLOC_030441	CDCA2	chr8:25316512-25369235	4,107	0,996	2,04	CRC > NCT	0,0004	0,01042
XLOC_032541	TNFSF15	chr9:117546800-117568721	3,205	0,778	2,04	CRC > NCT	0,0004	0,01042
XLOC_010200	ARHGAP11A	chr15:32878335-32932262	6,053	1,473	2,04	CRC > NCT	0,0001	0,003647
XLOC_013053	CDR2L	chr17:72983726-73001894	5,094	1,241	2,04	CRC > NCT	0,0007	0,0155855
XLOC_029760	AZGP1	chr7:99564347-99578379	57,697	14,144	2,03	CRC > NCT	0,00055	0,0131101
XLOC_033834	PHKA1	chrX:71798610-71934157	2,665	0,655	2,02	CRC > NCT	0,00175	0,0294656
XLOC_020476	GTSE1	chr22:46692637-46730114	5,373	1,324	2,02	CRC > NCT	0,00035	0,0094833
XLOC_018983	TPX2	chr20:30326903-30389606	22,832	5,927	2,02	CRC > NCT	5,00E-05	0,0020443
XLOC_001338	EXO1	chr1:242011489-242055511	3,679	0,909	2,02	CRC > NCT	0,00105	0,0206545
XLOC_003476	DDIT4	chr10:74033676-74035797	139,058	34,408	2,01	CRC > NCT	5,00E-05	0,0020443
XLOC_000372	CTPS1	chr1:41444970-41478356	16,135	4,005	2,01	CRC > NCT	0,0001	0,003647
XLOC_016530	RRM2	chr2:10260596-10272759	28,711	7,219	1,99	CRC > NCT	5,00E-05	0,0020443
XLOC_023806	AADAT	chr4:170981372-171011538	4,151	1,045	1,99	CRC > NCT	0,0021	0,0336498
XLOC_018965	GINS1	chr20:25388322-25429677	5,608	1,412	1,99	CRC > NCT	0,0005	0,0123209

XLOC_007380	FAIM2	chr12:50260678-50297996	2,926	0,742	1,98	CRC > NCT	0,0014	0,0253013
XLOC_013626	KIF18B	chr17:43002078-43025119	3,387	0,859	1,98	CRC > NCT	0,002	0,03265
XLOC_013505	TOP2A	chr17:38544769-38574202	23,521	6,009	1,97	CRC > NCT	5,00E-05	0,0020443
XLOC_017923	BUB1	chr2:111393695-111435684	10,833	2,776	1,96	CRC > NCT	0,00015	0,0050841
XLOC_033355	CENPI	chrX:100353166-100421533	1,922	0,494	1,96	CRC > NCT	0,00145	0,0259223
XLOC_014360	PMAIP1	chr18:57567191-57571544	7,009	1,802	1,96	CRC > NCT	0,0024	0,0366226
XLOC_006791	HMGA2	chr12:66151799-66360389	5,173	1,334	1,96	CRC > NCT	0,0018	0,0301682
XLOC_005101	ANO1	chr11:69812163-70035652	7,750	2,002	1,95	CRC > NCT	0,00035	0,0094833
XLOC_003425	CDK1	chr10:62538088-62561445	36,070	9,346	1,95	CRC > NCT	5,00E-05	0,0020443
XLOC_030581	SULF1	chr8:70378858-70573216	17,048	4,420	1,95	CRC > NCT	0,00285	0,0411153
XLOC_010286	SORD	chr15:45315272-45369368	28,499	7,423	1,94	CRC > NCT	5,00E-05	0,0020443
XLOC_006918	PARPBP	chr12:102513858-102592307	5,270	1,373	1,94	CRC > NCT	0,0013	0,0239449
XLOC_006784	XPOT	chr12:64798152-64844902	17,775	4,632	1,94	CRC > NCT	5,00E-05	0,0020443
XLOC_014225	APCDD1	chr18:10454624-10489932	28,592	7,493	1,93	CRC > NCT	0,0036	0,0481629
XLOC_015120	WDR62	chr19:36545782-36596056	1,863	0,493	1,92	CRC > NCT	0,002	0,03265
XLOC_030521	MCM4	chr8:48872762-48891859	24,658	6,541	1,91	CRC > NCT	5,00E-05	0,0020443
XLOC_010225	BUB1B,PAK6	chr15:40453209-40569689	9,323	2,484	1,91	CRC > NCT	5,00E-05	0,0020443
XLOC_029499	OSBPL3	chr7:24836036-25020200	4,755	1,268	1,91	CRC > NCT	0,00015	0,0050841
XLOC_018335	HJURP	chr2:234745121-234763212	7,567	2,017	1,91	CRC > NCT	0,0006	0,0139198
XLOC_031171	FSBP,RAD54B	chr8:95384187-95487343	3,684	0,984	1,91	CRC > NCT	0,00235	0,0361928
XLOC_030031	NCAPG2	chr7:158420869-158497522	8,420	2,249	1,90	CRC > NCT	0,00025	0,0074979
XLOC_033161	JADE3	chrX:46771710-46920641	3,492	0,933	1,90	CRC > NCT	0,00095	0,0192909
XLOC_002661	AGT	chr1:230838271-230850336	7,026	1,878	1,90	CRC > NCT	0,0008	0,0171221
XLOC_009153	TTC9	chr14:71108503-71142077	1,549	0,414	1,90	CRC > NCT	0,00375	0,0494799
XLOC_031943	ZNF618	chr9:116638561-116818992	1,458	0,391	1,90	CRC > NCT	0,0027	0,0395776
XLOC_023632	CENPE	chr4:104026936-104119566	2,780	0,749	1,89	CRC > NCT	0,0003	0,0084946
XLOC_007139	FOXM1	chr12:2945981-2998691	15,800	4,259	1,89	CRC > NCT	0,00275	0,0400849
XLOC_019301	FERMT1	chr20:6040842-6104555	22,638	6,105	1,89	CRC > NCT	0,0001	0,003647
XLOC_006467	ENO2	chr12:7023613-7033271	12,054	3,260	1,89	CRC > NCT	0,00025	0,0074979
XLOC_023684	MAD2L1	chr4:120980578-120991294	24,245	6,574	1,88	CRC > NCT	0,00015	0,0050841
XLOC_005099	CCND1	chr11:69454427-69469393	38,559	10,471	1,88	CRC > NCT	5,00E-05	0,0020443
XLOC_018176	PGAP1	chr2:197697557-197791691	2,109	0,573	1,88	CRC > NCT	0,00025	0,0074979
XLOC_030150	-	chr7:56358509-56359121	18,249	4,975	1,88	CRC > NCT	0,00315	0,0437473
XLOC_029614	PSPH	chr7:56078084-56119268	9,952	2,713	1,88	CRC > NCT	0,00025	0,0074979
XLOC_032423	IARS	chr9:94972489-95056038	34,945	9,568	1,87	CRC > NCT	5,00E-05	0,0020443
XLOC_014329	LIPG	chr18:47086015-47119304	6,836	1,877	1,86	CRC > NCT	0,0008	0,0171221
XLOC_029603	GRB10	chr7:50657759-50861159	7,884	2,166	1,86	CRC > NCT	0,0003	0,0084946
XLOC_012432	FAM57A	chr17:635645-658576	14,009	3,861	1,86	CRC > NCT	0,0033	0,045347

XLOC_018909	PLCB4	chr20:9049437-9461882	27,343	7,541	1,86	CRC > NCT	0,00235	0,0361928
XLOC_023606	TSPAN5	chr4:99391514-99579812	6,767	1,867	1,86	CRC > NCT	0,00065	0,0147056
XLOC_029695	SEMA3A	chr7:83584903-83946127	1,911	0,529	1,85	CRC > NCT	0,0025	0,037738
XLOC_033736	SLC38A5	chrX:48316919-48328644	22,241	6,165	1,85	CRC > NCT	5,00E-05	0,0020443
XLOC_004326	MKI67	chr10:129892992-129924643	10,334	2,882	1,84	CRC > NCT	5,00E-05	0,0020443
XLOC_026153	TTK	chr6:80714282-80753285	7,087	1,977	1,84	CRC > NCT	0,00055	0,0131101
XLOC_029007	GTF2IRD1	chr7:73868119-74017019	16,350	4,563	1,84	CRC > NCT	0,00175	0,0294656
XLOC_000984	NUF2	chr1:163291722-163325553	9,859	2,756	1,84	CRC > NCT	0,00105	0,0206545
XLOC_033924	MORC4	chrX:106182384-106243500	11,041	3,088	1,84	CRC > NCT	5,00E-05	0,0020443
XLOC_006539	PTPRO	chr12:15475190-15751265	9,769	2,735	1,84	CRC > NCT	0,0011	0,0213843
XLOC_017534	WDR35	chr2:20110022-20189913	2,600	0,731	1,83	CRC > NCT	0,00165	0,0284652
XLOC_029008	GTF2I	chr7:74071990-74175022	11,817	3,357	1,82	CRC > NCT	0,00205	0,0331105
XLOC_012788	FKBP10	chr17:39958134-39979469	41,863	11,958	1,81	CRC > NCT	0,0027	0,0395776
XLOC_033583	DKC1	chrX:153991016-154005965	48,998	14,054	1,80	CRC > NCT	5,00E-05	0,0020443
XLOC_001297	URB2	chr1:229761837-229796878	3,620	1,040	1,80	CRC > NCT	0,0026	0,0386713
XLOC_006440	RAD51AP1	chr12:4647949-4669332	6,733	1,934	1,80	CRC > NCT	0,00145	0,0259223
XLOC_001936	SSX2IP	chr1:85108851-85156240	5,371	1,545	1,80	CRC > NCT	5,00E-05	0,0020443
XLOC_032454	ZNF367	chr9:99148224-99180669	3,166	0,912	1,80	CRC > NCT	0,0033	0,045347
XLOC_005135	PLEKHB1	chr11:73357222-73373996	14,952	4,317	1,79	CRC > NCT	0,0003	0,0084946
XLOC_005627	KIF18A	chr11:28042162-28129746	3,140	0,908	1,79	CRC > NCT	0,0031	0,0433833
XLOC_012543	PFAS	chr17:8152591-8173939	3,757	1,089	1,79	CRC > NCT	0,00065	0,0147056
XLOC_033425	PLS3	chrX:114752496-114885179	37,962	11,022	1,78	CRC > NCT	0,00025	0,0074979
XLOC_004283	PDZD8	chr10:119000583-119134978	10,596	3,078	1,78	CRC > NCT	0,0003	0,0084946
XLOC_008439	SLC7A1	chr13:30083387-30169825	12,831	3,730	1,78	CRC > NCT	5,00E-05	0,0020443
XLOC_002593	DUSP10	chr1:221874761-221915518	7,879	2,298	1,78	CRC > NCT	0,00155	0,0273878
XLOC_023848	CFAP97	chr4:186079839-186125182	8,588	2,515	1,77	CRC > NCT	0,00015	0,0050841
XLOC_032239	NOL6	chr9:33461284-33473941	11,024	3,231	1,77	CRC > NCT	5,00E-05	0,0020443
XLOC_008215	CKAP2	chr13:53028914-53050938	11,168	3,290	1,76	CRC > NCT	0,0002	0,006387
XLOC_022285	RPL22L1	chr3:170582664-170588445	71,427	21,197	1,75	CRC > NCT	5,00E-05	0,0020443
XLOC_033287	KIF4A	chrX:69509878-69640774	7,588	2,253	1,75	CRC > NCT	0,0006	0,0139198
XLOC_007469	TIMELESS	chr12:56807385-56843215	7,716	2,293	1,75	CRC > NCT	5,00E-05	0,0020443
XLOC_006755	SHMT2	chr12:57623284-57634500	76,399	22,756	1,75	CRC > NCT	5,00E-05	0,0020443
XLOC_011006	PRC1	chr15:91509266-91537881	20,049	5,976	1,75	CRC > NCT	5,00E-05	0,0020443
XLOC_022960	PAICS	chr4:57301914-57327534	49,488	14,805	1,74	CRC > NCT	5,00E-05	0,0020443
XLOC_022759	FGFRL1	chr4:1000611-1020687	12,378	3,705	1,74	CRC > NCT	0,0037	0,0490331
XLOC_019809	CHAF1B	chr21:37757671-37791313	5,358	1,605	1,74	CRC > NCT	0,0021	0,0336498
XLOC_021371	MCM2	chr3:127317199-127341278	15,995	4,799	1,74	CRC > NCT	5,00E-05	0,0020443
XLOC_031711	MELK	chr9:36572858-36677683	12,228	3,681	1,73	CRC > NCT	0,00065	0,0147056

XLOC_020631	LIF	chr22:30636434-30654020	9,164	2,759	1,73	CRC > NCT	0,00035	0,0094833
XLOC_020225	ZNF74	chr22:20748404-20763792	2,896	0,874	1,73	CRC > NCT	0,0037	0,0490331
XLOC_030573	RRS1	chr8:67331821-67344164	21,909	6,622	1,73	CRC > NCT	5,00E-05	0,0020443
XLOC_021725	NUP210	chr3:13357727-13461819	13,039	3,945	1,72	CRC > NCT	0,00075	0,0164378
XLOC_030961	PBK	chr8:27667127-27695612	15,502	4,699	1,72	CRC > NCT	0,0006	0,0139198
XLOC_000657	GPSM2	chr1:109419268-109506121	16,508	5,030	1,71	CRC > NCT	0,00195	0,0320923
XLOC_011878	TFAP4	chr16:4307186-4323001	8,139	2,486	1,71	CRC > NCT	0,00165	0,0284652
XLOC_004910	FAM111B	chr11:58638531-58897176	6,020	1,849	1,70	CRC > NCT	0,0012	0,0226522
XLOC_031224	NUDCD1	chr8:110222708-110346350	14,117	4,337	1,70	CRC > NCT	0,0003	0,0084946
XLOC_032693	QSOX2	chr9:139098176-139137687	4,963	1,527	1,70	CRC > NCT	0,00095	0,0192909
XLOC_026332	ADGRG6	chr6:142623001-142767403	6,135	1,889	1,70	CRC > NCT	0,0004	0,01042
XLOC_017682	PNPT1	chr2:55860888-55921125	12,693	3,927	1,69	CRC > NCT	0,0001	0,003647
XLOC_009630	WDHD1	chr14:55405617-55493819	2,590	0,802	1,69	CRC > NCT	0,00205	0,0331105
XLOC_009861	JAG2	chr14:105607317-105635161	3,781	1,173	1,69	CRC > NCT	0,00055	0,0131101
XLOC_031653	MTAP	chr9:21802518-21914861	7,313	2,275	1,68	CRC > NCT	5,00E-05	0,0020443
XLOC_016623	WDR43	chr2:29117508-29171392	18,782	5,845	1,68	CRC > NCT	5,00E-05	0,0020443
XLOC_019099	UBE2C	chr20:44441214-44445596	69,163	21,587	1,68	CRC > NCT	0,00035	0,0094833
XLOC_019302	-	chr20:6194322-6196306	7,394	2,309	1,68	CRC > NCT	0,00315	0,0437473
XLOC_029601	FIGNL1	chr7:50503096-50518088	4,199	1,315	1,68	CRC > NCT	0,0024	0,0366226
XLOC_007388	SLC11A2	chr12:51373267-51422058	21,260	6,660	1,67	CRC > NCT	5,00E-05	0,0020443
XLOC_026271	TPD52L1	chr6:125474874-125584805	26,849	8,415	1,67	CRC > NCT	0,0004	0,01042
XLOC_015084	GRAMD1A	chr19:35487146-35517389	21,263	6,716	1,66	CRC > NCT	0,00015	0,0050841
XLOC_010524	AEN	chr15:89152028-89175555	13,984	4,422	1,66	CRC > NCT	0,00015	0,0050841
XLOC_012767	CDC6	chr17:38443972-38460910	10,357	3,276	1,66	CRC > NCT	0,00035	0,0094833
XLOC_022801	WFS1	chr4:6271573-6305083	16,041	5,079	1,66	CRC > NCT	5,00E-05	0,0020443
XLOC_003449	DDX21	chr10:70707746-70745316	35,645	11,322	1,65	CRC > NCT	0,0001	0,003647
XLOC_014740	ARID3A	chr19:925764-975926	5,111	1,628	1,65	CRC > NCT	0,00115	0,0220507
XLOC_026059	SLC29A1	chr6:44187032-44208572	31,539	10,100	1,64	CRC > NCT	0,0014	0,0253013
XLOC_021589	EPHB3	chr3:184279456-184300198	18,214	5,872	1,63	CRC > NCT	5,00E-05	0,0020443
XLOC_006509	-	chr12:10658058-10660060	34,008	10,999	1,63	CRC > NCT	0,00025	0,0074979
XLOC_032558	PHF19	chr9:123617850-123657174	23,021	7,450	1,63	CRC > NCT	0,00215	0,0341212
XLOC_021850	CDC25A	chr3:48198666-48229892	4,894	1,585	1,63	CRC > NCT	0,0022	0,0346434
XLOC_011614	PLEKHG4	chr16:67282854-67360661	4,525	1,472	1,62	CRC > NCT	0,00295	0,0419277
XLOC_003580	KIF11	chr10:94352824-94415154	7,302	2,394	1,61	CRC > NCT	0,0005	0,0123209
XLOC_013941	PYCR1	chr17:79890258-79895204	64,260	21,082	1,61	CRC > NCT	5,00E-05	0,0020443
XLOC_000733	WDR3	chr1:118472371-118727848	11,949	3,929	1,60	CRC > NCT	0,00015	0,0050841
XLOC_001554	ALDH4A1	chr1:19197923-19229293	9,463	3,119	1,60	CRC > NCT	0,00305	0,0429805
XLOC_020214	CDC45	chr22:19467034-19509171	8,818	2,911	1,60	CRC > NCT	0,0027	0,0395776

XLOC_031904	SMC2	chr9:106856540-106907931	8,008	2,648	1,60	CRC > NCT	0,00015	0,0050841
XLOC_019236	PCMTD2	chr20:62887047-62907671	13,524	4,479	1,59	CRC > NCT	0,00045	0,0114127
XLOC_024332	CCNB1	chr5:68462836-68474571	37,264	12,348	1,59	CRC > NCT	0,0004	0,01042
XLOC_022645	-	chr3:155705121-155706910	54,531	18,075	1,59	CRC > NCT	5,00E-05	0,0020443
XLOC_004940	CCDC86	chr11:60609428-60623444	16,458	5,464	1,59	CRC > NCT	0,0006	0,0139198
XLOC_024894	NUP155	chr5:37286424-37371468	5,529	1,839	1,59	CRC > NCT	0,00025	0,0074979
XLOC_010328	TCF12	chr15:57178367-57585000	16,299	5,423	1,59	CRC > NCT	0,0027	0,0395776
XLOC_022866	NCAPG	chr4:17812435-18023529	7,037	2,342	1,59	CRC > NCT	0,0036	0,0481629
XLOC_004794	CD44	chr11:35154196-35253951	59,844	19,953	1,58	CRC > NCT	5,00E-05	0,0020443
XLOC_021369	PLXNA1	chr3:126701142-126756235	5,826	1,944	1,58	CRC > NCT	5,00E-05	0,0020443
XLOC_032480	TEX10	chr9:102861501-103151206	9,506	3,176	1,58	CRC > NCT	0,00255	0,0382395
XLOC_016076	KCNN4	chr19:44270633-44287236	35,776	11,963	1,58	CRC > NCT	0,0003	0,0084946
XLOC_023172	PLK4	chr4:128802015-128820377	4,053	1,357	1,58	CRC > NCT	0,00295	0,0419277
XLOC_017809	POLR1A	chr2:86247099-86333278	6,045	2,025	1,58	CRC > NCT	5,00E-05	0,0020443
XLOC_008398	XPO4	chr13:21351038-21476986	3,885	1,308	1,57	CRC > NCT	0,0002	0,006387
XLOC_001813	STIL	chr1:47715641-47780566	3,723	1,260	1,56	CRC > NCT	0,0026	0,0386713
XLOC_004963	INCENP	chr11:61891444-61923085	4,586	1,558	1,56	CRC > NCT	0,00065	0,0147056
XLOC_031029	PRKDC	chr8:48685340-48872743	19,958	6,791	1,56	CRC > NCT	5,00E-05	0,0020443
XLOC_024255	SKP2	chr5:36152144-36184145	8,651	2,955	1,55	CRC > NCT	0,0009	0,0185023
XLOC_009130	FUT8	chr14:65754784-66221534	15,279	5,250	1,54	CRC > NCT	5,00E-05	0,0020443
XLOC_013594	BRCA1	chr17:41196311-41277500	4,067	1,398	1,54	CRC > NCT	0,0023	0,035694
XLOC_008526	RCBTB1	chr13:50106078-50159726	6,157	2,118	1,54	CRC > NCT	0,0027	0,0395776
XLOC_033462	UTP14A	chrX:129040091-129067784	17,951	6,185	1,54	CRC > NCT	0,0003	0,0084946
XLOC_000209	EPHB2	chr1:23037272-23247994	14,510	5,001	1,54	CRC > NCT	0,0032	0,0443068
XLOC_008187	LRCH1	chr13:47127276-47327239	11,083	3,821	1,54	CRC > NCT	0,00035	0,0094833
XLOC_004986	C11orf84	chr11:63580845-63595460	8,912	3,073	1,54	CRC > NCT	0,00265	0,0390961
XLOC_007383	RACGAP1	chr12:50382944-50425514	19,529	6,757	1,53	CRC > NCT	5,00E-05	0,0020443
XLOC_032183	HAUS6	chr9:19053134-19103085	3,869	1,341	1,53	CRC > NCT	0,0012	0,0226522
XLOC_003649	NOLC1	chr10:103911932-103923630	39,011	13,539	1,53	CRC > NCT	5,00E-05	0,0020443
XLOC_023987	-	chr4:88812967-88814395	58,982	20,527	1,52	CRC > NCT	0,0004	0,01042
XLOC_019071	MYBL2	chr20:42295658-42345136	22,405	7,815	1,52	CRC > NCT	0,0009	0,0185023
XLOC_015728	DNMT1	chr19:10243898-10311599	12,456	4,348	1,52	CRC > NCT	0,0001	0,003647
XLOC_020648	PATZ1	chr22:31721789-31742561	9,164	3,215	1,51	CRC > NCT	0,0011	0,0213843
XLOC_005363	CHEK1	chr11:125494717-125550793	8,100	2,847	1,51	CRC > NCT	0,0009	0,0185023
XLOC_002807	-	chr1:27533338-27534161	40,514	14,290	1,50	CRC > NCT	0,0005	0,0123209
XLOC_029187	MET	chr7:116312407-116438445	22,040	7,811	1,50	CRC > NCT	5,00E-05	0,0020443
XLOC_000410	KIF2C	chr1:45205480-45233442	11,791	4,183	1,49	CRC > NCT	0,0016	0,0279196
XLOC_011287	KREMEN2,PAQR4	chr16:3013878-3030540	15,369	5,453	1,49	CRC > NCT	0,0025	0,037738

XLOC_026824	MCM3	chr6:52128810-52150691	43,455	15,423	1,49	CRC > NCT	5,00E-05	0,0020443
XLOC_024639	TCOF1	chr5:149737201-149780386	10,644	3,793	1,49	CRC > NCT	0,0001	0,003647
XLOC_013011	KPNA2	chr17:66031685-66043702	64,327	22,973	1,49	CRC > NCT	5,00E-05	0,0020443
XLOC_033958	NKRF	chrX:118722108-118739846	5,808	2,087	1,48	CRC > NCT	0,0023	0,035694
XLOC_033174	TIMP1	chrX:47419263-47479256	402,750	145,138	1,47	CRC > NCT	0,00275	0,0400849
XLOC_019860	RRP1B	chr21:45079431-45116036	8,346	3,008	1,47	CRC > NCT	0,0002	0,006387
XLOC_030507	AP3M2	chr8:42010463-42028904	5,966	2,151	1,47	CRC > NCT	0,00215	0,0341212
XLOC_017329	TTLL4	chr2:219575567-219620269	5,718	2,064	1,47	CRC > NCT	0,00055	0,0131101
XLOC_013876	RHBDF2	chr17:74466867-74497509	6,966	2,517	1,47	CRC > NCT	0,00185	0,0307518
XLOC_013904	CBX4	chr17:77806954-77813213	12,694	4,596	1,47	CRC > NCT	0,00075	0,0164378
XLOC_033086	SCML1	chrX:17755568-17773108	8,689	3,172	1,45	CRC > NCT	0,00335	0,0457925
XLOC_026429	SMOC2	chr6:168841830-169068696	19,444	7,101	1,45	CRC > NCT	0,0014	0,0253013
XLOC_007395	GALNT6	chr12:51745050-51909549	10,440	3,816	1,45	CRC > NCT	0,0004	0,01042
XLOC_019117	CSE1L	chr20:47662782-47713497	46,618	17,049	1,45	CRC > NCT	5,00E-05	0,0020443
XLOC_012394	-	chr16:69790277-69791898	13,153	4,813	1,45	CRC > NCT	0,00295	0,0419277
XLOC_006912	UTP20	chr12:101673902-101783305	3,662	1,347	1,44	CRC > NCT	0,00255	0,0382395
XLOC_017860	SEMA4C	chr2:97525470-97539319	8,721	3,214	1,44	CRC > NCT	0,00355	0,0477391
XLOC_004817	CD82	chr11:44585982-44643437	31,654	11,674	1,44	CRC > NCT	0,0007	0,0155855
XLOC_029715	CDK6	chr7:92234234-92546501	4,918	1,821	1,43	CRC > NCT	0,00015	0,0050841
XLOC_015092	FXYD5	chr19:35645624-35660788	124,024	45,942	1,43	CRC > NCT	0,0001	0,003647
XLOC_017927	ANAPC1	chr2:112524749-112641987	4,065	1,513	1,43	CRC > NCT	0,0008	0,0171221
XLOC_004015	ZWINT	chr10:58117008-58121034	33,955	12,705	1,42	CRC > NCT	0,0004	0,01042
XLOC_019480	TOMM34	chr20:43570770-43589114	34,119	12,799	1,41	CRC > NCT	0,00025	0,0074979
XLOC_022132	RUVBL1	chr3:127799798-128127489	33,980	12,759	1,41	CRC > NCT	0,00165	0,0284652
XLOC_007696	NAA25	chr12:112464490-112547129	7,465	2,811	1,41	CRC > NCT	0,0012	0,0226522
XLOC_001207	DIEXF	chr1:210001108-210031064	3,718	1,401	1,41	CRC > NCT	0,001	0,0199726
XLOC_004581	ATHL1	chr11:289075-296258	10,710	4,052	1,40	CRC > NCT	0,002	0,03265
XLOC_031426	-	chr8:30209156-30210091	34,435	13,034	1,40	CRC > NCT	0,00265	0,0390961
XLOC_029879	RBM28	chr7:127924036-127983962	9,176	3,479	1,40	CRC > NCT	0,00255	0,0382395
XLOC_016226	C19orf48	chr19:51299837-51308230	60,826	23,228	1,39	CRC > NCT	0,0003	0,0084946
XLOC_002492	UBE2T	chr1:202298051-202311104	35,308	13,528	1,38	CRC > NCT	0,0024	0,0366226
XLOC_017506	ODC1	chr2:10580496-10588680	129,165	49,541	1,38	CRC > NCT	0,0002	0,006387
XLOC_008302	IPO5	chr13:98605928-98676645	32,994	12,661	1,38	CRC > NCT	5,00E-05	0,0020443
XLOC_000343	CDCA8	chr1:38158072-38175416	11,987	4,602	1,38	CRC > NCT	0,00185	0,0307518
XLOC_005731	SLC43A1	chr11:57251574-57283279	9,496	3,652	1,38	CRC > NCT	0,003	0,0424399
XLOC_016653	QPCT	chr2:37571752-37600466	18,359	7,088	1,37	CRC > NCT	0,00365	0,0486178
XLOC_033393	PRPS1	chrX:106871609-106894262	18,964	7,321	1,37	CRC > NCT	0,0012	0,0226522
XLOC_012025	SEZ6L2	chr16:29882479-29911036	11,663	4,512	1,37	CRC > NCT	0,00205	0,0331105

XLOC_021 848	SMARCC1	chr3:47626771-47823405	22,532	8,733	1,37	CRC > NCT	5,00E-05	0,0020443
XLOC_024 452	WDR36	chr5:110427869-110466426	5,711	2,214	1,37	CRC > NCT	0,00065	0,0147056
XLOC_004 169	RRP12	chr10:99116331-99185823	9,606	3,737	1,36	CRC > NCT	0,0026	0,0386713
XLOC_002 152	ANP32E	chr1:150190716-150208504	16,068	6,305	1,35	CRC > NCT	0,0005	0,0123209
XLOC_021 372	PODXL2	chr3:127348001-127391653	19,994	7,849	1,35	CRC > NCT	0,00115	0,0220507
XLOC_033 943	-	chrX:110862792-110864747	19,037	7,489	1,35	CRC > NCT	0,00175	0,0294656
XLOC_017 193	DNAJC10	chr2:183580764-183650012	27,839	10,954	1,35	CRC > NCT	0,00015	0,0050841
XLOC_029 881	IMPDH1	chr7:128032330-128050041	25,937	10,223	1,34	CRC > NCT	0,001	0,0199726
XLOC_031 225	SYBU	chr8:110586240-110704020	12,400	4,891	1,34	CRC > NCT	0,0012	0,0226522
XLOC_013 867	EVPL	chr17:74002649-74023519	7,225	2,852	1,34	CRC > NCT	0,0008	0,0171221
XLOC_018 014	MCM6	chr2:136597195-136634047	18,242	7,217	1,34	CRC > NCT	0,00035	0,0094833
XLOC_013 097	BIRC5	chr17:76210276-76221716	16,447	6,517	1,34	CRC > NCT	0,0019	0,0313827
XLOC_003 667	PDCD11	chr10:105156330-105212392	8,136	3,225	1,33	CRC > NCT	0,00115	0,0220507
XLOC_017 400	INPP5D	chr2:233924532-234116550	11,993	4,766	1,33	CRC > NCT	0,0009	0,0185023
XLOC_028 747	TTYH3	chr7:2671602-2704438	31,848	12,689	1,33	CRC > NCT	0,0002	0,006387
XLOC_008 473	PROSER1	chr13:39584001-39612290	14,991	5,990	1,32	CRC > NCT	0,00135	0,0246666
XLOC_011 370	ABCC1	chr16:16043433-16237134	8,309	3,329	1,32	CRC > NCT	0,00035	0,0094833
XLOC_030 731	SQLE	chr8:126010719-126034527	21,522	8,628	1,32	CRC > NCT	0,0024	0,0366226
XLOC_013 268	TP53	chr17:7571719-7606820	30,839	12,374	1,32	CRC > NCT	0,00085	0,0178569
**XLOC_024 815**	**LPCAT1**	**chr5:1461538-1524076**	**21,642**	**8,690**	**1,32**	**CRC > NCT**	**0,0008**	**0,0171221**
XLOC_030 025	LMBR1	chr7:156433352-156685910	9,847	3,956	1,32	CRC > NCT	0,00135	0,0246666
XLOC_007 016	MLEC	chr12:121124632-121142494	78,955	31,738	1,31	CRC > NCT	0,00185	0,0307518
XLOC_018 276	CHPF	chr2:220403668-220415317	43,471	17,563	1,31	CRC > NCT	0,00075	0,0164378
XLOC_022 211	HLTF	chr3:148746179-148820610	5,587	2,260	1,31	CRC > NCT	0,00335	0,0457925
XLOC_001 493	SRM	chr1:11114648-11120091	50,639	20,551	1,30	CRC > NCT	0,0006	0,0139198
XLOC_007 556	NAP1L1	chr12:76438573-76478813	112,926	46,033	1,29	CRC > NCT	0,0018	0,0301682
XLOC_017 257	NOP58	chr2:203130313-203168659	53,813	21,978	1,29	CRC > NCT	0,00015	0,0050841
XLOC_016 782	SMYD5	chr2:73441365-73454362	9,942	4,064	1,29	CRC > NCT	0,00285	0,0411153
XLOC_007 112	PGAM5	chr12:133287392-133299323	21,652	8,850	1,29	CRC > NCT	0,0006	0,0139198
XLOC_027 080	LRP11	chr6:150139893-150390574	13,233	5,423	1,29	CRC > NCT	0,0011	0,0213843
XLOC_006 056	DCUN1D5	chr11:102921292-102962944	2,650	1,087	1,29	CRC > NCT	0,00125	0,0233782
XLOC_004 959	MYRF	chr11:61518162-61555992	8,545	3,508	1,28	CRC > NCT	0,0019	0,0313827
XLOC_024 249	BRIX1	chr5:34905365-34926953	41,973	17,234	1,28	CRC > NCT	0,0037	0,0490331
XLOC_023 113	NPNT	chr4:106816074-106892831	15,071	6,190	1,28	CRC > NCT	0,0006	0,0139198
XLOC_005 950	KCNE3	chr11:74165809-74178641	22,645	9,326	1,28	CRC > NCT	0,00105	0,0206545
XLOC_021 493	GMPS	chr3:155588252-155661927	18,523	7,643	1,28	CRC > NCT	0,00125	0,0233782
XLOC_023 087	SMARCAD1	chr4:95128758-95213777	7,841	3,244	1,27	CRC > NCT	0,0023	0,035694
XLOC_011 638	CIRH1A	chr16:69166498-69202937	20,523	8,493	1,27	CRC > NCT	0,00115	0,0220507

XLOC_030493	ADAM9	chr8:38854504-38963086	44,466	18,406	1,27	CRC > NCT	0,00055	0,0131101
XLOC_033474	PHF6	chrX:133507271-133562822	7,556	3,134	1,27	CRC > NCT	0,0019	0,0313827
XLOC_004214	FGF8,NPM3	chr10:103529886-103543170	41,146	17,077	1,27	CRC > NCT	0,0031	0,0433833
XLOC_002679	TARBP1	chr1:234527058-234614928	10,349	4,296	1,27	CRC > NCT	0,0025	0,037738
XLOC_013326	CDRT1,TRIM16	chr17:15468795-15587619	22,815	9,479	1,27	CRC > NCT	0,00115	0,0220507
XLOC_008099	NUPL1	chr13:25875665-25916672	11,321	4,707	1,27	CRC > NCT	0,00105	0,0206545
XLOC_033997	ZDHHC9	chrX:128934725-128977910	21,699	9,039	1,26	CRC > NCT	0,0003	0,0084946
XLOC_022083	LRRC58	chr3:120043316-120068186	4,510	1,881	1,26	CRC > NCT	0,0019	0,0313827
XLOC_021908	RRP9	chr3:51967425-51976075	21,373	8,922	1,26	CRC > NCT	0,00205	0,0331105
XLOC_001541	ESPNP	chr1:17009631-17046652	9,865	4,128	1,26	CRC > NCT	0,002	0,03265
XLOC_019413	AHCY	chr20:32868070-32899608	135,364	56,640	1,26	CRC > NCT	0,00065	0,0147056
XLOC_024751	FGFR4	chr5:176513171-176525143	22,281	9,330	1,26	CRC > NCT	0,00135	0,0246666
XLOC_028859	GARS	chr7:30624712-30673653	77,377	32,472	1,25	CRC > NCT	0,00025	0,0074979
XLOC_006045	TMEM123	chr11:102267055-102332774	79,583	33,572	1,25	CRC > NCT	0,00015	0,0050841
XLOC_018206	WDR12	chr2:203737677-203852940	16,971	7,200	1,24	CRC > NCT	0,0036	0,0481629
XLOC_026936	ASCC3	chr6:100956070-101331114	9,994	4,240	1,24	CRC > NCT	0,0013	0,0239449
XLOC_019297	GPCPD1	chr20:5524984-5591677	15,871	6,737	1,24	CRC > NCT	0,00185	0,0307518
XLOC_007099	PUS1	chr12:132413744-132428410	20,875	8,870	1,23	CRC > NCT	0,0031	0,0433833
XLOC_010373	HACD3	chr15:65822794-65870701	34,426	14,639	1,23	CRC > NCT	0,0001	0,003647
XLOC_033701	BCOR	chrX:39909113-40036582	5,480	2,333	1,23	CRC > NCT	0,002	0,03265
XLOC_031829	CKS2	chr9:91924589-91932965	137,175	58,406	1,23	CRC > NCT	0,00135	0,0246666
XLOC_006145	PVRL1	chr11:119508807-119599435	13,216	5,640	1,23	CRC > NCT	0,00295	0,0419277
XLOC_031335	TOP1MT	chr8:144391464-144442147	18,955	8,112	1,22	CRC > NCT	0,00335	0,0457925
XLOC_034016	GPC4	chrX:132433850-132549677	14,094	6,040	1,22	CRC > NCT	0,00125	0,0233782
XLOC_021514	SMC4	chr3:159974773-160152788	16,872	7,318	1,21	CRC > NCT	0,0022	0,0346434
XLOC_021571	YEATS2	chr3:183415588-183530798	4,957	2,150	1,20	CRC > NCT	0,0033	0,045347
XLOC_033057	PRPS2	chrX:12809473-12843667	27,006	11,757	1,20	CRC > NCT	0,00075	0,0164378
XLOC_006803	NUP107	chr12:69080674-69139578	15,274	6,655	1,20	CRC > NCT	0,0017	0,0290257
XLOC_001551	RCC2	chr1:17733250-17766262	39,402	17,173	1,20	CRC > NCT	0,00015	0,0050841
XLOC_024965	PLK2	chr5:57749806-57755966	13,537	5,901	1,20	CRC > NCT	0,00275	0,0400849
XLOC_007496	CDK4	chr12:58137114-58146504	79,263	34,568	1,20	CRC > NCT	0,00325	0,0448628
XLOC_020906	BHLHE40	chr3:4938492-5027119	61,759	27,007	1,19	CRC > NCT	0,00295	0,0419277
XLOC_032147	KIAA0020	chr9:2769579-2844130	25,739	11,272	1,19	CRC > NCT	0,00145	0,0259223
XLOC_018015	DARS	chr2:136662634-136765134	28,102	12,331	1,19	CRC > NCT	0,0011	0,0213843
XLOC_031152	NBN	chr8:90945434-90996952	13,379	5,874	1,19	CRC > NCT	0,0016	0,0279196
XLOC_002623	PARP1	chr1:226548391-226595801	33,321	14,634	1,19	CRC > NCT	0,00035	0,0094833
XLOC_030691	DCAF13	chr8:104410865-104455680	27,535	12,094	1,19	CRC > NCT	0,0033	0,045347
XLOC_004704	IPO7	chr11:9405995-9469678	24,768	10,957	1,18	CRC > NCT	0,0005	0,0123209

XLOC_033 858	ATRX	chrX:76759460-77041755	4,678	2,073	1,17	CRC > NCT	0,00365	0,0486178
XLOC_006 731	CDK2	chr12:56360549-56366573	15,980	7,080	1,17	CRC > NCT	0,00255	0,0382395
XLOC_011 579	ADGRG1	chr16:57653604-57699430	35,246	15,644	1,17	CRC > NCT	0,0008	0,0171221
XLOC_025 972	HMGA1	chr6:34204576-34218326	189,633	84,495	1,17	CRC > NCT	0,0029	0,0416718
XLOC_029 261	NUP205	chr7:135242517-135333501	18,521	8,314	1,16	CRC > NCT	0,00235	0,0361928
XLOC_016 695	MSH2	chr2:47630205-47710455	11,732	5,268	1,16	CRC > NCT	0,0037	0,0490331
XLOC_005 929	DHCR7	chr11:71145456-71159477	21,799	9,791	1,15	CRC > NCT	0,0021	0,0336498
XLOC_018 940	DTD1	chr20:18568555-18744560	35,334	15,877	1,15	CRC > NCT	0,00355	0,0477391
XLOC_023 038	ANXA3	chr4:79472741-79531605	74,792	33,640	1,15	CRC > NCT	0,0016	0,0279196
XLOC_024 500	LMNB1	chr5:126112314-126172712	33,660	15,244	1,14	CRC > NCT	0,003	0,0424399
XLOC_018 314	NCL	chr2:232319458-232329205	228,281	103,390	1,14	CRC > NCT	0,0011	0,0213843
XLOC_013 568	ACLY	chr17:40023022-40075275	31,318	14,210	1,14	CRC > NCT	0,00345	0,046739
XLOC_029 511	HOXA10,HOXA9	chr7:27201739-27219880	48,824	22,254	1,13	CRC > NCT	0,0029	0,0416718
XLOC_020 287	MIF,SLC2A11	chr22:24198771-24268664	767,542	350,074	1,13	CRC > NCT	0,00175	0,0294656
XLOC_029 544	DPY19L1	chr7:34966246-35225774	9,564	4,372	1,13	CRC > NCT	0,00365	0,0486178
XLOC_001 741	PABPC4	chr1:40026482-40042581	50,121	23,016	1,12	CRC > NCT	0,00235	0,0361928
XLOC_013 572	KAT2A	chr17:40265075-40273382	20,208	9,286	1,12	CRC > NCT	0,00315	0,0437473
XLOC_002 182	S100A11	chr1:152004981-152009511	967,842	445,734	1,12	CRC > NCT	0,0032	0,0443068
XLOC_014 197	TGIF1	chr18:3411924-3458633	45,573	21,172	1,11	CRC > NCT	0,0025	0,037738
XLOC_011 653	SF3B3	chr16:70557532-70613116	14,793	6,882	1,10	CRC > NCT	0,00065	0,0147056
XLOC_028 942	CCT6A	chr7:56119377-56131682	99,257	46,179	1,10	CRC > NCT	0,0009	0,0185023
XLOC_005 232	YAP1	chr11:101980499-102104809	19,443	9,075	1,10	CRC > NCT	0,0024	0,0366226
XLOC_007 090	RAN	chr12:131356520-131362472	102,924	48,150	1,10	CRC > NCT	0,00105	0,0206545
XLOC_015 588	TCF3	chr19:1609285-1652428	20,216	9,468	1,09	CRC > NCT	0,00135	0,0246666
XLOC_011 607	CBFB	chr16:67063012-67134993	16,685	7,855	1,09	CRC > NCT	0,0038	0,049887
XLOC_021 520	NMD3	chr3:160938936-160971766	19,302	9,098	1,09	CRC > NCT	0,00375	0,0494799
XLOC_017 208	WDR75	chr2:190306067-190348086	21,323	10,084	1,08	CRC > NCT	0,003	0,0424399
XLOC_024 709	NPM1	chr5:170814657-170839109	260,622	124,478	1,07	CRC > NCT	0,0015	0,0267114
XLOC_003 359	BMS1	chr10:43274921-43330385	8,973	4,304	1,06	CRC > NCT	0,0023	0,035694
XLOC_000 015	AGRN	chr1:955502-991499	15,230	8,084	1,05	CRC > NCT	0,00215	0,0341212
XLOC_026 033	RPL7L1	chr6:42847360-42858825	23,813	11,478	1,05	CRC > NCT	0,00165	0,0284652
XLOC_029 213	SND1	chr7:127292201-127732659	68,442	33,047	1,05	CRC > NCT	0,0012	0,0226522
XLOC_032 256	SIGMAR1	chr9:34634677-34637823	53,869	26,039	1,05	CRC > NCT	0,00265	0,0390961
XLOC_007 731	GCN1	chr12:120564922-120632515	14,432	6,995	1,04	CRC > NCT	0,00115	0,0220507
XLOC_001 627	STMN1	chr1:26210676-26233368	119,982	58,310	1,04	CRC > NCT	0,00255	0,0382395
XLOC_007 162	CHD4,NOP2	chr12:6666033-6723326	48,342	23,509	1,04	CRC > NCT	0,0026	0,0386713
XLOC_026 896	SYNCRIP	chr6:86316710-86353748	35,167	17,191	1,03	CRC > NCT	0,0027	0,0395776
XLOC_033 104	EIF2S3	chrX:24072719-24097166	59,021	28,982	1,03	CRC > NCT	0,0035	0,0472409

XLOC_006 899	TMPO	chr12:98906750-98944157	33,095	16,256	1,03	CRC > NCT	0,0028	0,0405866
XLOC_006 811	CCT2	chr12:69979207-69995357	141,324	69,639	1,02	CRC > NCT	0,00205	0,0331105
XLOC_011 922	RSL1D1	chr16:11928054-11945442	34,584	17,202	1,01	CRC > NCT	0,00165	0,0284652
XLOC_003 925	KIF5B	chr10:32297176-32345467	46,478	23,137	1,01	CRC > NCT	0,00295	0,0419277
XLOC_008 097	PABPC3	chr13:25670275-25672704	28,096	14,051	1,00	CRC > NCT	0,0038	0,049887
XLOC_017 101	PKP4	chr2:159313391-159539466	17,438	8,777	0,99	CRC > NCT	0,00295	0,0419277
XLOC_022 462	-	chr3:22423206-22424413	124,721	62,888	0,99	CRC > NCT	0,0025	0,037738
XLOC_014 886	ILF3	chr19:10764936-10803098	42,813	21,705	0,98	CRC > NCT	0,00295	0,0419277
XLOC_033 780	HUWE1	chrX:53559056-53713674	11,702	6,022	0,96	CRC > NCT	0,00225	0,0351876
XLOC_008 998	DCAF11	chr14:24583275-24594763	19,632	37,793	-0,94	CRC < NCT	0,0033	0,045347
XLOC_006 560	CMAS	chr12:22199109-22218606	42,334	82,865	-0,97	CRC < NCT	0,00325	0,0448628
XLOC_033 439	UBE2A	chrX:118706917-118719461	45,729	89,589	-0,97	CRC < NCT	0,00335	0,0457925
XLOC_024 743	HIGD2A	chr5:175815783-175816751	161,064	316,375	-0,97	CRC < NCT	0,0036	0,0481629
XLOC_026 151	SH3BGRL2	chr6:80247367-80414079	24,573	48,370	-0,98	CRC < NCT	0,00365	0,0486178
XLOC_011 663	IST1	chr16:71927366-72033877	29,493	58,113	-0,98	CRC < NCT	0,00225	0,0351876
XLOC_029 931	MKRN1	chr7:140152567-140179445	51,766	102,018	-0,98	CRC < NCT	0,00315	0,0437473
XLOC_032 235	B4GALT1	chr9:33110638-33180526	29,236	57,731	-0,98	CRC < NCT	0,00375	0,0494799
XLOC_020 744	NDUFA6	chr22:42481529-42549086	75,250	148,782	-0,98	CRC < NCT	0,0035	0,0472409
XLOC_013 789	GNA13	chr17:62992552-63052920	10,702	21,244	-0,99	CRC < NCT	0,0036	0,0481629
XLOC_019 866	C21orf33	chr21:45553493-45565605	53,636	106,648	-0,99	CRC < NCT	0,00375	0,0494799
XLOC_021 567	B3GNT5	chr3:182895791-183146057	13,749	27,432	-1,00	CRC < NCT	0,00345	0,046739
XLOC_000 888	SLC50A1	chr1:155107819-155111599	47,551	95,581	-1,01	CRC < NCT	0,00375	0,0494799
XLOC_004 184	GOT1	chr10:101156626-101190530	27,403	55,105	-1,01	CRC < NCT	0,00285	0,0411153
XLOC_010 459	IDH3A	chr15:78441676-78527049	23,803	47,881	-1,01	CRC < NCT	0,00205	0,0331105
XLOC_001 548	SDHB	chr1:17345224-17380665	93,814	188,952	-1,01	CRC < NCT	0,00185	0,0307518
XLOC_021 889	UBA7	chr3:49842635-49855514	11,957	24,273	-1,02	CRC < NCT	0,0029	0,0416718
XLOC_000 268	STX12	chr1:28099693-28150963	16,799	34,104	-1,02	CRC < NCT	0,0017	0,0290257
XLOC_017 378	CAB39	chr2:231574872-231685791	30,935	62,947	-1,02	CRC < NCT	0,0021	0,0336498
XLOC_001 423	AURKAIP1	chr1:1309105-1312012	112,320	228,953	-1,03	CRC < NCT	0,003	0,0424399
XLOC_027 257	-	chr6:37012569-37013538	229,540	468,378	-1,03	CRC < NCT	0,00295	0,0419277
XLOC_013 278	VAMP2	chr17:8062462-8066305	18,237	37,268	-1,03	CRC < NCT	0,00345	0,046739
XLOC_010 669	LPCAT4	chr15:34651088-34659395	33,280	68,143	-1,03	CRC < NCT	0,0025	0,037738
XLOC_016 144	NAPA	chr19:47978397-48018515	54,633	112,286	-1,04	CRC < NCT	0,0023	0,035694
XLOC_005 414	SIGIRR	chr11:405708-417403	30,143	62,125	-1,04	CRC < NCT	0,0031	0,0433833
XLOC_007 765	VPS37B	chr12:123349079-123380766	32,077	66,147	-1,04	CRC < NCT	0,0018	0,0301682
XLOC_016 041	RABAC1	chr19:42460824-42463528	113,736	234,548	-1,04	CRC < NCT	0,0026	0,0386713
XLOC_000 258	ZDHHC18	chr1:27153200-27184072	9,466	19,547	-1,05	CRC < NCT	0,0035	0,0472409
XLOC_000 043	RER1	chr1:2252642-2345153	41,188	85,111	-1,05	CRC < NCT	0,00245	0,0372298

XLOC_028 970	ASL	chr7:65540775-65558329	50,090	103,979	-1,05	CRC < NCT	0,0027	0,0395776
XLOC_016 656	GALM	chr2:38893051-38961909	19,679	40,999	-1,06	CRC < NCT	0,0036	0,0481629
XLOC_032 080	RXRA	chr9:137218308-137332831	10,275	21,417	-1,06	CRC < NCT	0,0019	0,0313827
XLOC_033 323	COX7B	chrX:77151252-77160881	619,180	1293,560	-1,06	CRC < NCT	0,00265	0,0390961
XLOC_024 171	SDHA	chr5:201130-257197	60,867	127,239	-1,06	CRC < NCT	0,0025	0,037738
XLOC_020 084	PTTG1IP	chr21:46269499-46293818	92,056	192,533	-1,06	CRC < NCT	0,00165	0,0284652
XLOC_021 865	IP6K2	chr3:48725431-48754901	43,360	90,689	-1,06	CRC < NCT	0,00235	0,0361928
XLOC_013 850	HN1	chr17:73131335-73150778	98,164	205,966	-1,07	CRC < NCT	0,0022	0,0346434
XLOC_010 919	ETFA	chr15:76352298-76604008	110,012	231,029	-1,07	CRC < NCT	0,0012	0,0226522
XLOC_000 395	PTPRF	chr1:43989771-44089538	30,551	64,232	-1,07	CRC < NCT	0,00205	0,0331105
XLOC_030 638	DECR1	chr8:91013529-91095109	86,855	183,301	-1,08	CRC < NCT	0,0018	0,0301682
XLOC_013 218	ATP2A3	chr17:3827159-3867929	25,224	53,325	-1,08	CRC < NCT	0,0026	0,0386713
XLOC_021 352	KALRN	chr3:123752448-124440344	11,544	24,412	-1,08	CRC < NCT	0,00355	0,0477391
XLOC_011 445	SH2B1	chr16:28857864-28885536	13,735	29,047	-1,08	CRC < NCT	0,0031	0,0433833
XLOC_012 801	MLX	chr17:40719077-40730458	31,820	67,296	-1,08	CRC < NCT	0,0012	0,0226522
XLOC_001 893	SLC35D1	chr1:67463957-67520298	7,854	16,629	-1,08	CRC < NCT	0,00225	0,0351876
XLOC_032 717	FBXW5	chr9:139834882-139841429	48,228	102,155	-1,08	CRC < NCT	0,0022	0,0346434
XLOC_008 395	CRYL1	chr13:20977803-21100039	53,526	113,738	-1,09	CRC < NCT	0,0022	0,0346434
XLOC_014 232	IMPA2	chr18:11981426-12033336	34,185	72,698	-1,09	CRC < NCT	0,00155	0,0273878
XLOC_001 583	ECE1	chr1:21543584-21672034	20,830	44,394	-1,09	CRC < NCT	0,0014	0,0253013
XLOC_013 940	MAFG,SIRT7	chr17:79869702-79885593	21,510	46,039	-1,10	CRC < NCT	0,00295	0,0419277
XLOC_026 872	COX7A2	chr6:75947390-75954098	397,934	853,594	-1,10	CRC < NCT	0,00205	0,0331105
XLOC_005 551	TMEM9B	chr11:8968747-9001111	41,671	89,431	-1,10	CRC < NCT	0,00125	0,0233782
XLOC_014 231	CHMP1B	chr18:11689013-11909195	26,614	57,154	-1,10	CRC < NCT	0,00265	0,0390961
XLOC_020 438	SMDT1	chr22:42475694-42480288	25,150	54,131	-1,11	CRC < NCT	0,00205	0,0331105
XLOC_003 681	DUSP5	chr10:112256521-112271337	17,402	37,500	-1,11	CRC < NCT	0,00295	0,0419277
XLOC_001 505	DHRS3	chr1:12627936-12679969	53,289	114,993	-1,11	CRC < NCT	0,00155	0,0273878
XLOC_020 671	APOL2	chr22:36622247-36636000	9,443	20,381	-1,11	CRC < NCT	0,0035	0,0472409
XLOC_024 637	CDX1,SLC6A7	chr5:149546070-149590790	100,799	218,347	-1,12	CRC < NCT	0,0024	0,0366226
XLOC_005 050	CNIH2,RAB1B	chr11:66035863-66051685	79,619	172,485	-1,12	CRC < NCT	0,00125	0,0233782
XLOC_003 616	ZFYVE27	chr10:99496864-99520697	12,653	27,414	-1,12	CRC < NCT	0,0017	0,0290257
XLOC_024 735	SFXN1	chr5:174904845-174956872	14,879	32,275	-1,12	CRC < NCT	0,0021	0,0336498
XLOC_029 087	LMTK2	chr7:97734941-97838952	6,794	14,749	-1,12	CRC < NCT	0,00235	0,0361928
XLOC_006 798	DYRK2	chr12:68041852-68062784	7,926	17,210	-1,12	CRC < NCT	0,00155	0,0273878
XLOC_001 942	BCL10	chr1:85726836-85743771	12,276	26,678	-1,12	CRC < NCT	0,00205	0,0331105
XLOC_011 306	NAA60	chr16:3493665-3537112	21,964	47,749	-1,12	CRC < NCT	0,0016	0,0279196
XLOC_023 323	ATP5I	chr4:666224-668127	661,681	1439,870	-1,12	CRC < NCT	0,00225	0,0351876
XLOC_005 956	ARRB1	chr11:74970597-75062875	5,398	11,750	-1,12	CRC < NCT	0,00145	0,0259223

XLOC_023117	HADH	chr4:108745720-108956331	55,618	121,175	-1,12	CRC < NCT	0,00265	0,0390961
XLOC_017796	SUCLG1	chr2:84650646-84686586	112,563	245,856	-1,13	CRC < NCT	0,00105	0,0206545
XLOC_032282	HINT2	chr9:35812956-35815042	44,221	96,687	-1,13	CRC < NCT	0,00355	0,0477391
XLOC_010306	SLC27A2	chr15:50474392-50528589	17,192	37,641	-1,13	CRC < NCT	0,0022	0,0346434
XLOC_031339	MROH6,NAPRT	chr8:144648356-144661030	55,713	122,118	-1,13	CRC < NCT	0,00175	0,0294656
XLOC_000732	FAM46C	chr1:118148603-118171011	4,699	10,299	-1,13	CRC < NCT	0,0021	0,0336498
XLOC_004639	STIM1	chr11:3875260-4114442	13,726	30,111	-1,13	CRC < NCT	0,00255	0,0382395
XLOC_003810	KLF6	chr10:3818071-3827473	44,478	97,715	-1,14	CRC < NCT	0,00315	0,0437473
XLOC_015025	NDUFA13,YJEFN3	chr19:19624232-19648393	429,247	946,041	-1,14	CRC < NCT	0,0025	0,037738
XLOC_011839	ECI1	chr16:2289255-2301678	50,285	110,829	-1,14	CRC < NCT	0,0016	0,0279196
XLOC_022204	PLOD2	chr3:145787227-145879282	13,072	28,816	-1,14	CRC < NCT	0,00265	0,0390961
XLOC_018171	STK17B	chr2:196998133-197039072	6,584	14,515	-1,14	CRC < NCT	0,0011	0,0213843
XLOC_033531	MTM1	chrX:149737046-149841755	6,277	13,848	-1,14	CRC < NCT	0,00375	0,0494799
XLOC_015106	COX6B1	chr19:36139124-36149686	494,006	1090,040	-1,14	CRC < NCT	0,00315	0,0437473
XLOC_015801	NDUFB7	chr19:14676889-14682889	235,321	519,255	-1,14	CRC < NCT	0,0013	0,0239449
XLOC_033660	SH3KBP1	chrX:19551920-19905744	20,918	46,181	-1,14	CRC < NCT	0,00055	0,0131101
XLOC_009553	EAPP	chr14:34985134-35008943	36,688	81,140	-1,15	CRC < NCT	0,00125	0,0233782
XLOC_005895	CDK2AP2	chr11:67273960-67276199	50,302	111,252	-1,15	CRC < NCT	0,0015	0,0267114
XLOC_026056	TMEM63B	chr6:44095298-44123256	15,325	33,913	-1,15	CRC < NCT	0,00075	0,0164378
XLOC_032014	COQ4	chr9:131084786-131096352	27,746	61,420	-1,15	CRC < NCT	0,00225	0,0351876
XLOC_004989	COX8A	chr11:63742078-63744015	512,575	1135,360	-1,15	CRC < NCT	0,00085	0,0178569
XLOC_031366	TONSL,VPS28	chr8:145648977-145669853	101,442	225,095	-1,15	CRC < NCT	0,00105	0,0206545
XLOC_010478	ABHD17C	chr15:80987651-81047962	49,250	109,352	-1,15	CRC < NCT	0,0008	0,0171221
XLOC_010782	RAB27A	chr15:55495161-55582589	9,470	21,030	-1,15	CRC < NCT	0,002	0,03265
XLOC_025999	CDKN1A	chr6:36644236-36655116	135,341	300,797	-1,15	CRC < NCT	0,0034	0,0463025
XLOC_030446	PTK2B	chr8:27168984-27316910	12,366	27,505	-1,15	CRC < NCT	0,00055	0,0131101
XLOC_002024	EPS8L3	chr1:110292695-110307191	59,113	131,534	-1,15	CRC < NCT	0,00085	0,0178569
XLOC_003826	ASB13	chr10:5680753-5708563	11,825	26,318	-1,15	CRC < NCT	0,0021	0,0336498
XLOC_014836	ARHGEF18	chr19:7420491-7537680	9,973	22,198	-1,15	CRC < NCT	0,003	0,0424399
XLOC_015674	GPR108	chr19:6729924-6737633	23,080	51,390	-1,15	CRC < NCT	0,00055	0,0131101
XLOC_020328	UQCR10	chr22:30116343-30166918	117,269	261,407	-1,16	CRC < NCT	0,0017	0,0290257
XLOC_013076	UBALD2	chr17:74261285-74267379	39,454	87,967	-1,16	CRC < NCT	0,0007	0,0155855
XLOC_012836	GRN	chr17:42422453-42430481	155,158	345,977	-1,16	CRC < NCT	0,00205	0,0331105
XLOC_021450	PLS1	chr3:142315228-142432705	51,962	115,973	-1,16	CRC < NCT	0,0028	0,0405866
XLOC_016734	LGALSL	chr2:64672658-64688518	5,946	13,280	-1,16	CRC < NCT	0,0022	0,0346434
XLOC_015921	CEBPA	chr19:33790601-33793507	24,241	54,410	-1,17	CRC < NCT	0,0027	0,0395776
XLOC_016246	ETFB	chr19:51848408-51869672	104,049	234,149	-1,17	CRC < NCT	0,00105	0,0206545
XLOC_026343	RAB32	chr6:146864827-146876086	30,248	68,179	-1,17	CRC < NCT	0,00165	0,0284652

XLOC_012057	PYCARD	chr16:31210639-31215432	58,600	132,121	-1,17	CRC < NCT	0,00245	0,0372298
XLOC_020363	TOM1	chr22:35695267-35744401	19,429	43,847	-1,17	CRC < NCT	0,00085	0,0178569
XLOC_013496	GSDMB,ORMDL3	chr17:38060845-38083884	89,115	201,147	-1,17	CRC < NCT	0,00065	0,0147056
XLOC_012676	ADAP2,RNF135	chr17:29248753-29326930	12,252	27,656	-1,17	CRC < NCT	0,0038	0,049887
XLOC_010999	AP3S2,ARPIN,C15orf38-AP3S2	chr15:90373648-90456234	14,593	32,974	-1,18	CRC < NCT	0,00035	0,0094833
XLOC_018724	-	chr2:183936524-183936787	631,580	1429,530	-1,18	CRC < NCT	0,0011	0,0213843
XLOC_029006	CLIP2	chr7:73703804-73822852	9,765	22,121	-1,18	CRC < NCT	0,00055	0,0131101
XLOC_004595	EPS8L2,TMEM80	chr11:644206-727729	38,664	87,826	-1,18	CRC < NCT	0,00185	0,0307518
XLOC_014843	STXBP2	chr19:7701986-7712761	46,662	106,027	-1,18	CRC < NCT	0,0005	0,0123209
XLOC_015499	EPN1	chr19:56186505-56249768	54,914	124,846	-1,18	CRC < NCT	0,0011	0,0213843
XLOC_021975	SUCLG2	chr3:67291046-67998136	46,709	106,290	-1,19	CRC < NCT	0,0005	0,0123209
XLOC_015648	PLIN3	chr19:4838309-4867780	43,509	99,167	-1,19	CRC < NCT	0,0003	0,0084946
XLOC_016009	-	chr19:40448541-40450040	13,933	31,814	-1,19	CRC < NCT	0,0024	0,0366226
XLOC_025234	PCDH1	chr5:141229669-141258797	18,722	42,751	-1,19	CRC < NCT	0,00155	0,0273878
XLOC_020591	DDT	chr22:24309025-24322019	83,261	190,187	-1,19	CRC < NCT	0,00175	0,0294656
XLOC_007011	COX6A1	chr12:120867415-120878694	209,909	479,658	-1,19	CRC < NCT	0,0005	0,0123209
XLOC_014542	SMAD7	chr18:46446221-46478429	8,804	20,184	-1,20	CRC < NCT	0,0013	0,0239449
XLOC_000989	MGST3	chr1:165600109-165625372	115,723	266,080	-1,20	CRC < NCT	0,00055	0,0131101
XLOC_000274	SESN2	chr1:28585962-28609007	7,934	18,305	-1,21	CRC < NCT	0,0014	0,0253013
XLOC_025163	SHROOM1	chr5:132157301-132167441	8,177	18,878	-1,21	CRC < NCT	0,00195	0,0320923
XLOC_019011	CHMP4B	chr20:32399109-32442173	163,635	377,883	-1,21	CRC < NCT	0,001	0,0199726
XLOC_017241	SPATS2L	chr2:201170603-201346986	18,072	41,751	-1,21	CRC < NCT	0,0002	0,006387
XLOC_021862	UQCRC1	chr3:48636431-48647098	161,530	373,792	-1,21	CRC < NCT	0,0016	0,0279196
XLOC_014621	PQLC1	chr18:77662358-77711653	16,873	39,073	-1,21	CRC < NCT	0,00095	0,0192909
XLOC_009803	CLMN	chr14:95648273-95786245	5,879	13,169	-1,21	CRC < NCT	0,00265	0,0390961
XLOC_012297	CHMP1A	chr16:89710822-89739686	40,800	94,539	-1,21	CRC < NCT	0,0002	0,006387
XLOC_011465	TMEM219	chr16:29973339-29984375	100,581	233,236	-1,21	CRC < NCT	0,0006	0,0139198
XLOC_006062	CARD16,CARD17,CASP1	chr11:104896115-104972165	45,593	105,731	-1,21	CRC < NCT	0,00285	0,0411153
XLOC_011042	VIMP	chr15:101811075-101817932	62,809	145,699	-1,21	CRC < NCT	0,00025	0,0074979
XLOC_029500	C7orf31,CYCS	chr7:25158167-25220009	38,462	89,227	-1,21	CRC < NCT	0,00055	0,0131101
XLOC_031880	TDRD7	chr9:100174301-100258408	10,151	23,556	-1,21	CRC < NCT	0,001	0,0199726
XLOC_000183	MINOS1,MINOS1-NBL1,NBL1	chr1:19923470-19984949	149,062	346,183	-1,22	CRC < NCT	0,00185	0,0307518
XLOC_000046	TNFRSF14	chr1:2473023-2497277	17,017	39,534	-1,22	CRC < NCT	0,00145	0,0259223
XLOC_014751	MIDN	chr19:1248407-1259145	26,473	61,812	-1,22	CRC < NCT	0,00035	0,0094833
XLOC_009202	CIPC	chr14:77564577-77583754	6,393	14,951	-1,23	CRC < NCT	0,00105	0,0206545
XLOC_009149	SUSD6	chr14:70078309-70182033	9,002	21,061	-1,23	CRC < NCT	0,00045	0,0114127
XLOC_016046	DEDD2	chr19:42701468-42724304	20,355	47,661	-1,23	CRC < NCT	0,0004	0,01042
XLOC_000054	TPRG1L	chr1:3541555-3546696	35,867	84,114	-1,23	CRC < NCT	0,00025	0,0074979

XLOC_004591	RASSF7	chr11:535885-564059	42,924	100,706	-1,23	CRC < NCT	0,0013	0,0239449
XLOC_001611	IFNLR1,IL22RA1	chr1:24446255-24513765	13,805	32,414	-1,23	CRC < NCT	0,00295	0,0419277
XLOC_013362	FAM83G	chr17:18853988-18950465	10,305	24,207	-1,23	CRC < NCT	0,0023	0,035694
XLOC_020294	ADORA2A,SPECC1L	chr22:24666784-24890783	8,932	20,996	-1,23	CRC < NCT	0,0009	0,0185023
XLOC_011401	ANKS4B	chr16:21237525-21329912	11,644	27,421	-1,24	CRC < NCT	0,00235	0,0361928
XLOC_025182	CXCL14	chr5:134906370-134914969	56,021	131,961	-1,24	CRC < NCT	0,00215	0,0341212
XLOC_010904	SCAMP2	chr15:75136019-75165733	53,483	126,017	-1,24	CRC < NCT	0,0002	0,006387
XLOC_005391	ST14	chr11:130029645-130081837	140,472	331,187	-1,24	CRC < NCT	0,0023	0,035694
XLOC_000052	ARHGEF16	chr1:3371143-3397713	19,314	45,551	-1,24	CRC < NCT	0,0004	0,01042
XLOC_014750	ATP5D	chr19:1239374-1244830	154,236	363,814	-1,24	CRC < NCT	0,00095	0,0192909
XLOC_005589	USH1C	chr11:17515441-17565963	81,151	191,845	-1,24	CRC < NCT	0,00175	0,0294656
XLOC_014490	DSC2,DSC3	chr18:28569330-28742819	24,910	58,912	-1,24	CRC < NCT	0,003	0,0424399
XLOC_024267	PTGER4	chr5:40678569-40694043	7,415	17,558	-1,24	CRC < NCT	0,0013	0,0239449
XLOC_006456	CD27,TAPBPL	chr12:6548166-6582174	26,671	63,217	-1,25	CRC < NCT	0,0034	0,0463025
XLOC_011995	ARHGAP17	chr16:24930695-25026704	13,648	32,395	-1,25	CRC < NCT	0,0001	0,003647
XLOC_012509	ACADVL	chr17:7093209-7138248	138,714	329,607	-1,25	CRC < NCT	0,0013	0,0239449
XLOC_013044	GPRC5C	chr17:72426932-72447821	30,053	71,464	-1,25	CRC < NCT	0,00175	0,0294656
XLOC_005828	CDC42BPG	chr11:64590539-64612148	6,790	16,147	-1,25	CRC < NCT	0,00185	0,0307518
XLOC_011726	COX4I1	chr16:85833172-85840607	537,517	1278,990	-1,25	CRC < NCT	0,0028	0,0405866
XLOC_004007	A1CF	chr10:52559027-52645462	2,722	6,484	-1,25	CRC < NCT	0,00195	0,0320923
XLOC_015904	UQCRFS1	chr19:29695715-29704176	105,357	250,985	-1,25	CRC < NCT	0,00025	0,0074979
XLOC_026702	BAK1	chr6:33538522-33548225	19,347	46,157	-1,25	CRC < NCT	0,00045	0,0114127
XLOC_001603	ID3	chr1:23884420-23886285	60,435	144,382	-1,26	CRC < NCT	0,00055	0,0131101
XLOC_028787	ARL4A	chr7:12726451-12732753	19,020	45,477	-1,26	CRC < NCT	0,0001	0,003647
XLOC_032031	DOLPP1	chr9:131843226-131852726	12,864	30,777	-1,26	CRC < NCT	0,00085	0,0178569
XLOC_001129	C1orf106	chr1:200860626-200884873	15,226	36,450	-1,26	CRC < NCT	0,0003	0,0084946
XLOC_032156	PLGRKT	chr9:5357966-5439392	43,601	104,385	-1,26	CRC < NCT	0,0005	0,0123209
XLOC_020479	GRAMD4	chr22:46972081-47075688	10,024	24,017	-1,26	CRC < NCT	0,00025	0,0074979
XLOC_005478	RHOG	chr11:3848193-3862215	41,719	99,978	-1,26	CRC < NCT	0,00015	0,0050841
XLOC_016020	SERTAD3	chr19:40946747-40950610	18,314	44,055	-1,27	CRC < NCT	0,0003	0,0084946
XLOC_008997	PCK2	chr14:24563339-24573383	36,124	87,082	-1,27	CRC < NCT	0,0003	0,0084946
XLOC_032453	SLC35D2	chr9:99075640-99146096	33,798	81,785	-1,27	CRC < NCT	0,00035	0,0094833
XLOC_005584	PLEKHA7	chr11:16798788-17074591	5,530	13,383	-1,27	CRC < NCT	0,00075	0,0164378
XLOC_017868	MGAT4A	chr2:99235568-99347589	11,938	28,919	-1,28	CRC < NCT	0,0009	0,0185023
XLOC_002993	-	chr1:157043249-157044472	24,442	59,221	-1,28	CRC < NCT	0,0007	0,0155855
XLOC_026524	MBOAT1	chr6:20099866-20212892	5,748	13,932	-1,28	CRC < NCT	0,00085	0,0178569
XLOC_018220	FZD5	chr2:208576194-208637145	8,275	20,125	-1,28	CRC < NCT	0,0034	0,0463025
XLOC_000250	CEP85,SH3BGRL3	chr1:26560643-26608125	288,041	701,993	-1,29	CRC < NCT	0,0008	0,0171221

XLOC_004 351	ECHS1	chr10:135175986-135186908	145,560	355,070	-1,29	CRC < NCT	0,00065	0,0147056
XLOC_017 016	MYO7B	chr2:128293253-128395512	23,261	56,797	-1,29	CRC < NCT	0,00145	0,0259223
XLOC_026 238	MARCKS	chr6:114177394-114186154	33,767	82,707	-1,29	CRC < NCT	0,0002	0,006387
XLOC_010 923	TSPAN3	chr15:77331323-77364709	118,426	290,188	-1,29	CRC < NCT	0,0019	0,0313827
XLOC_023 458	ATP8A1	chr4:42410262-42659142	6,053	14,835	-1,29	CRC < NCT	0,0008	0,0171221
XLOC_016 897	COX5B	chr2:98261912-98264657	719,250	1768,620	-1,30	CRC < NCT	0,0014	0,0253013
XLOC_033 066	RAB9A	chrX:13707221-13729067	23,751	58,555	-1,30	CRC < NCT	0,00025	0,0074979
XLOC_001 868	CYP2J2	chr1:60358971-60392469	13,799	34,052	-1,30	CRC < NCT	0,0008	0,0171221
XLOC_013 659	PRR15L	chr17:46018888-46036311	106,081	262,780	-1,31	CRC < NCT	0,00185	0,0307518
XLOC_030 448	EPHX2	chr8:27348518-27402439	22,196	54,996	-1,31	CRC < NCT	0,00095	0,0192909
XLOC_011 461	MAZ,MVP,PAGR1,PRRT2	chr16:29817356-29861063	188,548	467,508	-1,31	CRC < NCT	0,00355	0,0477391
XLOC_029 866	WASL	chr7:123319529-123391293	16,266	40,366	-1,31	CRC < NCT	0,0001	0,003647
XLOC_026 468	ECI2	chr6:4068592-4135831	30,830	76,625	-1,31	CRC < NCT	0,00085	0,0178569
XLOC_020 475	TTC38	chr22:46663839-46690990	28,723	71,413	-1,31	CRC < NCT	0,00025	0,0074979
XLOC_027 698	HLA-DPB1	chr6_cox_hap2:4476525-4501576	13,031	32,419	-1,31	CRC < NCT	0,00165	0,0284652
XLOC_012 906	PDK2	chr17:48172100-48207246	15,615	38,855	-1,32	CRC < NCT	0,001	0,0199726
XLOC_031 885	NANS	chr9:100807717-100881900	94,392	235,027	-1,32	CRC < NCT	0,00155	0,0273878
XLOC_018 345	RAB17	chr2:238482947-238504835	15,605	38,909	-1,32	CRC < NCT	0,0014	0,0253013
XLOC_020 426	ACO2	chr22:41855708-41940610	56,386	140,612	-1,32	CRC < NCT	0,00085	0,0178569
XLOC_004 236	COL17A1	chr10:105791042-105845869	41,663	104,070	-1,32	CRC < NCT	0,0017	0,0290257
XLOC_015 252	RELB	chr19:45504703-45542097	10,752	26,923	-1,32	CRC < NCT	0,001	0,0199726
XLOC_002 082	IGSF3	chr1:117115963-117210401	3,121	7,819	-1,32	CRC < NCT	0,00075	0,0164378
XLOC_014 351	NEDD4L	chr18:55711602-56068882	6,772	16,967	-1,33	CRC < NCT	0,0001	0,003647
XLOC_020 700	BAIAP2L2	chr22:38480888-38507208	21,822	54,674	-1,33	CRC < NCT	0,0004	0,01042
XLOC_004 711	ADM	chr11:10326526-10328949	25,920	65,082	-1,33	CRC < NCT	0,0008	0,0171221
XLOC_015 190	EGLN2,MIA,RAB4B	chr19:41275773-41316757	54,899	137,898	-1,33	CRC < NCT	0,0002	0,006387
XLOC_030 784	GSDMD	chr8:144519822-144645233	43,011	108,221	-1,33	CRC < NCT	0,00035	0,0094833
XLOC_020 925	IL17RE	chr3:9943697-9958084	14,846	37,481	-1,34	CRC < NCT	0,0002	0,006387
XLOC_000 254	RPS6KA1	chr1:26856248-26901523	31,573	79,813	-1,34	CRC < NCT	0,0002	0,006387
XLOC_020 938	PPARG	chr3:12329348-12476055	42,846	108,643	-1,34	CRC < NCT	5,00E-05	0,0020443
XLOC_023 307	TLR3	chr4:186989988-187009433	3,877	9,832	-1,34	CRC < NCT	0,0029	0,0416718
XLOC_011 428	LCMT1	chr16:25043061-25189554	28,321	72,009	-1,35	CRC < NCT	0,00035	0,0094833
XLOC_025 056	EDIL3	chr5:83235518-83713718	2,699	6,881	-1,35	CRC < NCT	0,0035	0,0472409
XLOC_012 173	CTRL,LCAT,PSMB10,SLC12A 4	chr16:67927174-68002597	112,330	287,305	-1,35	CRC < NCT	0,00085	0,0178569
XLOC_004 756	HTATIP2	chr11:20385230-20405550	63,899	163,760	-1,36	CRC < NCT	5,00E-05	0,0020443
XLOC_021 787	LRRFIP2	chr3:37026570-37218313	15,786	40,482	-1,36	CRC < NCT	0,0022	0,0346434
XLOC_031 886	GALNT12	chr9:101569610-101613552	22,987	58,955	-1,36	CRC < NCT	0,0001	0,003647
XLOC_029 927	PARP12	chr7:139722297-139763759	10,180	26,128	-1,36	CRC < NCT	0,00015	0,0050841

XLOC_001 790	HECTD3	chr1:45468050-45481341	16,711	42,993	-1,36	CRC < NCT	0,00165	0,0284652
XLOC_011 571	CPNE2	chr16:57126454-57181878	15,698	40,472	-1,37	CRC < NCT	0,0003	0,0084946
XLOC_017 755	PRADC1	chr2:73455131-73460658	19,723	50,967	-1,37	CRC < NCT	0,0009	0,0185023
XLOC_030 424	SORBS3	chr8:22407433-22433011	23,695	61,281	-1,37	CRC < NCT	0,0003	0,0084946
XLOC_029 402	PDGFA	chr7:536075-564869	7,221	18,704	-1,37	CRC < NCT	0,00065	0,0147056
XLOC_011 586	MMP15	chr16:58059281-58080807	17,839	46,221	-1,37	CRC < NCT	0,0006	0,0139198
XLOC_008 433	CDX2,URAD	chr13:28532462-28563098	73,949	191,780	-1,37	CRC < NCT	0,00065	0,0147056
XLOC_003 263	OPTN	chr10:13141907-13201897	25,837	67,888	-1,38	CRC < NCT	5,00E-05	0,0020443
XLOC_031 613	SLC1A1	chr9:4489692-4587475	8,569	22,269	-1,38	CRC < NCT	0,00045	0,0114127
XLOC_032 133	MRPL41	chr9:140446308-140448160	108,730	283,062	-1,38	CRC < NCT	0,00015	0,0050841
XLOC_006 984	OAS1,OAS3	chr12:113344496-113412164	77,951	203,433	-1,38	CRC < NCT	0,0013	0,0239449
XLOC_021 345	DIRC2	chr3:122513888-122599989	5,017	13,103	-1,38	CRC < NCT	0,00065	0,0147056
XLOC_012 918	ABCC3	chr17:48711987-48769063	20,464	52,515	-1,39	CRC < NCT	0,0001	0,003647
XLOC_000 565	PRKACB	chr1:84543657-84705471	15,786	41,315	-1,39	CRC < NCT	0,0005	0,0123209
XLOC_001 835	ECHDC2	chr1:53361581-53387493	14,867	38,935	-1,39	CRC < NCT	0,0002	0,006387
XLOC_020 634	TBC1D10A	chr22:30687978-30722955	8,799	23,064	-1,39	CRC < NCT	0,0009	0,0185023
XLOC_011 117	-	chr15:56365655-56366133	32,147	84,277	-1,39	CRC < NCT	0,00315	0,0437473
XLOC_006 533	H2AFJ	chr12:14926153-14930936	24,953	65,584	-1,39	CRC < NCT	0,0002	0,006387
XLOC_001 606	HMGCL	chr1:24128355-24165102	32,570	85,701	-1,40	CRC < NCT	0,0003	0,0084946
XLOC_023 341	LETM1	chr4:1813118-1858232	10,093	26,577	-1,40	CRC < NCT	5,00E-05	0,0020443
XLOC_029 113	LAMTOR4	chr7:99728304-99756354	175,076	461,914	-1,40	CRC < NCT	0,0011	0,0213843
XLOC_004 325	CLRN3	chr10:129674211-129691211	78,110	206,149	-1,40	CRC < NCT	0,0002	0,006387
XLOC_006 173	SIAE	chr11:124492741-124568400	43,681	115,372	-1,40	CRC < NCT	0,00215	0,0341212
XLOC_032 248	KIAA1161	chr9:34366090-34376971	8,657	22,888	-1,40	CRC < NCT	5,00E-05	0,0020443
XLOC_019 789	IFNAR2,IL10RB	chr21:34602199-34669717	34,532	91,547	-1,41	CRC < NCT	0,0001	0,003647
XLOC_000 311	KIAA1522	chr1:33207233-33324480	37,743	100,083	-1,41	CRC < NCT	0,0032	0,0443068
XLOC_016 285	LENG1,TMC4	chr19:54641435-54676967	40,880	108,461	-1,41	CRC < NCT	0,0009	0,0185023
XLOC_002 162	FAM63A	chr1:150954111-150980854	7,185	19,064	-1,41	CRC < NCT	0,00085	0,0178569
XLOC_010 906	COX5A,RPP25	chr15:75212616-75287738	261,686	694,363	-1,41	CRC < NCT	0,00035	0,0094833
XLOC_021 984	EIF4E3	chr3:71728272-71804328	3,909	10,380	-1,41	CRC < NCT	0,00035	0,0094833
XLOC_018 267	NHEJ1,SLC23A3	chr2:219933061-220034873	12,400	32,929	-1,41	CRC < NCT	0,0003	0,0084946
XLOC_002 159	CTSS	chr1:150701540-150738482	64,634	171,695	-1,41	CRC < NCT	0,00015	0,0050841
XLOC_025 249	PRELID2	chr5:145082976-145215103	5,895	15,699	-1,41	CRC < NCT	0,0014	0,0253013
XLOC_016 364	CHMP2A,UBE2M	chr19:59062921-59086353	169,883	452,784	-1,41	CRC < NCT	0,00015	0,0050841
XLOC_012 041	SEPHS2	chr16:30454769-30458104	51,045	136,227	-1,42	CRC < NCT	5,00E-05	0,0020443
XLOC_010 515	PDE8A	chr15:85523375-85682437	11,268	30,076	-1,42	CRC < NCT	0,00025	0,0074979
XLOC_008 437	SLC46A3	chr13:29274211-29293373	10,843	28,949	-1,42	CRC < NCT	0,00045	0,0114127
XLOC_026 883	FAM46A	chr6:82454828-82462428	8,854	23,694	-1,42	CRC < NCT	0,0006	0,0139198

XLOC_017252	STRADB	chr2:202316391-202345606	6,279	16,838	-1,42	CRC < NCT	0,0006	0,0139198
XLOC_017773	DQX1	chr2:74745240-74753465	5,477	14,723	-1,43	CRC < NCT	0,0028	0,0405866
XLOC_026040	KLC4	chr6:43005354-43042876	25,207	67,812	-1,43	CRC < NCT	0,0032	0,0443068
XLOC_005130	P2RY2	chr11:72929342-72953472	1,077	2,900	-1,43	CRC < NCT	0,0024	0,0366226
XLOC_012846	ACBD4	chr17:43209966-43221544	5,375	14,494	-1,43	CRC < NCT	0,0024	0,0366226
XLOC_015754	ACP5	chr19:11685474-11689801	26,222	70,810	-1,43	CRC < NCT	0,00025	0,0074979
XLOC_013492	PGAP3	chr17:37827373-37844323	13,921	37,782	-1,44	CRC < NCT	0,0003	0,0084946
XLOC_032631	CRAT	chr9:131857005-131873070	18,439	50,299	-1,45	CRC < NCT	5,00E-05	0,0020443
XLOC_026099	LRRC1	chr6:53610598-53789519	13,780	37,591	-1,45	CRC < NCT	5,00E-05	0,0020443
XLOC_032385	FRMD3	chr9:85834232-86153348	2,227	6,082	-1,45	CRC < NCT	0,00345	0,046739
XLOC_019020	ACSS2	chr20:33462761-33515769	44,820	122,432	-1,45	CRC < NCT	0,0003	0,0084946
XLOC_032224	MOB3B	chr9:27325073-27529850	4,369	11,979	-1,46	CRC < NCT	5,00E-05	0,0020443
XLOC_003780	PWWP2B	chr10:134210701-134231363	16,620	45,578	-1,46	CRC < NCT	0,0004	0,01042
XLOC_015647	TICAM1	chr19:4815922-4832129	10,389	28,497	-1,46	CRC < NCT	5,00E-05	0,0020443
XLOC_025984	PPARD	chr6:35310321-35395999	15,368	42,293	-1,46	CRC < NCT	0,00025	0,0074979
XLOC_013213	P2RX5,TAX1BP3	chr17:3539761-3599698	118,122	326,820	-1,47	CRC < NCT	0,0002	0,006387
XLOC_001257	CAPN2	chr1:223889294-223963786	68,757	190,270	-1,47	CRC < NCT	5,00E-05	0,0020443
XLOC_004155	PDLIM1	chr10:96997324-97050905	166,415	460,714	-1,47	CRC < NCT	0,0001	0,003647
XLOC_009253	IFI27L1	chr14:94547632-94569062	13,519	37,447	-1,47	CRC < NCT	0,00255	0,0382395
XLOC_017330	CYP27A1	chr2:219646471-219680016	17,113	47,482	-1,47	CRC < NCT	0,00015	0,0050841
XLOC_014754	EFNA2	chr19:1281807-1301429	9,280	25,749	-1,47	CRC < NCT	0,0004	0,01042
XLOC_003857	PHYH	chr10:13319795-13342130	27,074	75,244	-1,47	CRC < NCT	5,00E-05	0,0020443
XLOC_024375	F2RL1	chr5:76114408-76133281	29,154	81,035	-1,47	CRC < NCT	5,00E-05	0,0020443
XLOC_000389	TMEM125	chr1:43735603-43739679	19,059	52,996	-1,48	CRC < NCT	0,00025	0,0074979
XLOC_032269	FAM214B	chr9:35104112-35116303	9,517	26,505	-1,48	CRC < NCT	5,00E-05	0,0020443
XLOC_000040	PRKCZ	chr1:1979959-2144777	11,168	31,120	-1,48	CRC < NCT	0,00365	0,0486178
XLOC_022085	HGD	chr3:120347012-120405386	7,796	21,773	-1,48	CRC < NCT	0,0013	0,0239449
XLOC_021154	PRKCD	chr3:53190175-53226733	21,163	59,303	-1,49	CRC < NCT	5,00E-05	0,0020443
XLOC_017460	FAM110C	chr2:36329-46907	6,070	17,021	-1,49	CRC < NCT	0,0024	0,0366226
XLOC_032625	ZER1	chr9:131486738-131534748	10,365	29,080	-1,49	CRC < NCT	0,0001	0,003647
XLOC_015601	PLEKHJ1	chr19:2163833-2260525	69,013	193,917	-1,49	CRC < NCT	0,0026	0,0386713
XLOC_031775	GDA	chr9:74729510-74867200	7,879	22,160	-1,49	CRC < NCT	0,00045	0,0114127
XLOC_032506	EPB41L4B	chr9:111934107-112083501	21,262	59,975	-1,50	CRC < NCT	5,00E-05	0,0020443
XLOC_027750	HLA-B,HLA-C	chr6_cox_hap2:2749774-2837927	97,812	245,225	-1,50	CRC < NCT	0,00155	0,0273878
XLOC_000424	MAST2	chr1:46269265-46501906	13,054	36,878	-1,50	CRC < NCT	5,00E-05	0,0020443
XLOC_031023	SLC20A2	chr8:42273858-42409895	18,761	53,044	-1,50	CRC < NCT	0,0008	0,0171221
XLOC_000376	RIMKLA	chr1:42846467-42891556	1,050	2,969	-1,50	CRC < NCT	0,0007	0,0155855
XLOC_023588	HSD17B11	chr4:88256817-88312784	72,149	204,487	-1,50	CRC < NCT	5,00E-05	0,0020443

XLOC_009 270	BDKRB2	chr14:96671020-96711122	5,803	16,480	-1,51	CRC < NCT	0,0004	0,01042
XLOC_032 706	AGPAT2	chr9:139567594-139581911	118,631	337,241	-1,51	CRC < NCT	5,00E-05	0,0020443
XLOC_017 964	TFCP2L1	chr2:121974103-122042778	4,572	13,010	-1,51	CRC < NCT	0,00035	0,0094833
XLOC_002 322	ARHGAP30,TSTD1,USF1	chr1:161007411-161039790	130,815	372,590	-1,51	CRC < NCT	0,001	0,0199726
XLOC_019 847	ABCG1	chr21:43619798-43724500	9,928	28,290	-1,51	CRC < NCT	0,00015	0,0050841
XLOC_013 653	OSBPL7	chr17:45884628-45899199	5,006	14,271	-1,51	CRC < NCT	0,00015	0,0050841
XLOC_001 539	NBPF1	chr1:16854837-16996604	8,684	24,760	-1,51	CRC < NCT	0,00075	0,0164378
XLOC_032 532	HDHD3	chr9:116134439-116142479	46,817	133,513	-1,51	CRC < NCT	5,00E-05	0,0020443
XLOC_006 686	PRR13	chr12:53835375-53841906	162,105	462,592	-1,51	CRC < NCT	0,0001	0,003647
XLOC_024 844	CMBL	chr5:10274832-10308925	20,472	58,504	-1,51	CRC < NCT	0,00025	0,0074979
XLOC_020 002	RCAN1	chr21:35888739-35987441	24,023	68,717	-1,52	CRC < NCT	0,0001	0,003647
XLOC_003 261	CAMK1D	chr10:12391505-12877545	3,833	11,001	-1,52	CRC < NCT	0,00175	0,0294656
XLOC_026 090	PAQR8	chr6:52226547-52281378	17,006	48,816	-1,52	CRC < NCT	5,00E-05	0,0020443
XLOC_010 902	ULK3	chr15:75128456-75135552	10,462	30,055	-1,52	CRC < NCT	5,00E-05	0,0020443
XLOC_026 359	PPP1R14C	chr6:150464187-150571530	9,824	28,233	-1,52	CRC < NCT	0,00065	0,0147056
XLOC_015 832	NR2F6	chr19:17342634-17356714	44,531	128,045	-1,52	CRC < NCT	5,00E-05	0,0020443
XLOC_015 360	MYH14	chr19:50691218-50814033	46,589	133,974	-1,52	CRC < NCT	0,0003	0,0084946
XLOC_017 338	ZFAND2B	chr2:220071505-220074373	31,389	90,285	-1,52	CRC < NCT	5,00E-05	0,0020443
XLOC_014 263	RIOK3	chr18:21032786-21063107	19,411	55,934	-1,53	CRC < NCT	5,00E-05	0,0020443
XLOC_029 790	MOGAT3,NAT16	chr7:100812737-100844439	8,227	23,716	-1,53	CRC < NCT	0,0037	0,0490331
XLOC_009 551	EGLN3	chr14:34393420-34420284	22,910	66,296	-1,53	CRC < NCT	0,0001	0,003647
XLOC_016 291	CDC42EP5	chr19:54976208-54984437	92,479	268,062	-1,54	CRC < NCT	5,00E-05	0,0020443
XLOC_005 819	BAD	chr11:64037299-64056972	46,793	135,660	-1,54	CRC < NCT	0,0004	0,01042
XLOC_002 547	PLXNA2	chr1:208195581-208417665	3,020	8,799	-1,54	CRC < NCT	5,00E-05	0,0020443
XLOC_014 779	GNA11	chr19:3094407-3124388	23,894	69,340	-1,54	CRC < NCT	5,00E-05	0,0020443
XLOC_023 020	PARM1	chr4:75858284-75975604	33,392	97,009	-1,54	CRC < NCT	0,00025	0,0074979
XLOC_011 222	FAM195A	chr16:691547-699253	53,244	155,317	-1,54	CRC < NCT	0,0002	0,006387
XLOC_020 335	TCN2	chr22:31003069-31023047	13,734	40,147	-1,55	CRC < NCT	0,00015	0,0050841
XLOC_006 121	AMICA1	chr11:118064441-118095820	9,469	27,712	-1,55	CRC < NCT	0,00055	0,0131101
XLOC_015 634	MAP2K2	chr19:4087695-4124214	98,529	288,547	-1,55	CRC < NCT	5,00E-05	0,0020443
XLOC_014 982	OCEL1	chr19:17336787-17340029	16,609	48,670	-1,55	CRC < NCT	0,0012	0,0226522
XLOC_023 451	RBM47	chr4:40421402-40632918	24,799	72,774	-1,55	CRC < NCT	5,00E-05	0,0020443
XLOC_012 031	YPEL3	chr16:30103633-30107580	30,048	88,177	-1,55	CRC < NCT	0,00015	0,0050841
XLOC_007 344	VDR	chr12:48234192-48298849	14,981	44,102	-1,56	CRC < NCT	5,00E-05	0,0020443
XLOC_033 823	IL2RG	chrX:70327251-70331922	55,679	164,254	-1,56	CRC < NCT	5,00E-05	0,0020443
XLOC_003 792	PRAP1	chr10:135156637-135168377	197,638	583,114	-1,56	CRC < NCT	0,00305	0,0429805
XLOC_024 716	NEURL1B	chr5:172068260-172118542	8,353	24,705	-1,56	CRC < NCT	5,00E-05	0,0020443
XLOC_018 185	SATB2	chr2:200133645-200342223	9,693	28,684	-1,57	CRC < NCT	0,0012	0,0226522

XLOC_024641	SYNPO	chr5:149965445-150038935	13,046	38,682	-1,57	CRC < NCT	0,00115	0,0220507
XLOC_003550	PAPSS2	chr10:89419475-89507462	30,343	90,195	-1,57	CRC < NCT	5,00E-05	0,0020443
XLOC_019572	CTSZ	chr20:57556262-57582309	161,492	480,450	-1,57	CRC < NCT	0,00035	0,0094833
XLOC_022415	BDH1	chr3:197232149-197300194	18,402	54,764	-1,57	CRC < NCT	0,0001	0,003647
XLOC_027131	MPC1	chr6:166778400-166796502	34,692	103,456	-1,58	CRC < NCT	5,00E-05	0,0020443
XLOC_000512	PGM1	chr1:64058946-64125916	25,592	76,732	-1,58	CRC < NCT	5,00E-05	0,0020443
XLOC_010692	BMF	chr15:40380088-40401094	3,259	9,776	-1,58	CRC < NCT	0,00025	0,0074979
XLOC_032030	FAM73B	chr9:131798821-131834363	8,006	24,015	-1,58	CRC < NCT	5,00E-05	0,0020443
XLOC_001641	SLC9A1	chr1:27425299-27481888	14,529	43,659	-1,59	CRC < NCT	5,00E-05	0,0020443
XLOC_025117	TRIM36	chr5:114460276-114516243	2,138	6,426	-1,59	CRC < NCT	0,0034	0,0463025
XLOC_006417	B4GALNT3	chr12:569184-672933	9,273	27,899	-1,59	CRC < NCT	5,00E-05	0,0020443
XLOC_011874	ADCY9	chr16:4012463-4166186	2,417	7,276	-1,59	CRC < NCT	0,00035	0,0094833
XLOC_007203	KLRB1	chr12:9747869-9760497	14,808	44,628	-1,59	CRC < NCT	0,00265	0,0390961
XLOC_023244	ETFDH	chr4:159587826-159644847	13,682	41,465	-1,60	CRC < NCT	0,0038	0,049887
XLOC_002236	MUC1	chr1:155158298-155164440	72,939	221,218	-1,60	CRC < NCT	0,00065	0,0147056
XLOC_000953	SLAMF7	chr1:160708846-160725076	6,013	18,283	-1,60	CRC < NCT	0,00025	0,0074979
XLOC_029408	ZFAND2A	chr7:1187702-1214905	31,639	96,229	-1,60	CRC < NCT	0,00015	0,0050841
XLOC_001600	TCEA3	chr1:23704700-23751651	33,043	100,584	-1,61	CRC < NCT	0,0005	0,0123209
XLOC_021646	FAM43A	chr3:194406172-194410449	3,429	10,445	-1,61	CRC < NCT	0,0006	0,0139198
XLOC_025158	IRF1	chr5:131817299-131828016	23,016	70,126	-1,61	CRC < NCT	5,00E-05	0,0020443
XLOC_000470	CPT2	chr1:53662100-53686601	14,264	43,463	-1,61	CRC < NCT	0,0004	0,01042
XLOC_016019	SERTAD1	chr19:40927532-40931932	22,286	68,122	-1,61	CRC < NCT	0,0001	0,003647
XLOC_021341	FAM162A	chr3:122078351-122233831	137,290	419,805	-1,61	CRC < NCT	0,00075	0,0164378
XLOC_017916	MALL	chr2:110841446-110874143	3,758	11,517	-1,62	CRC < NCT	0,0008	0,0171221
XLOC_001632	AIM1L	chr1:26648349-26685400	2,789	8,558	-1,62	CRC < NCT	0,00015	0,0050841
XLOC_004174	AVPI1	chr10:99437174-99447570	25,255	77,625	-1,62	CRC < NCT	5,00E-05	0,0020443
XLOC_020382	SH3BP1	chr22:38035450-38054384	15,844	48,893	-1,63	CRC < NCT	5,00E-05	0,0020443
XLOC_013433	MYO1D	chr17:30819452-31233604	41,289	127,584	-1,63	CRC < NCT	5,00E-05	0,0020443
XLOC_013219	ZZEF1	chr17:3907496-4060999	5,920	18,362	-1,63	CRC < NCT	5,00E-05	0,0020443
XLOC_005164	CAPN5	chr11:76777991-76837211	28,024	86,933	-1,63	CRC < NCT	5,00E-05	0,0020443
XLOC_030401	NAT2	chr8:18248754-18258729	8,327	25,849	-1,63	CRC < NCT	0,0019	0,0313827
XLOC_011710	OSGIN1	chr16:83986800-83999940	3,981	12,370	-1,64	CRC < NCT	0,00105	0,0206545
XLOC_010434	MPI	chr15:75182047-75199462	9,218	28,676	-1,64	CRC < NCT	0,00135	0,0246666
XLOC_025370	CLTB	chr5:175818581-175843639	141,989	442,126	-1,64	CRC < NCT	5,00E-05	0,0020443
XLOC_021304	GRAMD1C	chr3:113557680-113666368	2,185	6,812	-1,64	CRC < NCT	0,0012	0,0226522
XLOC_026744	KCNK5	chr6:39151691-39197572	9,166	28,589	-1,64	CRC < NCT	5,00E-05	0,0020443
XLOC_001974	BCAR3	chr1:94027297-94315109	8,805	27,545	-1,65	CRC < NCT	0,00085	0,0178569
XLOC_003589	SLC35G1	chr10:95653696-95714457	3,631	11,364	-1,65	CRC < NCT	0,0006	0,0139198

XLOC_000 883	ZBTB7B	chr1:154975105-155006257	19,135	60,012	-1,65	CRC < NCT	5,00E-05	0,0020443
XLOC_005 911	CPT1A	chr11:68522087-68609399	19,937	62,530	-1,65	CRC < NCT	5,00E-05	0,0020443
XLOC_005 323	OAF	chr11:120081403-120102085	38,622	121,388	-1,65	CRC < NCT	5,00E-05	0,0020443
XLOC_005 252	ACAT1	chr11:107992249-108019481	51,643	162,464	-1,65	CRC < NCT	5,00E-05	0,0020443
XLOC_005 999	CCDC89,SYTL2	chr11:85394886-85522203	16,221	51,156	-1,66	CRC < NCT	5,00E-05	0,0020443
XLOC_029 716	SAMD9	chr7:92727481-92747349	4,626	14,606	-1,66	CRC < NCT	5,00E-05	0,0020443
XLOC_022 894	C4orf19	chr4:37455425-37687999	8,771	27,745	-1,66	CRC < NCT	0,0005	0,0123209
XLOC_007 952	-	chr12:53535755-53536289	11,166	35,368	-1,66	CRC < NCT	0,00275	0,0400849
XLOC_001 293	RHOU	chr1:228780393-228882417	9,123	28,985	-1,67	CRC < NCT	5,00E-05	0,0020443
XLOC_005 916	-	chr11:69290371-69294855	4,020	12,778	-1,67	CRC < NCT	0,0021	0,0336498
XLOC_026 639	HLA-B	chr6:31321648-31325028	19,258	61,260	-1,67	CRC < NCT	0,00095	0,0192909
XLOC_019 498	ACOT8	chr20:44451854-44507769	27,399	87,196	-1,67	CRC < NCT	0,0008	0,0171221
XLOC_022 365	P3H2	chr3:189674508-189862635	5,615	17,871	-1,67	CRC < NCT	0,00045	0,0114127
XLOC_001 123	NR5A2	chr1:199996713-200146723	4,235	13,546	-1,68	CRC < NCT	5,00E-05	0,0020443
XLOC_020 810	ARSA	chr22:51061181-51066601	9,355	30,147	-1,69	CRC < NCT	5,00E-05	0,0020443
XLOC_020 403	APOBEC3A,APOBEC3A_B,APOBEC3B,APOBEC3C,APOBEC3F,APOBEC3G	chr22:39348619-39483748	21,936	70,694	-1,69	CRC < NCT	5,00E-05	0,0020443
XLOC_011 720	KIAA0513	chr16:85061356-85127835	2,037	6,578	-1,69	CRC < NCT	0,0014	0,0253013
XLOC_010 578	ARRDC4	chr15:98503920-98517072	8,215	26,532	-1,69	CRC < NCT	5,00E-05	0,0020443
XLOC_013 453	CCL5	chr17:34198495-34207377	21,957	70,953	-1,69	CRC < NCT	0,00025	0,0074979
XLOC_025 126	SEMA6A	chr5:115735251-115910622	3,586	11,588	-1,69	CRC < NCT	0,001	0,0199726
XLOC_031 125	PAG1	chr8:81880042-82024344	2,201	7,115	-1,69	CRC < NCT	5,00E-05	0,0020443
XLOC_029 119	AGFG2	chr7:100136795-100165955	6,722	21,731	-1,69	CRC < NCT	5,00E-05	0,0020443
XLOC_033 152	MAOA	chrX:43514154-43606071	25,631	83,136	-1,70	CRC < NCT	5,00E-05	0,0020443
XLOC_003 695	CASP7	chr10:115438920-115491429	36,705	119,079	-1,70	CRC < NCT	5,00E-05	0,0020443
XLOC_022 006	STX19	chr3:93698237-93774522	13,008	42,225	-1,70	CRC < NCT	0,00235	0,0361928
XLOC_020 997	GPD1L	chr3:32148002-32210272	14,167	46,037	-1,70	CRC < NCT	5,00E-05	0,0020443
XLOC_004 298	FGFR2	chr10:123237843-123357972	5,703	18,555	-1,70	CRC < NCT	0,0003	0,0084946
XLOC_012 618	SPECC1	chr17:19912648-20223063	6,094	19,830	-1,70	CRC < NCT	5,00E-05	0,0020443
XLOC_021 487	P2RY1	chr3:152552527-152559405	1,408	4,585	-1,70	CRC < NCT	0,0009	0,0185023
XLOC_004 065	C10orf54	chr10:73156690-73575729	12,724	41,486	-1,71	CRC < NCT	5,00E-05	0,0020443
XLOC_001 575	CAMK2N1	chr1:20808883-20812728	50,313	164,059	-1,71	CRC < NCT	5,00E-05	0,0020443
XLOC_011 776	SPIRE2	chr16:89894906-89937855	11,510	37,586	-1,71	CRC < NCT	5,00E-05	0,0020443
XLOC_000 887	EFNA1	chr1:155100348-155107386	58,633	191,976	-1,71	CRC < NCT	5,00E-05	0,0020443
XLOC_021 449	ATP1B3	chr3:141595469-141645382	95,598	313,833	-1,71	CRC < NCT	5,00E-05	0,0020443
XLOC_012 223	CFDP1	chr16:75327607-75467387	67,297	221,282	-1,72	CRC < NCT	5,00E-05	0,0020443
XLOC_004 632	SLC22A18	chr11:2907517-2946653	37,568	123,615	-1,72	CRC < NCT	0,0001	0,003647
XLOC_009 526	RIPK3	chr14:24805226-24809242	12,521	41,222	-1,72	CRC < NCT	5,00E-05	0,0020443

XLOC_017 800	RETSAT	chr2:85567685-85581821	41,376	136,253	-1,72	CRC < NCT	5,00E-05	0,0020443
XLOC_024 858	FAM134B	chr5:16471694-16630078	12,695	41,833	-1,72	CRC < NCT	0,0001	0,003647
XLOC_021 867	SLC25A20	chr3:48894355-48936426	16,272	54,153	-1,73	CRC < NCT	5,00E-05	0,0020443
XLOC_005 313	C2CD2L	chr11:118977678-118989250	5,496	18,306	-1,74	CRC < NCT	5,00E-05	0,0020443
XLOC_022 339	LIPH	chr3:185223903-185270574	14,999	50,128	-1,74	CRC < NCT	5,00E-05	0,0020443
XLOC_013 261	YBX2	chr17:7185025-7197930	5,854	19,661	-1,75	CRC < NCT	0,0034	0,0463025
XLOC_012 206	ZNF821	chr16:71893579-71919302	3,018	10,138	-1,75	CRC < NCT	0,00115	0,0220507
XLOC_020 587	CHCHD10	chr22:24105207-24110199	149,542	503,024	-1,75	CRC < NCT	5,00E-05	0,0020443
XLOC_021 760	THRB	chr3:24156882-24541502	0,877	2,954	-1,75	CRC < NCT	0,00315	0,0437473
XLOC_006 575	PPFIBP1,REP15	chr12:27677044-27855806	20,164	68,060	-1,76	CRC < NCT	0,00115	0,0220507
XLOC_010 764	SPPL2A	chr15:50994460-51058999	20,897	70,606	-1,76	CRC < NCT	5,00E-05	0,0020443
XLOC_014 561	CCDC68	chr18:52568131-52626739	5,387	18,219	-1,76	CRC < NCT	5,00E-05	0,0020443
XLOC_022 131	MGLL	chr3:127407776-127542233	39,134	132,481	-1,76	CRC < NCT	5,00E-05	0,0020443
XLOC_002 709	CHML,OPN3	chr1:241695433-241803701	9,655	32,744	-1,76	CRC < NCT	5,00E-05	0,0020443
XLOC_019 102	CTSA,ZSWIM1	chr20:44509847-44541003	111,252	378,398	-1,77	CRC < NCT	0,0007	0,0155855
XLOC_000 025	CPTP	chr1:1260142-1264318	13,908	47,478	-1,77	CRC < NCT	5,00E-05	0,0020443
XLOC_024 443	C5orf30	chr5:102594441-102614404	9,228	31,525	-1,77	CRC < NCT	5,00E-05	0,0020443
XLOC_031 677	PRSS3	chr9:33750463-33799236	135,414	462,719	-1,77	CRC < NCT	5,00E-05	0,0020443
XLOC_017 386	B3GNT7	chr2:232260334-232265877	18,851	64,426	-1,77	CRC < NCT	0,0003	0,0084946
XLOC_005 387	TMEM45B	chr11:129685707-129765493	58,474	200,697	-1,78	CRC < NCT	0,0003	0,0084946
XLOC_019 010	ZNF341	chr20:32319565-32398905	2,021	6,941	-1,78	CRC < NCT	0,0009	0,0185023
XLOC_007 677	GLTP	chr12:110288745-110318293	16,706	57,410	-1,78	CRC < NCT	5,00E-05	0,0020443
XLOC_010 526	ISG20	chr15:89178867-89199646	18,037	62,143	-1,78	CRC < NCT	0,0001	0,003647
XLOC_015 798	GIPC1	chr19:14588540-14607252	95,341	329,597	-1,79	CRC < NCT	5,00E-05	0,0020443
XLOC_021 959	FAM3D	chr3:58619661-58693628	246,846	855,124	-1,79	CRC < NCT	5,00E-05	0,0020443
XLOC_026 304	TCF21	chr6:133562494-134216937	4,739	16,440	-1,79	CRC < NCT	0,00275	0,0400849
XLOC_015 344	FCGRT,RCN3	chr19:50015535-50046890	110,391	386,113	-1,81	CRC < NCT	5,00E-05	0,0020443
XLOC_032 743	PNPLA7	chr9:140354010-140445083	1,379	4,827	-1,81	CRC < NCT	0,00215	0,0341212
XLOC_024 498	GRAMD3	chr5:125695787-125829987	8,959	31,411	-1,81	CRC < NCT	5,00E-05	0,0020443
XLOC_024 528	SOWAHA	chr5:132149015-132152489	3,430	12,030	-1,81	CRC < NCT	0,00035	0,0094833
XLOC_003 680	MXI1	chr10:111966934-112050062	14,757	51,795	-1,81	CRC < NCT	5,00E-05	0,0020443
XLOC_023 798	DDX60,DDX60L	chr4:169137408-169401665	15,174	53,385	-1,81	CRC < NCT	0,0001	0,003647
XLOC_013 630	PLCD3	chr17:43189005-43209900	29,338	103,432	-1,82	CRC < NCT	5,00E-05	0,0020443
XLOC_020 360	ISX	chr22:35461926-35489158	8,233	29,033	-1,82	CRC < NCT	0,00045	0,0114127
XLOC_020 693	PDXP	chr22:38054600-38062939	9,786	34,578	-1,82	CRC < NCT	0,00055	0,0131101
XLOC_025 818	HIST1H1E,HIST1H2BD	chr6:26156509-26203032	25,477	90,038	-1,82	CRC < NCT	0,00225	0,0351876
XLOC_007 217	STYK1	chr12:10766264-10826891	4,854	17,189	-1,82	CRC < NCT	0,0001	0,003647
XLOC_026 103	TINAG	chr6:54172563-54254950	7,315	25,975	-1,83	CRC < NCT	0,0005	0,0123209

XLOC_004800	PRR5L	chr11:36317724-36503998	8,706	31,229	-1,84	CRC < NCT	5,00E-05	0,0020443
XLOC_021816	ANO10	chr3:43396014-43663560	24,529	88,341	-1,85	CRC < NCT	5,00E-05	0,0020443
XLOC_007543	PTPRR	chr12:71031846-71314584	8,519	30,812	-1,85	CRC < NCT	0,0004	0,01042
XLOC_012032	GDPD3,MAPK3	chr16:30114753-30134630	88,274	320,247	-1,86	CRC < NCT	5,00E-05	0,0020443
XLOC_018301	PID1	chr2:229888650-230136057	7,347	26,709	-1,86	CRC < NCT	5,00E-05	0,0020443
XLOC_006655	METTL7A	chr12:51316449-51326844	14,579	53,008	-1,86	CRC < NCT	0,0001	0,003647
XLOC_015147	C19orf33	chr19:38793886-38808502	339,404	1235,550	-1,86	CRC < NCT	0,0004	0,01042
XLOC_019252	FKBP1A,SDCBP2	chr20:1290554-1374081	195,671	712,389	-1,86	CRC < NCT	5,00E-05	0,0020443
XLOC_013185	MIR22,MIR22HG	chr17:1614796-1641893	12,780	46,532	-1,86	CRC < NCT	0,00315	0,0437473
XLOC_016827	VAMP8	chr2:85804613-85809156	176,122	641,417	-1,86	CRC < NCT	5,00E-05	0,0020443
XLOC_003686	ADRA2A	chr10:112836653-112840733	5,568	20,320	-1,87	CRC < NCT	0,00065	0,0147056
XLOC_010824	DAPK2	chr15:64199232-64338552	6,488	23,716	-1,87	CRC < NCT	5,00E-05	0,0020443
XLOC_029681	FGL2	chr7:76589113-76924684	8,477	30,997	-1,87	CRC < NCT	0,00075	0,0164378
XLOC_030724	FER1L6	chr8:124864226-125183763	1,170	4,278	-1,87	CRC < NCT	0,0001	0,003647
XLOC_001730	EPHA10	chr1:38179259-38230824	2,453	8,990	-1,87	CRC < NCT	5,00E-05	0,0020443
XLOC_000820	CGN	chr1:151483861-151511170	18,414	67,626	-1,88	CRC < NCT	5,00E-05	0,0020443
XLOC_003531	CDHR1	chr10:85954322-85985345	2,544	9,358	-1,88	CRC < NCT	0,0015	0,0267114
XLOC_010343	GCNT3	chr15:59848172-59982273	95,605	352,509	-1,88	CRC < NCT	0,0018	0,0301682
XLOC_030838	DEFB1	chr8:6728096-6735529	42,211	155,842	-1,88	CRC < NCT	0,00175	0,0294656
XLOC_012226	CHST5,TMEM231	chr16:75531846-75590933	9,366	34,615	-1,89	CRC < NCT	5,00E-05	0,0020443
XLOC_015333	TRPM4	chr19:49661015-49715163	25,174	93,153	-1,89	CRC < NCT	5,00E-05	0,0020443
XLOC_023345	ZFYVE28	chr4:2269461-2420429	3,394	12,566	-1,89	CRC < NCT	0,0002	0,006387
XLOC_012640	LGALS9	chr17:25958173-25976586	49,792	184,654	-1,89	CRC < NCT	5,00E-05	0,0020443
XLOC_029784	ACHE	chr7:100487414-100493754	4,432	16,467	-1,89	CRC < NCT	0,00065	0,0147056
XLOC_024648	SLC36A1	chr5:150816552-150876994	2,692	10,009	-1,89	CRC < NCT	5,00E-05	0,0020443
XLOC_028849	PRR15	chr7:29019582-29609367	48,251	179,948	-1,90	CRC < NCT	0,0012	0,0226522
XLOC_005220	ENDOD1	chr11:94822973-94870460	10,048	37,481	-1,90	CRC < NCT	5,00E-05	0,0020443
XLOC_010246	CHP1	chr15:41523436-41574090	46,157	172,420	-1,90	CRC < NCT	5,00E-05	0,0020443
XLOC_013159	METRNL	chr17:81037563-81057934	28,163	105,216	-1,90	CRC < NCT	5,00E-05	0,0020443
XLOC_015848	LOC729966	chr19:18360759-18366404	19,493	73,223	-1,91	CRC < NCT	0,00115	0,0220507
XLOC_005386	BARX2	chr11:129169582-129322317	5,933	22,311	-1,91	CRC < NCT	0,0012	0,0226522
XLOC_000876	IL6R	chr1:154375918-154520624	3,049	11,525	-1,92	CRC < NCT	0,0006	0,0139198
XLOC_011918	LITAF	chr16:11640030-11681322	60,393	228,835	-1,92	CRC < NCT	5,00E-05	0,0020443
XLOC_006090	IL18	chr11:112013913-112035147	29,672	112,496	-1,92	CRC < NCT	5,00E-05	0,0020443
XLOC_015668	SLC25A23	chr19:6440074-6459809	19,492	73,947	-1,92	CRC < NCT	5,00E-05	0,0020443
XLOC_020342	INPP5J	chr22:31518560-31536593	3,605	13,678	-1,92	CRC < NCT	0,001	0,0199726
XLOC_007481	MYO1A,TAC3	chr12:57403780-57444985	21,710	82,419	-1,92	CRC < NCT	0,0005	0,0123209
XLOC_003590	PLCE1	chr10:95753695-96122735	3,192	12,148	-1,93	CRC < NCT	0,0002	0,006387

XLOC_021016	VILL	chr3:38029429-38178733	25,610	97,565	-1,93	CRC < NCT	0,0038	0,049887
XLOC_007247	LINC01559	chr12:13523301-13540128	17,664	67,571	-1,94	CRC < NCT	5,00E-05	0,0020443
XLOC_014883	ATG4D	chr19:10654411-10676727	11,072	42,437	-1,94	CRC < NCT	0,0001	0,003647
XLOC_026073	MEP1A	chr6:46761066-46817343	67,569	259,316	-1,94	CRC < NCT	0,00045	0,0114127
XLOC_022166	C3orf36,SLCO2A1	chr3:133646649-133757888	7,413	28,449	-1,94	CRC < NCT	0,0001	0,003647
XLOC_000194	CDA	chr1:20915417-20945406	28,367	109,163	-1,94	CRC < NCT	5,00E-05	0,0020443
XLOC_020737	PMM1	chr22:41972889-41990936	23,039	88,764	-1,95	CRC < NCT	5,00E-05	0,0020443
XLOC_030812	GPT,MFSD3	chr8:145728352-145743225	29,274	112,920	-1,95	CRC < NCT	0,0001	0,003647
XLOC_032731	TPRN	chr9:140086006-140095809	22,632	87,422	-1,95	CRC < NCT	5,00E-05	0,0020443
XLOC_013182	RILP,SCARF1	chr17:1536940-1553540	10,947	42,381	-1,95	CRC < NCT	5,00E-05	0,0020443
XLOC_000290	SERINC2	chr1:31882411-31907660	93,710	364,058	-1,96	CRC < NCT	5,00E-05	0,0020443
XLOC_017776	HK2	chr2:75059668-75123333	29,815	116,107	-1,96	CRC < NCT	0,00125	0,0233782
XLOC_002050	RHOC	chr1:113216933-113250025	264,422	1030,610	-1,96	CRC < NCT	5,00E-05	0,0020443
XLOC_016918	IL1R2	chr2:102608273-102658359	11,607	45,635	-1,98	CRC < NCT	5,00E-05	0,0020443
XLOC_000633	CDC14A	chr1:100817819-100986123	1,459	5,741	-1,98	CRC < NCT	0,00025	0,0074979
XLOC_011661	MARVELD3	chr16:71659875-71676061	15,006	59,063	-1,98	CRC < NCT	5,00E-05	0,0020443
XLOC_000314	AZIN2	chr1:33546713-33586157	1,814	7,140	-1,98	CRC < NCT	0,0015	0,0267114
XLOC_024619	SPINK5	chr5:147443534-147518996	2,585	10,189	-1,98	CRC < NCT	0,00055	0,0131101
XLOC_026732	CPNE5	chr6:36708546-36807382	2,246	8,867	-1,98	CRC < NCT	0,0014	0,0253013
XLOC_006453	PLEKHG6	chr12:6419601-6451895	17,793	70,231	-1,98	CRC < NCT	0,0022	0,0346434
XLOC_015638	STAP2	chr19:4324037-4400565	61,768	244,504	-1,98	CRC < NCT	0,00165	0,0284652
XLOC_016596	MAPRE3	chr2:27193238-27250087	6,474	25,651	-1,99	CRC < NCT	5,00E-05	0,0020443
XLOC_021278	FLJ22763	chr3:108844991-108870972	3,576	14,207	-1,99	CRC < NCT	0,0006	0,0139198
XLOC_006663	ACVRL1	chr12:52300716-52317210	12,882	51,426	-2,00	CRC < NCT	5,00E-05	0,0020443
XLOC_012764	RAPGEFL1	chr17:38333262-38352120	14,359	57,371	-2,00	CRC < NCT	5,00E-05	0,0020443
XLOC_032611	AK1,ST6GALNAC4,ST6GALNAC6	chr9:130625997-130679336	63,953	256,108	-2,00	CRC < NCT	5,00E-05	0,0020443
XLOC_007023	P2RX4	chr12:121647663-121672051	21,499	86,169	-2,00	CRC < NCT	5,00E-05	0,0020443
XLOC_013048	SLC9A3R1	chr17:72744750-72765499	60,961	245,396	-2,01	CRC < NCT	5,00E-05	0,0020443
XLOC_013184	TLCD2	chr17:1606083-1613917	2,898	11,679	-2,01	CRC < NCT	5,00E-05	0,0020443
XLOC_020275	IGLL5	chr22:23229959-23238293	247,882	998,938	-2,01	CRC < NCT	5,00E-05	0,0020443
XLOC_003221	-	chr10:3848936-3856828	0,628	2,535	-2,01	CRC < NCT	0,00365	0,0486178
XLOC_014961	CYP4F12,CYP4F3,CYP4F8	chr19:15674468-15810097	11,954	48,542	-2,02	CRC < NCT	5,00E-05	0,0020443
XLOC_015004	JUND,MIR3188	chr19:18367905-18394826	44,550	181,271	-2,02	CRC < NCT	0,00245	0,0372298
XLOC_007756	RHOF	chr12:122150649-122231841	24,250	98,858	-2,03	CRC < NCT	5,00E-05	0,0020443
XLOC_001795	CCDC17	chr1:46085651-46089843	0,285	1,165	-2,03	CRC < NCT	0,0017	0,0290257
XLOC_013487	CACNB1	chr17:37329708-37353956	1,189	4,852	-2,03	CRC < NCT	0,00105	0,0206545
XLOC_020961	PLCL2	chr3:16926451-17132137	5,715	23,331	-2,03	CRC < NCT	0,0001	0,003647
XLOC_001774	C1orf210	chr1:43747556-43751288	17,295	70,839	-2,03	CRC < NCT	5,00E-05	0,0020443

XLOC_018905	BMP2	chr20:6748338-6764278	6,823	27,961	-2,03	CRC < NCT	0,0001	0,003647
XLOC_021811	HIGD1A	chr3:42823936-42846027	51,224	210,403	-2,04	CRC < NCT	5,00E-05	0,0020443
XLOC_024922	CCL28	chr5:43376747-43412512	14,552	60,463	-2,05	CRC < NCT	5,00E-05	0,0020443
XLOC_011294	IL32	chr16:3115285-3131887	139,749	581,321	-2,06	CRC < NCT	5,00E-05	0,0020443
XLOC_007748	OASL	chr12:121455564-121477045	13,341	56,334	-2,08	CRC < NCT	5,00E-05	0,0020443
XLOC_008710	-	chr13:23269998-23270841	123,264	521,155	-2,08	CRC < NCT	5,00E-05	0,0020443
XLOC_003684	PDCD4	chr10:112628646-112679133	35,602	150,544	-2,08	CRC < NCT	5,00E-05	0,0020443
XLOC_013308	LINC00675	chr17:10575675-10721948	19,353	82,240	-2,09	CRC < NCT	0,00145	0,0259223
XLOC_008559	PCDH20	chr13:61982714-61989655	0,710	3,023	-2,09	CRC < NCT	0,00205	0,0331105
XLOC_009271	BDKRB1	chr14:96722450-96734946	2,444	10,410	-2,09	CRC < NCT	0,00275	0,0400849
XLOC_013210	TRPV3	chr17:3413649-3461289	0,417	1,779	-2,09	CRC < NCT	0,00335	0,0457925
XLOC_028924	UPP1	chr7:48127707-48148330	16,634	70,968	-2,09	CRC < NCT	5,00E-05	0,0020443
XLOC_013866	ACOX1	chr17:73937456-74002162	12,520	52,207	-2,09	CRC < NCT	5,00E-05	0,0020443
XLOC_024960	MIER3	chr5:56204944-56255288	6,052	25,943	-2,10	CRC < NCT	5,00E-05	0,0020443
XLOC_007646	APPL2	chr12:105567073-105630008	18,756	80,452	-2,10	CRC < NCT	5,00E-05	0,0020443
XLOC_007311	CPNE8	chr12:39046001-39303638	1,794	7,713	-2,10	CRC < NCT	0,0003	0,0084946
XLOC_014800	SHD	chr19:4278597-4290721	8,554	36,839	-2,11	CRC < NCT	0,0006	0,0139198
XLOC_001032	PRDX6	chr1:173152869-173457953	144,326	622,071	-2,11	CRC < NCT	5,00E-05	0,0020443
XLOC_014468	ABHD3	chr18:19230855-19284766	18,568	80,076	-2,11	CRC < NCT	5,00E-05	0,0020443
XLOC_014730	MISP	chr19:748420-764604	112,065	487,467	-2,12	CRC < NCT	5,00E-05	0,0020443
XLOC_008175	SMIM2-AS1	chr13:44684533-44814657	4,170	18,243	-2,13	CRC < NCT	0,00195	0,0320923
XLOC_007111	PXMP2	chr12:133264191-133287344	26,573	116,490	-2,13	CRC < NCT	5,00E-05	0,0020443
XLOC_016967	SLC20A1	chr2:113399195-113422302	23,394	99,517	-2,14	CRC < NCT	5,00E-05	0,0020443
XLOC_023057	AGPAT9	chr4:84456964-84527057	7,111	31,400	-2,14	CRC < NCT	5,00E-05	0,0020443
XLOC_026292	ENPP1	chr6:132129155-132216301	1,347	5,953	-2,14	CRC < NCT	5,00E-05	0,0020443
XLOC_023678	FABP2	chr4:120238404-120243316	11,389	50,455	-2,15	CRC < NCT	0,0004	0,01042
XLOC_000259	SFN	chr1:27188857-27191094	138,060	611,870	-2,15	CRC < NCT	5,00E-05	0,0020443
XLOC_010250	ITPKA	chr15:41785499-41808017	8,861	39,413	-2,15	CRC < NCT	0,0006	0,0139198
XLOC_014548	ACAA2	chr18:47309873-47721526	63,396	301,837	-2,16	CRC < NCT	0,0001	0,003647
XLOC_002354	GPA33	chr1:167011909-167060130	86,201	385,380	-2,16	CRC < NCT	5,00E-05	0,0020443
XLOC_001607	FUCA1	chr1:24171571-24194859	45,110	202,283	-2,16	CRC < NCT	5,00E-05	0,0020443
XLOC_016890	CNNM4	chr2:97426638-97480055	12,713	57,288	-2,17	CRC < NCT	5,00E-05	0,0020443
XLOC_025322	CCNJL	chr5:159678541-159766599	2,789	12,576	-2,17	CRC < NCT	5,00E-05	0,0020443
XLOC_024295	GZMA	chr5:54398473-54406080	15,139	68,418	-2,18	CRC < NCT	5,00E-05	0,0020443
XLOC_020365	HMOX1	chr22:35777059-35790207	30,851	139,428	-2,18	CRC < NCT	5,00E-05	0,0020443
XLOC_019547	BCAS1	chr20:52560062-52687304	18,878	85,853	-2,19	CRC < NCT	5,00E-05	0,0020443
XLOC_005374	ST3GAL4	chr11:126225539-126284536	30,392	138,595	-2,19	CRC < NCT	5,00E-05	0,0020443
XLOC_012055	PRSS8	chr16:31125950-31147151	132,236	603,664	-2,19	CRC < NCT	5,00E-05	0,0020443

XLOC_024522	SLC22A4,SLC22A5	chr5:131628194-131731308	8,444	38,604	-2,19	CRC < NCT	5,00E-05	0,0020443
XLOC_022293	TNFSF10	chr3:172220032-172241385	39,592	181,623	-2,20	CRC < NCT	5,00E-05	0,0020443
XLOC_016764	MXD1	chr2:70142172-70170220	13,997	64,589	-2,21	CRC < NCT	5,00E-05	0,0020443
XLOC_010294	SQRDL	chr15:45923345-45995383	95,998	442,987	-2,21	CRC < NCT	5,00E-05	0,0020443
XLOC_026534	TDP2	chr6:24650147-24721261	65,613	303,027	-2,21	CRC < NCT	0,0001	0,003647
XLOC_029234	AHCYL2	chr7:128864854-129070167	23,112	107,475	-2,22	CRC < NCT	5,00E-05	0,0020443
XLOC_002040	ADORA3,TMIGD3	chr1:112025874-112106625	2,478	11,540	-2,22	CRC < NCT	0,00105	0,0206545
XLOC_009203	TMEM63C	chr14:77648101-77725838	0,723	3,371	-2,22	CRC < NCT	0,001	0,0199726
XLOC_010393	PAQR5	chr15:69591233-69740766	2,992	14,035	-2,23	CRC < NCT	0,00285	0,0411153
XLOC_004984	RARRES3	chr11:63304272-63313930	43,385	203,738	-2,23	CRC < NCT	5,00E-05	0,0020443
XLOC_021785	TRANK1	chr3:36861826-36986548	3,054	14,343	-2,23	CRC < NCT	5,00E-05	0,0020443
XLOC_000153	FBLIM1,TMEM82	chr1:16062549-16113085	27,285	128,778	-2,24	CRC < NCT	5,00E-05	0,0020443
XLOC_020039	TMPRSS2	chr21:42834709-42880085	57,027	269,789	-2,24	CRC < NCT	5,00E-05	0,0020443
XLOC_033134	TSPAN7	chrX:38420730-38548174	19,114	90,608	-2,25	CRC < NCT	5,00E-05	0,0020443
XLOC_015636	SIRT6	chr19:4174103-4182597	19,885	82,106	-2,25	CRC < NCT	5,00E-05	0,0020443
XLOC_016731	UGP2	chr2:64068097-64246595	92,129	437,902	-2,25	CRC < NCT	5,00E-05	0,0020443
XLOC_008632	SLC15A1	chr13:99335985-99404929	1,086	5,166	-2,25	CRC < NCT	0,00135	0,0246666
XLOC_031519	-	chr8:82433964-82434350	133,363	634,857	-2,25	CRC < NCT	5,00E-05	0,0020443
XLOC_020201	PEX26	chr22:18555305-18583259	10,400	50,383	-2,28	CRC < NCT	5,00E-05	0,0020443
XLOC_009254	IFI27	chr14:94572229-94583040	655,882	3201,700	-2,29	CRC < NCT	5,00E-05	0,0020443
XLOC_025462	-	chr5:17353813-17354498	58,902	288,064	-2,29	CRC < NCT	5,00E-05	0,0020443
XLOC_004676	SMPD1	chr11:6411643-6462697	14,575	71,779	-2,30	CRC < NCT	5,00E-05	0,0020443
XLOC_005886	POLD4	chr11:67083881-67169376	62,282	306,860	-2,30	CRC < NCT	0,00045	0,0114127
XLOC_029932	DENND2A	chr7:140216480-140340388	2,463	12,158	-2,30	CRC < NCT	5,00E-05	0,0020443
XLOC_032498	KLF4	chr9:110247132-110252047	48,108	238,279	-2,31	CRC < NCT	5,00E-05	0,0020443
XLOC_007179	CD163L1	chr12:7506163-7596977	2,729	13,610	-2,32	CRC < NCT	5,00E-05	0,0020443
XLOC_017597	XDH	chr2:31556972-31638224	4,970	24,802	-2,32	CRC < NCT	0,0001	0,003647
XLOC_004722	MICALCL	chr11:12297546-12383898	1,158	5,818	-2,33	CRC < NCT	5,00E-05	0,0020443
XLOC_034329	-	chrX:147133674-147134430	131,372	661,829	-2,33	CRC < NCT	5,00E-05	0,0020443
XLOC_011559	MT1A,MT1E,MT1F,MT1H,MT1M,MT2A	chr16:56642339-56705163	490,937	2477,580	-2,34	CRC < NCT	0,0017	0,0290257
XLOC_030615	STMN2	chr8:80523048-80578411	1,759	8,880	-2,34	CRC < NCT	0,0023	0,035694
XLOC_015205	CEACAM3	chr19:42300320-42315689	0,934	4,721	-2,34	CRC < NCT	0,0026	0,0386713
XLOC_020368	RASD2	chr22:35937351-35950052	2,974	15,082	-2,34	CRC < NCT	5,00E-05	0,0020443
XLOC_007018	ACADS	chr12:121163451-121177813	33,101	168,653	-2,35	CRC < NCT	5,00E-05	0,0020443
XLOC_001853	TTC22	chr1:55245379-55310140	7,440	38,147	-2,36	CRC < NCT	5,00E-05	0,0020443
XLOC_007602	TMCC3	chr12:94954008-95211930	4,200	21,724	-2,37	CRC < NCT	5,00E-05	0,0020443
XLOC_013225	GGT6	chr17:4459207-4464215	23,068	119,971	-2,38	CRC < NCT	5,00E-05	0,0020443
XLOC_007953	-	chr12:53536471-53536847	3,286	17,139	-2,38	CRC < NCT	0,002	0,03265

XLOC_010296	SEMA6D	chr15:47476402-48066602	2,063	10,797	-2,39	CRC < NCT	5,00E-05	0,0020443
XLOC_010996	MESP1	chr15:90290475-90294540	2,895	15,177	-2,39	CRC < NCT	0,00085	0,0178569
XLOC_023748	NR3C2	chr4:148999672-149496084	4,531	23,956	-2,40	CRC < NCT	5,00E-05	0,0020443
XLOC_029361	AOC1	chr7:150549564-150558379	83,289	441,248	-2,41	CRC < NCT	5,00E-05	0,0020443
XLOC_012998	LOC100507002	chr17:63096697-63113267	2,882	15,272	-2,41	CRC < NCT	0,0024	0,0366226
XLOC_011438	APOBR	chr16:28474388-28518155	11,608	61,529	-2,41	CRC < NCT	0,0001	0,003647
XLOC_001692	TMEM54	chr1:33352097-33366953	246,387	1307,070	-2,41	CRC < NCT	5,00E-05	0,0020443
XLOC_009192	FLVCR2	chr14:76035585-76117107	2,061	10,947	-2,41	CRC < NCT	5,00E-05	0,0020443
XLOC_013451	MMP28	chr17:34083248-34122725	8,297	44,090	-2,41	CRC < NCT	5,00E-05	0,0020443
XLOC_020667	MB	chr22:36002810-36031040	5,649	30,021	-2,41	CRC < NCT	0,0007	0,0155855
XLOC_000941	LINC01133	chr1:159930858-159957532	22,193	118,123	-2,41	CRC < NCT	5,00E-05	0,0020443
XLOC_016304	PTPRH	chr19:55692598-55720874	14,798	79,074	-2,42	CRC < NCT	5,00E-05	0,0020443
XLOC_033910	GLRA4	chrX:102957970-102983552	0,537	2,874	-2,42	CRC < NCT	0,0033	0,045347
XLOC_001246	C1orf115	chr1:220863627-220872499	12,444	66,744	-2,42	CRC < NCT	5,00E-05	0,0020443
XLOC_021040	VIPR1	chr3:42530790-42579666	10,988	59,038	-2,43	CRC < NCT	5,00E-05	0,0020443
XLOC_005422	CDHR5,IRF7	chr11:612554-640705	54,704	294,449	-2,43	CRC < NCT	0,0002	0,006387
XLOC_016512	RSAD2	chr2:6968684-7039891	2,025	10,905	-2,43	CRC < NCT	0,00295	0,0419277
XLOC_011604	CES2	chr16:66965956-66979730	47,552	256,427	-2,43	CRC < NCT	5,00E-05	0,0020443
XLOC_011605	CES3	chr16:66994988-67009313	9,828	53,002	-2,43	CRC < NCT	5,00E-05	0,0020443
XLOC_016068	ETHE1,PHLDB3	chr19:43978426-44031825	187,304	1015,600	-2,44	CRC < NCT	5,00E-05	0,0020443
XLOC_011266	MIR940	chr16:2318575-2323595	0,541	2,936	-2,44	CRC < NCT	0,00045	0,0114127
XLOC_001415	FAM132A	chr1:1177825-1183427	4,133	22,564	-2,45	CRC < NCT	0,00165	0,0284652
XLOC_011343	TNFRSF17	chr16:12058963-12061925	5,459	29,915	-2,45	CRC < NCT	0,0004	0,01042
XLOC_009721	ENTPD5	chr14:74318388-74551216	11,188	61,578	-2,46	CRC < NCT	0,00055	0,0131101
XLOC_015935	-	chr19:35914477-35925512	0,166	0,923	-2,48	CRC < NCT	0,00275	0,0400849
XLOC_000068	ESPN	chr1:6484847-6580121	5,791	32,401	-2,48	CRC < NCT	0,00045	0,0114127
XLOC_021598	LOC344887	chr3:185677633-185703042	0,382	2,154	-2,50	CRC < NCT	0,0023	0,035694
XLOC_010819	CA12	chr15:63605508-63674762	31,232	176,284	-2,50	CRC < NCT	5,00E-05	0,0020443
XLOC_025758	GCNT2	chr6:10521567-10629632	1,318	7,462	-2,50	CRC < NCT	0,0026	0,0386713
XLOC_024745	CDHR2	chr5:175953699-176037131	13,743	78,065	-2,51	CRC < NCT	0,0002	0,006387
XLOC_008971	-	chr14:22918564-22935569	1,859	10,645	-2,52	CRC < NCT	0,00055	0,0131101
XLOC_026498	ADTRP	chr6:11711517-11803262	24,896	142,892	-2,52	CRC < NCT	5,00E-05	0,0020443
XLOC_030632	ATP6V0D2	chr8:87111110-87166464	0,572	3,292	-2,52	CRC < NCT	0,0013	0,0239449
XLOC_021518	ARL14	chr3:160394776-160398761	16,719	96,348	-2,53	CRC < NCT	5,00E-05	0,0020443
XLOC_028562	HLA-A	chr6_ssto_hap7:1239291-1243012	12,525	72,711	-2,54	CRC < NCT	0,0008	0,0171221
XLOC_002306	IGSF9	chr1:159896742-159915791	6,159	35,777	-2,54	CRC < NCT	5,00E-05	0,0020443
XLOC_026551	HIST1H1C	chr6:26044483-26056854	52,394	304,814	-2,54	CRC < NCT	5,00E-05	0,0020443
XLOC_023541	NAAA	chr4:76831807-76862252	21,507	125,791	-2,55	CRC < NCT	5,00E-05	0,0020443

XLOC_005155	MOGAT2	chr11:75412617-75446941	4,418	25,843	-2,55	CRC < NCT	0,0004	0,01042
XLOC_022329	CLCN2	chr3:184053716-184086516	7,210	42,557	-2,56	CRC < NCT	0,0007	0,0155855
XLOC_016366	-	chr19:291719-296164	0,113	0,672	-2,57	CRC < NCT	0,0031	0,0433833
XLOC_028786	SCIN	chr7:12609038-12699802	5,938	35,831	-2,59	CRC < NCT	5,00E-05	0,0020443
XLOC_017132	DHRS9	chr2:169923544-169952678	49,230	300,455	-2,61	CRC < NCT	5,00E-05	0,0020443
XLOC_029704	SRI	chr7:87563476-87856352	281,592	1725,890	-2,62	CRC < NCT	5,00E-05	0,0020443
XLOC_027026	SGK1	chr6:134490383-134639196	31,026	192,109	-2,63	CRC < NCT	5,00E-05	0,0020443
XLOC_019069	SGK2	chr20:42187576-42216951	11,618	72,776	-2,65	CRC < NCT	5,00E-05	0,0020443
XLOC_012202	PHLPP2	chr16:71678602-71759685	2,235	14,018	-2,65	CRC < NCT	5,00E-05	0,0020443
XLOC_020682	TST	chr22:37406866-37425863	146,837	924,088	-2,65	CRC < NCT	5,00E-05	0,0020443
XLOC_013065	MYO15B,SMIM5	chr17:73583591-73704142	10,136	64,342	-2,67	CRC < NCT	5,00E-05	0,0020443
XLOC_004338	-	chr10:134266775-134272610	0,250	1,611	-2,68	CRC < NCT	0,00305	0,0429805
XLOC_017406	UGT1A1,UGT1A10,UGT1A3,UGT1A4,UGT1A5,UGT1A6,UGT1A7,UGT1A8,UGT1A9	chr2:234512410-234681951	29,712	191,995	-2,69	CRC < NCT	5,00E-05	0,0020443
XLOC_026797	CLIC5	chr6:45866123-46048085	9,385	61,445	-2,71	CRC < NCT	5,00E-05	0,0020443
XLOC_029701	ABCB1,ABCB4	chr7:87031137-87461623	5,996	39,329	-2,71	CRC < NCT	5,00E-05	0,0020443
XLOC_022411	PIGZ	chr3:196673157-196702293	7,520	49,525	-2,72	CRC < NCT	5,00E-05	0,0020443
XLOC_026266	SMPDL3A	chr6:123108303-123130868	14,308	94,439	-2,72	CRC < NCT	5,00E-05	0,0020443
XLOC_015225	ZNF575	chr19:44033447-44044038	1,406	9,324	-2,73	CRC < NCT	0,00035	0,0094833
XLOC_016033	B3GNT8	chr19:41903599-41934635	9,357	62,880	-2,75	CRC < NCT	0,0004	0,01042
XLOC_006104	NXPE4	chr11:114389414-114522285	22,689	152,639	-2,75	CRC < NCT	0,00165	0,0284652
XLOC_018915	LOC101929395	chr20:10727477-10735362	3,843	26,081	-2,76	CRC < NCT	0,0031	0,0433833
XLOC_014582	RNF152	chr18:59475294-59561575	0,907	6,160	-2,76	CRC < NCT	5,00E-05	0,0020443
XLOC_019019	TP53INP2	chr20:33291914-33301245	13,659	93,532	-2,78	CRC < NCT	5,00E-05	0,0020443
XLOC_005836	NAALADL1	chr11:64808366-64826017	2,739	18,819	-2,78	CRC < NCT	0,00175	0,0294656
XLOC_032221	LRRC19	chr9:26903194-27066091	5,933	41,575	-2,81	CRC < NCT	0,00125	0,0233782
XLOC_007794	CRAT8	chr12:128507189-128511738	3,544	24,868	-2,81	CRC < NCT	0,00215	0,0341212
XLOC_032212	CDKN2B	chr9:21994789-22122216	5,313	37,473	-2,82	CRC < NCT	5,00E-05	0,0020443
XLOC_001549	PADI2	chr1:17393035-17445948	5,708	40,475	-2,83	CRC < NCT	0,00015	0,0050841
XLOC_011616	HSD11B2	chr16:67461562-67515171	55,368	393,134	-2,83	CRC < NCT	0,00055	0,0131101
XLOC_009243	CHGA	chr14:93389023-93401645	21,920	155,794	-2,83	CRC < NCT	5,00E-05	0,0020443
XLOC_024358	TMEM171	chr5:72407211-72430445	15,689	112,125	-2,84	CRC < NCT	5,00E-05	0,0020443
XLOC_030436	ADAMDEC1	chr8:24151541-24406131	14,991	107,694	-2,84	CRC < NCT	5,00E-05	0,0020443
XLOC_015866	TM6SF2	chr19:19374870-19384151	1,618	11,654	-2,85	CRC < NCT	0,00135	0,0246666
XLOC_003812	LINC00704	chr10:4648043-4720262	0,371	2,715	-2,87	CRC < NCT	0,0037	0,0490331
XLOC_005592	TPH1	chr11:18038890-18067349	0,331	2,421	-2,87	CRC < NCT	0,0017	0,0290257
XLOC_004020	SLC16A9	chr10:61410501-61469649	2,628	19,321	-2,88	CRC < NCT	5,00E-05	0,0020443
XLOC_032740	ENTPD8	chr9:140317836-140336300	11,847	87,523	-2,89	CRC < NCT	0,0004	0,01042

XLOC_029 255	AKR1B10,AKR1B15	chr7:134212314-134264592	16,766	125,109	-2,90	CRC < NCT	5,00E-05	0,0020443
XLOC_003 530	C10orf99	chr10:85933383-85945050	96,905	737,688	-2,93	CRC < NCT	5,00E-05	0,0020443
XLOC_006 175	VSIG2	chr11:124617369-124622109	40,620	311,716	-2,94	CRC < NCT	5,00E-05	0,0020443
XLOC_021 955	DNASE1L3	chr3:58178321-58200115	7,779	60,557	-2,96	CRC < NCT	5,00E-05	0,0020443
XLOC_012 720	DHRS11	chr17:34948225-34957234	23,550	183,565	-2,96	CRC < NCT	5,00E-05	0,0020443
XLOC_011 703	HSD17B2	chr16:82068623-82132141	41,950	331,114	-2,98	CRC < NCT	5,00E-05	0,0020443
XLOC_001 938	MCOLN2	chr1:85391204-85463890	2,248	17,918	-2,99	CRC < NCT	5,00E-05	0,0020443
XLOC_012 894	B4GALNT2	chr17:47209821-47256017	3,539	28,243	-3,00	CRC < NCT	0,0013	0,0239449
XLOC_012 495	WSCD1	chr17:5973873-6027911	1,262	10,100	-3,00	CRC < NCT	0,00015	0,0050841
XLOC_026 962	-	chr6:110851862-110887817	0,467	3,741	-3,00	CRC < NCT	0,0006	0,0139198
XLOC_003 441	MYPN	chr10:69865873-69971773	0,111	0,891	-3,01	CRC < NCT	0,00025	0,0074979
XLOC_033 288	GDPD2	chrX:69642880-69653553	2,399	19,463	-3,02	CRC < NCT	0,0002	0,006387
XLOC_023 571	PLAC8	chr4:84005880-84036150	71,217	581,748	-3,03	CRC < NCT	5,00E-05	0,0020443
XLOC_023 004	SLC4A4	chr4:72052460-72438207	3,932	32,269	-3,04	CRC < NCT	5,00E-05	0,0020443
XLOC_023 816	HPGD	chr4:175410208-175462407	16,339	134,572	-3,04	CRC < NCT	5,00E-05	0,0020443
XLOC_024 877	SLC45A2	chr5:33943658-33984968	0,077	0,637	-3,04	CRC < NCT	0,0016	0,0279196
XLOC_017 593	CAPN13	chr2:30945637-31030321	1,596	13,243	-3,05	CRC < NCT	0,0001	0,003647
XLOC_012 220	LDHD	chr16:75032914-75150695	9,361	77,716	-3,05	CRC < NCT	0,00035	0,0094833
XLOC_015 481	KIR2DL4,KIR3DL2	chr19:55315003-55378692	0,060	0,502	-3,06	CRC < NCT	0,00145	0,0259223
XLOC_013 517	KRT12	chr17:39017201-39024905	0,226	1,910	-3,08	CRC < NCT	0,00035	0,0094833
XLOC_024 785	BTNL3,BTNL8	chr5:180326064-180434228	17,670	149,543	-3,08	CRC < NCT	5,00E-05	0,0020443
XLOC_005 062	C11orf86	chr11:66742630-66744483	12,530	107,039	-3,09	CRC < NCT	5,00E-05	0,0020443
XLOC_027 149	FRMD1	chr6:168453753-168502179	4,025	34,551	-3,10	CRC < NCT	5,00E-05	0,0020443
XLOC_002 095	HMGCS2	chr1:120290618-120311774	71,352	628,998	-3,14	CRC < NCT	5,00E-05	0,0020443
XLOC_023 559	PRKG2	chr4:82007433-82139110	0,478	4,239	-3,15	CRC < NCT	5,00E-05	0,0020443
XLOC_019 853	PDE9A	chr21:44073817-44195624	13,934	124,588	-3,16	CRC < NCT	5,00E-05	0,0020443
XLOC_021 047	ACKR2	chr3:42846160-42910802	0,273	2,478	-3,18	CRC < NCT	0,0001	0,003647
XLOC_016 994	TMEM37	chr2:120187526-120282028	18,226	166,230	-3,19	CRC < NCT	5,00E-05	0,0020443
XLOC_021 275	HHLA2	chr3:108014321-108248169	17,340	158,261	-3,19	CRC < NCT	5,00E-05	0,0020443
XLOC_017 213	C2orf88	chr2:191002485-191184771	4,707	42,985	-3,19	CRC < NCT	0,00025	0,0074979
XLOC_005 317	PDZD3	chr11:119056141-119076916	3,491	31,949	-3,19	CRC < NCT	5,00E-05	0,0020443
XLOC_012 912	ACSF2	chr17:48503518-48553433	11,776	108,082	-3,20	CRC < NCT	5,00E-05	0,0020443
XLOC_001 130	-	chr1:200889607-200935959	0,540	5,018	-3,22	CRC < NCT	5,00E-05	0,0020443
XLOC_019 950	CLDN8	chr21:31586323-31588469	3,852	35,775	-3,22	CRC < NCT	0,0007	0,0155855
XLOC_023 048	BMP3	chr4:81951749-81979417	0,841	7,840	-3,22	CRC < NCT	0,00035	0,0094833
XLOC_017 134	-	chr2:169958246-169963721	0,438	4,097	-3,23	CRC < NCT	0,00305	0,0429805
XLOC_021 656	SLC51A	chr3:195943043-196015331	13,468	126,663	-3,23	CRC < NCT	5,00E-05	0,0020443
XLOC_020 041	-	chr21:42924030-42935777	0,786	7,399	-3,23	CRC < NCT	0,0007	0,0155855

XLOC_025805	SCGN	chr6:25652428-25702008	1,214	11,623	-3,26	CRC < NCT	0,00055	0,0131101
XLOC_019159	PCK1	chr20:56135307-56146417	27,240	260,923	-3,26	CRC < NCT	5,00E-05	0,0020443
XLOC_014289	MEP1B	chr18:29769986-29802585	1,961	18,882	-3,27	CRC < NCT	0,00215	0,0341212
XLOC_019174	EDN3	chr20:57875469-57901049	4,537	43,791	-3,27	CRC < NCT	5,00E-05	0,0020443
XLOC_011422	CHP2	chr16:23765947-23791318	23,451	232,440	-3,31	CRC < NCT	0,00015	0,0050841
XLOC_013951	CD7,SECTM1	chr17:80271360-80293275	21,448	213,071	-3,31	CRC < NCT	5,00E-05	0,0020443
XLOC_014921	BEST2	chr19:12861469-12869274	2,599	25,918	-3,32	CRC < NCT	0,0007	0,0155855
XLOC_012009	SULT1A1,SULT1A2	chr16:28564867-28634915	25,429	257,191	-3,34	CRC < NCT	5,00E-05	0,0020443
XLOC_016825	SH2D6	chr2:85655769-85664152	0,636	6,869	-3,43	CRC < NCT	0,002	0,03265
XLOC_002520	LRRN2	chr1:204586301-204654597	1,378	14,981	-3,44	CRC < NCT	5,00E-05	0,0020443
XLOC_025808	SLC17A4	chr6:25753540-25832287	2,849	31,741	-3,48	CRC < NCT	5,00E-05	0,0020443
XLOC_010998	ANPEP	chr15:90328050-90358075	132,923	1486,220	-3,48	CRC < NCT	0,00345	0,046739
XLOC_012649	SLC13A2	chr17:26800332-26824800	1,650	19,149	-3,54	CRC < NCT	5,00E-05	0,0020443
XLOC_009420	ASPG	chr14:104546675-104581918	1,663	19,333	-3,54	CRC < NCT	0,0017	0,0290257
XLOC_010371	SLC51B	chr15:65337119-65360650	22,061	259,714	-3,56	CRC < NCT	0,0008	0,0171221
XLOC_003279	TMEM236	chr10:17794211-18200192	1,598	18,852	-3,56	CRC < NCT	0,002	0,03265
XLOC_000168	PADI2	chr1:17393035-17445948	10,846	136,435	-3,65	CRC < NCT	0,00055	0,0131101
XLOC_026264	PKIB	chr6:122793061-123047518	13,850	175,431	-3,66	CRC < NCT	5,00E-05	0,0020443
XLOC_015363	SPIB	chr19:50922194-50934309	2,007	25,529	-3,67	CRC < NCT	5,00E-05	0,0020443
XLOC_031701	HRCT1	chr9:35905275-35907827	5,938	76,262	-3,68	CRC < NCT	5,00E-05	0,0020443
XLOC_030351	CLDN23	chr8:8316017-8637584	10,876	140,792	-3,69	CRC < NCT	5,00E-05	0,0020443
XLOC_001890	INSL5	chr1:67263423-67266942	6,361	84,645	-3,73	CRC < NCT	0,00025	0,0074979
XLOC_022355	SST	chr3:187386693-187388201	2,687	35,887	-3,74	CRC < NCT	0,00035	0,0094833
XLOC_000226	LOC284632	chr1:24523685-24547792	0,078	1,058	-3,76	CRC < NCT	0,00345	0,046739
XLOC_007110	P2RX2	chr12:133195365-133198972	0,025	0,343	-3,77	CRC < NCT	5,00E-05	0,0020443
XLOC_014441	LAMA1	chr18:6941742-7117813	4,092	56,753	-3,79	CRC < NCT	5,00E-05	0,0020443
XLOC_033818	TEX11	chrX:69744363-70128567	1,598	23,487	-3,88	CRC < NCT	0,00215	0,0341212
XLOC_011419	SCNN1B	chr16:23313590-23392620	5,401	79,995	-3,89	CRC < NCT	5,00E-05	0,0020443
XLOC_024635	SLC26A2	chr5:149340299-149367040	19,866	300,034	-3,92	CRC < NCT	5,00E-05	0,0020443
XLOC_022937	CWH43	chr4:48988264-49064095	1,147	18,036	-3,97	CRC < NCT	5,00E-05	0,0020443
XLOC_002747	LYPD8	chr1:248898171-248907165	142,374	2314,010	-4,02	CRC < NCT	0,0009	0,0185023
XLOC_031776	-	chr9:74868084-74875166	0,185	3,030	-4,03	CRC < NCT	0,0003	0,0084946
XLOC_018085	ABCB11	chr2:169770712-169887833	0,098	1,629	-4,06	CRC < NCT	0,00035	0,0094833
XLOC_025267	PDE6A	chr5:149237303-149324681	0,236	4,097	-4,12	CRC < NCT	0,00345	0,046739
XLOC_012967	CA4	chr17:58225176-58245480	65,670	1148,350	-4,13	CRC < NCT	5,00E-05	0,0020443
XLOC_012601	LGALS9C	chr17:18380055-18398259	2,246	39,664	-4,14	CRC < NCT	5,00E-05	0,0020443
XLOC_005753	-	chr11:59025629-59039458	0,053	0,946	-4,17	CRC < NCT	0,0036	0,0481629
XLOC_003373	TMEM72	chr10:45306471-45455137	0,499	9,131	-4,19	CRC < NCT	0,00285	0,0411153

XLOC_013420	TMIGD1	chr17:28638007-28661105	9,489	181,241	-4,26	CRC < NCT	5,00E-05	0,0020443
XLOC_006863	-	chr12:90353865-90537187	0,302	5,790	-4,26	CRC < NCT	0,00015	0,0050841
XLOC_032369	TRPM6	chr9:77337401-77503010	1,868	39,694	-4,41	CRC < NCT	5,00E-05	0,0020443
XLOC_005810	HRASLS2	chr11:63320238-63332522	3,408	76,509	-4,49	CRC < NCT	5,00E-05	0,0020443
XLOC_004933	MS4A12	chr11:60260065-60281276	45,502	1040,390	-4,52	CRC < NCT	5,00E-05	0,0020443
XLOC_020976	-	chr3:23688102-23711786	0,080	1,894	-4,57	CRC < NCT	5,00E-05	0,0020443
XLOC_005582	LOC102724957	chr11:15665430-15790137	1,346	32,281	-4,58	CRC < NCT	0,00085	0,0178569
XLOC_000375	GUCA2B	chr1:42618863-42622649	51,235	1351,410	-4,72	CRC < NCT	0,0003	0,0084946
XLOC_024181	SLC6A19	chr5:1201695-1225242	0,367	10,217	-4,80	CRC < NCT	0,0001	0,003647
XLOC_015229	-	chr19:44191723-44204678	0,230	6,604	-4,84	CRC < NCT	5,00E-05	0,0020443
XLOC_023591	ABCG2	chr4:89008292-89152474	1,334	38,468	-4,85	CRC < NCT	5,00E-05	0,0020443
XLOC_014696	-	chr18:60108511-60110981	0,016	0,488	-4,89	CRC < NCT	0,0038	0,049887
XLOC_014365	-	chr18:60076883-60078706	0,069	2,074	-4,92	CRC < NCT	0,0016	0,0279196
XLOC_011602	CA7	chr16:66875156-66907511	4,674	145,288	-4,96	CRC < NCT	0,00045	0,0114127
XLOC_015223	CD177	chr19:43857774-43874750	16,815	662,578	-5,30	CRC < NCT	5,00E-05	0,0020443
XLOC_013050	OTOP2	chr17:72920146-72930054	2,564	102,070	-5,32	CRC < NCT	0,0002	0,006387
XLOC_002278	-	chr1:157462222-157467905	0,030	1,424	-5,57	CRC < NCT	0,0008	0,0171221
XLOC_024060	-	chr4:149555490-149558069	0,043	3,475	-6,35	CRC < NCT	5,00E-05	0,0020443

Table S3

The enriched Gene Ontology (GO) categories of differentially expressed genes in colorectal carcinoma.

ID	Title	CRC>NCT logP	CRC<NCT logP	CRC>NCT GeneSet	CRC<NCT GeneSet
**GO: Biological Process**		**pValues**		**Gene List**	
GO:0051276	chromosome organization	10,0000		ANP32E ASCC3 ATAD2 ATRX AXIN2 BCOR BIRC5 BRCA1 BUB1 CBX2CBX4 CCNB1 CCT2 CCT6A CDC45 CDC6 CDCA8 CDK1 CDK2 CDKN2ACENPE CENPF CENPI CEP55 CHAF1B CHEK1 DKC1 DNMT1 DSCC1 GINS1HELLS HJURP HLTF HMGA1 HMGA2 HUWE1 IL1B INCENP JADE3 KAT2AKIF14 KIF18A KIF18B KIF2C KIF4A LIF MAD2L1 MAPK15 MCM2 MCM3MCM4 MCM6 MSH2 NAP1L1 NBN NCAPG NCAPG2 NCAPH NEK2 NPM1NUF2 NUP107 NUP155 PARP1 PHF19 PRC1 PRKDC RACGAP1 RCBTB1RCC2 RFC3 RRS1 RUVBL1 SMARCAD1 SMARCC1 SMC2 SMC4 SOX9 STILTCF3 TOP1MT TOP2A TP53 TTK UBE2C UHRF1 VENTX WDHD1 WDR75YEATS2 ZWINT	
GO:0019637	organophosphate metabolic process		10,0000		ABHD3 ACHE ADCY9 ADM ADRA2A ADSL AGPAT2 ASPG ATP5D ATP5IBAD CDA CHCHD10 CHGA COX4I1 COX5B COX6A1 COX6B1 COX7BCOX8A CRYL1 CWH43 DOLPP1 ECE1 ENPP1 ENTPD5 ENTPD8 FGFR2GPD1L GUCA2B HK2 IDH3A IMPA2 INPP5J IP6K2 ITPKA KLF4 LDHDLGALS13 LIPC **LPCAT1** MBOAT1 MBOAT2 MPC1 MPI MTM1 NDUFA6NDUFB7 P2RY1 PAPSS2 PCK1 PCK2 PDE6A PDE8A PDE9A PDGFA PDXPPDZD3 PGAP3 PGM1 PID1 PIGZ PLCD3 PLCE1 PPARD PRKCD PTGER4PTK2B RARRES3 SERINC2 SIRT6 SLC25A23 SLC26A2 SMPD1 SMPDL3ASUCLG1 UGP2 UPP1 UQCR10 UQCRC1 UQCRFS1 XDH
GO:0044255	cellular lipid metabolic process		10,0000		ABCG1 ABHD3 ACAA2 ACADS ACADVL ACHE ACOT8 ACOX1 ACSF2ACSS2 AGPAT2 APOBR ARSA ASPG B3GNT5 B4GALNT2 CES1 CPT1ACPT2 CPTP CRAT CRYL1 CWH43 CYP2J2 DECR1 DHRS3 DHRS9 DOLPP1ECHDC2 ECHS1 ECI1 ECI2 EPHX2 ETFA ETFB ETFDH FABP2 FGFR2 GGT6GLTP GPD1L HADH HMGCS2 HPGD IMPA2 INPP5J IP6K2 ISX KLF4LGALS13 LIPC **LPCAT1** MBOAT1 MBOAT2 MGLL MOGAT2 MTM1 NANSPCK1 PDGFA PDK2 PGAP3 PHYH PIGZ PLCE1 PPARD PPARG PRKCDPTK2B RARRES3 RETSAT RXRA SERINC2 SESN2 SLC27A2 SMPD1SMPDL3A ST3GAL4 THRB
GO:0000280	nuclear division	10,0000		ANAPC1 ANLN ATRX BIRC5 BMP4 BMP7 BRSK2 BUB1 CCNB1 CDC25ACDC25B CDC6 CDCA2 CDCA8 CDK1 CDK2 CENPE CENPF CEP55 CHEK1CKS2 DSCC1 HAUS6 HELLS HMGA2 IL1A IL1B INCENP KIF11 KIF14KIF18A KIF18B KIF2C KIF4A LIF MAD2L1 MET MKI67 MSH2 MSX2 MYBL2NCAPG NCAPG2 NCAPH NEK2 NOLC1 NUF2 PBK PRC1 RACGAP1 RANRCC2 RRS1 RUVBL1 SMC2 SMC4 STIL TIMELESS TOP2A TPX2 TTK UBE2CXRCC2 ZWINT	
GO:1903047	mitotic cell cycle process	10,0000		AJUBA ANAPC1 ANLN ARID3A ATRX BIRC5 BMP4 BMP7 BRSK2 BUB1CCNB1 CCND1 CDC25A CDC25B CDC45 CDC6 CDCA2 CDCA8 CDK1CDK2 CDK4 CDK6 CDKN2A CENPE CENPF CEP55 CHEK1 CKAP2 CKS2CLSPN DSCC1 E2F7 FOXM1 GINS1 GPSM2 GTSE1 HAUS6 HELLS HMGA2IL1A IL1B INCENP INHBA IQGAP3 KIF11 KIF14 KIF18A KIF18B KIF2CKIF4A LMNB1 MAD2L1 MCM10 MCM2 MCM3 MCM4 MCM6 MELK METMSH2 MYBL2 NBN NCAPG NCAPG2 NCAPH NEK2 NOLC1 NPM1 NUF2NUP107 NUP155 NUP205 NUP210 NUP58 PBK PHLDA1 PLK2 PLK4PPAT PRC1 PRKDC RACGAP1 RAN RCC2 RRM2 RRS1 RUVBL1 SKP2 SMC2SMC4 SOX4 STIL STMN1 TCF3 TIMELESS TOP2A TP53 TPD52L1 TPX2 TTKUBE2C WDR43 WDR62 ZWINT	
GO:0006629	lipid metabolic process		10,0000		ABCB11 ABCG1 ABHD3 ACAA2 ACADS ACADVL ACHE ACOT8 ACOX1ACSF2 ACSS2 ADM ADRA2A AGPAT2 APLP2 APOBR APOL2 ARSA ASPGB3GNT5 B4GALNT2 BMP2 CEBPA CES1 CPT1A CPT2 CPTP CRAT CRYL1CWH43 CYP27A1 CYP27B1 CYP2J2 DECR1 DHRS3 DHRS9 DOLPP1ECHDC2 ECHS1 ECI1 ECI2 EPHX2 ETFA ETFB ETFDH FABP2 FGFR2GDPD2 GGT6 GLTP GPD1L H2AFY HADH HINT2 HMGCS2 HPGDHRASLS2 HSD11B2 HSD17B11 HSD17B2 IMPA2 INPP5J IP6K2 ISX KLF4LGALS13 LIPC **LPCAT1** MBOAT1 MBOAT2 MGLL MGST3 MOGAT2 MTM1NAAA NANS NR5A2 PCK1 PDGFA PDK2 PGAP3 PHYH PIGZ PLCD3 PLCE1 PLCL2 PNPLA7 PPARD PPARG PRDX6 PRKCD PTK2B RARRES3 RETSATRXRA SERINC2 SESN2 SLC27A2 SMPD1 SMPDL3A ST3GAL4 TFCP2L1THRB TM6SF2
GO:0055114	oxidation-reduction process		10,0000		ACAA2 ACADS ACADVL ACO2 ACOT8 ACOX1 ACSS2 ADSL AOC1 BDH1BMP2 COX4I1 COX5B COX6A1 COX6B1 COX7B COX8A CPT1A CPT2CRAT CRYL1 CYP27A1 CYP27B1 CYP2J2 DECR1 DHRS11 DHRS3 DHRS9DYRK2 ECHS1 ECI1 ECI2 EGLN3 ENPP1 ETFA ETFB ETFDH GPD1L HADHHGD HIGD1A HIGD2A HK2 HMOX1 HPGD HSD11B2 HSD17B11HSD17B2 HTATIP2 IDH3A LDHD MAOA MGST3 NDUFA6 NDUFB7OSGIN1 P3H2 PCK2 PGM1 PHYH PID1 PLOD2 PPARD PPARG PRDX6RETSAT SDHA SDHB SELENOS SESN2 SLC25A23 SLC27A2 SQOR SUCLG1SUCLG2 TPH1 UGP2 UQCR10 UQCRC1 UQCRFS1 XDH
GO:0022402	cell cycle process	10,0000		AJUBA ANAPC1 ANLN ARID3A ATRX AXIN2 BIRC5 BMP4 BMP7 BRCA1BRSK2 BUB1 CCNB1 CCND1 CDC25A CDC25B CDC45 CDC6 CDCA2CDCA8 CDK1 CDK2 CDK4 CDK6 CDKN2A CENPE CENPF CENPI CEP55CHEK1 CKAP2 CKS2 CLSPN DACH1 DDIAS DSCC1 E2F7 ECT2 FOXM1GINS1 GPSM2 GTSE1 HAUS6 HELLS HMGA2 IL1A IL1B INCENP INHBAIQGAP3 KIF11 KIF14 KIF18A KIF18B KIF2C KIF4A LIF LMNB1 MAD2L1MAP3K20 MCM10 MCM2 MCM3 MCM4 MCM6 MELK MET MKI67 MSH2MSX2 MYBL2 NBN NCAPG NCAPG2 NCAPH NEK2 NOLC1 NPM1 NUF2NUP107 NUP155 NUP205 NUP210 NUP58 PBK PHGDH PHLDA1 PKP4PLK2 PLK4 PNPT1 PPAT PRC1 PRKDC PROX1 RACGAP1 RAN RCC2 RPRMRRM2 RRS1 RUVBL1 SKP2 SMARCAD1 SMC2 SMC4 SOX4 SOX9 STILSTMN1 TCF3 TFAP4 TIMELESS TOP2A TP53 TPD52L1 TPX2 TTK UBE2CWDR43 WDR62 XRCC2 ZWINT	
GO:0000278	mitotic cell cycle	10,0000		AJUBA ANAPC1 ANLN ARID3A ATRX BIRC5 BMP4 BMP7 BRSK2 BUB1CCNB1 CCND1 CDC25A CDC25B CDC45 CDC6 CDCA2 CDCA8 CDK1CDK2 CDK4 CDK6 CDKN2A CENPE CENPF CEP55 CHEK1 CKAP2 CKS2CLSPN DSCC1 E2F7 FOXM1 FZD3 GINS1 GPSM2 GTSE1 HAUS6 HELLSHMGA2 IL1A IL1B INCENP INHBA IQGAP3 KIF11 KIF14 KIF18A KIF18BKIF2C KIF4A LMNB1 MAD2L1 MCIDAS MCM10 MCM2 MCM3 MCM4MCM6 MELK MET MSH2 MYBL2 NBN NCAPG NCAPG2 NCAPH NEK2NOLC1 NPM1 NUF2 NUP107 NUP155 NUP205 NUP210 NUP58 PBKPHLDA1 PLK2 PLK4 PPAT PRC1 PRKDC RACGAP1 RAN RCC2 RPRMRRM2 RRS1 RUVBL1 SKP2 SMC2 SMC4 SOX4 STIL STMN1 TCF3TIMELESS TOP2A TP53 TPD52L1 TPX2 TTK UBE2C WDR43 WDR62XRCC2 ZWINT	

Table S4

The Enriched pathways of all deregulated genes in colorectal carcinoma.

ID	Title	Alternative splicing genes in CRC_logP	Mutated genes in CRC_logP	Overexpressed genes in CRC_logP	Downregulated genes in CRC_logP	Alternative splicing genes in CRC_GeneSet	Mutated genes in CRC_GeneSet	Overexpressed genes in CRC_GeneSet	Downregulated genes in CRC_GeneSet
Pathway		pValues				Gene List			
83054	Cell cycle	4,9273	2,7484	10		CCND1 CDC25B CDKN2B MAD2L1 MCM2 MCM3 MDM2 SFN	ANAPC5 CREBBP MAD2L1 MCM7 ORC4 PRKDC TP53TTK	ANAPC1 BUB1 CCNB1 CCND1 CDC25A CDC25B CDC45 CDC6 CDK1 CDK6 CDKN2A CHEK1 MAD2L1 MCM2 MCM3 MCM4 MCM6 PRKDC SKP2 TP53 TTK	
82935	Fatty acid degradation	4,4303			5,9342	ACAA2 ACADS ACAT1 ACOX1 CPT1A			ACAA2 ACADS ACADVL ACAT1 ACOX1 CPT1A CPT2ECHS1 ECI1 ECI2 HADHA
167325	Protein processing in endoplasmic reticulum		10				CANX DDIT3 DDOST DERL1 EIF2AK2 ERO1A GANABHSP90AA1 HSP90AB1 HSPH1 HYOU1 NSFL1C P4HBPDIA4 RRBP1 SEC24D VCP		
125136	Spliceosome		10				DDX42 DDX5 HNRNPK HNRNPU PLRG1 PRPF19 PRPF3 PRPF40A RBMX SF3B2 SF3B4 SNRNP200 SNRPB SNU13 SNW1 SRSF6 THOC2 U2SURP		
132956	Metabolic pathways				10				ACAA2 ACADS ACADVL ACAT1 ACLY ACO2 ACOT8ACOX1 ACP5 ACSS2 ADSL AGPAT2 AHCY AHCYL2 ANPEP AOC1 ATP5F1D ATP5ME ATP6V0D2 AZIN2B3GNT5 B4GALNT3 B4GALT1 BDH1 CDA CES1 CHPFCMAS CMBL COX4I1 COX5B COX6A1 COX6B1 COX7BCOX8A CRYL1 CYP27A1 CYP27B1 CYP2J2 DHCR7DHRS3 DHRS9 ECHS1 ENPP1 EPHX2 GALM GALNT12 GCNT2 GCNT3 GGT6 GMPS GOT1 HADHA HGD HK2 HMGCL HMGCS2 HMOX1 HSD17B2 IDH3A IMPA2INPP5J ITPKA LIPC LPCAT1 MAOA MBOAT1 MBOAT2 MGAT4A MGLL MPI MTM1 NANS NAT2 NDUFA6NDUFB7 PAPSS2 PCK1 PCK2 PDXP PGM1 PLCD3PLCE1 PMM1 PRDX6 PRPS2 RIMKLA SDHA SDHBSEPHS2
83055	p53 signaling pathway			10				CCNB1 CCND1 CD82 CDK1 CDK6 CDKN2A CHEK1GTSE1 PMAIP1 RPRM RRM2 SERPINB5 TP53	
82927	Citrate cycle (TCA cycle)		3,7496		5,6769		ACO2 DLST IDH1 IDH2 SUCLA2		ACLY ACO2 IDH3A PCK1 PCK2 SDHA SDHB SUCLG1 SUCLG2
868084	Fatty acid metabolism	4,2445			4,6866	ACAA2 ACADS ACAT1 ACOX1 CPT1A			ACAA2 ACADS ACADVL ACAT1 ACOX1 CPT1A CPT2ECHS1 HACD3 HADHA
814926	Carbon metabolism		2,9831		3,6172		ACO2 DLST ESD GLUD1 GOT2 IDH1 IDH2 SUCLA2		ACADS ACAT1 ACO2 ACSS2 ECHS1 GOT1 HADHA HK2IDH3A PRPS2 SDHA SDHB SUCLG1 SUCLG2
82952	Valine, leucine and isoleucine degradation	3,1096			2,4831	ACAA2 ACADS ACAT1 HMGCS2			ACAA2 ACADS ACAT1 ECHS1 HADHA HMGCL HMGCS2
177876	RNA transport		4,8919				EIF2S2 EIF3D EIF3E EIF4G2 EIF5B FXR1 NUP88 NUP98PABPC1 PABPC4 POM121 THOC2 XPO1		
413350	Serine biosynthesis, glycerate-3P => serine			4,706				PHGDH PSAT1 PSPH	
790012	Biosynthesis of amino acids			4,5265				AADAT CPS1 ENO2 MAT1A PHGDH PRPS1 PSAT1PSPH PYCR1 SHMT2	
82942	Oxidative phosphorylation				4,494				ATP1D ATPI ATP6V0D2 COX4I1 COX5B COX6A1 COX6B1 COX7A2 COX7B COX8A NDUFA6 NDUFB7 SDHA SDHB UQCR10 UQCRC1 UQCRFS1
83042	PPAR signaling pathway				4,4701				ACOX1 CPT1A CPT2 CYP27A1 FABP2 HMGCS2 PCK1 PCK2 PPARD PPARG RXRA SLC27A2
658418	Viral carcinogenesis		4,1543				ACTN1 CASP8 CHD4 CREBBP DDB1 EIF2AK2 HNRNPKKRAS NFKB2 SCRIB SND1 SNW1 TP53		
83007	Butanoate metabolism				3,9677				ACADS ACAT1 BDH1 ECHS1 HADHA HMGCL HMGCS2
83061	Wnt signaling pathway			3,9108				AXIN2 CCND1 DKK1 FZD3 MMP7 NKD1 NKD2 NOTUMPLCB4 PRKCG RUVBL1 TCF7 TP53	
852705	MicroRNAs in cancer			3,7818				ABCC1 BRCA1 CCND1 CD44 CDC25A CDC25B CDK6 CDKN2A DDIT4 DNMT1 FSCN1 FZD3 HMGA2 IGF2BP1MET PRKCG SERPINB5 SLC7A1 SOX4 TP53	
714485	2-Oxocarboxylic acid metabolism		3,5917				ACO2 GOT2 IDH1 IDH2		
82957	Arginine and proline metabolism		3,5847				ALDH18A1 CNDP2 GOT2 OAT ODC1 P4HA1		
413392	Inosine monophosphate biosynthesis, PRPP + glutamine => IMP			3,4433				PAICS PFAS PPAT	
131226	Peroxisome				3,2414				ACOT8 ACOX1 CRAT ECI2 EPHX2 HMGCL PEX26 PHYHPXMP2 SLC27A2 XDH
172847	Protein digestion and absorption				2,9425				ATP1B3 COL17A1 KCNE3 KCNK5 MEP1A MEP1BPRSS3 SLC15A1 SLC1A1 SLC36A1 SLC6A19
413380	Fatty acid biosynthesis, elongation, mitochondria				2,8971				ACAA2 ECHS1 HADHA
83004	Propanoate metabolism				2,763				ACAT1 ACSS2 ECHS1 HADHA SUCLG1 SUCLG2
82979	Amino sugar and nucleotide sugar metabolism				2,4831				CMAS HK2 MPI NANS PGM1 PMM1 UGP2

Table S5

Section S5-A: The Enriched pathways of deregulated genes in right vs left colorectal carcinoma; **Section S5-B:** The Enriched pathways of deregulated genes in colorectal carcinoma with GI-II vs GIII-IV; **Section: S5-C** The Enriched pathways of deregulated genes in colorectal carcinoma with GI-II vs GIII-IV.

Section	Category	ID	Title (or Source)	Cluster 2_logP pValues	Cluster 2_GeneSet Gene List
**S5-A**	Pathway	83036	Ribosome	3,9620	RPL10 RPL10A RPL12 RPL13 RPL14 RPL17 RPL21 RP L22 RPL26 RPL35A RPL37A RPL38 RPL39 RPL5 RPL6 RPS11 RPS15 RPS19 RPS21 RPS4X RPS5 RPS9
**S5-B**	Pathway	790012	Biosynthesis of amino acids	3,7944	ALDOB CPS1 PGAM4
	Pathway	413342	Gluconeogenesis, oxaloacetate => fructose-6P	3,5766	ALDOB PGAM4
	Pathway	814926	Carbon metabolism	3,2581	ALDOB CPS1 PGAM4
	Pathway	413340	Glycolysis (Embden-Meyerhof pathway), glucose => pyruvate	3,236	ALDOB PGAM4
**S5-C**	Pathway	82957	Arginine and proline metabolism	3,4845	CKMT2 MAOB

Table S6.

Mutated genes and different types of mutations in colorectal carcinoma

# of patients with mutations	% of mutations	GeneSymb	Samples															
			3	11	17	25	35	39	43	49	51	53	55	59	61	69	81	97
9	56,3	CEBPZ																
9	56,3	RRBP1																
8	50,0	FXR1																
8	50,0	LRPPRC																
8	50,0	NAP1L1																
8	50,0	PDE4DIP																
8	50,0	SF3B1																
7	43,8	AHCY																
7	43,8	AHNAK																
7	43,8	ALDH1A1																
7	43,8	COPB2																
7	43,8	CSDE1																
7	43,8	DDOST																
7	43,8	HNRNPU																
7	43,8	IARS																
7	43,8	ITGB1																
7	43,8	KEAP1																
7	43,8	KIF5B																
7	43,8	NCL																
7	43,8	NDUFA10																
7	43,8	OLA1																
7	43,8	PLEC																
7	43,8	REG4																
7	43,8	RSRC2																
7	43,8	YME1L1																
6	37,5	ADD3																
6	37,5	APP																
6	37,5	AXIN2																
6	37,5	CTSH																
6	37,5	ETF1																
6	37,5	FAT1																
6	37,5	FN1																
6	37,5	GOT2																
6	37,5	HDLBP																
6	37,5	IDH1																
6	37,5	ILF2																
6	37,5	KRAS																
6	37,5	MATR3																
6	37,5	NUP88																
6	37,5	P4HB																
6	37,5	PARP1																
6	37,5	PRPF40A																
6	37,5	SMARCD2																
6	37,5	SNRNP200																
6	37,5	SPTAN1																
6	37,5	SRPR																
6	37,5	TLN1																
6	37,5	U2SURP																
6	37,5	USP34																
5	31,3	ADNP																
5	31,3	ARF4																
5	31,3	ASAH1																
5	31,3	CCNI																
5	31,3	CCT3																
5	31,3	CDH17																
5	31,3	CHD4																
5	31,3	CHMP3																
5	31,3	CPNE1																
5	31,3	DDB1																
5	31,3	DDX42																
5	31,3	DERL1																
5	31,3	DNTTIP2																
5	31,3	EIF2AK1																
5	31,3	EIF3D																
5	31,3	EIF3E																
5	31,3	EIF5B																
5	31,3	ELF1																
5	31,3	ERBB2																
5	31,3	ESD																
5	31,3	FERMT1																
5	31,3	FHL2																
5	31,3	FLNA																
5	31,3	GANAB																
5	31,3	HNRNPK																
5	31,3	HSP90AA1																
5	31,3	HSPA9																
5	31,3	HSPG2																
5	31,3	HSPH1																
5	31,3	HYOU1																
5	31,3	IGHG4																
5	31,3	IMPDH2																
5	31,3	IVNS1ABP																
5	31,3	LAMB1																
5	31,3	LTA4H																
5	31,3	MAD2L1																
5	31,3	MAP4																
5	31,3	MRPL3																
5	31,3	MUC20																
5	31,3	NAMPT																
5	31,3	NCKAP1																
5	31,3	ODC1																
5	31,3	ORC4																
5	31,3	P4HA1																
5	31,3	PABPC1																
5	31,3	PAK1																
5	31,3	PLEK2																
5	31,3	POF1B																
5	31,3	POM121																

missense
frameshift
splice variant
stop gained
Inframe insertion or deletion
stop lost
mix

5	31,3	PRCP
5	31,3	RBBP7
5	31,3	RBM12
5	31,3	RBM39
5	31,3	SAFB2
5	31,3	SCRIB
5	31,3	SEL1L3
5	31,3	SNW1
5	31,3	SRP68
5	31,3	SRSF11
5	31,3	SUPT16H
5	31,3	SUV420H1
5	31,3	SYNCRIP
5	31,3	TCP1
5	31,3	TFRC
5	31,3	TKT
5	31,3	TP53
5	31,3	TPD52
5	31,3	UBAP2L
5	31,3	XRCC5
5	31,3	ZMYM2
4	25,0	ABHD12
4	25,0	ACO2
4	25,0	ACTN1
4	25,0	ACVR2A
4	25,0	ADSS
4	25,0	ALDH18A1
4	25,0	ALG3
4	25,0	ANAPC5
4	25,0	AP1B1
4	25,0	APIP
4	25,0	ARAP1
4	25,0	ARHGAP21
4	25,0	ARSE
4	25,0	ASNSD1
4	25,0	ATP8B1
4	25,0	AZIN1
4	25,0	BAZ1B
4	25,0	BRIX1
4	25,0	C1QBP
4	25,0	C1R
4	25,0	C7orf49
4	25,0	CABIN1
4	25,0	CAND1
4	25,0	CANX
4	25,0	CAPN7
4	25,0	CAPRIN1
4	25,0	CASP8
4	25,0	CBWD1
4	25,0	CCDC90B
4	25,0	CCT4
4	25,0	CCT8
4	25,0	CD46
4	25,0	CD47
4	25,0	CHPF2
4	25,0	CIC
4	25,0	CLK1
4	25,0	CNDP2
4	25,0	COL1A1
4	25,0	COPA
4	25,0	CPVL
4	25,0	CREBBP
4	25,0	CSE1L
4	25,0	CTAGE5
4	25,0	CUL4B
4	25,0	CYHR1
4	25,0	DDX11
4	25,0	DDX5
4	25,0	DGKZ
4	25,0	DLST
4	25,0	EED
4	25,0	EIF2S2
4	25,0	EIF4G2
4	25,0	EPB41L2
4	25,0	ERO1L
4	25,0	ERRFI1
4	25,0	ESRP1
4	25,0	EZR
4	25,0	FAM129B
4	25,0	FAM160A2
4	25,0	GARS
4	25,0	GBE1
4	25,0	GLT8D1
4	25,0	GLUD1
4	25,0	GNAI3
4	25,0	GPR125
4	25,0	GRSF1
4	25,0	HNRNPR
4	25,0	HNRNPUL2
4	25,0	HSP90AB1
4	25,0	IBTK
4	25,0	IDH2
4	25,0	ING1
4	25,0	IWS1
4	25,0	KARS
4	25,0	LACTB2
4	25,0	LDHB
4	25,0	LIMA1
4	25,0	LMNB1
4	25,0	MACF1

4	25,0	MCM7																
4	25,0	MCMBP																
4	25,0	MESDC2																
4	25,0	MFF																
4	25,0	MGEA5																
4	25,0	MLF2																
4	25,0	MRPL10																
4	25,0	MTHFD1																
4	25,0	MTHFD2																
4	25,0	MUC13																
4	25,0	MYH9																
4	25,0	MYOF																
4	25,0	NAE1																
4	25,0	NBN																
4	25,0	NCK2																
4	25,0	NDUFA9																
4	25,0	NFKB2																
4	25,0	NHP2L1																
4	25,0	NSFL1C																
4	25,0	NSMAF																
4	25,0	NT5DC2																
4	25,0	NUP98																
4	25,0	OAT																
4	25,0	PABPC4																
4	25,0	PAM																
4	25,0	PAPOLA																
4	25,0	PCDH1																
4	25,0	PDIA4																
4	25,0	PDLIM7																
4	25,0	PHB2																
4	25,0	PHF14																
4	25,0	PLIN2																
4	25,0	PLRG1																
4	25,0	PLXNB2																
4	25,0	PNISR																
4	25,0	PPIG																
4	25,0	PPP2R1A																
4	25,0	PPP4R1																
4	25,0	PRDX3																
4	25,0	PRKDC																
4	25,0	PRPF19																
4	25,0	PRPF3																
4	25,0	PRPF4B																
4	25,0	RAB11FIP1																
4	25,0	RBM45																
4	25,0	RBMX																
4	25,0	REPIN1																
4	25,0	RNF43																
4	25,0	RP11-407N17.3																
4	25,0	RTN4																
4	25,0	SAAL1																
4	25,0	SEC24D																
4	25,0	SF3B4																
4	25,0	SLC2A1																
4	25,0	SLC40A1																
4	25,0	SMU1																
4	25,0	SND1																
4	25,0	SNRPB																
4	25,0	SPATA2L																
4	25,0	SRPK1																
4	25,0	SRRM2																
4	25,0	SRSF6																
4	25,0	SSRP1																
4	25,0	STAT6																
4	25,0	STAU1																
4	25,0	STRBP																
4	25,0	SUCLA2																
4	25,0	TAF1D																
4	25,0	TFIP11																
4	25,0	THOC2																
4	25,0	TM2D1																
4	25,0	TM7SF3																
4	25,0	TM9SF3																
4	25,0	TNIP1																
4	25,0	TOMM70A																
4	25,0	TOP2A																
4	25,0	TOP2B																
4	25,0	TPM2																
4	25,0	TROAP																
4	25,0	TRPC4AP																
4	25,0	TTK																
4	25,0	USP1																
4	25,0	VCP																
4	25,0	WDR12																
4	25,0	XPO1																
4	25,0	ZC3H7A																
4	25,0	ZDHHC5																
	Totale		69	97	149	24	46	46	80	53	128	102	36	109	11	133	157	102

Table S7.

Significant splice events identified in colorectal cancer samples

seqname	source	feature	start	end	strand	gene id	transcript id	exon number	gene name	oId ref	nearest ref	class code	tss id	p id
chr1	Cufflinks	exon	20915444	20915776	+	XLOC 00019	TCONS 0000103	1	CDA	NM 001785	NM 001785	=	TSS403	P240
chr1	Cufflinks	exon	20931421	20931532	+	XLOC 00019	TCONS 0000103	2	CDA	NM 001785	NM 001785	=	TSS403	P240
chr1	Cufflinks	exon	20940335	20940392	+	XLOC 00019	TCONS 0000103	3	CDA	NM 001785	NM 001785	=	TSS403	P240
chr1	Cufflinks	exon	20944945	20945400	+	XLOC 00019	TCONS 0000103	4	CDA	NM 001785	NM 001785	=	TSS403	P240
chr1	Cufflinks	exon	27189633	27190947	+	XLOC 00025	TCONS 0000141	1	SFN	NM 006142	NM 006142	=	TSS541	P330
chr1	Cufflinks	exon	31885963	31886151	+	XLOC 00029	TCONS 0000163	1	SERINC2	NM 178865	NM 178865	=	TSS607	P396
chr1	Cufflinks	exon	31896540	31896701	+	XLOC 00029	TCONS 0000163	2	SERINC2	NM 178865	NM 178865	=	TSS607	P396
chr1	Cufflinks	exon	31897530	31897720	+	XLOC 00029	TCONS 0000163	3	SERINC2	NM 178865	NM 178865	=	TSS607	P396
chr1	Cufflinks	exon	31898177	31898256	+	XLOC 00029	TCONS 0000163	4	SERINC2	NM 178865	NM 178865	=	TSS607	P396
chr1	Cufflinks	exon	31898623	31898760	+	XLOC 00029	TCONS 0000163	5	SERINC2	NM 178865	NM 178865	=	TSS607	P396
chr1	Cufflinks	exon	31899501	31899670	+	XLOC 00029	TCONS 0000163	6	SERINC2	NM 178865	NM 178865	=	TSS607	P396
chr1	Cufflinks	exon	31901825	31901915	+	XLOC 00029	TCONS 0000163	7	SERINC2	NM 178865	NM 178865	=	TSS607	P396
chr1	Cufflinks	exon	31902244	31902385	+	XLOC 00029	TCONS 0000163	8	SERINC2	NM 178865	NM 178865	=	TSS607	P396
chr1	Cufflinks	exon	31905814	31906032	+	XLOC 00029	TCONS 0000163	9	SERINC2	NM 178865	NM 178865	=	TSS607	P396
chr1	Cufflinks	exon	31906911	31907527	+	XLOC 00029	TCONS 0000163	10	SERINC2	NM 178865	NM 178865	=	TSS607	P396
chr1	Cufflinks	exon	92495533	92495867	+	XLOC 00059	TCONS 0000334	1	EPHX4	NM 173567	NM 173567	=	TSS1264	P821
chr1	Cufflinks	exon	92498062	92498147	+	XLOC 00059	TCONS 0000334	2	EPHX4	NM 173567	NM 173567	=	TSS1264	P821
chr1	Cufflinks	exon	92508380	92508537	+	XLOC 00059	TCONS 0000334	3	EPHX4	NM 173567	NM 173567	=	TSS1264	P821
chr1	Cufflinks	exon	92511089	92511217	+	XLOC 00059	TCONS 0000334	4	EPHX4	NM 173567	NM 173567	=	TSS1264	P821
chr1	Cufflinks	exon	92515874	92515977	+	XLOC 00059	TCONS 0000334	5	EPHX4	NM 173567	NM 173567	=	TSS1264	P821
chr1	Cufflinks	exon	92518067	92518215	+	XLOC 00059	TCONS 0000334	6	EPHX4	NM 173567	NM 173567	=	TSS1264	P821
chr1	Cufflinks	exon	92528612	92529093	+	XLOC 00059	TCONS 0000334	7	EPHX4	NM 173567	NM 173567	=	TSS1264	P821
chr1	Cufflinks	exon	151483862	151483991	+	XLOC 00082	TCONS 0000457	1	CGN	NM 020770	NM 020770	=	TSS1750	P1099
chr1	Cufflinks	exon	151490982	151491868	+	XLOC 00082	TCONS 0000457	2	CGN	NM 020770	NM 020770	=	TSS1750	P1099
chr1	Cufflinks	exon	151492642	151492742	+	XLOC 00082	TCONS 0000457	3	CGN	NM 020770	NM 020770	=	TSS1750	P1099
chr1	Cufflinks	exon	151492890	151492959	+	XLOC 00082	TCONS 0000457	4	CGN	NM 020770	NM 020770	=	TSS1750	P1099
chr1	Cufflinks	exon	151493072	151493167	+	XLOC 00082	TCONS 0000457	5	CGN	NM 020770	NM 020770	=	TSS1750	P1099
chr1	Cufflinks	exon	151495910	151496037	+	XLOC 00082	TCONS 0000457	6	CGN	NM 020770	NM 020770	=	TSS1750	P1099
chr1	Cufflinks	exon	151496702	151496834	+	XLOC 00082	TCONS 0000457	7	CGN	NM 020770	NM 020770	=	TSS1750	P1099
chr1	Cufflinks	exon	151497150	151497362	+	XLOC 00082	TCONS 0000457	8	CGN	NM 020770	NM 020770	=	TSS1750	P1099
chr1	Cufflinks	exon	151498118	151498266	+	XLOC 00082	TCONS 0000457	9	CGN	NM 020770	NM 020770	=	TSS1750	P1099
chr1	Cufflinks	exon	151499451	151499583	+	XLOC 00082	TCONS 0000457	10	CGN	NM 020770	NM 020770	=	TSS1750	P1099
chr1	Cufflinks	exon	151501826	151502035	+	XLOC 00082	TCONS 0000457	11	CGN	NM 020770	NM 020770	=	TSS1750	P1099
chr1	Cufflinks	exon	151502385	151502591	+	XLOC 00082	TCONS 0000457	12	CGN	NM 020770	NM 020770	=	TSS1750	P1099
chr1	Cufflinks	exon	151502965	151503222	+	XLOC 00082	TCONS 0000457	13	CGN	NM 020770	NM 020770	=	TSS1750	P1099
chr1	Cufflinks	exon	151504878	151505048	+	XLOC 00082	TCONS 0000457	14	CGN	NM 020770	NM 020770	=	TSS1750	P1099
chr1	Cufflinks	exon	151506451	151506612	+	XLOC 00082	TCONS 0000457	15	CGN	NM 020770	NM 020770	=	TSS1750	P1099
chr1	Cufflinks	exon	151507518	151507607	+	XLOC 00082	TCONS 0000457	16	CGN	NM 020770	NM 020770	=	TSS1750	P1099
chr1	Cufflinks	exon	151508076	151508159	+	XLOC 00082	TCONS 0000457	17	CGN	NM 020770	NM 020770	=	TSS1750	P1099
chr1	Cufflinks	exon	151508256	151508374	+	XLOC 00082	TCONS 0000457	18	CGN	NM 020770	NM 020770	=	TSS1750	P1099
chr1	Cufflinks	exon	151508713	151508821	+	XLOC 00082	TCONS 0000457	19	CGN	NM 020770	NM 020770	=	TSS1750	P1099
chr1	Cufflinks	exon	151509206	151509369	+	XLOC 00082	TCONS 0000457	20	CGN	NM 020770	NM 020770	=	TSS1750	P1099
chr1	Cufflinks	exon	151509681	151511167	+	XLOC 00082	TCONS 0000457	21	CGN	NM 020770	NM 020770	=	TSS1750	P1099
chr1	Cufflinks	exon	153190060	153190095	+	XLOC 00085	TCONS 0000464	1	PRR9	NM 001195571	NM 001195571	=	TSS1801	P1132
chr1	Cufflinks	exon	153190601	153191793	+	XLOC 00085	TCONS 0000464	2	PRR9	NM 001195571	NM 001195571	=	TSS1801	P1132
chr1	Cufflinks	exon	154975106	154975162	+	XLOC 00088	TCONS 0000479	1	ZBTB7B	NR 049765	NR 049765	=	TSS1854	
chr1	Cufflinks	exon	154975319	154975419	+	XLOC 00088	TCONS 0000479	2	ZBTB7B	NR 049765	NR 049765	=	TSS1854	
chr1	Cufflinks	exon	154987131	154988290	+	XLOC 00088	TCONS 0000479	3	ZBTB7B	NR 049765	NR 049765	=	TSS1854	
chr1	Cufflinks	exon	154988696	154991001	+	XLOC 00088	TCONS 0000479	4	ZBTB7B	NR 049765	NR 049765	=	TSS1854	
chr1	Cufflinks	exon	155100349	155100545	+	XLOC 00088	TCONS 0000484	1	EFNA1	NM 004428	NM 004428	=	TSS1864	P1185
chr1	Cufflinks	exon	155103815	155104110	+	XLOC 00088	TCONS 0000484	2	EFNA1	NM 004428	NM 004428	=	TSS1864	P1185
chr1	Cufflinks	exon	155105979	155106044	+	XLOC 00088	TCONS 0000484	3	EFNA1	NM 004428	NM 004428	=	TSS1864	P1185
chr1	Cufflinks	exon	155106206	155106256	+	XLOC 00088	TCONS 0000484	4	EFNA1	NM 004428	NM 004428	=	TSS1864	P1185
chr1	Cufflinks	exon	155106431	155107386	+	XLOC 00088	TCONS 0000484	5	EFNA1	NM 004428	NM 004428	=	TSS1864	P1185
chr1	Cufflinks	exon	159931014	159931195	+	XLOC 00094	TCONS 0000519	1	LINC01133	NR 038849	NR 038849	=	TSS1980	
chr1	Cufflinks	exon	159946434	159946902	+	XLOC 00094	TCONS 0000519	2	LINC01133	NR 038849	NR 038849	=	TSS1980	
chr1	Cufflinks	exon	159948390	159948876	+	XLOC 00094	TCONS 0000519	3	LINC01133	NR 038849	NR 038849	=	TSS1980	
chr1	Cufflinks	exon	220863628	220864053	+	XLOC 00124	TCONS 0000687	1	C1orf115	NM 024709	NM 024709	=	TSS2603	P1757
chr1	Cufflinks	exon	220869954	220872499	+	XLOC 00124	TCONS 0000687	2	C1orf115	NM 024709	NM 024709	=	TSS2603	P1757
chr1	Cufflinks	exon	228780394	228780779	+	XLOC 00129	TCONS 0000711	1	RHOU	NR 037962	NR 037962	=	TSS2696	
chr1	Cufflinks	exon	228873420	228873478	+	XLOC 00129	TCONS 0000711	2	RHOU	NR 037962	NR 037962	=	TSS2696	
chr1	Cufflinks	exon	228879032	228882416	+	XLOC 00129	TCONS 0000711	3	RHOU	NR 037962	NR 037962	=	TSS2696	
chr1	Cufflinks	exon	20808884	20810212	-	XLOC 00157	TCONS 0000853	1	CAMK2N1	NM 018584	NM 018584	=	TSS3275	P2146
chr1	Cufflinks	exon	20811707	20812728	-	XLOC 00157	TCONS 0000853	2	CAMK2N1	NM 018584	NM 018584	=	TSS3275	P2146
chr1	Cufflinks	exon	24171572	24172345	-	XLOC 00160	TCONS 0000875	1	FUCA1	NM 000147	NM 000147	=	TSS3344	P2191
chr1	Cufflinks	exon	24172564	24172663	-	XLOC 00160	TCONS 0000875	2	FUCA1	NM 000147	NM 000147	=	TSS3344	P2191
chr1	Cufflinks	exon	24175139	24175329	-	XLOC 00160	TCONS 0000875	3	FUCA1	NM 000147	NM 000147	=	TSS3344	P2191
chr1	Cufflinks	exon	24180850	24181050	-	XLOC 00160	TCONS 0000875	4	FUCA1	NM 000147	NM 000147	=	TSS3344	P2191
chr1	Cufflinks	exon	24186288	24186393	-	XLOC 00160	TCONS 0000875	5	FUCA1	NM 000147	NM 000147	=	TSS3344	P2191
chr1	Cufflinks	exon	24189624	24189761	-	XLOC 00160	TCONS 0000875	6	FUCA1	NM 000147	NM 000147	=	TSS3344	P2191
chr1	Cufflinks	exon	24191981	24192115	-	XLOC 00160	TCONS 0000875	7	FUCA1	NM 000147	NM 000147	=	TSS3344	P2191
chr1	Cufflinks	exon	24194288	24194859	-	XLOC 00160	TCONS 0000875	8	FUCA1	NM 000147	NM 000147	=	TSS3344	P2191
chr1	Cufflinks	exon	33360196	33360480	-	XLOC 00169	TCONS 0000927	1	TMEM54	NM 033504	NM 033504	=	TSS3528	P2302
chr1	Cufflinks	exon	33360906	33361040	-	XLOC 00169	TCONS 0000927	2	TMEM54	NM 033504	NM 033504	=	TSS3528	P2302
chr1	Cufflinks	exon	33361156	33361344	-	XLOC 00169	TCONS 0000927	3	TMEM54	NM 033504	NM 033504	=	TSS3528	P2302
chr1	Cufflinks	exon	33361511	33361570	-	XLOC 00169	TCONS 0000927	4	TMEM54	NM 033504	NM 033504	=	TSS3528	P2302
chr1	Cufflinks	exon	33363727	33363920	-	XLOC 00169	TCONS 0000927	5	TMEM54	NM 033504	NM 033504	=	TSS3528	P2302
chr1	Cufflinks	exon	33366824	33366953	-	XLOC 00169	TCONS 0000927	6	TMEM54	NM 033504	NM 033504	=	TSS3528	P2302
chr1	Cufflinks	exon	43747557	43748778	-	XLOC 00177	TCONS 0000979	1	C1orf210	NM 182517	NM 182517	=	TSS3707	P2416
chr1	Cufflinks	exon	43748923	43749039	-	XLOC 00177	TCONS 0000979	2	C1orf210	NM 182517	NM 182517	=	TSS3707	P2416
chr1	Cufflinks	exon	43751115	43751250	-	XLOC 00177	TCONS 0000979	3	C1orf210	NM 182517	NM 182517	=	TSS3707	P2416
chr1	Cufflinks	exon	120290619	120291464	-	XLOC 00209	TCONS 0001166	1	HMGCS2	NM 005518	NM 005518	=	TSS4373	P2835
chr1	Cufflinks	exon	120293420	120293531	-	XLOC 00209	TCONS 0001166	2	HMGCS2	NM 005518	NM 005518	=	TSS4373	P2835
chr1	Cufflinks	exon	120295172	120295297	-	XLOC 00209	TCONS 0001166	3	HMGCS2	NM 005518	NM 005518	=	TSS4373	P2835
chr1	Cufflinks	exon	120295903	120296009	-	XLOC 00209	TCONS 0001166	4	HMGCS2	NM 005518	NM 005518	=	TSS4373	P2835
chr1	Cufflinks	exon	120298050	120298220	-	XLOC 00209	TCONS 0001166	5	HMGCS2	NM 005518	NM 005518	=	TSS4373	P2835
chr1	Cufflinks	exon	120299896	120300061	-	XLOC 00209	TCONS 0001166	6	HMGCS2	NM 005518	NM 005518	=	TSS4373	P2835
chr1	Cufflinks	exon	120301741	120301905	-	XLOC 00209	TCONS 0001166	7	HMGCS2	NM 005518	NM 005518	=	TSS4373	P2835
chr1	Cufflinks	exon	120302487	120302612	-	XLOC 00209	TCONS 0001166	8	HMGCS2	NM 005518	NM 005518	=	TSS4373	P2835
chr1	Cufflinks	exon	120306795	120307249	-	XLOC 00209	TCONS 0001166	9	HMGCS2	NM 005518	NM 005518	=	TSS4373	P2835
chr1	Cufflinks	exon	120311364	120311555	-	XLOC 00209	TCONS 0001166	10	HMGCS2	NM 005518	NM 005518	=	TSS4373	P2835
chr1	Cufflinks	exon	167022073	167023703	-	XLOC 00235	TCONS 0001318	1	GPA33	NM 005814	NM 005814	=	TSS4910	P3270
chr1	Cufflinks	exon	167024213	167024348	-	XLOC 00235	TCONS 0001318	2	GPA33	NM 005814	NM 005814	=	TSS4910	P3270
chr1	Cufflinks	exon	167024967	167025086	-	XLOC 00235	TCONS 0001318	3	GPA33	NM 005814	NM 005814	=	TSS4910	P3270
chr1	Cufflinks	exon	167032819	167032974	-	XLOC 00235	TCONS 0001318	4	GPA33	NM 005814	NM 005814	=	TSS4910	P3270
chr1	Cufflinks	exon	167038159	167038375	-	XLOC 00235	TCONS 0001318	5	GPA33	NM 005814	NM 005814	=	TSS4910	P3270
chr1	Cufflinks	exon	167042622	167042776	-	XLOC 00235	TCONS 0001318	6	GPA33	NM 005814	NM 005814	=	TSS4910	P3270
chr1	Cufflinks	exon	167059482	167059868	-	XLOC 00235	TCONS 0001318	7	GPA33	NM 005814	NM 005814	=	TSS4910	P3270
chr1	Cufflinks	exon	27425300	27427135	-	XLOC 00164	TCONS 0000894	1	SLC9A1	CUFF.671.3	NM 003047	j	TSS3417	

chr1	Cufflinks	exon	27427694	27427766	-	XLOC 00164	TCONS 0000894	2	SLC9A1	CUFF.671.3	NM 003047	j	TSS3417	
chr1	Cufflinks	exon	27428216	27428317	-	XLOC 00164	TCONS 0000894	3	SLC9A1	CUFF.671.3	NM 003047	j	TSS3417	
chr1	Cufflinks	exon	27428507	27428621	-	XLOC 00164	TCONS 0000894	4	SLC9A1	CUFF.671.3	NM 003047	j	TSS3417	
chr1	Cufflinks	exon	27428876	27429049	-	XLOC 00164	TCONS 0000894	5	SLC9A1	CUFF.671.3	NM 003047	j	TSS3417	
chr1	Cufflinks	exon	27429164	27429234	-	XLOC 00164	TCONS 0000894	6	SLC9A1	CUFF.671.3	NM 003047	j	TSS3417	
chr1	Cufflinks	exon	27429714	27429803	-	XLOC 00164	TCONS 0000894	7	SLC9A1	CUFF.671.3	NM 003047	j	TSS3417	
chr1	Cufflinks	exon	27432376	27432578	-	XLOC 00164	TCONS 0000894	8	SLC9A1	CUFF.671.3	NM 003047	j	TSS3417	
chr1	Cufflinks	exon	27434139	27434356	-	XLOC 00164	TCONS 0000894	9	SLC9A1	CUFF.671.3	NM 003047	j	TSS3417	
chr1	Cufflinks	exon	27436018	27436268	-	XLOC 00164	TCONS 0000894	10	SLC9A1	CUFF.671.3	NM 003047	j	TSS3417	
chr1	Cufflinks	exon	27440317	27440777	-	XLOC 00164	TCONS 0000894	11	SLC9A1	CUFF.671.3	NM 003047	j	TSS3417	
chr1	Cufflinks	exon	27480474	27480852	-	XLOC 00164	TCONS 0000894	12	SLC9A1	CUFF.671.3	NM 003047	j	TSS3417	
chr1	Cufflinks	exon	27481845	27481888	-	XLOC 00164	TCONS 0000894	13	SLC9A1	CUFF.671.3	NM 003047	j	TSS3417	
chr1	Cufflinks	exon	55245380	55247452	-	XLOC 00185	TCONS 0001039	1	TTC22	CUFF.1311.3	NM 001114108	j	TSS3898	
chr1	Cufflinks	exon	55247998	55248150	-	XLOC 00185	TCONS 0001039	2	TTC22	CUFF.1311.3	NM 001114108	j	TSS3898	
chr1	Cufflinks	exon	55251656	55251817	-	XLOC 00185	TCONS 0001039	3	TTC22	CUFF.1311.3	NM 001114108	j	TSS3898	
chr1	Cufflinks	exon	55252630	55252748	-	XLOC 00185	TCONS 0001039	4	TTC22	CUFF.1311.3	NM 001114108	j	TSS3898	
chr1	Cufflinks	exon	55253384	55253500	-	XLOC 00185	TCONS 0001039	5	TTC22	CUFF.1311.3	NM 001114108	j	TSS3898	
chr1	Cufflinks	exon	55253715	55253770	-	XLOC 00185	TCONS 0001039	6	TTC22	CUFF.1311.3	NM 001114108	j	TSS3898	
chr1	Cufflinks	exon	55266270	55267152	-	XLOC 00185	TCONS 0001039	7	TTC22	CUFF.1311.3	NM 001114108	j	TSS3898	
chr1	Cufflinks	exon	120336641	120337308	-	XLOC 00209	TCONS 0001166	1	REG4	CUFF.2192.3	NM 001159352	j	TSS4377	
chr1	Cufflinks	exon	120341120	120341225	-	XLOC 00209	TCONS 0001166	2	REG4	CUFF.2192.3	NM 001159352	j	TSS4377	
chr1	Cufflinks	exon	120342348	120342485	-	XLOC 00209	TCONS 0001166	3	REG4	CUFF.2192.3	NM 001159352	j	TSS4377	
chr1	Cufflinks	exon	120345691	120345788	-	XLOC 00209	TCONS 0001166	4	REG4	CUFF.2192.3	NM 001159352	j	TSS4377	
chr1	Cufflinks	exon	120351326	120351486	-	XLOC 00209	TCONS 0001166	5	REG4	CUFF.2192.3	NM 001159352	j	TSS4377	
chr1	Cufflinks	exon	120354032	120354123	-	XLOC 00209	TCONS 0001166	6	REG4	CUFF.2192.3	NM 001159352	j	TSS4377	
chr1	Cufflinks	exon	120355307	120355341	-	XLOC 00209	TCONS 0001166	7	REG4	CUFF.2192.3	NM 001159352	j	TSS4377	
chr1	Cufflinks	exon	204586302	204589346	-	XLOC 00252	TCONS 0001398	1	LRRN2	CUFF.3453.1	NM 006338	j	TSS5238	
chr1	Cufflinks	exon	204602272	204602377	-	XLOC 00252	TCONS 0001398	2	LRRN2	CUFF.3453.1	NM 006338	j	TSS5238	
chr10	Cufflinks	exon	54074041	54074437	+	XLOC 00341	TCONS 0001665	1	DKK1	NM 012242	NM 012242	=	TSS6554	P3918
chr10	Cufflinks	exon	54074683	54074845	+	XLOC 00341	TCONS 0001665	2	DKK1	NM 012242	NM 012242	=	TSS6554	P3918
chr10	Cufflinks	exon	54076055	54076195	+	XLOC 00341	TCONS 0001665	3	DKK1	NM 012242	NM 012242	=	TSS6554	P3918
chr10	Cufflinks	exon	54076314	54077417	+	XLOC 00341	TCONS 0001665	4	DKK1	NM 012242	NM 012242	=	TSS6554	P3918
chr10	Cufflinks	exon	74033677	74033818	+	XLOC 00347	TCONS 0001696	1	DDIT4	NM 019058	NM 019058	=	TSS6686	P3999
chr10	Cufflinks	exon	74033915	74034179	+	XLOC 00347	TCONS 0001696	2	DDIT4	NM 019058	NM 019058	=	TSS6686	P3999
chr10	Cufflinks	exon	74034453	74035797	+	XLOC 00347	TCONS 0001696	3	DDIT4	NM 019058	NM 019058	=	TSS6686	P3999
chr10	Cufflinks	exon	85933554	85933677	+	XLOC 00353	TCONS 0001730	1	C10orf99	NM 207373	NM 207373	=	TSS6818	P4069
chr10	Cufflinks	exon	85936236	85936325	+	XLOC 00353	TCONS 0001730	2	C10orf99	NM 207373	NM 207373	=	TSS6818	P4069
chr10	Cufflinks	exon	85944437	85945050	+	XLOC 00353	TCONS 0001730	3	C10orf99	NM 207373	NM 207373	=	TSS6818	P4069
chr10	Cufflinks	exon	112631553	112631776	+	XLOC 00368	TCONS 0001824	1	PDCD4	NM 014456	NM 014456	=	TSS7158	P4299
chr10	Cufflinks	exon	112635724	112635828	+	XLOC 00368	TCONS 0001824	2	PDCD4	NM 014456	NM 014456	=	TSS7158	P4299
chr10	Cufflinks	exon	112640991	112641293	+	XLOC 00368	TCONS 0001824	3	PDCD4	NM 014456	NM 014456	=	TSS7158	P4299
chr10	Cufflinks	exon	112642761	112642855	+	XLOC 00368	TCONS 0001824	4	PDCD4	NM 014456	NM 014456	=	TSS7158	P4299
chr10	Cufflinks	exon	112645011	112645124	+	XLOC 00368	TCONS 0001824	5	PDCD4	NM 014456	NM 014456	=	TSS7158	P4299
chr10	Cufflinks	exon	112647423	112647644	+	XLOC 00368	TCONS 0001824	6	PDCD4	NM 014456	NM 014456	=	TSS7158	P4299
chr10	Cufflinks	exon	112649291	112649388	+	XLOC 00368	TCONS 0001824	7	PDCD4	NM 014456	NM 014456	=	TSS7158	P4299
chr10	Cufflinks	exon	112650314	112650428	+	XLOC 00368	TCONS 0001824	8	PDCD4	NM 014456	NM 014456	=	TSS7158	P4299
chr10	Cufflinks	exon	112653849	112653956	+	XLOC 00368	TCONS 0001824	9	PDCD4	NM 014456	NM 014456	=	TSS7158	P4299
chr10	Cufflinks	exon	112654170	112654280	+	XLOC 00368	TCONS 0001824	10	PDCD4	NM 014456	NM 014456	=	TSS7158	P4299
chr10	Cufflinks	exon	112655706	112655845	+	XLOC 00368	TCONS 0001824	11	PDCD4	NM 014456	NM 014456	=	TSS7158	P4299
chr10	Cufflinks	exon	112657786	112659764	+	XLOC 00368	TCONS 0001824	12	PDCD4	NM 014456	NM 014456	=	TSS7158	P4299
chr10	Cufflinks	exon	115469104	115469205	+	XLOC 00369	TCONS 0001833	1	CASP7	NM 001267058	NM 001267058	=	TSS7185	P4326
chr10	Cufflinks	exon	115480791	115480927	+	XLOC 00369	TCONS 0001833	2	CASP7	NM 001267058	NM 001267058	=	TSS7185	P4326
chr10	Cufflinks	exon	115481410	115481538	+	XLOC 00369	TCONS 0001833	3	CASP7	NM 001267058	NM 001267058	=	TSS7185	P4326
chr10	Cufflinks	exon	115485121	115485296	+	XLOC 00369	TCONS 0001833	4	CASP7	NM 001267058	NM 001267058	=	TSS7185	P4326
chr10	Cufflinks	exon	115486064	115486193	+	XLOC 00369	TCONS 0001833	5	CASP7	NM 001267058	NM 001267058	=	TSS7185	P4326
chr10	Cufflinks	exon	115489070	115490668	+	XLOC 00369	TCONS 0001833	6	CASP7	NM 001267058	NM 001267058	=	TSS7185	P4326
chr10	Cufflinks	exon	13319796	13320354	-	XLOC 00385	TCONS 0001908	1	PHYH	NM 006214	NM 006214	=	TSS7472	P4476
chr10	Cufflinks	exon	13322976	13323110	-	XLOC 00385	TCONS 0001908	2	PHYH	NM 006214	NM 006214	=	TSS7472	P4476
chr10	Cufflinks	exon	13325690	13325839	-	XLOC 00385	TCONS 0001908	3	PHYH	NM 006214	NM 006214	=	TSS7472	P4476
chr10	Cufflinks	exon	13330360	13330541	-	XLOC 00385	TCONS 0001908	4	PHYH	NM 006214	NM 006214	=	TSS7472	P4476
chr10	Cufflinks	exon	13333831	13333912	-	XLOC 00385	TCONS 0001908	5	PHYH	NM 006214	NM 006214	=	TSS7472	P4476
chr10	Cufflinks	exon	13336428	13336596	-	XLOC 00385	TCONS 0001908	6	PHYH	NM 006214	NM 006214	=	TSS7472	P4476
chr10	Cufflinks	exon	13337496	13337606	-	XLOC 00385	TCONS 0001908	7	PHYH	NM 006214	NM 006214	=	TSS7472	P4476
chr10	Cufflinks	exon	13340187	13340245	-	XLOC 00385	TCONS 0001908	8	PHYH	NM 006214	NM 006214	=	TSS7472	P4476
chr10	Cufflinks	exon	13341968	13342130	-	XLOC 00385	TCONS 0001908	9	PHYH	NM 006214	NM 006214	=	TSS7472	P4476
chr10	Cufflinks	exon	21068903	21074852	-	XLOC 00388	TCONS 0001930	1	NEBL	NM 213569	NM 213569	=	TSS7549	P4507
chr10	Cufflinks	exon	21076131	21076237	-	XLOC 00388	TCONS 0001930	2	NEBL	NM 213569	NM 213569	=	TSS7549	P4507
chr10	Cufflinks	exon	21101698	21101869	-	XLOC 00388	TCONS 0001930	3	NEBL	NM 213569	NM 213569	=	TSS7549	P4507
chr10	Cufflinks	exon	21250601	21250708	-	XLOC 00388	TCONS 0001930	4	NEBL	NM 213569	NM 213569	=	TSS7549	P4507
chr10	Cufflinks	exon	21309046	21309130	-	XLOC 00388	TCONS 0001930	5	NEBL	NM 213569	NM 213569	=	TSS7549	P4507
chr10	Cufflinks	exon	21461312	21461406	-	XLOC 00388	TCONS 0001930	6	NEBL	NM 213569	NM 213569	=	TSS7549	P4507
chr10	Cufflinks	exon	21462694	21463116	-	XLOC 00388	TCONS 0001930	7	NEBL	NM 213569	NM 213569	=	TSS7549	P4507
chr10	Cufflinks	exon	96997325	96997868	-	XLOC 00415	TCONS 0002087	1	PDLIM1	NM 020992	NM 020992	=	TSS8104	P4861
chr10	Cufflinks	exon	96998325	96998442	-	XLOC 00415	TCONS 0002087	2	PDLIM1	NM 020992	NM 020992	=	TSS8104	P4861
chr10	Cufflinks	exon	97006972	97007123	-	XLOC 00415	TCONS 0002087	3	PDLIM1	NM 020992	NM 020992	=	TSS8104	P4861
chr10	Cufflinks	exon	97023621	97023820	-	XLOC 00415	TCONS 0002087	4	PDLIM1	NM 020992	NM 020992	=	TSS8104	P4861
chr10	Cufflinks	exon	97028535	97028619	-	XLOC 00415	TCONS 0002087	5	PDLIM1	NM 020992	NM 020992	=	TSS8104	P4861
chr10	Cufflinks	exon	97031390	97031541	-	XLOC 00415	TCONS 0002087	6	PDLIM1	NM 020992	NM 020992	=	TSS8104	P4861
chr10	Cufflinks	exon	97050577	97050905	-	XLOC 00415	TCONS 0002087	7	PDLIM1	NM 020992	NM 020992	=	TSS8104	P4861
chr10	Cufflinks	exon	99437181	99437782	-	XLOC 00417	TCONS 0002104	1	AVPI1	NM 021732	NM 021732	=	TSS8152	P4905
chr10	Cufflinks	exon	99439376	99439672	-	XLOC 00417	TCONS 0002104	2	AVPI1	NM 021732	NM 021732	=	TSS8152	P4905
chr10	Cufflinks	exon	99446523	99447015	-	XLOC 00417	TCONS 0002104	3	AVPI1	NM 021732	NM 021732	=	TSS8152	P4905
chr11	Cufflinks	exon	102267056	102269622	-	XLOC 00604	TCONS 0002995	1	TMEM123	NM 052932	NM 052932	=	TSS11655	P7017
chr11	Cufflinks	exon	2923512	2923594	+	XLOC 00463	TCONS 0002260	1	SLC22A18	NM 002555	NM 002555	=	TSS8866	P5188
chr11	Cufflinks	exon	2924444	2924719	+	XLOC 00463	TCONS 0002260	2	SLC22A18	NM 002555	NM 002555	=	TSS8866	P5188
chr11	Cufflinks	exon	2929463	2929560	+	XLOC 00463	TCONS 0002260	3	SLC22A18	NM 002555	NM 002555	=	TSS8866	P5188
chr11	Cufflinks	exon	2930426	2930586	+	XLOC 00463	TCONS 0002260	4	SLC22A18	NM 002555	NM 002555	=	TSS8866	P5188
chr11	Cufflinks	exon	2930808	2930940	+	XLOC 00463	TCONS 0002260	5	SLC22A18	NM 002555	NM 002555	=	TSS8866	P5188
chr11	Cufflinks	exon	2937852	2937970	+	XLOC 00463	TCONS 0002260	6	SLC22A18	NM 002555	NM 002555	=	TSS8866	P5188
chr11	Cufflinks	exon	2939218	2939325	+	XLOC 00463	TCONS 0002260	7	SLC22A18	NM 002555	NM 002555	=	TSS8866	P5188
chr11	Cufflinks	exon	2940537	2940637	+	XLOC 00463	TCONS 0002260	8	SLC22A18	NM 002555	NM 002555	=	TSS8866	P5188
chr11	Cufflinks	exon	2943332	2943433	+	XLOC 00463	TCONS 0002260	9	SLC22A18	NM 002555	NM 002555	=	TSS8866	P5188
chr11	Cufflinks	exon	2943666	2943786	+	XLOC 00463	TCONS 0002260	10	SLC22A18	NM 002555	NM 002555	=	TSS8866	P5188
chr11	Cufflinks	exon	2946240	2946476	+	XLOC 00463	TCONS 0002260	11	SLC22A18	NM 002555	NM 002555	=	TSS8866	P5188
chr11	Cufflinks	exon	63304273	63304349	+	XLOC 00498	TCONS 0002423	1	RARRES3	NM 004585	NM 004585	=	TSS9505	P5656
chr11	Cufflinks	exon	63306988	63307096	+	XLOC 00498	TCONS 0002423	2	RARRES3	NM 004585	NM 004585	=	TSS9505	P5656
chr11	Cufflinks	exon	63312093	63312361	+	XLOC 00498	TCONS 0002423	3	RARRES3	NM 004585	NM 004585	=	TSS9505	P5656
chr11	Cufflinks	exon	63313621	63313930	+	XLOC 00498	TCONS 0002423	4	RARRES3	NM 004585	NM 004585	=	TSS9505	P5656
chr11	Cufflinks	exon	69455873	69456279	+	XLOC 00509	TCONS 0002502	1	CCND1	NM 053056	NM 053056	=	TSS9750	P5832
chr11	Cufflinks	exon	69457799	69458014	+	XLOC 00509	TCONS 0002502	2	CCND1	NM 053056	NM 053056	=	TSS9750	P5832

chr11	Cufflinks	exon	69458600	69458759	+	XLOC 00509	TCONS 0002502	3	CCND1	NM 053056	NM 053056	=	TSS9750	P5832
chr11	Cufflinks	exon	69462762	69462910	+	XLOC 00509	TCONS 0002502	4	CCND1	NM 053056	NM 053056	=	TSS9750	P5832
chr11	Cufflinks	exon	69465886	69469242	+	XLOC 00509	TCONS 0002502	5	CCND1	NM 053056	NM 053056	=	TSS9750	P5832
chr11	Cufflinks	exon	2289728	2290356	-	XLOC 00545	TCONS 0002702	1	ASCL2	NM 005170	NM 005170	=	TSS10504	P6266
chr11	Cufflinks	exon	2290963	2292182	-	XLOC 00545	TCONS 0002702	2	ASCL2	NM 005170	NM 005170	=	TSS10504	P6266
chr11	Cufflinks	exon	63320242	63320537	-	XLOC 00581	TCONS 0002868	1	HRASLS2	NM 017878	NM 017878	=	TSS11168	P6717
chr11	Cufflinks	exon	63325864	63326132	-	XLOC 00581	TCONS 0002868	2	HRASLS2	NM 017878	NM 017878	=	TSS11168	P6717
chr11	Cufflinks	exon	63327557	63327665	-	XLOC 00581	TCONS 0002868	3	HRASLS2	NM 017878	NM 017878	=	TSS11168	P6717
chr11	Cufflinks	exon	63330788	63330855	-	XLOC 00581	TCONS 0002868	4	HRASLS2	NM 017878	NM 017878	=	TSS11168	P6717
chr11	Cufflinks	exon	68522351	68525198	-	XLOC 00591	TCONS 0002923	1	CPT1A	NM 001876	NM 001876	=	TSS11377	P6853
chr11	Cufflinks	exon	68527037	68527129	-	XLOC 00591	TCONS 0002923	2	CPT1A	NM 001876	NM 001876	=	TSS11377	P6853
chr11	Cufflinks	exon	68527693	68527806	-	XLOC 00591	TCONS 0002923	3	CPT1A	NM 001876	NM 001876	=	TSS11377	P6853
chr11	Cufflinks	exon	68529003	68529155	-	XLOC 00591	TCONS 0002923	4	CPT1A	NM 001876	NM 001876	=	TSS11377	P6853
chr11	Cufflinks	exon	68530095	68530229	-	XLOC 00591	TCONS 0002923	5	CPT1A	NM 001876	NM 001876	=	TSS11377	P6853
chr11	Cufflinks	exon	68540733	68540897	-	XLOC 00591	TCONS 0002923	6	CPT1A	NM 001876	NM 001876	=	TSS11377	P6853
chr11	Cufflinks	exon	68542784	68542900	-	XLOC 00591	TCONS 0002923	7	CPT1A	NM 001876	NM 001876	=	TSS11377	P6853
chr11	Cufflinks	exon	68548108	68548213	-	XLOC 00591	TCONS 0002923	8	CPT1A	NM 001876	NM 001876	=	TSS11377	P6853
chr11	Cufflinks	exon	68549239	68549427	-	XLOC 00591	TCONS 0002923	9	CPT1A	NM 001876	NM 001876	=	TSS11377	P6853
chr11	Cufflinks	exon	68552283	68552478	-	XLOC 00591	TCONS 0002923	10	CPT1A	NM 001876	NM 001876	=	TSS11377	P6853
chr11	Cufflinks	exon	68560783	68560870	-	XLOC 00591	TCONS 0002923	11	CPT1A	NM 001876	NM 001876	=	TSS11377	P6853
chr11	Cufflinks	exon	68562272	68562379	-	XLOC 00591	TCONS 0002923	12	CPT1A	NM 001876	NM 001876	=	TSS11377	P6853
chr11	Cufflinks	exon	68564324	68564401	-	XLOC 00591	TCONS 0002923	13	CPT1A	NM 001876	NM 001876	=	TSS11377	P6853
chr11	Cufflinks	exon	68566686	68566823	-	XLOC 00591	TCONS 0002923	14	CPT1A	NM 001876	NM 001876	=	TSS11377	P6853
chr11	Cufflinks	exon	68571468	68571569	-	XLOC 00591	TCONS 0002923	15	CPT1A	NM 001876	NM 001876	=	TSS11377	P6853
chr11	Cufflinks	exon	68574935	68575106	-	XLOC 00591	TCONS 0002923	16	CPT1A	NM 001876	NM 001876	=	TSS11377	P6853
chr11	Cufflinks	exon	68579905	68580044	-	XLOC 00591	TCONS 0002923	17	CPT1A	NM 001876	NM 001876	=	TSS11377	P6853
chr11	Cufflinks	exon	68582802	68582955	-	XLOC 00591	TCONS 0002923	18	CPT1A	NM 001876	NM 001876	=	TSS11377	P6853
chr11	Cufflinks	exon	68609243	68609399	-	XLOC 00591	TCONS 0002923	19	CPT1A	NM 001876	NM 001876	=	TSS11377	P6853
chr11	Cufflinks	exon	102272270	102272423	-	XLOC 00604	TCONS 0002995	2	TMEM123	NM 052932	NM 052932	=	TSS11655	P7017
chr11	Cufflinks	exon	102272647	102272937	-	XLOC 00604	TCONS 0002995	3	TMEM123	NM 052932	NM 052932	=	TSS11655	P7017
chr11	Cufflinks	exon	102319543	102319599	-	XLOC 00604	TCONS 0002995	4	TMEM123	NM 052932	NM 052932	=	TSS11655	P7017
chr11	Cufflinks	exon	102323255	102323775	-	XLOC 00604	TCONS 0002995	5	TMEM123	NM 052932	NM 052932	=	TSS11655	P7017
chr11	Cufflinks	exon	102391239	102391535	-	XLOC 00604	TCONS 0002995	1	MMP7	NM 002423	NM 002423	=	TSS11656	P7018
chr11	Cufflinks	exon	102393971	102394132	-	XLOC 00604	TCONS 0002995	2	MMP7	NM 002423	NM 002423	=	TSS11656	P7018
chr11	Cufflinks	exon	102395667	102395795	-	XLOC 00604	TCONS 0002995	3	MMP7	NM 002423	NM 002423	=	TSS11656	P7018
chr11	Cufflinks	exon	102398255	102398403	-	XLOC 00604	TCONS 0002995	4	MMP7	NM 002423	NM 002423	=	TSS11656	P7018
chr11	Cufflinks	exon	102398488	102398714	-	XLOC 00604	TCONS 0002995	5	MMP7	NM 002423	NM 002423	=	TSS11656	P7018
chr11	Cufflinks	exon	102401324	102401478	-	XLOC 00604	TCONS 0002995	6	MMP7	NM 002423	NM 002423	=	TSS11656	P7018
chr11	Cufflinks	exon	124617370	124617563	-	XLOC 00617	TCONS 0003064	1	VSIG2	NM 014312	NM 014312	=	TSS11909	P7196
chr11	Cufflinks	exon	124618286	124618430	-	XLOC 00617	TCONS 0003064	2	VSIG2	NM 014312	NM 014312	=	TSS11909	P7196
chr11	Cufflinks	exon	124618540	124618659	-	XLOC 00617	TCONS 0003064	3	VSIG2	NM 014312	NM 014312	=	TSS11909	P7196
chr11	Cufflinks	exon	124619604	124619762	-	XLOC 00617	TCONS 0003064	4	VSIG2	NM 014312	NM 014312	=	TSS11909	P7196
chr11	Cufflinks	exon	124620610	124620817	-	XLOC 00617	TCONS 0003064	5	VSIG2	NM 014312	NM 014312	=	TSS11909	P7196
chr11	Cufflinks	exon	124621319	124621476	-	XLOC 00617	TCONS 0003064	6	VSIG2	NM 014312	NM 014312	=	TSS11909	P7196
chr11	Cufflinks	exon	124621973	124622109	-	XLOC 00617	TCONS 0003064	7	VSIG2	NM 014312	NM 014312	=	TSS11909	P7196
chr11	Cufflinks	exon	107992250	107992405	+	XLOC 00525	TCONS 0002582	1	ACAT1	CUFF.7619.1	NM 000019	j	TSS10053	
chr11	Cufflinks	exon	108002634	108002681	+	XLOC 00525	TCONS 0002582	2	ACAT1	CUFF.7619.1	NM 000019	j	TSS10053	
chr11	Cufflinks	exon	108004547	108004664	+	XLOC 00525	TCONS 0002582	3	ACAT1	CUFF.7619.1	NM 000019	j	TSS10053	
chr11	Cufflinks	exon	108004948	108005043	+	XLOC 00525	TCONS 0002582	4	ACAT1	CUFF.7619.1	NM 000019	j	TSS10053	
chr11	Cufflinks	exon	108005869	108005969	+	XLOC 00525	TCONS 0002582	5	ACAT1	CUFF.7619.1	NM 000019	j	TSS10053	
chr11	Cufflinks	exon	108009625	108009768	+	XLOC 00525	TCONS 0002582	6	ACAT1	CUFF.7619.1	NM 000019	j	TSS10053	
chr11	Cufflinks	exon	108010792	108010942	+	XLOC 00525	TCONS 0002582	7	ACAT1	CUFF.7619.1	NM 000019	j	TSS10053	
chr11	Cufflinks	exon	108012332	108012427	+	XLOC 00525	TCONS 0002582	8	ACAT1	CUFF.7619.1	NM 000019	j	TSS10053	
chr11	Cufflinks	exon	108013164	108013277	+	XLOC 00525	TCONS 0002582	9	ACAT1	CUFF.7619.1	NM 000019	j	TSS10053	
chr11	Cufflinks	exon	108014710	108014774	+	XLOC 00525	TCONS 0002582	10	ACAT1	CUFF.7619.1	NM 000019	j	TSS10053	
chr11	Cufflinks	exon	108016929	108017086	+	XLOC 00525	TCONS 0002582	11	ACAT1	CUFF.7619.1	NM 000019	j	TSS10053	
chr11	Cufflinks	exon	108017997	108018185	+	XLOC 00525	TCONS 0002582	12	ACAT1	CUFF.7619.1	NM 000019	j	TSS10053	
chr11	Cufflinks	exon	108019418	108019481	+	XLOC 00525	TCONS 0002582	13	ACAT1	CUFF.7619.1	NM 000019	j	TSS10053	
chr11	Cufflinks	exon	1792673	1792711	-	XLOC 00544	TCONS 0002699	1		CUFF.6053.1		u	TSS10483	
chr11	Cufflinks	exon	1792816	1792918	-	XLOC 00544	TCONS 0002699	2		CUFF.6053.1		u	TSS10483	
chr11	Cufflinks	exon	1798161	1798404	-	XLOC 00544	TCONS 0002699	3		CUFF.6053.1		u	TSS10483	
chr11	Cufflinks	exon	1799755	1799802	-	XLOC 00544	TCONS 0002699	4		CUFF.6053.1		u	TSS10483	
chr12	Cufflinks	exon	69201952	69202271	+	XLOC 00680	TCONS 0003348	1	MDM2	NM 002392	NM 002392	=	TSS13093	P7810
chr12	Cufflinks	exon	69202988	69203072	+	XLOC 00680	TCONS 0003348	2	MDM2	NM 002392	NM 002392	=	TSS13093	P7810
chr12	Cufflinks	exon	69207334	69207408	+	XLOC 00680	TCONS 0003348	3	MDM2	NM 002392	NM 002392	=	TSS13093	P7810
chr12	Cufflinks	exon	69210592	69210725	+	XLOC 00680	TCONS 0003348	4	MDM2	NM 002392	NM 002392	=	TSS13093	P7810
chr12	Cufflinks	exon	69214105	69214154	+	XLOC 00680	TCONS 0003348	5	MDM2	NM 002392	NM 002392	=	TSS13093	P7810
chr12	Cufflinks	exon	69218143	69218210	+	XLOC 00680	TCONS 0003348	6	MDM2	NM 002392	NM 002392	=	TSS13093	P7810
chr12	Cufflinks	exon	69218335	69218431	+	XLOC 00680	TCONS 0003348	7	MDM2	NM 002392	NM 002392	=	TSS13093	P7810
chr12	Cufflinks	exon	69222551	69222711	+	XLOC 00680	TCONS 0003348	8	MDM2	NM 002392	NM 002392	=	TSS13093	P7810
chr12	Cufflinks	exon	69229609	69229764	+	XLOC 00680	TCONS 0003348	9	MDM2	NM 002392	NM 002392	=	TSS13093	P7810
chr12	Cufflinks	exon	69230452	69230529	+	XLOC 00680	TCONS 0003348	10	MDM2	NM 002392	NM 002392	=	TSS13093	P7810
chr12	Cufflinks	exon	69233054	69239324	+	XLOC 00680	TCONS 0003348	11	MDM2	NM 002392	NM 002392	=	TSS13093	P7810
chr12	Cufflinks	exon	121163541	121163734	+	XLOC 00701	TCONS 0003457	1	ACADS	NM 000017	NM 000017	=	TSS13524	P8056
chr12	Cufflinks	exon	121164829	121164992	+	XLOC 00701	TCONS 0003457	2	ACADS	NM 000017	NM 000017	=	TSS13524	P8056
chr12	Cufflinks	exon	121174789	121174938	+	XLOC 00701	TCONS 0003457	3	ACADS	NM 000017	NM 000017	=	TSS13524	P8056
chr12	Cufflinks	exon	121175159	121175270	+	XLOC 00701	TCONS 0003457	4	ACADS	NM 000017	NM 000017	=	TSS13524	P8056
chr12	Cufflinks	exon	121175640	121175791	+	XLOC 00701	TCONS 0003457	5	ACADS	NM 000017	NM 000017	=	TSS13524	P8056
chr12	Cufflinks	exon	121176083	121176253	+	XLOC 00701	TCONS 0003457	6	ACADS	NM 000017	NM 000017	=	TSS13524	P8056
chr12	Cufflinks	exon	121176336	121176473	+	XLOC 00701	TCONS 0003457	7	ACADS	NM 000017	NM 000017	=	TSS13524	P8056
chr12	Cufflinks	exon	121176623	121176718	+	XLOC 00701	TCONS 0003457	8	ACADS	NM 000017	NM 000017	=	TSS13524	P8056
chr12	Cufflinks	exon	121176943	121176999	+	XLOC 00701	TCONS 0003457	9	ACADS	NM 000017	NM 000017	=	TSS13524	P8056
chr12	Cufflinks	exon	121177099	121177811	+	XLOC 00701	TCONS 0003457	10	ACADS	NM 000017	NM 000017	=	TSS13524	P8056
chr12	Cufflinks	exon	133264192	133264378	+	XLOC 00711	TCONS 0003507	1	PXMP2	NM 018663	NM 018663	=	TSS13688	P8141
chr12	Cufflinks	exon	133266849	133266962	+	XLOC 00711	TCONS 0003507	2	PXMP2	NM 018663	NM 018663	=	TSS13688	P8141
chr12	Cufflinks	exon	133272470	133272632	+	XLOC 00711	TCONS 0003507	3	PXMP2	NM 018663	NM 018663	=	TSS13688	P8141
chr12	Cufflinks	exon	133277836	133277955	+	XLOC 00711	TCONS 0003507	4	PXMP2	NM 018663	NM 018663	=	TSS13688	P8141
chr12	Cufflinks	exon	133281205	133281577	+	XLOC 00711	TCONS 0003507	5	PXMP2	NM 018663	NM 018663	=	TSS13688	P8141
chr12	Cufflinks	exon	57422301	57422665	-	XLOC 00748	TCONS 0003732	1	MYO1A	NM 005379	NM 005379	=	TSS14510	P8666
chr12	Cufflinks	exon	57422916	57423043	-	XLOC 00748	TCONS 0003732	2	MYO1A	NM 005379	NM 005379	=	TSS14510	P8666
chr12	Cufflinks	exon	57423219	57423371	-	XLOC 00748	TCONS 0003732	3	MYO1A	NM 005379	NM 005379	=	TSS14510	P8666
chr12	Cufflinks	exon	57423524	57423656	-	XLOC 00748	TCONS 0003732	4	MYO1A	NM 005379	NM 005379	=	TSS14510	P8666
chr12	Cufflinks	exon	57423994	57424100	-	XLOC 00748	TCONS 0003732	5	MYO1A	NM 005379	NM 005379	=	TSS14510	P8666
chr12	Cufflinks	exon	57424824	57424958	-	XLOC 00748	TCONS 0003732	6	MYO1A	NM 005379	NM 005379	=	TSS14510	P8666
chr12	Cufflinks	exon	57430091	57430165	-	XLOC 00748	TCONS 0003732	7	MYO1A	NM 005379	NM 005379	=	TSS14510	P8666
chr12	Cufflinks	exon	57430556	57430624	-	XLOC 00748	TCONS 0003732	8	MYO1A	NM 005379	NM 005379	=	TSS14510	P8666
chr12	Cufflinks	exon	57430726	57430875	-	XLOC 00748	TCONS 0003732	9	MYO1A	NM 005379	NM 005379	=	TSS14510	P8666
chr12	Cufflinks	exon	57431332	57431425	-	XLOC 00748	TCONS 0003732	10	MYO1A	NM 005379	NM 005379	=	TSS14510	P8666
chr12	Cufflinks	exon	57431653	57431853	-	XLOC 00748	TCONS 0003732	11	MYO1A	NM 005379	NM 005379	=	TSS14510	P8666
chr12	Cufflinks	exon	57432196	57432422	-	XLOC 00748	TCONS 0003732	12	MYO1A	NM 005379	NM 005379	=	TSS14510	P8666

chr12	Cufflinks	exon	57432593	57432793	-	XLOC 00748	TCONS 0003732	13	MYO1A	NM 005379	NM 005379	=	TSS14510	P8666
chr12	Cufflinks	exon	57432996	57433058	-	XLOC 00748	TCONS 0003732	14	MYO1A	NM 005379	NM 005379	=	TSS14510	P8666
chr12	Cufflinks	exon	57434968	57435072	-	XLOC 00748	TCONS 0003732	15	MYO1A	NM 005379	NM 005379	=	TSS14510	P8666
chr12	Cufflinks	exon	57435216	57435281	-	XLOC 00748	TCONS 0003732	16	MYO1A	NM 005379	NM 005379	=	TSS14510	P8666
chr12	Cufflinks	exon	57436856	57436942	-	XLOC 00748	TCONS 0003732	17	MYO1A	NM 005379	NM 005379	=	TSS14510	P8666
chr12	Cufflinks	exon	57437024	57437142	-	XLOC 00748	TCONS 0003732	18	MYO1A	NM 005379	NM 005379	=	TSS14510	P8666
chr12	Cufflinks	exon	57437640	57437787	-	XLOC 00748	TCONS 0003732	19	MYO1A	NM 005379	NM 005379	=	TSS14510	P8666
chr12	Cufflinks	exon	57437890	57437993	-	XLOC 00748	TCONS 0003732	20	MYO1A	NM 005379	NM 005379	=	TSS14510	P8666
chr12	Cufflinks	exon	57440336	57440434	-	XLOC 00748	TCONS 0003732	21	MYO1A	NM 005379	NM 005379	=	TSS14510	P8666
chr12	Cufflinks	exon	57440647	57440710	-	XLOC 00748	TCONS 0003732	22	MYO1A	NM 005379	NM 005379	=	TSS14510	P8666
chr12	Cufflinks	exon	57440845	57440891	-	XLOC 00748	TCONS 0003732	23	MYO1A	NM 005379	NM 005379	=	TSS14510	P8666
chr12	Cufflinks	exon	57441087	57441191	-	XLOC 00748	TCONS 0003732	24	MYO1A	NM 005379	NM 005379	=	TSS14510	P8666
chr12	Cufflinks	exon	57441411	57441505	-	XLOC 00748	TCONS 0003732	25	MYO1A	NM 005379	NM 005379	=	TSS14510	P8666
chr12	Cufflinks	exon	57441773	57441888	-	XLOC 00748	TCONS 0003732	26	MYO1A	NM 005379	NM 005379	=	TSS14510	P8666
chr12	Cufflinks	exon	57441994	57442127	-	XLOC 00748	TCONS 0003732	27	MYO1A	NM 005379	NM 005379	=	TSS14510	P8666
chr12	Cufflinks	exon	57443671	57443901	-	XLOC 00748	TCONS 0003732	28	MYO1A	NM 005379	NM 005379	=	TSS14510	P8666
chr12	Cufflinks	exon	63037763	63042416	-	XLOC 00750	TCONS 0003749	1	PPM1H	NM 020700	NM 020700	=	TSS14576	P8700
chr12	Cufflinks	exon	63060958	63061109	-	XLOC 00750	TCONS 0003749	2	PPM1H	NM 020700	NM 020700	=	TSS14576	P8700
chr12	Cufflinks	exon	63083479	63083586	-	XLOC 00750	TCONS 0003749	3	PPM1H	NM 020700	NM 020700	=	TSS14576	P8700
chr12	Cufflinks	exon	63087716	63087779	-	XLOC 00750	TCONS 0003749	4	PPM1H	NM 020700	NM 020700	=	TSS14576	P8700
chr12	Cufflinks	exon	63113951	63114069	-	XLOC 00750	TCONS 0003749	5	PPM1H	NM 020700	NM 020700	=	TSS14576	P8700
chr12	Cufflinks	exon	63131282	63131366	-	XLOC 00750	TCONS 0003749	6	PPM1H	NM 020700	NM 020700	=	TSS14576	P8700
chr12	Cufflinks	exon	63182006	63182118	-	XLOC 00750	TCONS 0003749	7	PPM1H	NM 020700	NM 020700	=	TSS14576	P8700
chr12	Cufflinks	exon	63195596	63195940	-	XLOC 00750	TCONS 0003749	8	PPM1H	NM 020700	NM 020700	=	TSS14576	P8700
chr12	Cufflinks	exon	63225894	63226059	-	XLOC 00750	TCONS 0003749	9	PPM1H	NM 020700	NM 020700	=	TSS14576	P8700
chr12	Cufflinks	exon	63328272	63328665	-	XLOC 00750	TCONS 0003749	10	PPM1H	NM 020700	NM 020700	=	TSS14576	P8700
chr12	Cufflinks	exon	76419227	76423872	-	XLOC 00755	TCONS 0003772	1	PHLDA1	NM 007350	NM 007350	=	TSS14667	P8754
chr12	Cufflinks	exon	76424290	76425556	-	XLOC 00755	TCONS 0003772	2	PHLDA1	NM 007350	NM 007350	=	TSS14667	P8754
chr12	Cufflinks	exon	110288748	110290542	-	XLOC 00767	TCONS 0003834	1	GLTP	NM 016433	NM 016433	=	TSS14915	P8904
chr12	Cufflinks	exon	110293424	110293574	-	XLOC 00767	TCONS 0003834	2	GLTP	NM 016433	NM 016433	=	TSS14915	P8904
chr12	Cufflinks	exon	110295331	110295464	-	XLOC 00767	TCONS 0003834	3	GLTP	NM 016433	NM 016433	=	TSS14915	P8904
chr12	Cufflinks	exon	110296488	110296546	-	XLOC 00767	TCONS 0003834	4	GLTP	NM 016433	NM 016433	=	TSS14915	P8904
chr12	Cufflinks	exon	110318077	110318293	-	XLOC 00767	TCONS 0003834	5	GLTP	NM 016433	NM 016433	=	TSS14915	P8904
chr13	Cufflinks	exon	98628174	98628251	+	XLOC 00830	TCONS 0004055	1	IPO5	CUFF.11199.3	NM 002271	j	TSS15944	
chr13	Cufflinks	exon	98628947	98629040	+	XLOC 00830	TCONS 0004055	2	IPO5	CUFF.11199.3	NM 002271	j	TSS15944	
chr13	Cufflinks	exon	98634757	98634837	+	XLOC 00830	TCONS 0004055	3	IPO5	CUFF.11199.3	NM 002271	j	TSS15944	
chr13	Cufflinks	exon	98637675	98637867	+	XLOC 00830	TCONS 0004055	4	IPO5	CUFF.11199.3	NM 002271	j	TSS15944	
chr13	Cufflinks	exon	98641316	98641418	+	XLOC 00830	TCONS 0004055	5	IPO5	CUFF.11199.3	NM 002271	j	TSS15944	
chr13	Cufflinks	exon	98642380	98642476	+	XLOC 00830	TCONS 0004055	6	IPO5	CUFF.11199.3	NM 002271	j	TSS15944	
chr13	Cufflinks	exon	98642687	98642791	+	XLOC 00830	TCONS 0004055	7	IPO5	CUFF.11199.3	NM 002271	j	TSS15944	
chr13	Cufflinks	exon	98645146	98645268	+	XLOC 00830	TCONS 0004055	8	IPO5	CUFF.11199.3	NM 002271	j	TSS15944	
chr13	Cufflinks	exon	98645359	98645479	+	XLOC 00830	TCONS 0004055	9	IPO5	CUFF.11199.3	NM 002271	j	TSS15944	
chr13	Cufflinks	exon	98649785	98649872	+	XLOC 00830	TCONS 0004055	10	IPO5	CUFF.11199.3	NM 002271	j	TSS15944	
chr13	Cufflinks	exon	98652793	98652899	+	XLOC 00830	TCONS 0004055	11	IPO5	CUFF.11199.3	NM 002271	j	TSS15944	
chr13	Cufflinks	exon	98654721	98654845	+	XLOC 00830	TCONS 0004055	12	IPO5	CUFF.11199.3	NM 002271	j	TSS15944	
chr13	Cufflinks	exon	98654938	98655027	+	XLOC 00830	TCONS 0004055	13	IPO5	CUFF.11199.3	NM 002271	j	TSS15944	
chr13	Cufflinks	exon	98655118	98655291	+	XLOC 00830	TCONS 0004055	14	IPO5	CUFF.11199.3	NM 002271	j	TSS15944	
chr13	Cufflinks	exon	98658384	98658602	+	XLOC 00830	TCONS 0004055	15	IPO5	CUFF.11199.3	NM 002271	j	TSS15944	
chr13	Cufflinks	exon	98660313	98660396	+	XLOC 00830	TCONS 0004055	16	IPO5	CUFF.11199.3	NM 002271	j	TSS15944	
chr13	Cufflinks	exon	98662135	98662267	+	XLOC 00830	TCONS 0004055	17	IPO5	CUFF.11199.3	NM 002271	j	TSS15944	
chr13	Cufflinks	exon	98662357	98662478	+	XLOC 00830	TCONS 0004055	18	IPO5	CUFF.11199.3	NM 002271	j	TSS15944	
chr13	Cufflinks	exon	98664500	98664596	+	XLOC 00830	TCONS 0004055	19	IPO5	CUFF.11199.3	NM 002271	j	TSS15944	
chr13	Cufflinks	exon	98666296	98666468	+	XLOC 00830	TCONS 0004055	20	IPO5	CUFF.11199.3	NM 002271	j	TSS15944	
chr13	Cufflinks	exon	98667784	98667895	+	XLOC 00830	TCONS 0004055	21	IPO5	CUFF.11199.3	NM 002271	j	TSS15944	
chr13	Cufflinks	exon	98667980	98668035	+	XLOC 00830	TCONS 0004055	22	IPO5	CUFF.11199.3	NM 002271	j	TSS15944	
chr13	Cufflinks	exon	98668983	98669105	+	XLOC 00830	TCONS 0004055	23	IPO5	CUFF.11199.3	NM 002271	j	TSS15944	
chr13	Cufflinks	exon	98670739	98670958	+	XLOC 00830	TCONS 0004055	24	IPO5	CUFF.11199.3	NM 002271	j	TSS15944	
chr13	Cufflinks	exon	98671835	98672063	+	XLOC 00830	TCONS 0004055	25	IPO5	CUFF.11199.3	NM 002271	j	TSS15944	
chr13	Cufflinks	exon	98673246	98673387	+	XLOC 00830	TCONS 0004055	26	IPO5	CUFF.11199.3	NM 002271	j	TSS15944	
chr13	Cufflinks	exon	98673990	98676645	+	XLOC 00830	TCONS 0004055	27	IPO5	CUFF.11199.3	NM 002271	j	TSS15944	
chr13	Cufflinks	exon	23269999	23270841	.	XLOC 00871	TCONS 0004239	1		CUFF.10439.1		u	TSS16718	
chr13	Cufflinks	exon	66678844	66680617	.	XLOC 00879	TCONS 0004247	1		CUFF.11004.1		u	TSS16799	
chr14	Cufflinks	exon	93389445	93389750	+	XLOC 00924	TCONS 0004451	1	CHGA	NM 001275	NM 001275	=	TSS17680	P10051
chr14	Cufflinks	exon	93390544	93390590	+	XLOC 00924	TCONS 0004451	2	CHGA	NM 001275	NM 001275	=	TSS17680	P10051
chr14	Cufflinks	exon	93392950	93393043	+	XLOC 00924	TCONS 0004451	3	CHGA	NM 001275	NM 001275	=	TSS17680	P10051
chr14	Cufflinks	exon	93393895	93393963	+	XLOC 00924	TCONS 0004451	4	CHGA	NM 001275	NM 001275	=	TSS17680	P10051
chr14	Cufflinks	exon	93396062	93396160	+	XLOC 00924	TCONS 0004451	5	CHGA	NM 001275	NM 001275	=	TSS17680	P10051
chr14	Cufflinks	exon	93397595	93398047	+	XLOC 00924	TCONS 0004451	6	CHGA	NM 001275	NM 001275	=	TSS17680	P10051
chr14	Cufflinks	exon	93398715	93399196	+	XLOC 00924	TCONS 0004451	7	CHGA	NM 001275	NM 001275	=	TSS17680	P10051
chr14	Cufflinks	exon	93401146	93401638	+	XLOC 00924	TCONS 0004451	8	CHGA	NM 001275	NM 001275	=	TSS17680	P10051
chr14	Cufflinks	exon	24805227	24805601	-	XLOC 00952	TCONS 0004574	1	RIPK3	NM 006871	NM 006871	=	TSS18187	P10284
chr14	Cufflinks	exon	24806091	24806151	-	XLOC 00952	TCONS 0004574	2	RIPK3	NM 006871	NM 006871	=	TSS18187	P10284
chr14	Cufflinks	exon	24806292	24806666	-	XLOC 00952	TCONS 0004574	3	RIPK3	NM 006871	NM 006871	=	TSS18187	P10284
chr14	Cufflinks	exon	24806901	24806968	-	XLOC 00952	TCONS 0004574	4	RIPK3	NM 006871	NM 006871	=	TSS18187	P10284
chr14	Cufflinks	exon	24807079	24807246	-	XLOC 00952	TCONS 0004574	5	RIPK3	NM 006871	NM 006871	=	TSS18187	P10284
chr14	Cufflinks	exon	24807455	24807501	-	XLOC 00952	TCONS 0004574	6	RIPK3	NM 006871	NM 006871	=	TSS18187	P10284
chr14	Cufflinks	exon	24807628	24807773	-	XLOC 00952	TCONS 0004574	7	RIPK3	NM 006871	NM 006871	=	TSS18187	P10284
chr14	Cufflinks	exon	24808221	24808530	-	XLOC 00952	TCONS 0004574	8	RIPK3	NM 006871	NM 006871	=	TSS18187	P10284
chr14	Cufflinks	exon	24808663	24808803	-	XLOC 00952	TCONS 0004574	9	RIPK3	NM 006871	NM 006871	=	TSS18187	P10284
chr14	Cufflinks	exon	24809013	24809242	-	XLOC 00952	TCONS 0004574	10	RIPK3	NM 006871	NM 006871	=	TSS18187	P10284
chr15	Cufflinks	exon	75136020	75137558	-	XLOC 01090	TCONS 0005235	1	SCAMP2	CUFF.13818.1	NM 005697	=	TSS20827	P11549
chr15	Cufflinks	exon	75137814	75137934	-	XLOC 01090	TCONS 0005235	2	SCAMP2	CUFF.13818.1	NM 005697	=	TSS20827	P11549
chr15	Cufflinks	exon	75140941	75141042	-	XLOC 01090	TCONS 0005235	3	SCAMP2	CUFF.13818.1	NM 005697	=	TSS20827	P11549
chr15	Cufflinks	exon	75142855	75143014	-	XLOC 01090	TCONS 0005235	4	SCAMP2	CUFF.13818.1	NM 005697	=	TSS20827	P11549
chr15	Cufflinks	exon	75143694	75143822	-	XLOC 01090	TCONS 0005235	5	SCAMP2	CUFF.13818.1	NM 005697	=	TSS20827	P11549
chr15	Cufflinks	exon	75144410	75144527	-	XLOC 01090	TCONS 0005235	6	SCAMP2	CUFF.13818.1	NM 005697	=	TSS20827	P11549
chr15	Cufflinks	exon	75146362	75146460	-	XLOC 01090	TCONS 0005235	7	SCAMP2	CUFF.13818.1	NM 005697	=	TSS20827	P11549
chr15	Cufflinks	exon	75146922	75146990	-	XLOC 01090	TCONS 0005235	8	SCAMP2	CUFF.13818.1	NM 005697	=	TSS20827	P11549
chr15	Cufflinks	exon	75165540	75165733	-	XLOC 01090	TCONS 0005235	9	SCAMP2	CUFF.13818.1	NM 005697	=	TSS20827	P11549
chr15	Cufflinks	exon	45950461	45950592	+	XLOC 01029	TCONS 0004885	1	SQRDL	CUFF.13352.7	NM 001271213	j	TSS19537	
chr15	Cufflinks	exon	45951105	45951355	+	XLOC 01029	TCONS 0004885	2	SQRDL	CUFF.13352.7	NM 001271213	j	TSS19537	
chr15	Cufflinks	exon	45954153	45954323	+	XLOC 01029	TCONS 0004885	3	SQRDL	CUFF.13352.7	NM 001271213	j	TSS19537	
chr15	Cufflinks	exon	45962126	45962179	+	XLOC 01029	TCONS 0004885	4	SQRDL	CUFF.13352.7	NM 001271213	j	TSS19537	
chr15	Cufflinks	exon	45965805	45965999	+	XLOC 01029	TCONS 0004885	5	SQRDL	CUFF.13352.7	NM 001271213	j	TSS19537	
chr15	Cufflinks	exon	45968299	45968508	+	XLOC 01029	TCONS 0004885	6	SQRDL	CUFF.13352.7	NM 001271213	j	TSS19537	
chr15	Cufflinks	exon	45974676	45974859	+	XLOC 01029	TCONS 0004885	7	SQRDL	CUFF.13352.7	NM 001271213	j	TSS19537	
chr15	Cufflinks	exon	45980535	45980602	+	XLOC 01029	TCONS 0004885	8	SQRDL	CUFF.13352.7	NM 001271213	j	TSS19537	
chr15	Cufflinks	exon	45981237	45981415	+	XLOC 01029	TCONS 0004885	9	SQRDL	CUFF.13352.7	NM 001271213	j	TSS19537	
chr15	Cufflinks	exon	45983171	45983494	+	XLOC 01029	TCONS 0004885	10	SQRDL	CUFF.13352.7	NM 001271213	j	TSS19537	
chr15	Cufflinks	exon	98507249	98507311	+	XLOC 01057	TCONS 0005040	1	ARRDC4	CUFF.14206.6	NM 183376	j	TSS20145	

chr15	Cufflinks	exon	98508830	98508896	+	XLOC 01057	TCONS 0005040	2	ARRDC4	CUFF.14206.6	NM 183376	j	TSS20145	
chr15	Cufflinks	exon	98509125	98509272	+	XLOC 01057	TCONS 0005040	3	ARRDC4	CUFF.14206.6	NM 183376	j	TSS20145	
chr15	Cufflinks	exon	98511244	98511346	+	XLOC 01057	TCONS 0005040	4	ARRDC4	CUFF.14206.6	NM 183376	j	TSS20145	
chr15	Cufflinks	exon	98512353	98512609	+	XLOC 01057	TCONS 0005040	5	ARRDC4	CUFF.14206.6	NM 183376	j	TSS20145	
chr15	Cufflinks	exon	98513113	98513275	+	XLOC 01057	TCONS 0005040	6	ARRDC4	CUFF.14206.6	NM 183376	j	TSS20145	
chr15	Cufflinks	exon	98513819	98513973	+	XLOC 01057	TCONS 0005040	7	ARRDC4	CUFF.14206.6	NM 183376	j	TSS20145	
chr15	Cufflinks	exon	98514361	98517072	+	XLOC 01057	TCONS 0005040	8	ARRDC4	CUFF.14206.6	NM 183376	j	TSS20145	
chr16	Cufflinks	exon	28505970	28506059	+	XLOC 01143	TCONS 0005465	1	APOBR	NM 018690	NM 018690	=	TSS21781	P12013
chr16	Cufflinks	exon	28506420	28509317	+	XLOC 01143	TCONS 0005465	2	APOBR	NM 018690	NM 018690	=	TSS21781	P12013
chr16	Cufflinks	exon	28509402	28509670	+	XLOC 01143	TCONS 0005465	3	APOBR	NM 018690	NM 018690	=	TSS21781	P12013
chr16	Cufflinks	exon	28509757	28510291	+	XLOC 01143	TCONS 0005465	4	APOBR	NM 018690	NM 018690	=	TSS21781	P12013
chr16	Cufflinks	exon	66995132	66995292	+	XLOC 01160	TCONS 0005567	1	CES3	NM 024922	NM 024922	=	TSS22171	P12271
chr16	Cufflinks	exon	66997082	66997286	+	XLOC 01160	TCONS 0005567	2	CES3	NM 024922	NM 024922	=	TSS22171	P12271
chr16	Cufflinks	exon	66997394	66997532	+	XLOC 01160	TCONS 0005567	3	CES3	NM 024922	NM 024922	=	TSS22171	P12271
chr16	Cufflinks	exon	66997705	66997838	+	XLOC 01160	TCONS 0005567	4	CES3	NM 024922	NM 024922	=	TSS22171	P12271
chr16	Cufflinks	exon	66998260	66998413	+	XLOC 01160	TCONS 0005567	5	CES3	NM 024922	NM 024922	=	TSS22171	P12271
chr16	Cufflinks	exon	66998526	66998630	+	XLOC 01160	TCONS 0005567	6	CES3	NM 024922	NM 024922	=	TSS22171	P12271
chr16	Cufflinks	exon	67000147	67000248	+	XLOC 01160	TCONS 0005567	7	CES3	NM 024922	NM 024922	=	TSS22171	P12271
chr16	Cufflinks	exon	67000628	67000768	+	XLOC 01160	TCONS 0005567	8	CES3	NM 024922	NM 024922	=	TSS22171	P12271
chr16	Cufflinks	exon	67003582	67003662	+	XLOC 01160	TCONS 0005567	9	CES3	NM 024922	NM 024922	=	TSS22171	P12271
chr16	Cufflinks	exon	67005075	67005222	+	XLOC 01160	TCONS 0005567	10	CES3	NM 024922	NM 024922	=	TSS22171	P12271
chr16	Cufflinks	exon	67006259	67006408	+	XLOC 01160	TCONS 0005567	11	CES3	NM 024922	NM 024922	=	TSS22171	P12271
chr16	Cufflinks	exon	67006571	67006649	+	XLOC 01160	TCONS 0005567	12	CES3	NM 024922	NM 024922	=	TSS22171	P12271
chr16	Cufflinks	exon	67006757	67009052	+	XLOC 01160	TCONS 0005567	13	CES3	NM 024922	NM 024922	=	TSS22171	P12271
chr16	Cufflinks	exon	82068858	82069294	+	XLOC 01170	TCONS 0005632	1	HSD17B2	NM 002153	NM 002153	=	TSS22401	P12411
chr16	Cufflinks	exon	82101775	82101987	+	XLOC 01170	TCONS 0005632	2	HSD17B2	NM 002153	NM 002153	=	TSS22401	P12411
chr16	Cufflinks	exon	82104547	82104732	+	XLOC 01170	TCONS 0005632	3	HSD17B2	NM 002153	NM 002153	=	TSS22401	P12411
chr16	Cufflinks	exon	82124507	82124644	+	XLOC 01170	TCONS 0005632	4	HSD17B2	NM 002153	NM 002153	=	TSS22401	P12411
chr16	Cufflinks	exon	82131680	82132139	+	XLOC 01170	TCONS 0005632	5	HSD17B2	NM 002153	NM 002153	=	TSS22401	P12411
chr16	Cufflinks	exon	89680054	89680127	+	XLOC 01177	TCONS 0005663	1	DPEP1	CUFF.15817.7	NM 004413	j	TSS22537	
chr16	Cufflinks	exon	89696709	89696922	+	XLOC 01177	TCONS 0005663	2	DPEP1	CUFF.15817.7	NM 004413	j	TSS22537	
chr16	Cufflinks	exon	89702316	89702448	+	XLOC 01177	TCONS 0005663	3	DPEP1	CUFF.15817.7	NM 004413	j	TSS22537	
chr16	Cufflinks	exon	89702672	89702804	+	XLOC 01177	TCONS 0005663	4	DPEP1	CUFF.15817.7	NM 004413	j	TSS22537	
chr16	Cufflinks	exon	89702941	89703091	+	XLOC 01177	TCONS 0005663	5	DPEP1	CUFF.15817.7	NM 004413	j	TSS22537	
chr16	Cufflinks	exon	89703274	89703343	+	XLOC 01177	TCONS 0005663	6	DPEP1	CUFF.15817.7	NM 004413	j	TSS22537	
chr16	Cufflinks	exon	89703612	89703788	+	XLOC 01177	TCONS 0005663	7	DPEP1	CUFF.15817.7	NM 004413	j	TSS22537	
chr16	Cufflinks	exon	89703876	89703960	+	XLOC 01177	TCONS 0005663	8	DPEP1	CUFF.15817.7	NM 004413	j	TSS22537	
chr16	Cufflinks	exon	89704040	89704115	+	XLOC 01177	TCONS 0005663	9	DPEP1	CUFF.15817.7	NM 004413	j	TSS22537	
chr16	Cufflinks	exon	89704244	89704379	+	XLOC 01177	TCONS 0005663	10	DPEP1	CUFF.15817.7	NM 004413	j	TSS22537	
chr16	Cufflinks	exon	89704460	89704839	+	XLOC 01177	TCONS 0005663	11	DPEP1	CUFF.15817.7	NM 004413	j	TSS22537	
chr16	Cufflinks	exon	71678603	71683947	-	XLOC 01220	TCONS 0005896	1	PHLPP2	CUFF.15508.7	NM 015020	j	TSS23428	
chr16	Cufflinks	exon	71686693	71686924	-	XLOC 01220	TCONS 0005896	2	PHLPP2	CUFF.15508.7	NM 015020	j	TSS23428	
chr16	Cufflinks	exon	71689143	71689337	-	XLOC 01220	TCONS 0005896	3	PHLPP2	CUFF.15508.7	NM 015020	j	TSS23428	
chr16	Cufflinks	exon	71690474	71690584	-	XLOC 01220	TCONS 0005896	4	PHLPP2	CUFF.15508.7	NM 015020	j	TSS23428	
chr16	Cufflinks	exon	71692136	71692266	-	XLOC 01220	TCONS 0005896	5	PHLPP2	CUFF.15508.7	NM 015020	j	TSS23428	
chr16	Cufflinks	exon	71692556	71692718	-	XLOC 01220	TCONS 0005896	6	PHLPP2	CUFF.15508.7	NM 015020	j	TSS23428	
chr16	Cufflinks	exon	71697802	71698002	-	XLOC 01220	TCONS 0005896	7	PHLPP2	CUFF.15508.7	NM 015020	j	TSS23428	
chr16	Cufflinks	exon	71699914	71699984	-	XLOC 01220	TCONS 0005896	8	PHLPP2	CUFF.15508.7	NM 015020	j	TSS23428	
chr16	Cufflinks	exon	87863624	87866631	-	XLOC 01227	TCONS 0005941	1	SLC7A5	CUFF.15748.1	NM 003486	j	TSS23595	
chr16	Cufflinks	exon	87868020	87868197	-	XLOC 01227	TCONS 0005941	2	SLC7A5	CUFF.15748.1	NM 003486	j	TSS23595	
chr16	Cufflinks	exon	87870104	87870253	-	XLOC 01227	TCONS 0005941	3	SLC7A5	CUFF.15748.1	NM 003486	j	TSS23595	
chr16	Cufflinks	exon	87871451	87871547	-	XLOC 01227	TCONS 0005941	4	SLC7A5	CUFF.15748.1	NM 003486	j	TSS23595	
chr16	Cufflinks	exon	87872320	87872423	-	XLOC 01227	TCONS 0005941	5	SLC7A5	CUFF.15748.1	NM 003486	j	TSS23595	
chr16	Cufflinks	exon	87873308	87873431	-	XLOC 01227	TCONS 0005941	6	SLC7A5	CUFF.15748.1	NM 003486	j	TSS23595	
chr16	Cufflinks	exon	87874035	87874079	-	XLOC 01227	TCONS 0005941	7	SLC7A5	CUFF.15748.1	NM 003486	j	TSS23595	
chr16	Cufflinks	exon	87874656	87874761	-	XLOC 01227	TCONS 0005941	8	SLC7A5	CUFF.15748.1	NM 003486	j	TSS23595	
chr16	Cufflinks	exon	87885330	87885455	-	XLOC 01227	TCONS 0005941	9	SLC7A5	CUFF.15748.1	NM 003486	j	TSS23595	
chr16	Cufflinks	exon	87887072	87887156	-	XLOC 01227	TCONS 0005941	10	SLC7A5	CUFF.15748.1	NM 003486	j	TSS23595	
chr17	Cufflinks	exon	72744752	72744838	+	XLOC 01304	TCONS 0006284	1	MIR3615	NR 037409	NR 037409	=	TSS25033	
chr17	Cufflinks	exon	34948226	34948586	+	XLOC 01272	TCONS 0006123	1	DHRS11	NM 024308	NM 024308	=	TSS24382	P13478
chr17	Cufflinks	exon	34951401	34951610	+	XLOC 01272	TCONS 0006123	2	DHRS11	NM 024308	NM 024308	=	TSS24382	P13478
chr17	Cufflinks	exon	34954592	34954686	+	XLOC 01272	TCONS 0006123	3	DHRS11	NM 024308	NM 024308	=	TSS24382	P13478
chr17	Cufflinks	exon	34955350	34955479	+	XLOC 01272	TCONS 0006123	4	DHRS11	NM 024308	NM 024308	=	TSS24382	P13478
chr17	Cufflinks	exon	34956100	34956192	+	XLOC 01272	TCONS 0006123	5	DHRS11	NM 024308	NM 024308	=	TSS24382	P13478
chr17	Cufflinks	exon	34956401	34956466	+	XLOC 01272	TCONS 0006123	6	DHRS11	NM 024308	NM 024308	=	TSS24382	P13478
chr17	Cufflinks	exon	34956591	34957233	+	XLOC 01272	TCONS 0006123	7	DHRS11	NM 024308	NM 024308	=	TSS24382	P13478
chr17	Cufflinks	exon	38333263	38333375	+	XLOC 01276	TCONS 0006142	1	RAPGEFL1	NM 001303533	NM 001303533	=	TSS24472	P13527
chr17	Cufflinks	exon	38337868	38337946	+	XLOC 01276	TCONS 0006142	2	RAPGEFL1	NM 001303533	NM 001303533	=	TSS24472	P13527
chr17	Cufflinks	exon	38340466	38340601	+	XLOC 01276	TCONS 0006142	3	RAPGEFL1	NM 001303533	NM 001303533	=	TSS24472	P13527
chr17	Cufflinks	exon	38340833	38340930	+	XLOC 01276	TCONS 0006142	4	RAPGEFL1	NM 001303533	NM 001303533	=	TSS24472	P13527
chr17	Cufflinks	exon	38345118	38345230	+	XLOC 01276	TCONS 0006142	5	RAPGEFL1	NM 001303533	NM 001303533	=	TSS24472	P13527
chr17	Cufflinks	exon	38345460	38345627	+	XLOC 01276	TCONS 0006142	6	RAPGEFL1	NM 001303533	NM 001303533	=	TSS24472	P13527
chr17	Cufflinks	exon	38346686	38346783	+	XLOC 01276	TCONS 0006142	7	RAPGEFL1	NM 001303533	NM 001303533	=	TSS24472	P13527
chr17	Cufflinks	exon	38346892	38347014	+	XLOC 01276	TCONS 0006142	8	RAPGEFL1	NM 001303533	NM 001303533	=	TSS24472	P13527
chr17	Cufflinks	exon	38347568	38347746	+	XLOC 01276	TCONS 0006142	9	RAPGEFL1	NM 001303533	NM 001303533	=	TSS24472	P13527
chr17	Cufflinks	exon	38347834	38347924	+	XLOC 01276	TCONS 0006142	10	RAPGEFL1	NM 001303533	NM 001303533	=	TSS24472	P13527
chr17	Cufflinks	exon	38348465	38348515	+	XLOC 01276	TCONS 0006142	11	RAPGEFL1	NM 001303533	NM 001303533	=	TSS24472	P13527
chr17	Cufflinks	exon	38348858	38348945	+	XLOC 01276	TCONS 0006142	12	RAPGEFL1	NM 001303533	NM 001303533	=	TSS24472	P13527
chr17	Cufflinks	exon	38349178	38349242	+	XLOC 01276	TCONS 0006142	13	RAPGEFL1	NM 001303533	NM 001303533	=	TSS24472	P13527
chr17	Cufflinks	exon	38349615	38349669	+	XLOC 01276	TCONS 0006142	14	RAPGEFL1	NM 001303533	NM 001303533	=	TSS24472	P13527
chr17	Cufflinks	exon	38349916	38351908	+	XLOC 01276	TCONS 0006142	15	RAPGEFL1	NM 001303533	NM 001303533	=	TSS24472	P13527
chr17	Cufflinks	exon	48503519	48503750	+	XLOC 01291	TCONS 0006218	1	ACSF2	NM 025149	NM 025149	=	TSS24770	P13717
chr17	Cufflinks	exon	48538038	48538233	+	XLOC 01291	TCONS 0006218	2	ACSF2	NM 025149	NM 025149	=	TSS24770	P13717
chr17	Cufflinks	exon	48538603	48538731	+	XLOC 01291	TCONS 0006218	3	ACSF2	NM 025149	NM 025149	=	TSS24770	P13717
chr17	Cufflinks	exon	48538994	48539047	+	XLOC 01291	TCONS 0006218	4	ACSF2	NM 025149	NM 025149	=	TSS24770	P13717
chr17	Cufflinks	exon	48539545	48539663	+	XLOC 01291	TCONS 0006218	5	ACSF2	NM 025149	NM 025149	=	TSS24770	P13717
chr17	Cufflinks	exon	48539781	48539946	+	XLOC 01291	TCONS 0006218	6	ACSF2	NM 025149	NM 025149	=	TSS24770	P13717
chr17	Cufflinks	exon	48540517	48540612	+	XLOC 01291	TCONS 0006218	7	ACSF2	NM 025149	NM 025149	=	TSS24770	P13717
chr17	Cufflinks	exon	48540756	48540913	+	XLOC 01291	TCONS 0006218	8	ACSF2	NM 025149	NM 025149	=	TSS24770	P13717
chr17	Cufflinks	exon	48541179	48541270	+	XLOC 01291	TCONS 0006218	9	ACSF2	NM 025149	NM 025149	=	TSS24770	P13717
chr17	Cufflinks	exon	48541579	48541655	+	XLOC 01291	TCONS 0006218	10	ACSF2	NM 025149	NM 025149	=	TSS24770	P13717
chr17	Cufflinks	exon	48548389	48548496	+	XLOC 01291	TCONS 0006218	11	ACSF2	NM 025149	NM 025149	=	TSS24770	P13717
chr17	Cufflinks	exon	48549789	48549940	+	XLOC 01291	TCONS 0006218	12	ACSF2	NM 025149	NM 025149	=	TSS24770	P13717
chr17	Cufflinks	exon	48551026	48551167	+	XLOC 01291	TCONS 0006218	13	ACSF2	NM 025149	NM 025149	=	TSS24770	P13717
chr17	Cufflinks	exon	48551255	48551365	+	XLOC 01291	TCONS 0006218	14	ACSF2	NM 025149	NM 025149	=	TSS24770	P13717
chr17	Cufflinks	exon	48551560	48551628	+	XLOC 01291	TCONS 0006218	15	ACSF2	NM 025149	NM 025149	=	TSS24770	P13717
chr17	Cufflinks	exon	48551863	48552206	+	XLOC 01291	TCONS 0006218	16	ACSF2	NM 025149	NM 025149	=	TSS24770	P13717
chr17	Cufflinks	exon	58227302	58227453	+	XLOC 01296	TCONS 0006244	1	CA4	NM 000717	NM 000717	=	TSS24870	P13801
chr17	Cufflinks	exon	58232675	58232728	+	XLOC 01296	TCONS 0006244	2	CA4	NM 000717	NM 000717	=	TSS24870	P13801
chr17	Cufflinks	exon	58233921	58234076	+	XLOC 01296	TCONS 0006244	3	CA4	NM 000717	NM 000717	=	TSS24870	P13801

chr17	Cufflinks	exon	58234788	58234933	+	XLOC 01296	TCONS 0006244	4	CA4	NM 000717	NM 000717	=	TSS24870	P13801
chr17	Cufflinks	exon	58235051	58235149	+	XLOC 01296	TCONS 0006244	5	CA4	NM 000717	NM 000717	=	TSS24870	P13801
chr17	Cufflinks	exon	58235422	58235488	+	XLOC 01296	TCONS 0006244	6	CA4	NM 000717	NM 000717	=	TSS24870	P13801
chr17	Cufflinks	exon	58235644	58235807	+	XLOC 01296	TCONS 0006244	7	CA4	NM 000717	NM 000717	=	TSS24870	P13801
chr17	Cufflinks	exon	58236591	58236906	+	XLOC 01296	TCONS 0006244	8	CA4	NM 000717	NM 000717	=	TSS24870	P13801
chr17	Cufflinks	exon	70117161	70117963	+	XLOC 01303	TCONS 0006275	1	SOX9	NM 000346	NM 000346	=	TSS24991	P13860
chr17	Cufflinks	exon	70118860	70119113	+	XLOC 01303	TCONS 0006275	2	SOX9	NM 000346	NM 000346	=	TSS24991	P13860
chr17	Cufflinks	exon	70119684	70122560	+	XLOC 01303	TCONS 0006275	3	SOX9	NM 000346	NM 000346	=	TSS24991	P13860
chr17	Cufflinks	exon	72744751	72745426	+	XLOC 01304	TCONS 0006284	1	SLC9A3R1	NM 004252	NM 004252	=	TSS25033	P13884
chr17	Cufflinks	exon	72758151	72758312	+	XLOC 01304	TCONS 0006284	2	SLC9A3R1	NM 004252	NM 004252	=	TSS25033	P13884
chr17	Cufflinks	exon	72759506	72759659	+	XLOC 01304	TCONS 0006284	3	SLC9A3R1	NM 004252	NM 004252	=	TSS25033	P13884
chr17	Cufflinks	exon	72763075	72763115	+	XLOC 01304	TCONS 0006284	4	SLC9A3R1	NM 004252	NM 004252	=	TSS25033	P13884
chr17	Cufflinks	exon	72764286	72764375	+	XLOC 01304	TCONS 0006284	5	SLC9A3R1	NM 004252	NM 004252	=	TSS25033	P13884
chr17	Cufflinks	exon	72764607	72765499	+	XLOC 01304	TCONS 0006284	6	SLC9A3R1	NM 004252	NM 004252	=	TSS25033	P13884
chr17	Cufflinks	exon	30819540	30821933	-	XLOC 01343	TCONS 0006517	1	MYO1D	NM 015194	NM 015194	=	TSS25895	P14393
chr17	Cufflinks	exon	30932105	30932259	-	XLOC 01343	TCONS 0006517	2	MYO1D	NM 015194	NM 015194	=	TSS25895	P14393
chr17	Cufflinks	exon	30965740	30965853	-	XLOC 01343	TCONS 0006517	3	MYO1D	NM 015194	NM 015194	=	TSS25895	P14393
chr17	Cufflinks	exon	30980861	30980965	-	XLOC 01343	TCONS 0006517	4	MYO1D	NM 015194	NM 015194	=	TSS25895	P14393
chr17	Cufflinks	exon	30981495	30981639	-	XLOC 01343	TCONS 0006517	5	MYO1D	NM 015194	NM 015194	=	TSS25895	P14393
chr17	Cufflinks	exon	30986133	30986356	-	XLOC 01343	TCONS 0006517	6	MYO1D	NM 015194	NM 015194	=	TSS25895	P14393
chr17	Cufflinks	exon	31039006	31039213	-	XLOC 01343	TCONS 0006517	7	MYO1D	NM 015194	NM 015194	=	TSS25895	P14393
chr17	Cufflinks	exon	31048041	31048207	-	XLOC 01343	TCONS 0006517	8	MYO1D	NM 015194	NM 015194	=	TSS25895	P14393
chr17	Cufflinks	exon	31065271	31065403	-	XLOC 01343	TCONS 0006517	9	MYO1D	NM 015194	NM 015194	=	TSS25895	P14393
chr17	Cufflinks	exon	31072229	31072303	-	XLOC 01343	TCONS 0006517	10	MYO1D	NM 015194	NM 015194	=	TSS25895	P14393
chr17	Cufflinks	exon	31075954	31076024	-	XLOC 01343	TCONS 0006517	11	MYO1D	NM 015194	NM 015194	=	TSS25895	P14393
chr17	Cufflinks	exon	31082510	31082680	-	XLOC 01343	TCONS 0006517	12	MYO1D	NM 015194	NM 015194	=	TSS25895	P14393
chr17	Cufflinks	exon	31087308	31087422	-	XLOC 01343	TCONS 0006517	13	MYO1D	NM 015194	NM 015194	=	TSS25895	P14393
chr17	Cufflinks	exon	31087500	31087645	-	XLOC 01343	TCONS 0006517	14	MYO1D	NM 015194	NM 015194	=	TSS25895	P14393
chr17	Cufflinks	exon	31091896	31092099	-	XLOC 01343	TCONS 0006517	15	MYO1D	NM 015194	NM 015194	=	TSS25895	P14393
chr17	Cufflinks	exon	31094654	31094770	-	XLOC 01343	TCONS 0006517	16	MYO1D	NM 015194	NM 015194	=	TSS25895	P14393
chr17	Cufflinks	exon	31098143	31098238	-	XLOC 01343	TCONS 0006517	17	MYO1D	NM 015194	NM 015194	=	TSS25895	P14393
chr17	Cufflinks	exon	31099807	31099860	-	XLOC 01343	TCONS 0006517	18	MYO1D	NM 015194	NM 015194	=	TSS25895	P14393
chr17	Cufflinks	exon	31102882	31103047	-	XLOC 01343	TCONS 0006517	19	MYO1D	NM 015194	NM 015194	=	TSS25895	P14393
chr17	Cufflinks	exon	31105498	31105591	-	XLOC 01343	TCONS 0006517	20	MYO1D	NM 015194	NM 015194	=	TSS25895	P14393
chr17	Cufflinks	exon	31107594	31107802	-	XLOC 01343	TCONS 0006517	21	MYO1D	NM 015194	NM 015194	=	TSS25895	P14393
chr17	Cufflinks	exon	31203796	31204191	-	XLOC 01343	TCONS 0006517	22	MYO1D	NM 015194	NM 015194	=	TSS25895	P14393
chr17	Cufflinks	exon	45884733	45885765	-	XLOC 01365	TCONS 0006632	1	OSBPL7	NM 145798	NM 145798	=	TSS26311	P14691
chr17	Cufflinks	exon	45885904	45886026	-	XLOC 01365	TCONS 0006632	2	OSBPL7	NM 145798	NM 145798	=	TSS26311	P14691
chr17	Cufflinks	exon	45886230	45886356	-	XLOC 01365	TCONS 0006632	3	OSBPL7	NM 145798	NM 145798	=	TSS26311	P14691
chr17	Cufflinks	exon	45886442	45886586	-	XLOC 01365	TCONS 0006632	4	OSBPL7	NM 145798	NM 145798	=	TSS26311	P14691
chr17	Cufflinks	exon	45886700	45886844	-	XLOC 01365	TCONS 0006632	5	OSBPL7	NM 145798	NM 145798	=	TSS26311	P14691
chr17	Cufflinks	exon	45887960	45888038	-	XLOC 01365	TCONS 0006632	6	OSBPL7	NM 145798	NM 145798	=	TSS26311	P14691
chr17	Cufflinks	exon	45888138	45888201	-	XLOC 01365	TCONS 0006632	7	OSBPL7	NM 145798	NM 145798	=	TSS26311	P14691
chr17	Cufflinks	exon	45890632	45890769	-	XLOC 01365	TCONS 0006632	8	OSBPL7	NM 145798	NM 145798	=	TSS26311	P14691
chr17	Cufflinks	exon	45890953	45891200	-	XLOC 01365	TCONS 0006632	9	OSBPL7	NM 145798	NM 145798	=	TSS26311	P14691
chr17	Cufflinks	exon	45891887	45891980	-	XLOC 01365	TCONS 0006632	10	OSBPL7	NM 145798	NM 145798	=	TSS26311	P14691
chr17	Cufflinks	exon	45892581	45892718	-	XLOC 01365	TCONS 0006632	11	OSBPL7	NM 145798	NM 145798	=	TSS26311	P14691
chr17	Cufflinks	exon	45893473	45893568	-	XLOC 01365	TCONS 0006632	12	OSBPL7	NM 145798	NM 145798	=	TSS26311	P14691
chr17	Cufflinks	exon	45893754	45893848	-	XLOC 01365	TCONS 0006632	13	OSBPL7	NM 145798	NM 145798	=	TSS26311	P14691
chr17	Cufflinks	exon	45893929	45894061	-	XLOC 01365	TCONS 0006632	14	OSBPL7	NM 145798	NM 145798	=	TSS26311	P14691
chr17	Cufflinks	exon	45894146	45894238	-	XLOC 01365	TCONS 0006632	15	OSBPL7	NM 145798	NM 145798	=	TSS26311	P14691
chr17	Cufflinks	exon	45894622	45894725	-	XLOC 01365	TCONS 0006632	16	OSBPL7	NM 145798	NM 145798	=	TSS26311	P14691
chr17	Cufflinks	exon	45895635	45895752	-	XLOC 01365	TCONS 0006632	17	OSBPL7	NM 145798	NM 145798	=	TSS26311	P14691
chr17	Cufflinks	exon	45895872	45895982	-	XLOC 01365	TCONS 0006632	18	OSBPL7	NM 145798	NM 145798	=	TSS26311	P14691
chr17	Cufflinks	exon	45896352	45896465	-	XLOC 01365	TCONS 0006632	19	OSBPL7	NM 145798	NM 145798	=	TSS26311	P14691
chr17	Cufflinks	exon	45897095	45897148	-	XLOC 01365	TCONS 0006632	20	OSBPL7	NM 145798	NM 145798	=	TSS26311	P14691
chr17	Cufflinks	exon	45897337	45897462	-	XLOC 01365	TCONS 0006632	21	OSBPL7	NM 145798	NM 145798	=	TSS26311	P14691
chr17	Cufflinks	exon	45897570	45897731	-	XLOC 01365	TCONS 0006632	22	OSBPL7	NM 145798	NM 145798	=	TSS26311	P14691
chr17	Cufflinks	exon	45899032	45899147	-	XLOC 01365	TCONS 0006632	23	OSBPL7	NM 145798	NM 145798	=	TSS26311	P14691
chr17	Cufflinks	exon	73937589	73942876	-	XLOC 01386	TCONS 0006760	1	ACOX1	NM 004035	NM 004035	=	TSS26792	P14954
chr17	Cufflinks	exon	73944332	73944538	-	XLOC 01386	TCONS 0006760	2	ACOX1	NM 004035	NM 004035	=	TSS26792	P14954
chr17	Cufflinks	exon	73945298	73945441	-	XLOC 01386	TCONS 0006760	3	ACOX1	NM 004035	NM 004035	=	TSS26792	P14954
chr17	Cufflinks	exon	73945576	73945681	-	XLOC 01386	TCONS 0006760	4	ACOX1	NM 004035	NM 004035	=	TSS26792	P14954
chr17	Cufflinks	exon	73945799	73945978	-	XLOC 01386	TCONS 0006760	5	ACOX1	NM 004035	NM 004035	=	TSS26792	P14954
chr17	Cufflinks	exon	73946855	73947045	-	XLOC 01386	TCONS 0006760	6	ACOX1	NM 004035	NM 004035	=	TSS26792	P14954
chr17	Cufflinks	exon	73947496	73947658	-	XLOC 01386	TCONS 0006760	7	ACOX1	NM 004035	NM 004035	=	TSS26792	P14954
chr17	Cufflinks	exon	73949532	73949701	-	XLOC 01386	TCONS 0006760	8	ACOX1	NM 004035	NM 004035	=	TSS26792	P14954
chr17	Cufflinks	exon	73951647	73951762	-	XLOC 01386	TCONS 0006760	9	ACOX1	NM 004035	NM 004035	=	TSS26792	P14954
chr17	Cufflinks	exon	73951909	73952028	-	XLOC 01386	TCONS 0006760	10	ACOX1	NM 004035	NM 004035	=	TSS26792	P14954
chr17	Cufflinks	exon	73953540	73953647	-	XLOC 01386	TCONS 0006760	11	ACOX1	NM 004035	NM 004035	=	TSS26792	P14954
chr17	Cufflinks	exon	73956296	73956456	-	XLOC 01386	TCONS 0006760	12	ACOX1	NM 004035	NM 004035	=	TSS26792	P14954
chr17	Cufflinks	exon	73974615	73974774	-	XLOC 01386	TCONS 0006760	13	ACOX1	NM 004035	NM 004035	=	TSS26792	P14954
chr17	Cufflinks	exon	73975046	73975515	-	XLOC 01386	TCONS 0006760	14	ACOX1	NM 004035	NM 004035	=	TSS26792	P14954
chr17	Cufflinks	exon	3907497	3910264	-	XLOC 01321	TCONS 0006391	1	ZZEF1	CUFF.15972.2	NM 015113	j	TSS25433	
chr17	Cufflinks	exon	3912177	3912251	-	XLOC 01321	TCONS 0006391	2	ZZEF1	CUFF.15972.2	NM 015113	j	TSS25433	
chr17	Cufflinks	exon	3912898	3913051	-	XLOC 01321	TCONS 0006391	3	ZZEF1	CUFF.15972.2	NM 015113	j	TSS25433	
chr17	Cufflinks	exon	3916743	3916908	-	XLOC 01321	TCONS 0006391	4	ZZEF1	CUFF.15972.2	NM 015113	j	TSS25433	
chr17	Cufflinks	exon	3917384	3917482	-	XLOC 01321	TCONS 0006391	5	ZZEF1	CUFF.15972.2	NM 015113	j	TSS25433	
chr17	Cufflinks	exon	3917641	3917809	-	XLOC 01321	TCONS 0006391	6	ZZEF1	CUFF.15972.2	NM 015113	j	TSS25433	
chr17	Cufflinks	exon	3919617	3919760	-	XLOC 01321	TCONS 0006391	7	ZZEF1	CUFF.15972.2	NM 015113	j	TSS25433	
chr17	Cufflinks	exon	3920665	3921024	-	XLOC 01321	TCONS 0006391	8	ZZEF1	CUFF.15972.2	NM 015113	j	TSS25433	
chr17	Cufflinks	exon	3921130	3921265	-	XLOC 01321	TCONS 0006391	9	ZZEF1	CUFF.15972.2	NM 015113	j	TSS25433	
chr17	Cufflinks	exon	3922963	3923063	-	XLOC 01321	TCONS 0006391	10	ZZEF1	CUFF.15972.2	NM 015113	j	TSS25433	
chr17	Cufflinks	exon	3924423	3924614	-	XLOC 01321	TCONS 0006391	11	ZZEF1	CUFF.15972.2	NM 015113	j	TSS25433	
chr17	Cufflinks	exon	3926003	3926122	-	XLOC 01321	TCONS 0006391	12	ZZEF1	CUFF.15972.2	NM 015113	j	TSS25433	
chr17	Cufflinks	exon	3928213	3928412	-	XLOC 01321	TCONS 0006391	13	ZZEF1	CUFF.15972.2	NM 015113	j	TSS25433	
chr17	Cufflinks	exon	3935420	3935552	-	XLOC 01321	TCONS 0006391	14	ZZEF1	CUFF.15972.2	NM 015113	j	TSS25433	
chr17	Cufflinks	exon	3936122	3936296	-	XLOC 01321	TCONS 0006391	15	ZZEF1	CUFF.15972.2	NM 015113	j	TSS25433	
chr17	Cufflinks	exon	3937309	3937586	-	XLOC 01321	TCONS 0006391	16	ZZEF1	CUFF.15972.2	NM 015113	j	TSS25433	
chr17	Cufflinks	exon	3945723	3945862	-	XLOC 01321	TCONS 0006391	17	ZZEF1	CUFF.15972.2	NM 015113	j	TSS25433	
chr17	Cufflinks	exon	3947518	3947668	-	XLOC 01321	TCONS 0006391	18	ZZEF1	CUFF.15972.2	NM 015113	j	TSS25433	
chr17	Cufflinks	exon	3953002	3953153	-	XLOC 01321	TCONS 0006391	19	ZZEF1	CUFF.15972.2	NM 015113	j	TSS25433	
chr17	Cufflinks	exon	3954075	3954337	-	XLOC 01321	TCONS 0006391	20	ZZEF1	CUFF.15972.2	NM 015113	j	TSS25433	
chr17	Cufflinks	exon	3955265	3955430	-	XLOC 01321	TCONS 0006391	21	ZZEF1	CUFF.15972.2	NM 015113	j	TSS25433	
chr17	Cufflinks	exon	3957351	3957489	-	XLOC 01321	TCONS 0006391	22	ZZEF1	CUFF.15972.2	NM 015113	j	TSS25433	
chr17	Cufflinks	exon	3959510	3959639	-	XLOC 01321	TCONS 0006391	23	ZZEF1	CUFF.15972.2	NM 015113	j	TSS25433	
chr17	Cufflinks	exon	3961288	3961449	-	XLOC 01321	TCONS 0006391	24	ZZEF1	CUFF.15972.2	NM 015113	j	TSS25433	
chr17	Cufflinks	exon	3962465	3962584	-	XLOC 01321	TCONS 0006391	25	ZZEF1	CUFF.15972.2	NM 015113	j	TSS25433	
chr17	Cufflinks	exon	3966047	3966211	-	XLOC 01321	TCONS 0006391	26	ZZEF1	CUFF.15972.2	NM 015113	j	TSS25433	
chr17	Cufflinks	exon	3967655	3968123	-	XLOC 01321	TCONS 0006391	27	ZZEF1	CUFF.15972.2	NM 015113	j	TSS25433	

chr17	Cufflinks	exon	3969741	3969834	-	XLOC 01321	TCONS 0006391	28	ZZEF1	CUFF.15972.2	NM 015113	j	TSS25433	
chr17	Cufflinks	exon	3970457	3970536	-	XLOC 01321	TCONS 0006391	29	ZZEF1	CUFF.15972.2	NM 015113	j	TSS25433	
chr17	Cufflinks	exon	3974105	3974218	-	XLOC 01321	TCONS 0006391	30	ZZEF1	CUFF.15972.2	NM 015113	j	TSS25433	
chr17	Cufflinks	exon	3974633	3974719	-	XLOC 01321	TCONS 0006391	31	ZZEF1	CUFF.15972.2	NM 015113	j	TSS25433	
chr17	Cufflinks	exon	43189006	43190072	-	XLOC 01363	TCONS 0006619	1	PLCD3	CUFF.16948.1	NM 133373	j	TSS26252	
chr17	Cufflinks	exon	43190231	43190380	-	XLOC 01363	TCONS 0006619	2	PLCD3	CUFF.16948.1	NM 133373	j	TSS26252	
chr17	Cufflinks	exon	43190490	43190625	-	XLOC 01363	TCONS 0006619	3	PLCD3	CUFF.16948.1	NM 133373	j	TSS26252	
chr17	Cufflinks	exon	43190807	43190973	-	XLOC 01363	TCONS 0006619	4	PLCD3	CUFF.16948.1	NM 133373	j	TSS26252	
chr17	Cufflinks	exon	43191618	43191734	-	XLOC 01363	TCONS 0006619	5	PLCD3	CUFF.16948.1	NM 133373	j	TSS26252	
chr17	Cufflinks	exon	43192462	43192611	-	XLOC 01363	TCONS 0006619	6	PLCD3	CUFF.16948.1	NM 133373	j	TSS26252	
chr17	Cufflinks	exon	43192711	43192857	-	XLOC 01363	TCONS 0006619	7	PLCD3	CUFF.16948.1	NM 133373	j	TSS26252	
chr17	Cufflinks	exon	43193999	43194151	-	XLOC 01363	TCONS 0006619	8	PLCD3	CUFF.16948.1	NM 133373	j	TSS26252	
chr17	Cufflinks	exon	43195361	43195505	-	XLOC 01363	TCONS 0006619	9	PLCD3	CUFF.16948.1	NM 133373	j	TSS26252	
chr17	Cufflinks	exon	43195658	43195859	-	XLOC 01363	TCONS 0006619	10	PLCD3	CUFF.16948.1	NM 133373	j	TSS26252	
chr17	Cufflinks	exon	43196182	43196410	-	XLOC 01363	TCONS 0006619	11	PLCD3	CUFF.16948.1	NM 133373	j	TSS26252	
chr17	Cufflinks	exon	43197692	43197821	-	XLOC 01363	TCONS 0006619	12	PLCD3	CUFF.16948.1	NM 133373	j	TSS26252	
chr17	Cufflinks	exon	43198269	43198497	-	XLOC 01363	TCONS 0006619	13	PLCD3	CUFF.16948.1	NM 133373	j	TSS26252	
chr17	Cufflinks	exon	43198578	43198739	-	XLOC 01363	TCONS 0006619	14	PLCD3	CUFF.16948.1	NM 133373	j	TSS26252	
chr17	Cufflinks	exon	43200033	43200091	-	XLOC 01363	TCONS 0006619	15	PLCD3	CUFF.16948.1	NM 133373	j	TSS26252	
chr18	Cufflinks	exon	61144144	61144278	+	XLOC 01436	TCONS 0006941	1	SERPINB5	NM 002639	NM 002639	=	TSS27602	P15327
chr18	Cufflinks	exon	61151655	61151829	+	XLOC 01436	TCONS 0006941	2	SERPINB5	NM 002639	NM 002639	=	TSS27602	P15327
chr18	Cufflinks	exon	61154179	61154316	+	XLOC 01436	TCONS 0006941	3	SERPINB5	NM 002639	NM 002639	=	TSS27602	P15327
chr18	Cufflinks	exon	61156580	61156697	+	XLOC 01436	TCONS 0006941	4	SERPINB5	NM 002639	NM 002639	=	TSS27602	P15327
chr18	Cufflinks	exon	61160186	61160328	+	XLOC 01436	TCONS 0006941	5	SERPINB5	NM 002639	NM 002639	=	TSS27602	P15327
chr18	Cufflinks	exon	61166353	61166520	+	XLOC 01436	TCONS 0006941	6	SERPINB5	NM 002639	NM 002639	=	TSS27602	P15327
chr18	Cufflinks	exon	61170563	61172318	+	XLOC 01436	TCONS 0006941	7	SERPINB5	NM 002639	NM 002639	=	TSS27602	P15327
chr18	Cufflinks	exon	47309874	47310301	-	XLOC 01454	TCONS 0007035	1	ACAA2	NM 006111	NM 006111	=	TSS27997	P15545
chr18	Cufflinks	exon	47311567	47311721	-	XLOC 01454	TCONS 0007035	2	ACAA2	NM 006111	NM 006111	=	TSS27997	P15545
chr18	Cufflinks	exon	47313661	47313731	-	XLOC 01454	TCONS 0007035	3	ACAA2	NM 006111	NM 006111	=	TSS27997	P15545
chr18	Cufflinks	exon	47317840	47317969	-	XLOC 01454	TCONS 0007035	4	ACAA2	NM 006111	NM 006111	=	TSS27997	P15545
chr18	Cufflinks	exon	47318522	47318697	-	XLOC 01454	TCONS 0007035	5	ACAA2	NM 006111	NM 006111	=	TSS27997	P15545
chr18	Cufflinks	exon	47320650	47320797	-	XLOC 01454	TCONS 0007035	6	ACAA2	NM 006111	NM 006111	=	TSS27997	P15545
chr18	Cufflinks	exon	47322135	47322251	-	XLOC 01454	TCONS 0007035	7	ACAA2	NM 006111	NM 006111	=	TSS27997	P15545
chr18	Cufflinks	exon	47323836	47323964	-	XLOC 01454	TCONS 0007035	8	ACAA2	NM 006111	NM 006111	=	TSS27997	P15545
chr18	Cufflinks	exon	47329057	47329223	-	XLOC 01454	TCONS 0007035	9	ACAA2	NM 006111	NM 006111	=	TSS27997	P15545
chr18	Cufflinks	exon	47339839	47340251	-	XLOC 01454	TCONS 0007035	10	ACAA2	NM 006111	NM 006111	=	TSS27997	P15545
chr18	Cufflinks	exon	6941743	6942238	-	XLOC 01444	TCONS 0006978	1	LAMA1	CUFF.18031.2	NM 005559	j	TSS27765	
chr18	Cufflinks	exon	6943179	6943401	-	XLOC 01444	TCONS 0006978	2	LAMA1	CUFF.18031.2	NM 005559	j	TSS27765	
chr18	Cufflinks	exon	6947162	6947295	-	XLOC 01444	TCONS 0006978	3	LAMA1	CUFF.18031.2	NM 005559	j	TSS27765	
chr18	Cufflinks	exon	6947573	6947677	-	XLOC 01444	TCONS 0006978	4	LAMA1	CUFF.18031.2	NM 005559	j	TSS27765	
chr19	Cufflinks	exon	751146	751171	+	XLOC 01473	TCONS 0007085	1	MISP	NM 173481	NM 173481	=	TSS28256	P15660
chr19	Cufflinks	exon	756890	758726	+	XLOC 01473	TCONS 0007085	2	MISP	NM 173481	NM 173481	=	TSS28256	P15660
chr19	Cufflinks	exon	759909	760039	+	XLOC 01473	TCONS 0007085	3	MISP	NM 173481	NM 173481	=	TSS28256	P15660
chr19	Cufflinks	exon	761625	761663	+	XLOC 01473	TCONS 0007085	4	MISP	NM 173481	NM 173481	=	TSS28256	P15660
chr19	Cufflinks	exon	763501	764318	+	XLOC 01473	TCONS 0007085	5	MISP	NM 173481	NM 173481	=	TSS28256	P15660
chr19	Cufflinks	exon	3094408	3094785	+	XLOC 01477	TCONS 0007109	1	GNA11	NM 002067	NM 002067	=	TSS28351	P15725
chr19	Cufflinks	exon	3110147	3110331	+	XLOC 01477	TCONS 0007109	2	GNA11	NM 002067	NM 002067	=	TSS28351	P15725
chr19	Cufflinks	exon	3113328	3113482	+	XLOC 01477	TCONS 0007109	3	GNA11	NM 002067	NM 002067	=	TSS28351	P15725
chr19	Cufflinks	exon	3114942	3115070	+	XLOC 01477	TCONS 0007109	4	GNA11	NM 002067	NM 002067	=	TSS28351	P15725
chr19	Cufflinks	exon	3118922	3119051	+	XLOC 01477	TCONS 0007109	5	GNA11	NM 002067	NM 002067	=	TSS28351	P15725
chr19	Cufflinks	exon	3119204	3119357	+	XLOC 01477	TCONS 0007109	6	GNA11	NM 002067	NM 002067	=	TSS28351	P15725
chr19	Cufflinks	exon	3120987	3124000	+	XLOC 01477	TCONS 0007109	7	GNA11	NM 002067	NM 002067	=	TSS28351	P15725
chr19	Cufflinks	exon	50922195	50922242	+	XLOC 01536	TCONS 0007454	1	SPIB	NM 003121	NM 003121	=	TSS29580	P16551
chr19	Cufflinks	exon	50923203	50923230	+	XLOC 01536	TCONS 0007454	2	SPIB	NM 003121	NM 003121	=	TSS29580	P16551
chr19	Cufflinks	exon	50925730	50925802	+	XLOC 01536	TCONS 0007454	3	SPIB	NM 003121	NM 003121	=	TSS29580	P16551
chr19	Cufflinks	exon	50926080	50926294	+	XLOC 01536	TCONS 0007454	4	SPIB	NM 003121	NM 003121	=	TSS29580	P16551
chr19	Cufflinks	exon	50926862	50927012	+	XLOC 01536	TCONS 0007454	5	SPIB	NM 003121	NM 003121	=	TSS29580	P16551
chr19	Cufflinks	exon	50931295	50934309	+	XLOC 01536	TCONS 0007454	6	SPIB	NM 003121	NM 003121	=	TSS29580	P16551
chr19	Cufflinks	exon	4174106	4174943	-	XLOC 01563	TCONS 0007591	1	SIRT6	NM 016539	NM 016539	=	TSS30115	P16881
chr19	Cufflinks	exon	4175025	4175148	-	XLOC 01563	TCONS 0007591	2	SIRT6	NM 016539	NM 016539	=	TSS30115	P16881
chr19	Cufflinks	exon	4175677	4175757	-	XLOC 01563	TCONS 0007591	3	SIRT6	NM 016539	NM 016539	=	TSS30115	P16881
chr19	Cufflinks	exon	4175839	4175934	-	XLOC 01563	TCONS 0007591	4	SIRT6	NM 016539	NM 016539	=	TSS30115	P16881
chr19	Cufflinks	exon	4177076	4177135	-	XLOC 01563	TCONS 0007591	5	SIRT6	NM 016539	NM 016539	=	TSS30115	P16881
chr19	Cufflinks	exon	4179101	4179283	-	XLOC 01563	TCONS 0007591	6	SIRT6	NM 016539	NM 016539	=	TSS30115	P16881
chr19	Cufflinks	exon	4180779	4180906	-	XLOC 01563	TCONS 0007591	7	SIRT6	NM 016539	NM 016539	=	TSS30115	P16881
chr19	Cufflinks	exon	4182471	4182596	-	XLOC 01563	TCONS 0007591	8	SIRT6	NM 016539	NM 016539	=	TSS30115	P16881
chr19	Cufflinks	exon	4815936	4818528	-	XLOC 01564	TCONS 0007595	1	TICAM1	NM 182919	NM 182919	=	TSS30138	P16899
chr19	Cufflinks	exon	4831626	4831754	-	XLOC 01564	TCONS 0007595	2	TICAM1	NM 182919	NM 182919	=	TSS30138	P16899
chr19	Cufflinks	exon	17342694	17343435	-	XLOC 01583	TCONS 0007713	1	NR2F6	NM 005234	NM 005234	=	TSS30533	P17166
chr19	Cufflinks	exon	17346308	17346874	-	XLOC 01583	TCONS 0007713	2	NR2F6	NM 005234	NM 005234	=	TSS30533	P17166
chr19	Cufflinks	exon	17351480	17351574	-	XLOC 01583	TCONS 0007713	3	NR2F6	NM 005234	NM 005234	=	TSS30533	P17166
chr19	Cufflinks	exon	17355752	17356151	-	XLOC 01583	TCONS 0007713	4	NR2F6	NM 005234	NM 005234	=	TSS30533	P17166
chr19	Cufflinks	exon	40928409	40929453	-	XLOC 01601	TCONS 0007820	1	SERTAD1	NM 013376	NM 013376	=	TSS30875	P17412
chr19	Cufflinks	exon	40931774	40931932	-	XLOC 01601	TCONS 0007820	2	SERTAD1	NM 013376	NM 013376	=	TSS30875	P17412
chr19	Cufflinks	exon	54976209	54976731	-	XLOC 01629	TCONS 0007968	1	CDC42EP5	NM 145057	NM 145057	=	TSS31420	P17827
chr19	Cufflinks	exon	54982766	54982906	-	XLOC 01629	TCONS 0007968	2	CDC42EP5	NM 145057	NM 145057	=	TSS31420	P17827
chr19	Cufflinks	exon	54984211	54984437	-	XLOC 01629	TCONS 0007968	3	CDC42EP5	NM 145057	NM 145057	=	TSS31420	P17827
chr2	Cufflinks	exon	29117509	29117789	+	XLOC 01662	TCONS 0008085	1	WDR43	NM 015131	NM 015131	=	TSS31979	P18082
chr2	Cufflinks	exon	29124853	29124990	+	XLOC 01662	TCONS 0008085	2	WDR43	NM 015131	NM 015131	=	TSS31979	P18082
chr2	Cufflinks	exon	29129326	29129447	+	XLOC 01662	TCONS 0008085	3	WDR43	NM 015131	NM 015131	=	TSS31979	P18082
chr2	Cufflinks	exon	29135456	29135576	+	XLOC 01662	TCONS 0008085	4	WDR43	NM 015131	NM 015131	=	TSS31979	P18082
chr2	Cufflinks	exon	29136935	29137074	+	XLOC 01662	TCONS 0008085	5	WDR43	NM 015131	NM 015131	=	TSS31979	P18082
chr2	Cufflinks	exon	29140759	29140861	+	XLOC 01662	TCONS 0008085	6	WDR43	NM 015131	NM 015131	=	TSS31979	P18082
chr2	Cufflinks	exon	29145785	29145849	+	XLOC 01662	TCONS 0008085	7	WDR43	NM 015131	NM 015131	=	TSS31979	P18082
chr2	Cufflinks	exon	29147848	29148019	+	XLOC 01662	TCONS 0008085	8	WDR43	NM 015131	NM 015131	=	TSS31979	P18082
chr2	Cufflinks	exon	29149334	29149420	+	XLOC 01662	TCONS 0008085	9	WDR43	NM 015131	NM 015131	=	TSS31979	P18082
chr2	Cufflinks	exon	29150435	29150566	+	XLOC 01662	TCONS 0008085	10	WDR43	NM 015131	NM 015131	=	TSS31979	P18082
chr2	Cufflinks	exon	29152445	29152576	+	XLOC 01662	TCONS 0008085	11	WDR43	NM 015131	NM 015131	=	TSS31979	P18082
chr2	Cufflinks	exon	29158387	29158473	+	XLOC 01662	TCONS 0008085	12	WDR43	NM 015131	NM 015131	=	TSS31979	P18082
chr2	Cufflinks	exon	29159788	29159819	+	XLOC 01662	TCONS 0008085	13	WDR43	NM 015131	NM 015131	=	TSS31979	P18082
chr2	Cufflinks	exon	29160797	29160860	+	XLOC 01662	TCONS 0008085	14	WDR43	NM 015131	NM 015131	=	TSS31979	P18082
chr2	Cufflinks	exon	29164327	29164440	+	XLOC 01662	TCONS 0008085	15	WDR43	NM 015131	NM 015131	=	TSS31979	P18082
chr2	Cufflinks	exon	29165178	29165247	+	XLOC 01662	TCONS 0008085	16	WDR43	NM 015131	NM 015131	=	TSS31979	P18082
chr2	Cufflinks	exon	29169316	29169365	+	XLOC 01662	TCONS 0008085	17	WDR43	NM 015131	NM 015131	=	TSS31979	P18082
chr2	Cufflinks	exon	29169466	29171086	+	XLOC 01662	TCONS 0008085	18	WDR43	NM 015131	NM 015131	=	TSS31979	P18082
chr2	Cufflinks	exon	85804614	85804772	+	XLOC 01682	TCONS 0008196	1	VAMP8	NM 003761	NM 003761	=	TSS32415	P18311
chr2	Cufflinks	exon	85806132	85806290	+	XLOC 01682	TCONS 0008196	2	VAMP8	NM 003761	NM 003761	=	TSS32415	P18311
chr2	Cufflinks	exon	85808699	85809156	+	XLOC 01682	TCONS 0008196	3	VAMP8	NM 003761	NM 003761	=	TSS32415	P18311
chr2	Cufflinks	exon	219646472	219647160	+	XLOC 01733	TCONS 0008484	1	CYP27A1	NM 000784	NM 000784	=	TSS33507	P18903
chr2	Cufflinks	exon	219674300	219674490	+	XLOC 01733	TCONS 0008484	2	CYP27A1	NM 000784	NM 000784	=	TSS33507	P18903

chr2	Cufflinks	exon	219676945	219677144	+	XLOC 01733	TCONS 0008484	3	CYP27A1	NM 000784	NM 000784	=	TSS33507	P18903
chr2	Cufflinks	exon	219677275	219677472	+	XLOC 01733	TCONS 0008484	4	CYP27A1	NM 000784	NM 000784	=	TSS33507	P18903
chr2	Cufflinks	exon	219677647	219677819	+	XLOC 01733	TCONS 0008484	5	CYP27A1	NM 000784	NM 000784	=	TSS33507	P18903
chr2	Cufflinks	exon	219678744	219678910	+	XLOC 01733	TCONS 0008484	6	CYP27A1	NM 000784	NM 000784	=	TSS33507	P18903
chr2	Cufflinks	exon	219679103	219679181	+	XLOC 01733	TCONS 0008484	7	CYP27A1	NM 000784	NM 000784	=	TSS33507	P18903
chr2	Cufflinks	exon	219679268	219679480	+	XLOC 01733	TCONS 0008484	8	CYP27A1	NM 000784	NM 000784	=	TSS33507	P18903
chr2	Cufflinks	exon	219679634	219680016	+	XLOC 01733	TCONS 0008484	9	CYP27A1	NM 000784	NM 000784	=	TSS33507	P18903
chr2	Cufflinks	exon	113531492	113532844	-	XLOC 01793	TCONS 0008822	1	IL1A	NM 000575	NM 000575	=	TSS34820	P19577
chr2	Cufflinks	exon	113535564	113535688	-	XLOC 01793	TCONS 0008822	2	IL1A	NM 000575	NM 000575	=	TSS34820	P19577
chr2	Cufflinks	exon	113537073	113537243	-	XLOC 01793	TCONS 0008822	3	IL1A	NM 000575	NM 000575	=	TSS34820	P19577
chr2	Cufflinks	exon	113539181	113539403	-	XLOC 01793	TCONS 0008822	4	IL1A	NM 000575	NM 000575	=	TSS34820	P19577
chr2	Cufflinks	exon	113540293	113540341	-	XLOC 01793	TCONS 0008822	5	IL1A	NM 000575	NM 000575	=	TSS34820	P19577
chr2	Cufflinks	exon	113541301	113541355	-	XLOC 01793	TCONS 0008822	6	IL1A	NM 000575	NM 000575	=	TSS34820	P19577
chr2	Cufflinks	exon	113542020	113542971	-	XLOC 01793	TCONS 0008822	7	IL1A	NM 000575	NM 000575	=	TSS34820	P19577
chr2	Cufflinks	exon	64069666	64069733	+	XLOC 01673	TCONS 0008148	1	UGP2	CUFF.21705.3	NM 006759	j	TSS32231	
chr2	Cufflinks	exon	64083440	64083567	+	XLOC 01673	TCONS 0008148	2	UGP2	CUFF.21705.3	NM 006759	j	TSS32231	
chr2	Cufflinks	exon	64084963	64085070	+	XLOC 01673	TCONS 0008148	3	UGP2	CUFF.21705.3	NM 006759	j	TSS32231	
chr2	Cufflinks	exon	64109600	64109785	+	XLOC 01673	TCONS 0008148	4	UGP2	CUFF.21705.3	NM 006759	j	TSS32231	
chr2	Cufflinks	exon	64111094	64111227	+	XLOC 01673	TCONS 0008148	5	UGP2	CUFF.21705.3	NM 006759	j	TSS32231	
chr2	Cufflinks	exon	64112723	64113020	+	XLOC 01673	TCONS 0008148	6	UGP2	CUFF.21705.3	NM 006759	j	TSS32231	
chr2	Cufflinks	exon	64113475	64113672	+	XLOC 01673	TCONS 0008148	7	UGP2	CUFF.21705.3	NM 006759	j	TSS32231	
chr2	Cufflinks	exon	64114536	64114778	+	XLOC 01673	TCONS 0008148	8	UGP2	CUFF.21705.3	NM 006759	j	TSS32231	
chr2	Cufflinks	exon	64117215	64117319	+	XLOC 01673	TCONS 0008148	9	UGP2	CUFF.21705.3	NM 006759	j	TSS32231	
chr2	Cufflinks	exon	64118254	64118696	+	XLOC 01673	TCONS 0008148	10	UGP2	CUFF.21705.3	NM 006759	j	TSS32231	
chr20	Cufflinks	exon	361950	361983	+	XLOC 01885	TCONS 0009113	1	TRIB3	NM 001301188	NM 001301188	=	TSS36230	P20125
chr20	Cufflinks	exon	368655	368945	+	XLOC 01885	TCONS 0009113	2	TRIB3	NM 001301188	NM 001301188	=	TSS36230	P20125
chr20	Cufflinks	exon	371931	372223	+	XLOC 01885	TCONS 0009113	3	TRIB3	NM 001301188	NM 001301188	=	TSS36230	P20125
chr20	Cufflinks	exon	376842	378203	+	XLOC 01885	TCONS 0009113	4	TRIB3	NM 001301188	NM 001301188	=	TSS36230	P20125
chr20	Cufflinks	exon	43570771	43571854	-	XLOC 01948	TCONS 0009427	1	TOMM34	NM 006809	NM 006809	=	TSS37476	P20871
chr20	Cufflinks	exon	43572094	43572220	-	XLOC 01948	TCONS 0009427	2	TOMM34	NM 006809	NM 006809	=	TSS37476	P20871
chr20	Cufflinks	exon	43577371	43577518	-	XLOC 01948	TCONS 0009427	3	TOMM34	NM 006809	NM 006809	=	TSS37476	P20871
chr20	Cufflinks	exon	43580474	43580643	-	XLOC 01948	TCONS 0009427	4	TOMM34	NM 006809	NM 006809	=	TSS37476	P20871
chr20	Cufflinks	exon	43583709	43583861	-	XLOC 01948	TCONS 0009427	5	TOMM34	NM 006809	NM 006809	=	TSS37476	P20871
chr20	Cufflinks	exon	43585027	43585126	-	XLOC 01948	TCONS 0009427	6	TOMM34	NM 006809	NM 006809	=	TSS37476	P20871
chr20	Cufflinks	exon	43588848	43589114	-	XLOC 01948	TCONS 0009427	7	TOMM34	NM 006809	NM 006809	=	TSS37476	P20871
chr20	Cufflinks	exon	52560079	52561535	-	XLOC 01954	TCONS 0009465	1	BCAS1	NM 003657	NM 003657	=	TSS37618	P20977
chr20	Cufflinks	exon	52569971	52570234	-	XLOC 01954	TCONS 0009465	2	BCAS1	NM 003657	NM 003657	=	TSS37618	P20977
chr20	Cufflinks	exon	52573971	52574036	-	XLOC 01954	TCONS 0009465	3	BCAS1	NM 003657	NM 003657	=	TSS37618	P20977
chr20	Cufflinks	exon	52583445	52583612	-	XLOC 01954	TCONS 0009465	4	BCAS1	NM 003657	NM 003657	=	TSS37618	P20977
chr20	Cufflinks	exon	52591928	52591969	-	XLOC 01954	TCONS 0009465	5	BCAS1	NM 003657	NM 003657	=	TSS37618	P20977
chr20	Cufflinks	exon	52601826	52602038	-	XLOC 01954	TCONS 0009465	6	BCAS1	NM 003657	NM 003657	=	TSS37618	P20977
chr20	Cufflinks	exon	52611551	52611595	-	XLOC 01954	TCONS 0009465	7	BCAS1	NM 003657	NM 003657	=	TSS37618	P20977
chr20	Cufflinks	exon	52612431	52612589	-	XLOC 01954	TCONS 0009465	8	BCAS1	NM 003657	NM 003657	=	TSS37618	P20977
chr20	Cufflinks	exon	52644931	52645511	-	XLOC 01954	TCONS 0009465	9	BCAS1	NM 003657	NM 003657	=	TSS37618	P20977
chr20	Cufflinks	exon	52674624	52674693	-	XLOC 01954	TCONS 0009465	10	BCAS1	NM 003657	NM 003657	=	TSS37618	P20977
chr20	Cufflinks	exon	52675186	52675262	-	XLOC 01954	TCONS 0009465	11	BCAS1	NM 003657	NM 003657	=	TSS37618	P20977
chr20	Cufflinks	exon	52686972	52687304	-	XLOC 01954	TCONS 0009465	12	BCAS1	NM 003657	NM 003657	=	TSS37618	P20977
chr20	Cufflinks	exon	55743809	55746164	-	XLOC 01955	TCONS 0009467	1	BMP7	NM 001719	NM 001719	=	TSS37627	P20983
chr20	Cufflinks	exon	55748256	55748366	-	XLOC 01955	TCONS 0009467	2	BMP7	NM 001719	NM 001719	=	TSS37627	P20983
chr20	Cufflinks	exon	55749987	55750063	-	XLOC 01955	TCONS 0009467	3	BMP7	NM 001719	NM 001719	=	TSS37627	P20983
chr20	Cufflinks	exon	55758778	55758975	-	XLOC 01955	TCONS 0009467	4	BMP7	NM 001719	NM 001719	=	TSS37627	P20983
chr20	Cufflinks	exon	55777531	55777679	-	XLOC 01955	TCONS 0009467	5	BMP7	NM 001719	NM 001719	=	TSS37627	P20983
chr20	Cufflinks	exon	55803285	55803477	-	XLOC 01955	TCONS 0009467	6	BMP7	NM 001719	NM 001719	=	TSS37627	P20983
chr20	Cufflinks	exon	55840761	55841707	-	XLOC 01955	TCONS 0009467	7	BMP7	NM 001719	NM 001719	=	TSS37627	P20983
chr20	Cufflinks	exon	3767359	3767383	+	XLOC 01888	TCONS 0009127	1	CDC25B	CUFF.24325.1	NM 021873	j	TSS36297	
chr20	Cufflinks	exon	3767743	3767786	+	XLOC 01888	TCONS 0009127	2	CDC25B	CUFF.24325.1	NM 021873	j	TSS36297	
chr20	Cufflinks	exon	3778269	3778396	+	XLOC 01888	TCONS 0009127	3	CDC25B	CUFF.24325.1	NM 021873	j	TSS36297	
chr20	Cufflinks	exon	3779059	3779110	+	XLOC 01888	TCONS 0009127	4	CDC25B	CUFF.24325.1	NM 021873	j	TSS36297	
chr20	Cufflinks	exon	3780935	3780976	+	XLOC 01888	TCONS 0009127	5	CDC25B	CUFF.24325.1	NM 021873	j	TSS36297	
chr20	Cufflinks	exon	3781109	3781145	+	XLOC 01888	TCONS 0009127	6	CDC25B	CUFF.24325.1	NM 021873	j	TSS36297	
chr20	Cufflinks	exon	3781390	3781512	+	XLOC 01888	TCONS 0009127	7	CDC25B	CUFF.24325.1	NM 021873	j	TSS36297	
chr20	Cufflinks	exon	3781618	3781740	+	XLOC 01888	TCONS 0009127	8	CDC25B	CUFF.24325.1	NM 021873	j	TSS36297	
chr20	Cufflinks	exon	3781901	3782035	+	XLOC 01888	TCONS 0009127	9	CDC25B	CUFF.24325.1	NM 021873	j	TSS36297	
chr20	Cufflinks	exon	3782369	3782449	+	XLOC 01888	TCONS 0009127	10	CDC25B	CUFF.24325.1	NM 021873	j	TSS36297	
chr20	Cufflinks	exon	3782571	3782747	+	XLOC 01888	TCONS 0009127	11	CDC25B	CUFF.24325.1	NM 021873	j	TSS36297	
chr20	Cufflinks	exon	3782928	3783023	+	XLOC 01888	TCONS 0009127	12	CDC25B	CUFF.24325.1	NM 021873	j	TSS36297	
chr20	Cufflinks	exon	3783557	3783619	+	XLOC 01888	TCONS 0009127	13	CDC25B	CUFF.24325.1	NM 021873	j	TSS36297	
chr20	Cufflinks	exon	3783755	3783853	+	XLOC 01888	TCONS 0009127	14	CDC25B	CUFF.24325.1	NM 021873	j	TSS36297	
chr20	Cufflinks	exon	3784051	3784184	+	XLOC 01888	TCONS 0009127	15	CDC25B	CUFF.24325.1	NM 021873	j	TSS36297	
chr20	Cufflinks	exon	3785216	3785327	+	XLOC 01888	TCONS 0009127	16	CDC25B	CUFF.24325.1	NM 021873	j	TSS36297	
chr20	Cufflinks	exon	3785468	3786787	+	XLOC 01888	TCONS 0009127	17	CDC25B	CUFF.24325.1	NM 021873	j	TSS36297	
chr21	Cufflinks	exon	42836478	42838080	-	XLOC 02003	TCONS 0009663	1	TMPRSS2	NM 005656	NM 005656	=	TSS38486	P21379
chr21	Cufflinks	exon	42839661	42839813	-	XLOC 02003	TCONS 0009663	2	TMPRSS2	NM 005656	NM 005656	=	TSS38486	P21379
chr21	Cufflinks	exon	42840323	42840465	-	XLOC 02003	TCONS 0009663	3	TMPRSS2	NM 005656	NM 005656	=	TSS38486	P21379
chr21	Cufflinks	exon	42842575	42842670	-	XLOC 02003	TCONS 0009663	4	TMPRSS2	NM 005656	NM 005656	=	TSS38486	P21379
chr21	Cufflinks	exon	42843733	42843908	-	XLOC 02003	TCONS 0009663	5	TMPRSS2	NM 005656	NM 005656	=	TSS38486	P21379
chr21	Cufflinks	exon	42845252	42845423	-	XLOC 02003	TCONS 0009663	6	TMPRSS2	NM 005656	NM 005656	=	TSS38486	P21379
chr21	Cufflinks	exon	42848504	42848547	-	XLOC 02003	TCONS 0009663	7	TMPRSS2	NM 005656	NM 005656	=	TSS38486	P21379
chr21	Cufflinks	exon	42851099	42851209	-	XLOC 02003	TCONS 0009663	8	TMPRSS2	NM 005656	NM 005656	=	TSS38486	P21379
chr21	Cufflinks	exon	42852403	42852529	-	XLOC 02003	TCONS 0009663	9	TMPRSS2	NM 005656	NM 005656	=	TSS38486	P21379
chr21	Cufflinks	exon	42860321	42860440	-	XLOC 02003	TCONS 0009663	10	TMPRSS2	NM 005656	NM 005656	=	TSS38486	P21379
chr21	Cufflinks	exon	42861434	42861520	-	XLOC 02003	TCONS 0009663	11	TMPRSS2	NM 005656	NM 005656	=	TSS38486	P21379
chr21	Cufflinks	exon	42866283	42866505	-	XLOC 02003	TCONS 0009663	12	TMPRSS2	NM 005656	NM 005656	=	TSS38486	P21379
chr21	Cufflinks	exon	42870046	42870116	-	XLOC 02003	TCONS 0009663	13	TMPRSS2	NM 005656	NM 005656	=	TSS38486	P21379
chr21	Cufflinks	exon	42880008	42880085	-	XLOC 02003	TCONS 0009663	14	TMPRSS2	NM 005656	NM 005656	=	TSS38486	P21379
chr22	Cufflinks	exon	41972890	41973444	-	XLOC 02073	TCONS 0010002	1	PMM1	NM 002676	NM 002676	=	TSS39815	P22120
chr22	Cufflinks	exon	41973812	41973927	-	XLOC 02073	TCONS 0010002	2	PMM1	NM 002676	NM 002676	=	TSS39815	P22120
chr22	Cufflinks	exon	41974810	41974885	-	XLOC 02073	TCONS 0010002	3	PMM1	NM 002676	NM 002676	=	TSS39815	P22120
chr22	Cufflinks	exon	41979963	41980062	-	XLOC 02073	TCONS 0010002	4	PMM1	NM 002676	NM 002676	=	TSS39815	P22120
chr22	Cufflinks	exon	41980285	41980376	-	XLOC 02073	TCONS 0010002	5	PMM1	NM 002676	NM 002676	=	TSS39815	P22120
chr22	Cufflinks	exon	41980531	41980607	-	XLOC 02073	TCONS 0010002	6	PMM1	NM 002676	NM 002676	=	TSS39815	P22120
chr22	Cufflinks	exon	41982080	41982197	-	XLOC 02073	TCONS 0010002	7	PMM1	NM 002676	NM 002676	=	TSS39815	P22120
chr22	Cufflinks	exon	41985723	41985871	-	XLOC 02073	TCONS 0010002	8	PMM1	NM 002676	NM 002676	=	TSS39815	P22120
chr3	Cufflinks	exon	53195223	53195444	+	XLOC 02115	TCONS 0010246	1	PRKCD	NM 212539	NM 212539	=	TSS40700	P22582
chr3	Cufflinks	exon	53212420	53212553	+	XLOC 02115	TCONS 0010246	2	PRKCD	NM 212539	NM 212539	=	TSS40700	P22582
chr3	Cufflinks	exon	53213593	53213792	+	XLOC 02115	TCONS 0010246	3	PRKCD	NM 212539	NM 212539	=	TSS40700	P22582
chr3	Cufflinks	exon	53215223	53215283	+	XLOC 02115	TCONS 0010246	4	PRKCD	NM 212539	NM 212539	=	TSS40700	P22582
chr3	Cufflinks	exon	53215460	53215622	+	XLOC 02115	TCONS 0010246	5	PRKCD	NM 212539	NM 212539	=	TSS40700	P22582
chr3	Cufflinks	exon	53215717	53215748	+	XLOC 02115	TCONS 0010246	6	PRKCD	NM 212539	NM 212539	=	TSS40700	P22582
chr3	Cufflinks	exon	53217137	53217222	+	XLOC 02115	TCONS 0010246	7	PRKCD	NM 212539	NM 212539	=	TSS40700	P22582

chr3	Cufflinks	exon	53217468	53217597	+	XLOC 02115	TCONS 0010246	8	PRKCD	NM 212539	NM 212539	=	TSS40700	P22582
chr3	Cufflinks	exon	53218890	53218990	+	XLOC 02115	TCONS 0010246	9	PRKCD	NM 212539	NM 212539	=	TSS40700	P22582
chr3	Cufflinks	exon	53219620	53219716	+	XLOC 02115	TCONS 0010246	10	PRKCD	NM 212539	NM 212539	=	TSS40700	P22582
chr3	Cufflinks	exon	53219943	53220043	+	XLOC 02115	TCONS 0010246	11	PRKCD	NM 212539	NM 212539	=	TSS40700	P22582
chr3	Cufflinks	exon	53220183	53220356	+	XLOC 02115	TCONS 0010246	12	PRKCD	NM 212539	NM 212539	=	TSS40700	P22582
chr3	Cufflinks	exon	53220620	53220711	+	XLOC 02115	TCONS 0010246	13	PRKCD	NM 212539	NM 212539	=	TSS40700	P22582
chr3	Cufflinks	exon	53221356	53221418	+	XLOC 02115	TCONS 0010246	14	PRKCD	NM 212539	NM 212539	=	TSS40700	P22582
chr3	Cufflinks	exon	53222736	53222874	+	XLOC 02115	TCONS 0010246	15	PRKCD	NM 212539	NM 212539	=	TSS40700	P22582
chr3	Cufflinks	exon	53223074	53223262	+	XLOC 02115	TCONS 0010246	16	PRKCD	NM 212539	NM 212539	=	TSS40700	P22582
chr3	Cufflinks	exon	53223889	53224017	+	XLOC 02115	TCONS 0010246	17	PRKCD	NM 212539	NM 212539	=	TSS40700	P22582
chr3	Cufflinks	exon	53226124	53226733	+	XLOC 02115	TCONS 0010246	18	PRKCD	NM 212539	NM 212539	=	TSS40700	P22582
chr3	Cufflinks	exon	108030632	108030681	+	XLOC 02127	TCONS 0010305	1	HHLA2	NM 001282557	NM 001282557	=	TSS40938	P22712
chr3	Cufflinks	exon	108047117	108047194	+	XLOC 02127	TCONS 0010305	2	HHLA2	NM 001282557	NM 001282557	=	TSS40938	P22712
chr3	Cufflinks	exon	108070635	108070724	+	XLOC 02127	TCONS 0010305	3	HHLA2	NM 001282557	NM 001282557	=	TSS40938	P22712
chr3	Cufflinks	exon	108072274	108072627	+	XLOC 02127	TCONS 0010305	4	HHLA2	NM 001282557	NM 001282557	=	TSS40938	P22712
chr3	Cufflinks	exon	108073962	108074228	+	XLOC 02127	TCONS 0010305	5	HHLA2	NM 001282557	NM 001282557	=	TSS40938	P22712
chr3	Cufflinks	exon	108076691	108077008	+	XLOC 02127	TCONS 0010305	6	HHLA2	NM 001282557	NM 001282557	=	TSS40938	P22712
chr3	Cufflinks	exon	108081189	108081293	+	XLOC 02127	TCONS 0010305	7	HHLA2	NM 001282557	NM 001282557	=	TSS40938	P22712
chr3	Cufflinks	exon	108094597	108094647	+	XLOC 02127	TCONS 0010305	8	HHLA2	NM 001282557	NM 001282557	=	TSS40938	P22712
chr3	Cufflinks	exon	108095340	108095404	+	XLOC 02127	TCONS 0010305	9	HHLA2	NM 001282557	NM 001282557	=	TSS40938	P22712
chr3	Cufflinks	exon	108096105	108097131	+	XLOC 02127	TCONS 0010305	10	HHLA2	NM 001282557	NM 001282557	=	TSS40938	P22712
chr3	Cufflinks	exon	127317200	127317315	+	XLOC 02137	TCONS 0010352	1	MCM2	NM 004526	NM 004526	=	TSS41105	P22805
chr3	Cufflinks	exon	127318161	127318390	+	XLOC 02137	TCONS 0010352	2	MCM2	NM 004526	NM 004526	=	TSS41105	P22805
chr3	Cufflinks	exon	127323451	127323626	+	XLOC 02137	TCONS 0010352	3	MCM2	NM 004526	NM 004526	=	TSS41105	P22805
chr3	Cufflinks	exon	127323739	127323999	+	XLOC 02137	TCONS 0010352	4	MCM2	NM 004526	NM 004526	=	TSS41105	P22805
chr3	Cufflinks	exon	127324961	127325180	+	XLOC 02137	TCONS 0010352	5	MCM2	NM 004526	NM 004526	=	TSS41105	P22805
chr3	Cufflinks	exon	127325453	127325660	+	XLOC 02137	TCONS 0010352	6	MCM2	NM 004526	NM 004526	=	TSS41105	P22805
chr3	Cufflinks	exon	127327225	127327359	+	XLOC 02137	TCONS 0010352	7	MCM2	NM 004526	NM 004526	=	TSS41105	P22805
chr3	Cufflinks	exon	127327675	127327866	+	XLOC 02137	TCONS 0010352	8	MCM2	NM 004526	NM 004526	=	TSS41105	P22805
chr3	Cufflinks	exon	127334705	127334798	+	XLOC 02137	TCONS 0010352	9	MCM2	NM 004526	NM 004526	=	TSS41105	P22805
chr3	Cufflinks	exon	127335711	127335961	+	XLOC 02137	TCONS 0010352	10	MCM2	NM 004526	NM 004526	=	TSS41105	P22805
chr3	Cufflinks	exon	127336122	127336248	+	XLOC 02137	TCONS 0010352	11	MCM2	NM 004526	NM 004526	=	TSS41105	P22805
chr3	Cufflinks	exon	127336812	127336924	+	XLOC 02137	TCONS 0010352	12	MCM2	NM 004526	NM 004526	=	TSS41105	P22805
chr3	Cufflinks	exon	127337870	127338121	+	XLOC 02137	TCONS 0010352	13	MCM2	NM 004526	NM 004526	=	TSS41105	P22805
chr3	Cufflinks	exon	127339541	127339723	+	XLOC 02137	TCONS 0010352	14	MCM2	NM 004526	NM 004526	=	TSS41105	P22805
chr3	Cufflinks	exon	127339916	127340071	+	XLOC 02137	TCONS 0010352	15	MCM2	NM 004526	NM 004526	=	TSS41105	P22805
chr3	Cufflinks	exon	127340506	127341278	+	XLOC 02137	TCONS 0010352	16	MCM2	NM 004526	NM 004526	=	TSS41105	P22805
chr3	Cufflinks	exon	141595470	141595752	+	XLOC 02144	TCONS 0010391	1	ATP1B3	NM 001679	NM 001679	=	TSS41263	P22911
chr3	Cufflinks	exon	141622462	141622590	+	XLOC 02144	TCONS 0010391	2	ATP1B3	NM 001679	NM 001679	=	TSS41263	P22911
chr3	Cufflinks	exon	141626009	141626116	+	XLOC 02144	TCONS 0010391	3	ATP1B3	NM 001679	NM 001679	=	TSS41263	P22911
chr3	Cufflinks	exon	141632494	141632678	+	XLOC 02144	TCONS 0010391	4	ATP1B3	NM 001679	NM 001679	=	TSS41263	P22911
chr3	Cufflinks	exon	141634812	141634862	+	XLOC 02144	TCONS 0010391	5	ATP1B3	NM 001679	NM 001679	=	TSS41263	P22911
chr3	Cufflinks	exon	141640819	141640905	+	XLOC 02144	TCONS 0010391	6	ATP1B3	NM 001679	NM 001679	=	TSS41263	P22911
chr3	Cufflinks	exon	141644373	141645382	+	XLOC 02144	TCONS 0010391	7	ATP1B3	NM 001679	NM 001679	=	TSS41263	P22911
chr3	Cufflinks	exon	149192368	149192838	+	XLOC 02146	TCONS 0010399	1	TM4SF4	NM 004617	NM 004617	=	TSS41290	P22930
chr3	Cufflinks	exon	149193610	149193699	+	XLOC 02146	TCONS 0010399	2	TM4SF4	NM 004617	NM 004617	=	TSS41290	P22930
chr3	Cufflinks	exon	149205406	149205542	+	XLOC 02146	TCONS 0010399	3	TM4SF4	NM 004617	NM 004617	=	TSS41290	P22930
chr3	Cufflinks	exon	149216509	149216698	+	XLOC 02146	TCONS 0010399	4	TM4SF4	NM 004617	NM 004617	=	TSS41290	P22930
chr3	Cufflinks	exon	149220463	149221181	+	XLOC 02146	TCONS 0010399	5	TM4SF4	NM 004617	NM 004617	=	TSS41290	P22930
chr3	Cufflinks	exon	160394948	160396235	+	XLOC 02151	TCONS 0010422	1	ARL14	NM 025047	NM 025047	=	TSS41382	P22994
chr3	Cufflinks	exon	172468475	172468647	+	XLOC 02154	TCONS 0010434	1	ECT2	NM 018098	NM 018098	=	TSS41440	P23013
chr3	Cufflinks	exon	172472299	172472450	+	XLOC 02154	TCONS 0010434	2	ECT2	NM 018098	NM 018098	=	TSS41440	P23013
chr3	Cufflinks	exon	172473085	172473164	+	XLOC 02154	TCONS 0010434	3	ECT2	NM 018098	NM 018098	=	TSS41440	P23013
chr3	Cufflinks	exon	172474773	172474955	+	XLOC 02154	TCONS 0010434	4	ECT2	NM 018098	NM 018098	=	TSS41440	P23013
chr3	Cufflinks	exon	172476770	172476859	+	XLOC 02154	TCONS 0010434	5	ECT2	NM 018098	NM 018098	=	TSS41440	P23013
chr3	Cufflinks	exon	172477946	172478053	+	XLOC 02154	TCONS 0010434	6	ECT2	NM 018098	NM 018098	=	TSS41440	P23013
chr3	Cufflinks	exon	172479400	172479473	+	XLOC 02154	TCONS 0010434	7	ECT2	NM 018098	NM 018098	=	TSS41440	P23013
chr3	Cufflinks	exon	172480206	172480336	+	XLOC 02154	TCONS 0010434	8	ECT2	NM 018098	NM 018098	=	TSS41440	P23013
chr3	Cufflinks	exon	172480481	172480596	+	XLOC 02154	TCONS 0010434	9	ECT2	NM 018098	NM 018098	=	TSS41440	P23013
chr3	Cufflinks	exon	172480700	172480762	+	XLOC 02154	TCONS 0010434	10	ECT2	NM 018098	NM 018098	=	TSS41440	P23013
chr3	Cufflinks	exon	172482068	172482290	+	XLOC 02154	TCONS 0010434	11	ECT2	NM 018098	NM 018098	=	TSS41440	P23013
chr3	Cufflinks	exon	172486797	172486933	+	XLOC 02154	TCONS 0010434	12	ECT2	NM 018098	NM 018098	=	TSS41440	P23013
chr3	Cufflinks	exon	172491693	172491812	+	XLOC 02154	TCONS 0010434	13	ECT2	NM 018098	NM 018098	=	TSS41440	P23013
chr3	Cufflinks	exon	172499953	172500021	+	XLOC 02154	TCONS 0010434	14	ECT2	NM 018098	NM 018098	=	TSS41440	P23013
chr3	Cufflinks	exon	172501589	172501699	+	XLOC 02154	TCONS 0010434	15	ECT2	NM 018098	NM 018098	=	TSS41440	P23013
chr3	Cufflinks	exon	172502497	172502593	+	XLOC 02154	TCONS 0010434	16	ECT2	NM 018098	NM 018098	=	TSS41440	P23013
chr3	Cufflinks	exon	172504283	172504364	+	XLOC 02154	TCONS 0010434	17	ECT2	NM 018098	NM 018098	=	TSS41440	P23013
chr3	Cufflinks	exon	172520406	172520484	+	XLOC 02154	TCONS 0010434	18	ECT2	NM 018098	NM 018098	=	TSS41440	P23013
chr3	Cufflinks	exon	172520651	172520770	+	XLOC 02154	TCONS 0010434	19	ECT2	NM 018098	NM 018098	=	TSS41440	P23013
chr3	Cufflinks	exon	172523521	172523659	+	XLOC 02154	TCONS 0010434	20	ECT2	NM 018098	NM 018098	=	TSS41440	P23013
chr3	Cufflinks	exon	172525560	172525714	+	XLOC 02154	TCONS 0010434	21	ECT2	NM 018098	NM 018098	=	TSS41440	P23013
chr3	Cufflinks	exon	172533394	172533501	+	XLOC 02154	TCONS 0010434	22	ECT2	NM 018098	NM 018098	=	TSS41440	P23013
chr3	Cufflinks	exon	172534481	172534627	+	XLOC 02154	TCONS 0010434	23	ECT2	NM 018098	NM 018098	=	TSS41440	P23013
chr3	Cufflinks	exon	172537938	172539264	+	XLOC 02154	TCONS 0010434	24	ECT2	NM 018098	NM 018098	=	TSS41440	P23013
chr3	Cufflinks	exon	42824400	42826812	-	XLOC 02181	TCONS 0010571	1	HIGD1A	NM 014056	NM 014056	=	TSS41978	P23319
chr3	Cufflinks	exon	42827520	42827654	-	XLOC 02181	TCONS 0010571	2	HIGD1A	NM 014056	NM 014056	=	TSS41978	P23319
chr3	Cufflinks	exon	42835649	42835767	-	XLOC 02181	TCONS 0010571	3	HIGD1A	NM 014056	NM 014056	=	TSS41978	P23319
chr3	Cufflinks	exon	42845928	42846027	-	XLOC 02181	TCONS 0010571	4	HIGD1A	NM 014056	NM 014056	=	TSS41978	P23319
chr3	Cufflinks	exon	48894356	48895205	-	XLOC 02186	TCONS 0010612	1	SLC25A20	NM 000387	NM 000387	=	TSS42102	P23407
chr3	Cufflinks	exon	48895940	48896064	-	XLOC 02186	TCONS 0010612	2	SLC25A20	NM 000387	NM 000387	=	TSS42102	P23407
chr3	Cufflinks	exon	48896525	48896634	-	XLOC 02186	TCONS 0010612	3	SLC25A20	NM 000387	NM 000387	=	TSS42102	P23407
chr3	Cufflinks	exon	48896988	48897060	-	XLOC 02186	TCONS 0010612	4	SLC25A20	NM 000387	NM 000387	=	TSS42102	P23407
chr3	Cufflinks	exon	48899975	48900092	-	XLOC 02186	TCONS 0010612	5	SLC25A20	NM 000387	NM 000387	=	TSS42102	P23407
chr3	Cufflinks	exon	48916791	48916881	-	XLOC 02186	TCONS 0010612	6	SLC25A20	NM 000387	NM 000387	=	TSS42102	P23407
chr3	Cufflinks	exon	48921430	48921557	-	XLOC 02186	TCONS 0010612	7	SLC25A20	NM 000387	NM 000387	=	TSS42102	P23407
chr3	Cufflinks	exon	48929413	48929505	-	XLOC 02186	TCONS 0010612	8	SLC25A20	NM 000387	NM 000387	=	TSS42102	P23407
chr3	Cufflinks	exon	48936123	48936426	-	XLOC 02186	TCONS 0010612	9	SLC25A20	NM 000387	NM 000387	=	TSS42102	P23407
chr3	Cufflinks	exon	127407905	127411166	-	XLOC 02213	TCONS 0010786	1	MGLL	NM 001003794	NM 001003794	=	TSS42704	P23757
chr3	Cufflinks	exon	127413818	127414033	-	XLOC 02213	TCONS 0010786	2	MGLL	NM 001003794	NM 001003794	=	TSS42704	P23757
chr3	Cufflinks	exon	127429419	127429508	-	XLOC 02213	TCONS 0010786	3	MGLL	NM 001003794	NM 001003794	=	TSS42704	P23757
chr3	Cufflinks	exon	127439896	127440006	-	XLOC 02213	TCONS 0010786	4	MGLL	NM 001003794	NM 001003794	=	TSS42704	P23757
chr3	Cufflinks	exon	127441273	127441409	-	XLOC 02213	TCONS 0010786	5	MGLL	NM 001003794	NM 001003794	=	TSS42704	P23757
chr3	Cufflinks	exon	127500632	127500738	-	XLOC 02213	TCONS 0010786	6	MGLL	NM 001003794	NM 001003794	=	TSS42704	P23757
chr3	Cufflinks	exon	127540537	127540681	-	XLOC 02213	TCONS 0010786	7	MGLL	NM 001003794	NM 001003794	=	TSS42704	P23757
chr3	Cufflinks	exon	127541927	127542093	-	XLOC 02213	TCONS 0010786	8	MGLL	NM 001003794	NM 001003794	=	TSS42704	P23757
chr3	Cufflinks	exon	170582665	170584313	-	XLOC 02228	TCONS 0010871	1	RPL22L1	NM 001099645	NM 001099645	=	TSS43022	P23941
chr3	Cufflinks	exon	170585802	170585923	-	XLOC 02228	TCONS 0010871	2	RPL22L1	NM 001099645	NM 001099645	=	TSS43022	P23941
chr3	Cufflinks	exon	170586087	170586179	-	XLOC 02228	TCONS 0010871	3	RPL22L1	NM 001099645	NM 001099645	=	TSS43022	P23941
chr3	Cufflinks	exon	170587948	170588045	-	XLOC 02228	TCONS 0010871	4	RPL22L1	NM 001099645	NM 001099645	=	TSS43022	P23941
chr3	Cufflinks	exon	155705122	155706910	.	XLOC 02264	TCONS 0010970	1		CUFF.28683.1		u	TSS43550	

chr4	Cufflinks	exon	57302269	57302494	+	XLOC 02296	TCONS 0011101	1	PAICS	NM 001079524	NM 001079524	=	TSS44091	P24353
chr4	Cufflinks	exon	57307829	57308026	+	XLOC 02296	TCONS 0011101	2	PAICS	NM 001079524	NM 001079524	=	TSS44091	P24353
chr4	Cufflinks	exon	57312861	57313039	+	XLOC 02296	TCONS 0011101	3	PAICS	NM 001079524	NM 001079524	=	TSS44091	P24353
chr4	Cufflinks	exon	57314584	57314763	+	XLOC 02296	TCONS 0011101	4	PAICS	NM 001079524	NM 001079524	=	TSS44091	P24353
chr4	Cufflinks	exon	57314876	57314989	+	XLOC 02296	TCONS 0011101	5	PAICS	NM 001079524	NM 001079524	=	TSS44091	P24353
chr4	Cufflinks	exon	57316785	57316868	+	XLOC 02296	TCONS 0011101	6	PAICS	NM 001079524	NM 001079524	=	TSS44091	P24353
chr4	Cufflinks	exon	57318038	57318218	+	XLOC 02296	TCONS 0011101	7	PAICS	NM 001079524	NM 001079524	=	TSS44091	P24353
chr4	Cufflinks	exon	57319769	57319927	+	XLOC 02296	TCONS 0011101	8	PAICS	NM 001079524	NM 001079524	=	TSS44091	P24353
chr4	Cufflinks	exon	57325538	57327534	+	XLOC 02296	TCONS 0011101	9	PAICS	NM 001079524	NM 001079524	=	TSS44091	P24353
chr4	Cufflinks	exon	88896802	88896952	+	XLOC 02307	TCONS 0011147	1	SPP1	NM 001040058	NM 001040058	=	TSS44305	P24490
chr4	Cufflinks	exon	88898034	88898101	+	XLOC 02307	TCONS 0011147	2	SPP1	NM 001040058	NM 001040058	=	TSS44305	P24490
chr4	Cufflinks	exon	88898211	88898249	+	XLOC 02307	TCONS 0011147	3	SPP1	NM 001040058	NM 001040058	=	TSS44305	P24490
chr4	Cufflinks	exon	88901198	88901278	+	XLOC 02307	TCONS 0011147	4	SPP1	NM 001040058	NM 001040058	=	TSS44305	P24490
chr4	Cufflinks	exon	88901545	88901586	+	XLOC 02307	TCONS 0011147	5	SPP1	NM 001040058	NM 001040058	=	TSS44305	P24490
chr4	Cufflinks	exon	88902627	88902950	+	XLOC 02307	TCONS 0011147	6	SPP1	NM 001040058	NM 001040058	=	TSS44305	P24490
chr4	Cufflinks	exon	88903644	88904563	+	XLOC 02307	TCONS 0011147	7	SPP1	NM 001040058	NM 001040058	=	TSS44305	P24490
chr4	Cufflinks	exon	1813206	1816300	-	XLOC 02334	TCONS 0011298	1	LETM1	NM 012318	NM 012318	=	TSS44905	P24793
chr4	Cufflinks	exon	1817391	1817529	-	XLOC 02334	TCONS 0011298	2	LETM1	NM 012318	NM 012318	=	TSS44905	P24793
chr4	Cufflinks	exon	1818454	1818641	-	XLOC 02334	TCONS 0011298	3	LETM1	NM 012318	NM 012318	=	TSS44905	P24793
chr4	Cufflinks	exon	1821065	1821199	-	XLOC 02334	TCONS 0011298	4	LETM1	NM 012318	NM 012318	=	TSS44905	P24793
chr4	Cufflinks	exon	1823908	1824039	-	XLOC 02334	TCONS 0011298	5	LETM1	NM 012318	NM 012318	=	TSS44905	P24793
chr4	Cufflinks	exon	1824715	1824858	-	XLOC 02334	TCONS 0011298	6	LETM1	NM 012318	NM 012318	=	TSS44905	P24793
chr4	Cufflinks	exon	1825371	1825502	-	XLOC 02334	TCONS 0011298	7	LETM1	NM 012318	NM 012318	=	TSS44905	P24793
chr4	Cufflinks	exon	1827291	1827410	-	XLOC 02334	TCONS 0011298	8	LETM1	NM 012318	NM 012318	=	TSS44905	P24793
chr4	Cufflinks	exon	1834471	1834674	-	XLOC 02334	TCONS 0011298	9	LETM1	NM 012318	NM 012318	=	TSS44905	P24793
chr4	Cufflinks	exon	1836572	1836709	-	XLOC 02334	TCONS 0011298	10	LETM1	NM 012318	NM 012318	=	TSS44905	P24793
chr4	Cufflinks	exon	1838156	1838299	-	XLOC 02334	TCONS 0011298	11	LETM1	NM 012318	NM 012318	=	TSS44905	P24793
chr4	Cufflinks	exon	1843074	1843524	-	XLOC 02334	TCONS 0011298	12	LETM1	NM 012318	NM 012318	=	TSS44905	P24793
chr4	Cufflinks	exon	1850876	1850936	-	XLOC 02334	TCONS 0011298	13	LETM1	NM 012318	NM 012318	=	TSS44905	P24793
chr4	Cufflinks	exon	1857596	1857974	-	XLOC 02334	TCONS 0011298	14	LETM1	NM 012318	NM 012318	=	TSS44905	P24793
chr4	Cufflinks	exon	6322305	6325320	-	XLOC 02336	TCONS 0011312	1	PPP2R2C	NM 181876	NM 181876	=	TSS44963	P24828
chr4	Cufflinks	exon	6330989	6331080	-	XLOC 02336	TCONS 0011312	2	PPP2R2C	NM 181876	NM 181876	=	TSS44963	P24828
chr4	Cufflinks	exon	6335289	6335458	-	XLOC 02336	TCONS 0011312	3	PPP2R2C	NM 181876	NM 181876	=	TSS44963	P24828
chr4	Cufflinks	exon	6349573	6349737	-	XLOC 02336	TCONS 0011312	4	PPP2R2C	NM 181876	NM 181876	=	TSS44963	P24828
chr4	Cufflinks	exon	6374250	6374427	-	XLOC 02336	TCONS 0011312	5	PPP2R2C	NM 181876	NM 181876	=	TSS44963	P24828
chr4	Cufflinks	exon	6377546	6377658	-	XLOC 02336	TCONS 0011312	6	PPP2R2C	NM 181876	NM 181876	=	TSS44963	P24828
chr4	Cufflinks	exon	6380134	6380299	-	XLOC 02336	TCONS 0011312	7	PPP2R2C	NM 181876	NM 181876	=	TSS44963	P24828
chr4	Cufflinks	exon	6382724	6382821	-	XLOC 02336	TCONS 0011312	8	PPP2R2C	NM 181876	NM 181876	=	TSS44963	P24828
chr4	Cufflinks	exon	6383504	6383597	-	XLOC 02336	TCONS 0011312	9	PPP2R2C	NM 181876	NM 181876	=	TSS44963	P24828
chr4	Cufflinks	exon	76834809	76835491	-	XLOC 02354	TCONS 0011403	1	NAAA	NM 014435	NM 014435	=	TSS45295	P25058
chr4	Cufflinks	exon	76836021	76836138	-	XLOC 02354	TCONS 0011403	2	NAAA	NM 014435	NM 014435	=	TSS45295	P25058
chr4	Cufflinks	exon	76839914	76839942	-	XLOC 02354	TCONS 0011403	3	NAAA	NM 014435	NM 014435	=	TSS45295	P25058
chr4	Cufflinks	exon	76841062	76841128	-	XLOC 02354	TCONS 0011403	4	NAAA	NM 014435	NM 014435	=	TSS45295	P25058
chr4	Cufflinks	exon	76841891	76841953	-	XLOC 02354	TCONS 0011403	5	NAAA	NM 014435	NM 014435	=	TSS45295	P25058
chr4	Cufflinks	exon	76842104	76842276	-	XLOC 02354	TCONS 0011403	6	NAAA	NM 014435	NM 014435	=	TSS45295	P25058
chr4	Cufflinks	exon	76846888	76846964	-	XLOC 02354	TCONS 0011403	7	NAAA	NM 014435	NM 014435	=	TSS45295	P25058
chr4	Cufflinks	exon	76852367	76852457	-	XLOC 02354	TCONS 0011403	8	NAAA	NM 014435	NM 014435	=	TSS45295	P25058
chr4	Cufflinks	exon	76857262	76857388	-	XLOC 02354	TCONS 0011403	9	NAAA	NM 014435	NM 014435	=	TSS45295	P25058
chr4	Cufflinks	exon	76861154	76861318	-	XLOC 02354	TCONS 0011403	10	NAAA	NM 014435	NM 014435	=	TSS45295	P25058
chr4	Cufflinks	exon	76861897	76862166	-	XLOC 02354	TCONS 0011403	11	NAAA	NM 014435	NM 014435	=	TSS45295	P25058
chr4	Cufflinks	exon	120980579	120981445	-	XLOC 02368	TCONS 0011486	1	MAD2L1	NM 002358	NM 002358	=	TSS45586	P25246
chr4	Cufflinks	exon	120982029	120982132	-	XLOC 02368	TCONS 0011486	2	MAD2L1	NM 002358	NM 002358	=	TSS45586	P25246
chr4	Cufflinks	exon	120983130	120983250	-	XLOC 02368	TCONS 0011486	3	MAD2L1	NM 002358	NM 002358	=	TSS45586	P25246
chr4	Cufflinks	exon	120986827	120986973	-	XLOC 02368	TCONS 0011486	4	MAD2L1	NM 002358	NM 002358	=	TSS45586	P25246
chr4	Cufflinks	exon	120987817	120988013	-	XLOC 02368	TCONS 0011486	5	MAD2L1	NM 002358	NM 002358	=	TSS45586	P25246
chr4	Cufflinks	exon	175411328	175413245	-	XLOC 02381	TCONS 0011548	1	HPGD	NM 000860	NM 000860	=	TSS45834	P25390
chr4	Cufflinks	exon	175414302	175414465	-	XLOC 02381	TCONS 0011548	2	HPGD	NM 000860	NM 000860	=	TSS45834	P25390
chr4	Cufflinks	exon	175416699	175416775	-	XLOC 02381	TCONS 0011548	3	HPGD	NM 000860	NM 000860	=	TSS45834	P25390
chr4	Cufflinks	exon	175429847	175429943	-	XLOC 02381	TCONS 0011548	4	HPGD	NM 000860	NM 000860	=	TSS45834	P25390
chr4	Cufflinks	exon	175439122	175439228	-	XLOC 02381	TCONS 0011548	5	HPGD	NM 000860	NM 000860	=	TSS45834	P25390
chr4	Cufflinks	exon	175443095	175443218	-	XLOC 02381	TCONS 0011548	6	HPGD	NM 000860	NM 000860	=	TSS45834	P25390
chr4	Cufflinks	exon	175443510	175444049	-	XLOC 02381	TCONS 0011548	7	HPGD	NM 000860	NM 000860	=	TSS45834	P25390
chr4	Cufflinks	exon	149555491	149558069	.	XLOC 02406	TCONS 0011589	1		CUFF.30946.1		u	TSS46122	
chr5	Cufflinks	exon	54398474	54398580	+	XLOC 02429	TCONS 0011660	1	GZMA	NM 006144	NM 006144	=	TSS46473	P25575
chr5	Cufflinks	exon	54401302	54401446	+	XLOC 02429	TCONS 0011660	2	GZMA	NM 006144	NM 006144	=	TSS46473	P25575
chr5	Cufflinks	exon	54403622	54403763	+	XLOC 02429	TCONS 0011660	3	GZMA	NM 006144	NM 006144	=	TSS46473	P25575
chr5	Cufflinks	exon	54403953	54404222	+	XLOC 02429	TCONS 0011660	4	GZMA	NM 006144	NM 006144	=	TSS46473	P25575
chr5	Cufflinks	exon	54405849	54406080	+	XLOC 02429	TCONS 0011660	5	GZMA	NM 006144	NM 006144	=	TSS46473	P25575
chr5	Cufflinks	exon	102594442	102594597	+	XLOC 02444	TCONS 0011749	1	C5orf30	NM 033211	NM 033211	=	TSS46781	P25784
chr5	Cufflinks	exon	102601609	102601698	+	XLOC 02444	TCONS 0011749	2	C5orf30	NM 033211	NM 033211	=	TSS46781	P25784
chr5	Cufflinks	exon	102611598	102614361	+	XLOC 02444	TCONS 0011749	3	C5orf30	NM 033211	NM 033211	=	TSS46781	P25784
chr5	Cufflinks	exon	125800787	125800912	+	XLOC 02449	TCONS 0011777	1	GRAMD3	NM 001146322	NM 001146322	=	TSS46896	P25853
chr5	Cufflinks	exon	125801069	125801237	+	XLOC 02449	TCONS 0011777	2	GRAMD3	NM 001146322	NM 001146322	=	TSS46896	P25853
chr5	Cufflinks	exon	125805369	125805480	+	XLOC 02449	TCONS 0011777	3	GRAMD3	NM 001146322	NM 001146322	=	TSS46896	P25853
chr5	Cufflinks	exon	125807930	125807996	+	XLOC 02449	TCONS 0011777	4	GRAMD3	NM 001146322	NM 001146322	=	TSS46896	P25853
chr5	Cufflinks	exon	125808957	125809060	+	XLOC 02449	TCONS 0011777	5	GRAMD3	NM 001146322	NM 001146322	=	TSS46896	P25853
chr5	Cufflinks	exon	125813384	125813479	+	XLOC 02449	TCONS 0011777	6	GRAMD3	NM 001146322	NM 001146322	=	TSS46896	P25853
chr5	Cufflinks	exon	125816148	125816219	+	XLOC 02449	TCONS 0011777	7	GRAMD3	NM 001146322	NM 001146322	=	TSS46896	P25853
chr5	Cufflinks	exon	125816319	125816399	+	XLOC 02449	TCONS 0011777	8	GRAMD3	NM 001146322	NM 001146322	=	TSS46896	P25853
chr5	Cufflinks	exon	125819155	125819266	+	XLOC 02449	TCONS 0011777	9	GRAMD3	NM 001146322	NM 001146322	=	TSS46896	P25853
chr5	Cufflinks	exon	125820094	125820216	+	XLOC 02449	TCONS 0011777	10	GRAMD3	NM 001146322	NM 001146322	=	TSS46896	P25853
chr5	Cufflinks	exon	125821378	125821465	+	XLOC 02449	TCONS 0011777	11	GRAMD3	NM 001146322	NM 001146322	=	TSS46896	P25853
chr5	Cufflinks	exon	125822565	125822669	+	XLOC 02449	TCONS 0011777	12	GRAMD3	NM 001146322	NM 001146322	=	TSS46896	P25853
chr5	Cufflinks	exon	125824491	125824584	+	XLOC 02449	TCONS 0011777	13	GRAMD3	NM 001146322	NM 001146322	=	TSS46896	P25853
chr5	Cufflinks	exon	125828607	125829853	+	XLOC 02449	TCONS 0011777	14	GRAMD3	NM 001146322	NM 001146322	=	TSS46896	P25853
chr5	Cufflinks	exon	127419483	127420402	+	XLOC 02450	TCONS 0011780	1	SLC12A2	NM 001046	NM 001046	=	TSS46912	P25867
chr5	Cufflinks	exon	127448506	127448625	+	XLOC 02450	TCONS 0011780	2	SLC12A2	NM 001046	NM 001046	=	TSS46912	P25867
chr5	Cufflinks	exon	127449904	127449979	+	XLOC 02450	TCONS 0011780	3	SLC12A2	NM 001046	NM 001046	=	TSS46912	P25867
chr5	Cufflinks	exon	127450278	127450373	+	XLOC 02450	TCONS 0011780	4	SLC12A2	NM 001046	NM 001046	=	TSS46912	P25867
chr5	Cufflinks	exon	127466759	127466898	+	XLOC 02450	TCONS 0011780	5	SLC12A2	NM 001046	NM 001046	=	TSS46912	P25867
chr5	Cufflinks	exon	127469857	127469967	+	XLOC 02450	TCONS 0011780	6	SLC12A2	NM 001046	NM 001046	=	TSS46912	P25867
chr5	Cufflinks	exon	127471392	127471500	+	XLOC 02450	TCONS 0011780	7	SLC12A2	NM 001046	NM 001046	=	TSS46912	P25867
chr5	Cufflinks	exon	127474289	127474416	+	XLOC 02450	TCONS 0011780	8	SLC12A2	NM 001046	NM 001046	=	TSS46912	P25867
chr5	Cufflinks	exon	127474516	127474600	+	XLOC 02450	TCONS 0011780	9	SLC12A2	NM 001046	NM 001046	=	TSS46912	P25867
chr5	Cufflinks	exon	127477522	127477673	+	XLOC 02450	TCONS 0011780	10	SLC12A2	NM 001046	NM 001046	=	TSS46912	P25867
chr5	Cufflinks	exon	127483314	127483421	+	XLOC 02450	TCONS 0011780	11	SLC12A2	NM 001046	NM 001046	=	TSS46912	P25867
chr5	Cufflinks	exon	127484446	127484569	+	XLOC 02450	TCONS 0011780	12	SLC12A2	NM 001046	NM 001046	=	TSS46912	P25867
chr5	Cufflinks	exon	127485689	127485790	+	XLOC 02450	TCONS 0011780	13	SLC12A2	NM 001046	NM 001046	=	TSS46912	P25867
chr5	Cufflinks	exon	127486933	127487088	+	XLOC 02450	TCONS 0011780	14	SLC12A2	NM 001046	NM 001046	=	TSS46912	P25867
chr5	Cufflinks	exon	127488398	127488497	+	XLOC 02450	TCONS 0011780	15	SLC12A2	NM 001046	NM 001046	=	TSS46912	P25867

chr5	Cufflinks	exon	127493745	127493856	+	XLOC 02450	TCONS 0011780	16	SLC12A2	NM 001046	NM 001046	=	TSS46912	P25867
chr5	Cufflinks	exon	127497352	127497492	+	XLOC 02450	TCONS 0011780	17	SLC12A2	NM 001046	NM 001046	=	TSS46912	P25867
chr5	Cufflinks	exon	127503453	127503559	+	XLOC 02450	TCONS 0011780	18	SLC12A2	NM 001046	NM 001046	=	TSS46912	P25867
chr5	Cufflinks	exon	127507359	127507438	+	XLOC 02450	TCONS 0011780	19	SLC12A2	NM 001046	NM 001046	=	TSS46912	P25867
chr5	Cufflinks	exon	127510233	127510358	+	XLOC 02450	TCONS 0011780	20	SLC12A2	NM 001046	NM 001046	=	TSS46912	P25867
chr5	Cufflinks	exon	127512797	127512844	+	XLOC 02450	TCONS 0011780	21	SLC12A2	NM 001046	NM 001046	=	TSS46912	P25867
chr5	Cufflinks	exon	127514259	127514381	+	XLOC 02450	TCONS 0011780	22	SLC12A2	NM 001046	NM 001046	=	TSS46912	P25867
chr5	Cufflinks	exon	127516575	127516686	+	XLOC 02450	TCONS 0011780	23	SLC12A2	NM 001046	NM 001046	=	TSS46912	P25867
chr5	Cufflinks	exon	127518547	127518633	+	XLOC 02450	TCONS 0011780	24	SLC12A2	NM 001046	NM 001046	=	TSS46912	P25867
chr5	Cufflinks	exon	127520058	127520193	+	XLOC 02450	TCONS 0011780	25	SLC12A2	NM 001046	NM 001046	=	TSS46912	P25867
chr5	Cufflinks	exon	127520481	127520548	+	XLOC 02450	TCONS 0011780	26	SLC12A2	NM 001046	NM 001046	=	TSS46912	P25867
chr5	Cufflinks	exon	127522188	127525380	+	XLOC 02450	TCONS 0011780	27	SLC12A2	NM 001046	NM 001046	=	TSS46912	P25867
chr5	Cufflinks	exon	175819456	175819946	-	XLOC 02537	TCONS 0012225	1	CLTB	NM 001834	NM 001834	=	TSS48686	P26955
chr5	Cufflinks	exon	175824608	175824719	-	XLOC 02537	TCONS 0012225	2	CLTB	NM 001834	NM 001834	=	TSS48686	P26955
chr5	Cufflinks	exon	175824931	175825048	-	XLOC 02537	TCONS 0012225	3	CLTB	NM 001834	NM 001834	=	TSS48686	P26955
chr5	Cufflinks	exon	175837258	175837304	-	XLOC 02537	TCONS 0012225	4	CLTB	NM 001834	NM 001834	=	TSS48686	P26955
chr5	Cufflinks	exon	175843178	175843570	-	XLOC 02537	TCONS 0012225	5	CLTB	NM 001834	NM 001834	=	TSS48686	P26955
chr5	Cufflinks	exon	17353814	17354498	.	XLOC 02546	TCONS 0012264	1		CUFF.31599.1		u	TSS48841	
chr6	Cufflinks	exon	123110194	123110603	+	XLOC 02626	TCONS 0012549	1	SMPDL3A	NM 006714	NM 006714	=	TSS50142	P27692
chr6	Cufflinks	exon	123116822	123117035	+	XLOC 02626	TCONS 0012549	2	SMPDL3A	NM 006714	NM 006714	=	TSS50142	P27692
chr6	Cufflinks	exon	123117969	123118113	+	XLOC 02626	TCONS 0012549	3	SMPDL3A	NM 006714	NM 006714	=	TSS50142	P27692
chr6	Cufflinks	exon	123122455	123122551	+	XLOC 02626	TCONS 0012549	4	SMPDL3A	NM 006714	NM 006714	=	TSS50142	P27692
chr6	Cufflinks	exon	123124809	123124978	+	XLOC 02626	TCONS 0012549	5	SMPDL3A	NM 006714	NM 006714	=	TSS50142	P27692
chr6	Cufflinks	exon	123126054	123126234	+	XLOC 02626	TCONS 0012549	6	SMPDL3A	NM 006714	NM 006714	=	TSS50142	P27692
chr6	Cufflinks	exon	123127378	123127502	+	XLOC 02626	TCONS 0012549	7	SMPDL3A	NM 006714	NM 006714	=	TSS50142	P27692
chr6	Cufflinks	exon	123130236	123130864	+	XLOC 02626	TCONS 0012549	8	SMPDL3A	NM 006714	NM 006714	=	TSS50142	P27692
chr6	Cufflinks	exon	24650205	24651297	-	XLOC 02653	TCONS 0012678	1	TDP2	NM 016614	NM 016614	=	TSS50705	P28009
chr6	Cufflinks	exon	24653211	24653381	-	XLOC 02653	TCONS 0012678	2	TDP2	NM 016614	NM 016614	=	TSS50705	P28009
chr6	Cufflinks	exon	24654640	24654758	-	XLOC 02653	TCONS 0012678	3	TDP2	NM 016614	NM 016614	=	TSS50705	P28009
chr6	Cufflinks	exon	24658040	24658131	-	XLOC 02653	TCONS 0012678	4	TDP2	NM 016614	NM 016614	=	TSS50705	P28009
chr6	Cufflinks	exon	24658789	24658962	-	XLOC 02653	TCONS 0012678	5	TDP2	NM 016614	NM 016614	=	TSS50705	P28009
chr6	Cufflinks	exon	24666754	24666839	-	XLOC 02653	TCONS 0012678	6	TDP2	NM 016614	NM 016614	=	TSS50705	P28009
chr6	Cufflinks	exon	24666926	24667115	-	XLOC 02653	TCONS 0012678	7	TDP2	NM 016614	NM 016614	=	TSS50705	P28009
chr6	Cufflinks	exon	39156747	39159531	-	XLOC 02674	TCONS 0012746	1	KCNK5	NM 003740	NM 003740	=	TSS51029	P28278
chr6	Cufflinks	exon	39161945	39162113	-	XLOC 02674	TCONS 0012746	2	KCNK5	NM 003740	NM 003740	=	TSS51029	P28278
chr6	Cufflinks	exon	39162370	39162536	-	XLOC 02674	TCONS 0012746	3	KCNK5	NM 003740	NM 003740	=	TSS51029	P28278
chr6	Cufflinks	exon	39163652	39163763	-	XLOC 02674	TCONS 0012746	4	KCNK5	NM 003740	NM 003740	=	TSS51029	P28278
chr6	Cufflinks	exon	39196702	39197251	-	XLOC 02674	TCONS 0012746	5	KCNK5	NM 003740	NM 003740	=	TSS51029	P28278
chr6	Cufflinks	exon	52128812	52129584	-	XLOC 02682	TCONS 0012787	1	MCM3	NM 002388	NM 002388	=	TSS51194	P28396
chr6	Cufflinks	exon	52130873	52130942	-	XLOC 02682	TCONS 0012787	2	MCM3	NM 002388	NM 002388	=	TSS51194	P28396
chr6	Cufflinks	exon	52131409	52131494	-	XLOC 02682	TCONS 0012787	3	MCM3	NM 002388	NM 002388	=	TSS51194	P28396
chr6	Cufflinks	exon	52132663	52132766	-	XLOC 02682	TCONS 0012787	4	MCM3	NM 002388	NM 002388	=	TSS51194	P28396
chr6	Cufflinks	exon	52133884	52134024	-	XLOC 02682	TCONS 0012787	5	MCM3	NM 002388	NM 002388	=	TSS51194	P28396
chr6	Cufflinks	exon	52137099	52137249	-	XLOC 02682	TCONS 0012787	6	MCM3	NM 002388	NM 002388	=	TSS51194	P28396
chr6	Cufflinks	exon	52138028	52138154	-	XLOC 02682	TCONS 0012787	7	MCM3	NM 002388	NM 002388	=	TSS51194	P28396
chr6	Cufflinks	exon	52138540	52138714	-	XLOC 02682	TCONS 0012787	8	MCM3	NM 002388	NM 002388	=	TSS51194	P28396
chr6	Cufflinks	exon	52141066	52141274	-	XLOC 02682	TCONS 0012787	9	MCM3	NM 002388	NM 002388	=	TSS51194	P28396
chr6	Cufflinks	exon	52141865	52141996	-	XLOC 02682	TCONS 0012787	10	MCM3	NM 002388	NM 002388	=	TSS51194	P28396
chr6	Cufflinks	exon	52142333	52142486	-	XLOC 02682	TCONS 0012787	11	MCM3	NM 002388	NM 002388	=	TSS51194	P28396
chr6	Cufflinks	exon	52143540	52143648	-	XLOC 02682	TCONS 0012787	12	MCM3	NM 002388	NM 002388	=	TSS51194	P28396
chr6	Cufflinks	exon	52144159	52144397	-	XLOC 02682	TCONS 0012787	13	MCM3	NM 002388	NM 002388	=	TSS51194	P28396
chr6	Cufflinks	exon	52146843	52146973	-	XLOC 02682	TCONS 0012787	14	MCM3	NM 002388	NM 002388	=	TSS51194	P28396
chr6	Cufflinks	exon	52147451	52147659	-	XLOC 02682	TCONS 0012787	15	MCM3	NM 002388	NM 002388	=	TSS51194	P28396
chr6	Cufflinks	exon	52148092	52148204	-	XLOC 02682	TCONS 0012787	16	MCM3	NM 002388	NM 002388	=	TSS51194	P28396
chr6	Cufflinks	exon	52149395	52149679	-	XLOC 02682	TCONS 0012787	17	MCM3	NM 002388	NM 002388	=	TSS51194	P28396
chr7	Cufflinks	exon	5632436	5633399	+	XLOC 02875	TCONS 0013310	1	FSCN1	NM 003088	NM 003088	=	TSS53926	P30539
chr7	Cufflinks	exon	5642888	5643044	+	XLOC 02875	TCONS 0013310	2	FSCN1	NM 003088	NM 003088	=	TSS53926	P30539
chr7	Cufflinks	exon	5643127	5643248	+	XLOC 02875	TCONS 0013310	3	FSCN1	NM 003088	NM 003088	=	TSS53926	P30539
chr7	Cufflinks	exon	5643494	5643661	+	XLOC 02875	TCONS 0013310	4	FSCN1	NM 003088	NM 003088	=	TSS53926	P30539
chr7	Cufflinks	exon	5644903	5646287	+	XLOC 02875	TCONS 0013310	5	FSCN1	NM 003088	NM 003088	=	TSS53926	P30539
chr7	Cufflinks	exon	134212344	134212729	+	XLOC 02925	TCONS 0013603	1	AKR1B10	NM 020299	NM 020299	=	TSS55017	P31173
chr7	Cufflinks	exon	134215395	134215562	+	XLOC 02925	TCONS 0013603	2	AKR1B10	NM 020299	NM 020299	=	TSS55017	P31173
chr7	Cufflinks	exon	134216660	134216776	+	XLOC 02925	TCONS 0013603	3	AKR1B10	NM 020299	NM 020299	=	TSS55017	P31173
chr7	Cufflinks	exon	134217756	134217833	+	XLOC 02925	TCONS 0013603	4	AKR1B10	NM 020299	NM 020299	=	TSS55017	P31173
chr7	Cufflinks	exon	134221402	134221524	+	XLOC 02925	TCONS 0013603	5	AKR1B10	NM 020299	NM 020299	=	TSS55017	P31173
chr7	Cufflinks	exon	134221803	134221909	+	XLOC 02925	TCONS 0013603	6	AKR1B10	NM 020299	NM 020299	=	TSS55017	P31173
chr7	Cufflinks	exon	134222332	134222413	+	XLOC 02925	TCONS 0013603	7	AKR1B10	NM 020299	NM 020299	=	TSS55017	P31173
chr7	Cufflinks	exon	134222946	134223029	+	XLOC 02925	TCONS 0013603	8	AKR1B10	NM 020299	NM 020299	=	TSS55017	P31173
chr7	Cufflinks	exon	134223687	134223769	+	XLOC 02925	TCONS 0013603	9	AKR1B10	NM 020299	NM 020299	=	TSS55017	P31173
chr7	Cufflinks	exon	134225799	134226166	+	XLOC 02925	TCONS 0013603	10	AKR1B10	NM 020299	NM 020299	=	TSS55017	P31173
chr7	Cufflinks	exon	150549565	150549646	+	XLOC 02936	TCONS 0013654	1	AOC1	NM 001091	NM 001091	=	TSS55237	P31288
chr7	Cufflinks	exon	150553543	150555128	+	XLOC 02936	TCONS 0013654	2	AOC1	NM 001091	NM 001091	=	TSS55237	P31288
chr7	Cufflinks	exon	150555851	150556136	+	XLOC 02936	TCONS 0013654	3	AOC1	NM 001091	NM 001091	=	TSS55237	P31288
chr7	Cufflinks	exon	150557589	150557721	+	XLOC 02936	TCONS 0013654	4	AOC1	NM 001091	NM 001091	=	TSS55237	P31288
chr7	Cufflinks	exon	150558031	150558379	+	XLOC 02936	TCONS 0013654	5	AOC1	NM 001091	NM 001091	=	TSS55237	P31288
chr7	Cufflinks	exon	100400187	100401212	-	XLOC 02978	TCONS 0013911	1	EPHB4	NM 004444	NM 004444	=	TSS56203	P31790
chr7	Cufflinks	exon	100402788	100402943	-	XLOC 02978	TCONS 0013911	2	EPHB4	NM 004444	NM 004444	=	TSS56203	P31790
chr7	Cufflinks	exon	100403123	100403316	-	XLOC 02978	TCONS 0013911	3	EPHB4	NM 004444	NM 004444	=	TSS56203	P31790
chr7	Cufflinks	exon	100404042	100404191	-	XLOC 02978	TCONS 0013911	4	EPHB4	NM 004444	NM 004444	=	TSS56203	P31790
chr7	Cufflinks	exon	100404987	100405202	-	XLOC 02978	TCONS 0013911	5	EPHB4	NM 004444	NM 004444	=	TSS56203	P31790
chr7	Cufflinks	exon	100410369	100410616	-	XLOC 02978	TCONS 0013911	6	EPHB4	NM 004444	NM 004444	=	TSS56203	P31790
chr7	Cufflinks	exon	100410717	100410830	-	XLOC 02978	TCONS 0013911	7	EPHB4	NM 004444	NM 004444	=	TSS56203	P31790
chr7	Cufflinks	exon	100411274	100411338	-	XLOC 02978	TCONS 0013911	8	EPHB4	NM 004444	NM 004444	=	TSS56203	P31790
chr7	Cufflinks	exon	100411541	100411643	-	XLOC 02978	TCONS 0013911	9	EPHB4	NM 004444	NM 004444	=	TSS56203	P31790
chr7	Cufflinks	exon	100414814	100414979	-	XLOC 02978	TCONS 0013911	10	EPHB4	NM 004444	NM 004444	=	TSS56203	P31790
chr7	Cufflinks	exon	100416142	100416266	-	XLOC 02978	TCONS 0013911	11	EPHB4	NM 004444	NM 004444	=	TSS56203	P31790
chr7	Cufflinks	exon	100417179	100417511	-	XLOC 02978	TCONS 0013911	12	EPHB4	NM 004444	NM 004444	=	TSS56203	P31790
chr7	Cufflinks	exon	100417763	100417918	-	XLOC 02978	TCONS 0013911	13	EPHB4	NM 004444	NM 004444	=	TSS56203	P31790
chr7	Cufflinks	exon	100419893	100420289	-	XLOC 02978	TCONS 0013911	14	EPHB4	NM 004444	NM 004444	=	TSS56203	P31790
chr7	Cufflinks	exon	100421266	100421553	-	XLOC 02978	TCONS 0013911	15	EPHB4	NM 004444	NM 004444	=	TSS56203	P31790
chr7	Cufflinks	exon	100421825	100421895	-	XLOC 02978	TCONS 0013911	16	EPHB4	NM 004444	NM 004444	=	TSS56203	P31790
chr7	Cufflinks	exon	100424601	100425143	-	XLOC 02978	TCONS 0013911	17	EPHB4	NM 004444	NM 004444	=	TSS56203	P31790
chr7	Cufflinks	exon	131185021	131189267	-	XLOC 02990	TCONS 0013985	1	PODXL	NM 005397	NM 005397	=	TSS56473	P31937
chr7	Cufflinks	exon	131190627	131190794	-	XLOC 02990	TCONS 0013985	2	PODXL	NM 005397	NM 005397	=	TSS56473	P31937
chr7	Cufflinks	exon	131191019	131191080	-	XLOC 02990	TCONS 0013985	3	PODXL	NM 005397	NM 005397	=	TSS56473	P31937
chr7	Cufflinks	exon	131191338	131191485	-	XLOC 02990	TCONS 0013985	4	PODXL	NM 005397	NM 005397	=	TSS56473	P31937
chr7	Cufflinks	exon	131193710	131193787	-	XLOC 02990	TCONS 0013985	5	PODXL	NM 005397	NM 005397	=	TSS56473	P31937
chr7	Cufflinks	exon	131194124	131194344	-	XLOC 02990	TCONS 0013985	6	PODXL	NM 005397	NM 005397	=	TSS56473	P31937
chr7	Cufflinks	exon	131195587	131196192	-	XLOC 02990	TCONS 0013985	7	PODXL	NM 005397	NM 005397	=	TSS56473	P31937
chr7	Cufflinks	exon	131241019	131241376	-	XLOC 02990	TCONS 0013985	8	PODXL	NM 005397	NM 005397	=	TSS56473	P31937

chr7	Cufflinks	exon	87831028	87831030	-	XLOC 02970	TCONS 0013862	1	SRI	CUFF.37142.2	NM 003130	j	TSS56049	
chr7	Cufflinks	exon	87835720	87835819	-	XLOC 02970	TCONS 0013862	2	SRI	CUFF.37142.2	NM 003130	j	TSS56049	
chr7	Cufflinks	exon	87837822	87837880	-	XLOC 02970	TCONS 0013862	3	SRI	CUFF.37142.2	NM 003130	j	TSS56049	
chr7	Cufflinks	exon	87838654	87838767	-	XLOC 02970	TCONS 0013862	4	SRI	CUFF.37142.2	NM 003130	j	TSS56049	
chr7	Cufflinks	exon	87839298	87839445	-	XLOC 02970	TCONS 0013862	5	SRI	CUFF.37142.2	NM 003130	j	TSS56049	
chr7	Cufflinks	exon	87840197	87840240	-	XLOC 02970	TCONS 0013862	6	SRI	CUFF.37142.2	NM 003130	j	TSS56049	
chr7	Cufflinks	exon	87846437	87846506	-	XLOC 02970	TCONS 0013862	7	SRI	CUFF.37142.2	NM 003130	j	TSS56049	
chr7	Cufflinks	exon	87848174	87848257	-	XLOC 02970	TCONS 0013862	8	SRI	CUFF.37142.2	NM 003130	j	TSS56049	
chr7	Cufflinks	exon	87856224	87856308	-	XLOC 02970	TCONS 0013862	9	SRI	CUFF.37142.2	NM 003130	j	TSS56049	
chr8	Cufflinks	exon	24241798	24242105	+	XLOC 03043	TCONS 0014148	1	ADAMDEC1	NM 014479	NM 014479	=	TSS57291	P32200
chr8	Cufflinks	exon	24249775	24249893	+	XLOC 03043	TCONS 0014148	2	ADAMDEC1	NM 014479	NM 014479	=	TSS57291	P32200
chr8	Cufflinks	exon	24250775	24250851	+	XLOC 03043	TCONS 0014148	3	ADAMDEC1	NM 014479	NM 014479	=	TSS57291	P32200
chr8	Cufflinks	exon	24251582	24251660	+	XLOC 03043	TCONS 0014148	4	ADAMDEC1	NM 014479	NM 014479	=	TSS57291	P32200
chr8	Cufflinks	exon	24253233	24253309	+	XLOC 03043	TCONS 0014148	5	ADAMDEC1	NM 014479	NM 014479	=	TSS57291	P32200
chr8	Cufflinks	exon	24254783	24254969	+	XLOC 03043	TCONS 0014148	6	ADAMDEC1	NM 014479	NM 014479	=	TSS57291	P32200
chr8	Cufflinks	exon	24255196	24255258	+	XLOC 03043	TCONS 0014148	7	ADAMDEC1	NM 014479	NM 014479	=	TSS57291	P32200
chr8	Cufflinks	exon	24255993	24256064	+	XLOC 03043	TCONS 0014148	8	ADAMDEC1	NM 014479	NM 014479	=	TSS57291	P32200
chr8	Cufflinks	exon	24256387	24256553	+	XLOC 03043	TCONS 0014148	9	ADAMDEC1	NM 014479	NM 014479	=	TSS57291	P32200
chr8	Cufflinks	exon	24256906	24256987	+	XLOC 03043	TCONS 0014148	10	ADAMDEC1	NM 014479	NM 014479	=	TSS57291	P32200
chr8	Cufflinks	exon	24257683	24257813	+	XLOC 03043	TCONS 0014148	11	ADAMDEC1	NM 014479	NM 014479	=	TSS57291	P32200
chr8	Cufflinks	exon	24259428	24259605	+	XLOC 03043	TCONS 0014148	12	ADAMDEC1	NM 014479	NM 014479	=	TSS57291	P32200
chr8	Cufflinks	exon	24261516	24261601	+	XLOC 03043	TCONS 0014148	13	ADAMDEC1	NM 014479	NM 014479	=	TSS57291	P32200
chr8	Cufflinks	exon	24262805	24263526	+	XLOC 03043	TCONS 0014148	14	ADAMDEC1	NM 014479	NM 014479	=	TSS57291	P32200
chr8	Cufflinks	exon	67341263	67342968	+	XLOC 03057	TCONS 0014211	1	RRS1	NM 015169	NM 015169	=	TSS57537	P32363
chr8	Cufflinks	exon	6782216	6782449	-	XLOC 03083	TCONS 0014343	1	DEFA6	NM 001926	NM 001926	=	TSS58087	P32611
chr8	Cufflinks	exon	6783365	6783598	-	XLOC 03083	TCONS 0014343	2	DEFA6	NM 001926	NM 001926	=	TSS58087	P32611
chr8	Cufflinks	exon	6912822	6913065	-	XLOC 03085	TCONS 0014345	1	DEFA5	NM 021010	NM 021010	=	TSS58098	P32618
chr8	Cufflinks	exon	6914048	6914261	-	XLOC 03085	TCONS 0014345	2	DEFA5	NM 021010	NM 021010	=	TSS58098	P32618
chr8	Cufflinks	exon	29190579	29194928	-	XLOC 03097	TCONS 0014394	1	DUSP4	NM 001394	NM 001394	=	TSS58319	P32744
chr8	Cufflinks	exon	29195799	29196018	-	XLOC 03097	TCONS 0014394	2	DUSP4	NM 001394	NM 001394	=	TSS58319	P32744
chr8	Cufflinks	exon	29197615	29197760	-	XLOC 03097	TCONS 0014394	3	DUSP4	NM 001394	NM 001394	=	TSS58319	P32744
chr8	Cufflinks	exon	29207363	29208267	-	XLOC 03097	TCONS 0014394	4	DUSP4	NM 001394	NM 001394	=	TSS58319	P32744
chr8	Cufflinks	exon	119935796	119937001	-	XLOC 03123	TCONS 0014521	1	TNFRSF11B	NM 002546	NM 002546	=	TSS58828	P33049
chr8	Cufflinks	exon	119938733	119938957	-	XLOC 03123	TCONS 0014521	2	TNFRSF11B	NM 002546	NM 002546	=	TSS58828	P33049
chr8	Cufflinks	exon	119940977	119941168	-	XLOC 03123	TCONS 0014521	3	TNFRSF11B	NM 002546	NM 002546	=	TSS58828	P33049
chr8	Cufflinks	exon	119945170	119945539	-	XLOC 03123	TCONS 0014521	4	TNFRSF11B	NM 002546	NM 002546	=	TSS58828	P33049
chr8	Cufflinks	exon	119964031	119964383	-	XLOC 03123	TCONS 0014521	5	TNFRSF11B	NM 002546	NM 002546	=	TSS58828	P33049
chr8	Cufflinks	exon	82433965	82434350	.	XLOC 03151	TCONS 0014620	1		CUFF.39256.1		u	TSS59307	
chr9	Cufflinks	exon	33750819	33750850	+	XLOC 03167	TCONS 0014672	1	PRSS3	NM 001197098	NM 001197098	=	TSS59570	P33287
chr9	Cufflinks	exon	33796641	33796800	+	XLOC 03167	TCONS 0014672	2	PRSS3	NM 001197098	NM 001197098	=	TSS59570	P33287
chr9	Cufflinks	exon	33797827	33798080	+	XLOC 03167	TCONS 0014672	3	PRSS3	NM 001197098	NM 001197098	=	TSS59570	P33287
chr9	Cufflinks	exon	33798484	33798620	+	XLOC 03167	TCONS 0014672	4	PRSS3	NM 001197098	NM 001197098	=	TSS59570	P33287
chr9	Cufflinks	exon	33799026	33799229	+	XLOC 03167	TCONS 0014672	5	PRSS3	NM 001197098	NM 001197098	=	TSS59570	P33287
chr9	Cufflinks	exon	35673915	35674359	+	XLOC 03169	TCONS 0014681	1	CA9	NM 001216	NM 001216	=	TSS59605	P33315
chr9	Cufflinks	exon	35675535	35675564	+	XLOC 03169	TCONS 0014681	2	CA9	NM 001216	NM 001216	=	TSS59605	P33315
chr9	Cufflinks	exon	35675758	35675928	+	XLOC 03169	TCONS 0014681	3	CA9	NM 001216	NM 001216	=	TSS59605	P33315
chr9	Cufflinks	exon	35676061	35676203	+	XLOC 03169	TCONS 0014681	4	CA9	NM 001216	NM 001216	=	TSS59605	P33315
chr9	Cufflinks	exon	35676294	35676386	+	XLOC 03169	TCONS 0014681	5	CA9	NM 001216	NM 001216	=	TSS59605	P33315
chr9	Cufflinks	exon	35677787	35677853	+	XLOC 03169	TCONS 0014681	6	CA9	NM 001216	NM 001216	=	TSS59605	P33315
chr9	Cufflinks	exon	35679182	35679339	+	XLOC 03169	TCONS 0014681	7	CA9	NM 001216	NM 001216	=	TSS59605	P33315
chr9	Cufflinks	exon	35679851	35679995	+	XLOC 03169	TCONS 0014681	8	CA9	NM 001216	NM 001216	=	TSS59605	P33315
chr9	Cufflinks	exon	35680110	35680136	+	XLOC 03169	TCONS 0014681	9	CA9	NM 001216	NM 001216	=	TSS59605	P33315
chr9	Cufflinks	exon	35680750	35680831	+	XLOC 03169	TCONS 0014681	10	CA9	NM 001216	NM 001216	=	TSS59605	P33315
chr9	Cufflinks	exon	35680962	35681154	+	XLOC 03169	TCONS 0014681	11	CA9	NM 001216	NM 001216	=	TSS59605	P33315
chr9	Cufflinks	exon	80911991	80912186	+	XLOC 03179	TCONS 0014726	1	PSAT1	NM 058179	NM 058179	=	TSS59774	P33411
chr9	Cufflinks	exon	80915518	80915578	+	XLOC 03179	TCONS 0014726	2	PSAT1	NM 058179	NM 058179	=	TSS59774	P33411
chr9	Cufflinks	exon	80916870	80916939	+	XLOC 03179	TCONS 0014726	3	PSAT1	NM 058179	NM 058179	=	TSS59774	P33411
chr9	Cufflinks	exon	80919651	80919856	+	XLOC 03179	TCONS 0014726	4	PSAT1	NM 058179	NM 058179	=	TSS59774	P33411
chr9	Cufflinks	exon	80921230	80921402	+	XLOC 03179	TCONS 0014726	5	PSAT1	NM 058179	NM 058179	=	TSS59774	P33411
chr9	Cufflinks	exon	80923330	80923499	+	XLOC 03179	TCONS 0014726	6	PSAT1	NM 058179	NM 058179	=	TSS59774	P33411
chr9	Cufflinks	exon	80932592	80932720	+	XLOC 03179	TCONS 0014726	7	PSAT1	NM 058179	NM 058179	=	TSS59774	P33411
chr9	Cufflinks	exon	80942967	80943104	+	XLOC 03179	TCONS 0014726	8	PSAT1	NM 058179	NM 058179	=	TSS59774	P33411
chr9	Cufflinks	exon	80943897	80945009	+	XLOC 03179	TCONS 0014726	9	PSAT1	NM 058179	NM 058179	=	TSS59774	P33411
chr9	Cufflinks	exon	140446309	140446430	+	XLOC 03213	TCONS 0014930	1	MRPL41	NM 032477	NM 032477	=	TSS60516	P33860
chr9	Cufflinks	exon	140446526	140447007	+	XLOC 03213	TCONS 0014930	2	MRPL41	NM 032477	NM 032477	=	TSS60516	P33860
chr9	Cufflinks	exon	22002902	22006246	-	XLOC 03221	TCONS 0014970	1	CDKN2B	NM 004936	NM 004936	=	TSS60683	P33952
chr9	Cufflinks	exon	22008797	22009312	-	XLOC 03221	TCONS 0014970	2	CDKN2B	NM 004936	NM 004936	=	TSS60683	P33952
chr9	Cufflinks	exon	33441152	33442209	-	XLOC 03223	TCONS 0014983	1	AQP3	NM 004925	NM 004925	=	TSS60731	P33986
chr9	Cufflinks	exon	33442299	33442516	-	XLOC 03223	TCONS 0014983	2	AQP3	NM 004925	NM 004925	=	TSS60731	P33986
chr9	Cufflinks	exon	33442850	33442968	-	XLOC 03223	TCONS 0014983	3	AQP3	NM 004925	NM 004925	=	TSS60731	P33986
chr9	Cufflinks	exon	33443319	33443456	-	XLOC 03223	TCONS 0014983	4	AQP3	NM 004925	NM 004925	=	TSS60731	P33986
chr9	Cufflinks	exon	33443764	33443890	-	XLOC 03223	TCONS 0014983	5	AQP3	NM 004925	NM 004925	=	TSS60731	P33986
chr9	Cufflinks	exon	33447421	33447631	-	XLOC 03223	TCONS 0014983	6	AQP3	NM 004925	NM 004925	=	TSS60731	P33986
chr9	Cufflinks	exon	33461351	33462811	-	XLOC 03223	TCONS 0014983	1	NOL6	NM 022917	NM 022917	=	TSS60732	P33987
chr9	Cufflinks	exon	33463031	33463132	-	XLOC 03223	TCONS 0014983	2	NOL6	NM 022917	NM 022917	=	TSS60732	P33987
chr9	Cufflinks	exon	33463245	33463439	-	XLOC 03223	TCONS 0014983	3	NOL6	NM 022917	NM 022917	=	TSS60732	P33987
chr9	Cufflinks	exon	33463829	33463918	-	XLOC 03223	TCONS 0014983	4	NOL6	NM 022917	NM 022917	=	TSS60732	P33987
chr9	Cufflinks	exon	33464035	33464159	-	XLOC 03223	TCONS 0014983	5	NOL6	NM 022917	NM 022917	=	TSS60732	P33987
chr9	Cufflinks	exon	33464877	33464974	-	XLOC 03223	TCONS 0014983	6	NOL6	NM 022917	NM 022917	=	TSS60732	P33987
chr9	Cufflinks	exon	33465205	33465357	-	XLOC 03223	TCONS 0014983	7	NOL6	NM 022917	NM 022917	=	TSS60732	P33987
chr9	Cufflinks	exon	33465732	33465895	-	XLOC 03223	TCONS 0014983	8	NOL6	NM 022917	NM 022917	=	TSS60732	P33987
chr9	Cufflinks	exon	33466069	33466223	-	XLOC 03223	TCONS 0014983	9	NOL6	NM 022917	NM 022917	=	TSS60732	P33987
chr9	Cufflinks	exon	33466306	33466423	-	XLOC 03223	TCONS 0014983	10	NOL6	NM 022917	NM 022917	=	TSS60732	P33987
chr9	Cufflinks	exon	33466567	33466707	-	XLOC 03223	TCONS 0014983	11	NOL6	NM 022917	NM 022917	=	TSS60732	P33987
chr9	Cufflinks	exon	33466910	33466985	-	XLOC 03223	TCONS 0014983	12	NOL6	NM 022917	NM 022917	=	TSS60732	P33987
chr9	Cufflinks	exon	33467112	33467260	-	XLOC 03223	TCONS 0014983	13	NOL6	NM 022917	NM 022917	=	TSS60732	P33987
chr9	Cufflinks	exon	33467392	33467514	-	XLOC 03223	TCONS 0014983	14	NOL6	NM 022917	NM 022917	=	TSS60732	P33987
chr9	Cufflinks	exon	33467689	33467866	-	XLOC 03223	TCONS 0014983	15	NOL6	NM 022917	NM 022917	=	TSS60732	P33987
chr9	Cufflinks	exon	33468028	33468143	-	XLOC 03223	TCONS 0014983	16	NOL6	NM 022917	NM 022917	=	TSS60732	P33987
chr9	Cufflinks	exon	33468319	33468420	-	XLOC 03223	TCONS 0014983	17	NOL6	NM 022917	NM 022917	=	TSS60732	P33987
chr9	Cufflinks	exon	33468506	33468564	-	XLOC 03223	TCONS 0014983	18	NOL6	NM 022917	NM 022917	=	TSS60732	P33987
chr9	Cufflinks	exon	33468750	33468870	-	XLOC 03223	TCONS 0014983	19	NOL6	NM 022917	NM 022917	=	TSS60732	P33987
chr9	Cufflinks	exon	33468956	33469119	-	XLOC 03223	TCONS 0014983	20	NOL6	NM 022917	NM 022917	=	TSS60732	P33987
chr9	Cufflinks	exon	33469205	33469339	-	XLOC 03223	TCONS 0014983	21	NOL6	NM 022917	NM 022917	=	TSS60732	P33987
chr9	Cufflinks	exon	33469497	33469665	-	XLOC 03223	TCONS 0014983	22	NOL6	NM 022917	NM 022917	=	TSS60732	P33987
chr9	Cufflinks	exon	33470010	33470189	-	XLOC 03223	TCONS 0014983	23	NOL6	NM 022917	NM 022917	=	TSS60732	P33987
chr9	Cufflinks	exon	33472002	33472118	-	XLOC 03223	TCONS 0014983	24	NOL6	NM 022917	NM 022917	=	TSS60732	P33987
chr9	Cufflinks	exon	33472204	33472410	-	XLOC 03223	TCONS 0014983	25	NOL6	NM 022917	NM 022917	=	TSS60732	P33987
chr9	Cufflinks	exon	33473787	33473941	-	XLOC 03223	TCONS 0014983	26	NOL6	NM 022917	NM 022917	=	TSS60732	P33987
chr9	Cufflinks	exon	34366664	34373004	-	XLOC 03224	TCONS 0014987	1	KIAA1161	NM 020702	NM 020702	=	TSS60745	P33994

chr9	Cufflinks	exon	34376791	34376894	-	XLOC 03224	TCONS 0014987	2	KIAA1161	NM 020702	NM 020702	=	TSS60745	P33994
chr9	Cufflinks	exon	110247133	110248207	-	XLOC 03249	TCONS 0015108	1	KLF4	NM 004235	NM 004235	=	TSS61232	P34252
chr9	Cufflinks	exon	110249309	110249473	-	XLOC 03249	TCONS 0015108	2	KLF4	NM 004235	NM 004235	=	TSS61232	P34252
chr9	Cufflinks	exon	110249576	110250548	-	XLOC 03249	TCONS 0015108	3	KLF4	NM 004235	NM 004235	=	TSS61232	P34252
chr9	Cufflinks	exon	110251211	110251331	-	XLOC 03249	TCONS 0015108	4	KLF4	NM 004235	NM 004235	=	TSS61232	P34252
chr9	Cufflinks	exon	110251449	110252047	-	XLOC 03249	TCONS 0015108	5	KLF4	NM 004235	NM 004235	=	TSS61232	P34252
chr9	Cufflinks	exon	112002007	112004089	-	XLOC 03250	TCONS 0015111	1	EPB41L4B	NM 018424	NM 018424	=	TSS61242	P34260
chr9	Cufflinks	exon	112005898	112005962	-	XLOC 03250	TCONS 0015111	2	EPB41L4B	NM 018424	NM 018424	=	TSS61242	P34260
chr9	Cufflinks	exon	112010036	112010069	-	XLOC 03250	TCONS 0015111	3	EPB41L4B	NM 018424	NM 018424	=	TSS61242	P34260
chr9	Cufflinks	exon	112013761	112013791	-	XLOC 03250	TCONS 0015111	4	EPB41L4B	NM 018424	NM 018424	=	TSS61242	P34260
chr9	Cufflinks	exon	112015721	112015830	-	XLOC 03250	TCONS 0015111	5	EPB41L4B	NM 018424	NM 018424	=	TSS61242	P34260
chr9	Cufflinks	exon	112017791	112017960	-	XLOC 03250	TCONS 0015111	6	EPB41L4B	NM 018424	NM 018424	=	TSS61242	P34260
chr9	Cufflinks	exon	112018054	112018123	-	XLOC 03250	TCONS 0015111	7	EPB41L4B	NM 018424	NM 018424	=	TSS61242	P34260
chr9	Cufflinks	exon	112018416	112018504	-	XLOC 03250	TCONS 0015111	8	EPB41L4B	NM 018424	NM 018424	=	TSS61242	P34260
chr9	Cufflinks	exon	112018673	112018760	-	XLOC 03250	TCONS 0015111	9	EPB41L4B	NM 018424	NM 018424	=	TSS61242	P34260
chr9	Cufflinks	exon	112020457	112020577	-	XLOC 03250	TCONS 0015111	10	EPB41L4B	NM 018424	NM 018424	=	TSS61242	P34260
chr9	Cufflinks	exon	112025330	112025382	-	XLOC 03250	TCONS 0015111	11	EPB41L4B	NM 018424	NM 018424	=	TSS61242	P34260
chr9	Cufflinks	exon	112027260	112027304	-	XLOC 03250	TCONS 0015111	12	EPB41L4B	NM 018424	NM 018424	=	TSS61242	P34260
chr9	Cufflinks	exon	112029753	112029831	-	XLOC 03250	TCONS 0015111	13	EPB41L4B	NM 018424	NM 018424	=	TSS61242	P34260
chr9	Cufflinks	exon	112030671	112030713	-	XLOC 03250	TCONS 0015111	14	EPB41L4B	NM 018424	NM 018424	=	TSS61242	P34260
chr9	Cufflinks	exon	112042097	112042201	-	XLOC 03250	TCONS 0015111	15	EPB41L4B	NM 018424	NM 018424	=	TSS61242	P34260
chr9	Cufflinks	exon	112082421	112083244	-	XLOC 03250	TCONS 0015111	16	EPB41L4B	NM 018424	NM 018424	=	TSS61242	P34260
chr9	Cufflinks	exon	139567595	139568379	-	XLOC 03270	TCONS 0015234	1	AGPAT2	NM 006412	NM 006412	=	TSS61683	P34529
chr9	Cufflinks	exon	139569187	139569259	-	XLOC 03270	TCONS 0015234	2	AGPAT2	NM 006412	NM 006412	=	TSS61683	P34529
chr9	Cufflinks	exon	139571037	139571132	-	XLOC 03270	TCONS 0015234	3	AGPAT2	NM 006412	NM 006412	=	TSS61683	P34529
chr9	Cufflinks	exon	139571413	139571588	-	XLOC 03270	TCONS 0015234	4	AGPAT2	NM 006412	NM 006412	=	TSS61683	P34529
chr9	Cufflinks	exon	139571875	139572008	-	XLOC 03270	TCONS 0015234	5	AGPAT2	NM 006412	NM 006412	=	TSS61683	P34529
chr9	Cufflinks	exon	139581628	139581911	-	XLOC 03270	TCONS 0015234	6	AGPAT2	NM 006412	NM 006412	=	TSS61683	P34529
chr9	Cufflinks	exon	140086069	140086626	-	XLOC 03273	TCONS 0015245	1	TPRN	NM 001128228	NM 001128228	=	TSS61730	P34553
chr9	Cufflinks	exon	140086711	140086817	-	XLOC 03273	TCONS 0015245	2	TPRN	NM 001128228	NM 001128228	=	TSS61730	P34553
chr9	Cufflinks	exon	140086903	140087143	-	XLOC 03273	TCONS 0015245	3	TPRN	NM 001128228	NM 001128228	=	TSS61730	P34553
chr9	Cufflinks	exon	140093439	140095163	-	XLOC 03273	TCONS 0015245	4	TPRN	NM 001128228	NM 001128228	=	TSS61730	P34553
chrX	Cufflinks	exon	43514155	43515662	+	XLOC 03315	TCONS 0015345	1	MAOA	NM 000240	NM 000240	=	TSS62295	P34739
chrX	Cufflinks	exon	43542761	43542855	+	XLOC 03315	TCONS 0015345	2	MAOA	NM 000240	NM 000240	=	TSS62295	P34739
chrX	Cufflinks	exon	43552538	43552675	+	XLOC 03315	TCONS 0015345	3	MAOA	NM 000240	NM 000240	=	TSS62295	P34739
chrX	Cufflinks	exon	43571119	43571223	+	XLOC 03315	TCONS 0015345	4	MAOA	NM 000240	NM 000240	=	TSS62295	P34739
chrX	Cufflinks	exon	43571952	43572043	+	XLOC 03315	TCONS 0015345	5	MAOA	NM 000240	NM 000240	=	TSS62295	P34739
chrX	Cufflinks	exon	43587420	43587561	+	XLOC 03315	TCONS 0015345	6	MAOA	NM 000240	NM 000240	=	TSS62295	P34739
chrX	Cufflinks	exon	43590488	43590637	+	XLOC 03315	TCONS 0015345	7	MAOA	NM 000240	NM 000240	=	TSS62295	P34739
chrX	Cufflinks	exon	43590941	43591100	+	XLOC 03315	TCONS 0015345	8	MAOA	NM 000240	NM 000240	=	TSS62295	P34739
chrX	Cufflinks	exon	43591946	43592042	+	XLOC 03315	TCONS 0015345	9	MAOA	NM 000240	NM 000240	=	TSS62295	P34739
chrX	Cufflinks	exon	43595474	43595527	+	XLOC 03315	TCONS 0015345	10	MAOA	NM 000240	NM 000240	=	TSS62295	P34739
chrX	Cufflinks	exon	43599928	43599985	+	XLOC 03315	TCONS 0015345	11	MAOA	NM 000240	NM 000240	=	TSS62295	P34739
chrX	Cufflinks	exon	43601197	43601294	+	XLOC 03315	TCONS 0015345	12	MAOA	NM 000240	NM 000240	=	TSS62295	P34739
chrX	Cufflinks	exon	43603041	43603152	+	XLOC 03315	TCONS 0015345	13	MAOA	NM 000240	NM 000240	=	TSS62295	P34739
chrX	Cufflinks	exon	43603356	43603418	+	XLOC 03315	TCONS 0015345	14	MAOA	NM 000240	NM 000240	=	TSS62295	P34739
chrX	Cufflinks	exon	43603614	43606071	+	XLOC 03315	TCONS 0015345	15	MAOA	NM 000240	NM 000240	=	TSS62295	P34739
chrX	Cufflinks	exon	106161590	106161699	+	XLOC 03339	TCONS 0015458	1	CLDN2	NM 001171095	NM 001171095	=	TSS62706	P35040
chrX	Cufflinks	exon	106171281	106174091	+	XLOC 03339	TCONS 0015458	2	CLDN2	NM 001171095	NM 001171095	=	TSS62706	P35040
chrX	Cufflinks	exon	153991017	153991256	+	XLOC 03358	TCONS 0015545	1	DKC1	NM 001363	NM 001363	=	TSS63054	P35287
chrX	Cufflinks	exon	153993174	153993241	+	XLOC 03358	TCONS 0015545	2	DKC1	NM 001363	NM 001363	=	TSS63054	P35287
chrX	Cufflinks	exon	153993719	153993805	+	XLOC 03358	TCONS 0015545	3	DKC1	NM 001363	NM 001363	=	TSS63054	P35287
chrX	Cufflinks	exon	153994182	153994273	+	XLOC 03358	TCONS 0015545	4	DKC1	NM 001363	NM 001363	=	TSS63054	P35287
chrX	Cufflinks	exon	153994491	153994675	+	XLOC 03358	TCONS 0015545	5	DKC1	NM 001363	NM 001363	=	TSS63054	P35287
chrX	Cufflinks	exon	153995272	153995336	+	XLOC 03358	TCONS 0015545	6	DKC1	NM 001363	NM 001363	=	TSS63054	P35287
chrX	Cufflinks	exon	153995531	153995657	+	XLOC 03358	TCONS 0015545	7	DKC1	NM 001363	NM 001363	=	TSS63054	P35287
chrX	Cufflinks	exon	153996577	153996707	+	XLOC 03358	TCONS 0015545	8	DKC1	NM 001363	NM 001363	=	TSS63054	P35287
chrX	Cufflinks	exon	153997442	153997585	+	XLOC 03358	TCONS 0015545	9	DKC1	NM 001363	NM 001363	=	TSS63054	P35287
chrX	Cufflinks	exon	153999034	153999154	+	XLOC 03358	TCONS 0015545	10	DKC1	NM 001363	NM 001363	=	TSS63054	P35287
chrX	Cufflinks	exon	154001406	154001524	+	XLOC 03358	TCONS 0015545	11	DKC1	NM 001363	NM 001363	=	TSS63054	P35287
chrX	Cufflinks	exon	154002877	154002980	+	XLOC 03358	TCONS 0015545	12	DKC1	NM 001363	NM 001363	=	TSS63054	P35287
chrX	Cufflinks	exon	154003470	154003548	+	XLOC 03358	TCONS 0015545	13	DKC1	NM 001363	NM 001363	=	TSS63054	P35287
chrX	Cufflinks	exon	154004462	154004599	+	XLOC 03358	TCONS 0015545	14	DKC1	NM 001363	NM 001363	=	TSS63054	P35287
chrX	Cufflinks	exon	154005074	154005964	+	XLOC 03358	TCONS 0015545	15	DKC1	NM 001363	NM 001363	=	TSS63054	P35287
chrX	Cufflinks	exon	147133675	147134430	.	XLOC 03432	TCONS 0015827	1		CUFF.43237.1		u	TSS64228	

Table S8.

The fusion genes selected for RT-PCR and Sanger sequencing validation

# Patient	11	43		53	81
**Gene 1 symbol (5end fusion partner)**	EEF1A1	EEF1A1		KRT19	KRT19
**Gene 2 symbol (3end fusion partner)**	HSP90AB1	HSP90AB1		KRT18	KRT18
**Fusion description**	oncogene,cancer,m3	oncogene,cancer,m2		exon-exon	exon-exon
**Counts of common mapping reads**	0	0		0	0
**Spanning pairs**	4	5		2	2
**Spanning unique reads**	1	1		3	3
**Longest anchor found**	47	47		20	20
**Fusion finding method**	BOWTIE+BLAT	BOWTIE+STAR		BOWTIE	BOWTIE
**Fusion point for gene 1 (5end fusion partner)**	6:73519944:-	6:73519891:-		17:41524843:-	17:41524843:-
**Fusion point for gene 2 (3end fusion partner)**	6:44253176:+	6:44250304:+		12:52951481:+	12:52951481:+
**Gene 1 id (5end fusion partner)**	ENSG00000156508	ENSG00000156508		ENSG00000171345	ENSG00000171345
**Gene 2 id (3end fusion partner)**	ENSG00000096384	ENSG00000096384		ENSG00000111057	ENSG00000111057
**Exon 1 id (5end fusion partner)**				ENSE00003539860	ENSE00003539860
**Exon 2 id (3end fusion partner)**				ENSE00003502582	ENSE00003502582
**Fusion sequence**	GTCATTGGACACGTA<br>GATTCGGGCAAGTCC<br>ACCACTACTGGCCATC<br>TGAT*GGCCAAAAAG<br>CACCTGGAGATCAAC<br>CCTGACCACCCCATTG<br>TGGAGACGC	TAAATGCGGTGGCAT<br>CGACAAAAGAACCAT<br>TGAAAAATTTGAGAA<br>GGAGG*GAGAGAAGG<br>AAATTAGTGATGATG<br>AGGCAGAGGAAGAG<br>AAAGGTGAGAAA		CCTGAAGGAAGAGCT<br>GGCCTACCTGAAGAA<br>GAACCATGAGGAG*G<br>AAGTAAAAGGCCTAC<br>AAGCCCAGATTGCCA<br>GCTCTGGGTTGA	CCTGAAGGAAGAGCT<br>GGCCTACCTGAAGAA<br>GAACCATGAGGAG*G<br>AAGTAAAAGGCCTAC<br>AAGCCCAGATTGCCA<br>GCTCTGGGTTGA
**Predicted effect**	in-frame	in-frame		in-frame	in-frame
